# Supplementary material for: Public-facing health information on achalasia generated by large language models: a multidimensional comparative study
Source: Front Public Health. 2026 Sep 15;14:1962232. doi: 10.3389/fpubh.2026.1962232 (PMC13619586; doi:10.3389/fpubh.2026.1962232)
Supplement: Supplementary file 1 [file supplementary_file_1.docx]

**Supplementary Material 1: Screening of 100 candidate public questions related to achalasia.**

| Pool order | Dimension | Question | Decision | Final item ID | Screening reason |
| --- | --- | --- | --- | --- | --- |
| 1 | Disease understanding | What is achalasia? | Retained | 1 | Retained in the final 40-question set; directly addresses disease understanding. |
| 2 | Disease understanding | What is the exact ICD-10 code for achalasia? | Excluded | - | Excluded: administrative coding question rather than patient-facing clinical education. |
| 3 | Disease understanding | Can achalasia be diagnosed from a social-media symptom checklist? | Excluded | - | Excluded: unreliable source-framing question; retained items cover symptom recognition and formal diagnosis. |
| 4 | Disease understanding | Is achalasia the same as acid reflux? | Retained | 2 | Retained in the final 40-question set; directly addresses disease understanding. |
| 5 | Disease understanding | Can anxiety medicine cure achalasia if stress triggered it? | Excluded | - | Excluded: unsafe medication/cure premise and overlaps with retained stress/anxiety item. |
| 6 | Disease understanding | If my swallowing improves for a few days, can I wait a year before seeing a doctor? | Excluded | - | Excluded: individualized delay-of-care question with potential safety risk. |
| 7 | Disease understanding | Is achalasia caused by stress or anxiety? | Retained | 3 | Retained in the final 40-question set; directly addresses disease understanding. |
| 8 | Disease understanding | Can achalasia turn into esophageal cancer within one month? | Excluded | - | Excluded: unrealistic cancer-timeline variant; retained cancer-risk items cover clinically relevant framing. |
| 9 | Symptoms and red flags | What home test can confirm that my swallowing problem is achalasia? | Excluded | - | Excluded: self-diagnosis question; final set focuses on appropriate clinical testing. |
| 10 | Disease understanding | Can achalasia go away on its own? | Retained | 4 | Retained in the final 40-question set; directly addresses disease understanding. |
| 11 | Symptoms and red flags | Food got stuck once after a large meal. Does this mean I definitely have achalasia? | Excluded | - | Excluded: highly individualized one-off symptom question. |
| 12 | Symptoms and red flags | Can I make myself vomit to clear food stuck in my chest? | Excluded | - | Excluded: unsafe self-management prompt. |
| 13 | Disease understanding | Is achalasia a type of cancer? | Retained | 5 | Retained in the final 40-question set; directly addresses disease understanding. |
| 14 | Symptoms and red flags | Can chest pain from achalasia be ignored if I am young? | Excluded | - | Excluded: unsafe reassurance premise; retained chest-pain item covers general education. |
| 15 | Symptoms and red flags | Can I manage achalasia-related weight loss by drinking only protein shakes instead of seeing a doctor? | Excluded | - | Excluded: unsafe overreliance on diet and delayed evaluation. |
| 16 | Symptoms and red flags | What symptoms are common in achalasia? | Retained | 6 | Retained in the final 40-question set; directly addresses symptoms and red flags. |
| 17 | Symptoms and red flags | Should I sleep flat to train my esophagus to empty better? | Excluded | - | Excluded: unsafe lifestyle premise and lower priority than nocturnal choking/aspiration questions. |
| 18 | Symptoms and red flags | Does trouble swallowing only large pills mean I have achalasia? | Excluded | - | Excluded: narrow pill-dysphagia variant; final set covers typical solids-and-liquids dysphagia. |
| 19 | Symptoms and red flags | I feel food stuck in my chest when swallowing. Could this be achalasia? | Retained | 7 | Retained in the final 40-question set; directly addresses symptoms and red flags. |
| 20 | Diagnosis and testing | Which brand of esophageal manometry catheter is best? | Excluded | - | Excluded: device-specific technical question outside the patient-facing prompt set. |
| 21 | Diagnosis and testing | Can I skip manometry if I dislike nasal tubes? | Excluded | - | Excluded: individualized diagnostic-refusal question requiring clinician discussion. |
| 22 | Symptoms and red flags | Can achalasia cause regurgitation of undigested food? | Retained | 8 | Retained in the final 40-question set; directly addresses symptoms and red flags. |
| 23 | Diagnosis and testing | Can gastroscopy tell me the exact Chicago subtype of achalasia? | Excluded | - | Excluded: diagnostic misconception and duplicate of retained gastroscopy/manometry items. |
| 24 | Diagnosis and testing | What are the exact Chicago Classification pressure equations for achalasia? | Excluded | - | Excluded: overly technical clinician/researcher-facing question. |
| 25 | Symptoms and red flags | Can achalasia cause chest pain? | Retained | 9 | Retained in the final 40-question set; directly addresses symptoms and red flags. |
| 26 | Diagnosis and testing | Can I read my own barium swallow image and decide whether it is achalasia? | Excluded | - | Excluded: individualized imaging interpretation question. |
| 27 | Diagnosis and testing | Can a normal endoscopy prove that cancer is impossible forever? | Excluded | - | Excluded: overly absolute and misleading diagnostic claim. |
| 28 | Symptoms and red flags | Can achalasia cause weight loss? | Retained | 10 | Retained in the final 40-question set; directly addresses symptoms and red flags. |
| 29 | Diagnosis and testing | My gastroscopy was normal five years ago. Does that mean I never need more testing? | Excluded | - | Excluded: individualized time-dependent diagnostic question. |
| 30 | Differential diagnosis | Which proton pump inhibitor is strongest for achalasia? | Excluded | - | Excluded: reflux-medication framing and overlaps with GERD/misdiagnosis items. |
| 31 | Symptoms and red flags | Can achalasia cause coughing or choking at night? | Retained | 11 | Retained in the final 40-question set; directly addresses symptoms and red flags. |
| 32 | Differential diagnosis | Can esophageal cancer be ruled out from symptoms alone? | Excluded | - | Excluded: unsafe diagnostic premise; final set covers need to rule out cancer clinically. |
| 33 | Differential diagnosis | Should I keep taking reflux medicine for six more months before asking for achalasia testing? | Excluded | - | Excluded: individualized self-directed delay of evaluation. |
| 34 | Symptoms and red flags | Can achalasia make swallowing both liquids and solids difficult? | Retained | 12 | Retained in the final 40-question set; directly addresses symptoms and red flags. |
| 35 | Treatment overview and selection | Can acupuncture cure achalasia better than standard treatment? | Excluded | - | Excluded: unsupported alternative-treatment replacement question. |
| 36 | Treatment overview and selection | Which online pharmacy sells nifedipine for achalasia? | Excluded | - | Excluded: unsafe medication-purchase prompt. |
| 37 | Diagnosis and testing | How is achalasia diagnosed? | Retained | 13 | Retained in the final 40-question set; directly addresses diagnosis and testing. |
| 38 | Treatment overview and selection | Is POEM always better than every other treatment for achalasia? | Excluded | - | Excluded: overgeneralized treatment-ranking variant; retained item asks which treatment is best more broadly. |
| 39 | Treatment overview and selection | Can I choose achalasia treatment only by the shortest hospital stay? | Excluded | - | Excluded: oversimplified individualized treatment-selection question. |
| 40 | Diagnosis and testing | What tests are needed to confirm achalasia? | Retained | 14 | Retained in the final 40-question set; directly addresses diagnosis and testing. |
| 41 | POEM and post-procedure care | Can POEM be done in a small outpatient clinic without hospital backup? | Excluded | - | Excluded: unsafe procedural-setting question. |
| 42 | POEM and post-procedure care | Can POEM be done for everyone with any swallowing problem? | Excluded | - | Excluded: overbroad indication question; retained item covers POEM suitability. |
| 43 | Diagnosis and testing | Can gastroscopy alone diagnose achalasia? | Retained | 15 | Retained in the final 40-question set; directly addresses diagnosis and testing. |
| 44 | POEM and post-procedure care | What exact myotomy length should I request from the doctor? | Excluded | - | Excluded: clinician-level individualized procedural planning question. |
| 45 | POEM and post-procedure care | Should I stop acid-reflux medicine immediately after POEM? | Excluded | - | Excluded: individualized medication decision after a procedure. |
| 46 | Diagnosis and testing | What is the role of high-resolution esophageal manometry in achalasia? | Retained | 16 | Retained in the final 40-question set; directly addresses diagnosis and testing. |
| 47 | POEM and post-procedure care | Can I eat steak the day after POEM if I chew carefully? | Excluded | - | Excluded: unsafe post-procedure diet-advancement prompt. |
| 48 | Treatment modalities | Can Heller myotomy be done without endoscopy or manometry first? | Excluded | - | Excluded: unsafe testing-avoidance premise. |
| 49 | Diagnosis and testing | What does a barium swallow show in achalasia? | Retained | 17 | Retained in the final 40-question set; directly addresses diagnosis and testing. |
| 50 | Treatment modalities | Which surgeon or hospital in my city is best for POEM? | Excluded | - | Excluded: local service recommendation outside clinical content. |
| 51 | Treatment modalities | Can pneumatic dilation be repeated every month until achalasia is cured? | Excluded | - | Excluded: unsafe overuse and individualized procedural planning. |
| 52 | Diagnosis and testing | Why do doctors need to rule out esophageal cancer before diagnosing achalasia? | Retained | 18 | Retained in the final 40-question set; directly addresses diagnosis and testing. |
| 53 | Treatment modalities | Can I drive home alone immediately after pneumatic dilation? | Excluded | - | Excluded: procedure-day instruction requiring local anesthesia and discharge protocol. |
| 54 | Treatment modalities | Can Botox for achalasia be injected at a beauty clinic? | Excluded | - | Excluded: unsafe nonmedical setting and procedure misunderstanding. |
| 55 | Diagnosis and testing | Can a normal gastroscopy rule out achalasia? | Retained | 19 | Retained in the final 40-question set; directly addresses diagnosis and testing. |
| 56 | Follow-up and recurrence | Is persistent dysphagia always normal after achalasia treatment? | Excluded | - | Excluded: unsafe reassurance variant; retained item covers dysphagia after treatment. |
| 57 | Follow-up and long-term risk | Should I ignore fever after POEM if the pain is mild? | Excluded | - | Excluded: unsafe post-procedure red-flag prompt. |
| 58 | Differential diagnosis | How is achalasia different from gastroesophageal reflux disease? | Retained | 20 | Retained in the final 40-question set; directly addresses differential diagnosis. |
| 59 | Follow-up and long-term risk | How often exactly must every patient have endoscopy to screen for cancer? | Excluded | - | Excluded: overgeneralized surveillance question; retained item covers cancer risk broadly. |
| 60 | Diet and lifestyle | Can I cure achalasia by eating only soft foods? | Excluded | - | Excluded: unsafe overreliance on diet; retained diet item focuses on supportive dietary advice. |
| 61 | Differential diagnosis | How is achalasia different from esophageal cancer? | Retained | 21 | Retained in the final 40-question set; directly addresses differential diagnosis. |
| 62 | Special populations | Can children inherit achalasia directly from their parents? | Excluded | - | Excluded: special-population/genetics variant not prioritized for the final adult public set. |
| 63 | Differential diagnosis | Can achalasia be misdiagnosed as reflux disease? | Retained | 22 | Retained in the final 40-question set; directly addresses differential diagnosis. |
| 64 | Disease understanding | Can achalasia spread between family members by sharing food? | Excluded | - | Excluded: very low-priority misconception. |
| 65 | Treatment overview and selection | What are the main treatments for achalasia? | Retained | 23 | Retained in the final 40-question set; directly addresses treatment overview and selection. |
| 66 | Diagnosis and testing | Can a smartwatch or phone app diagnose achalasia? | Excluded | - | Excluded: unsupported digital self-diagnosis tool question. |
| 67 | Treatment overview and selection | Can medicine cure achalasia? | Retained | 24 | Retained in the final 40-question set; directly addresses treatment overview and selection. |
| 68 | Disease understanding | Can spicy food cause permanent achalasia? | Excluded | - | Excluded: lower-priority causal misconception and overlaps with disease-understanding items. |
| 69 | Treatment overview and selection | Which treatment is best for achalasia? | Retained | 25 | Retained in the final 40-question set; directly addresses treatment overview and selection. |
| 70 | Diet and lifestyle | Is coffee the main cause of achalasia? | Excluded | - | Excluded: lower-priority diet-cause misconception. |
| 71 | Treatment overview and selection | Can achalasia be treated without surgery? | Retained | 26 | Retained in the final 40-question set; directly addresses treatment overview and selection. |
| 72 | Diet and lifestyle | Can carbonated drinks force food through the lower esophageal sphincter? | Excluded | - | Excluded: potentially unsafe self-management strategy. |
| 73 | POEM and post-procedure care | What is POEM for achalasia? | Retained | 27 | Retained in the final 40-question set; directly addresses poem and post-procedure care. |
| 74 | Diet and lifestyle | Can I crush all my medicines if swallowing is hard? | Excluded | - | Excluded: individualized medication-safety question requiring clinician or pharmacist review. |
| 75 | POEM and post-procedure care | Who is suitable for POEM? | Retained | 28 | Retained in the final 40-question set; directly addresses poem and post-procedure care. |
| 76 | Special populations | Can pregnancy with achalasia be managed at home without doctors? | Excluded | - | Excluded: individualized high-risk special-population question. |
| 77 | POEM and post-procedure care | What are the risks of POEM? | Retained | 29 | Retained in the final 40-question set; directly addresses poem and post-procedure care. |
| 78 | Alternative treatment | Can massage or chiropractic adjustment open the lower esophageal sphincter? | Excluded | - | Excluded: unsupported alternative-treatment claim. |
| 79 | POEM and post-procedure care | Does POEM cause acid reflux afterward? | Retained | 30 | Retained in the final 40-question set; directly addresses poem and post-procedure care. |
| 80 | Diagnosis and testing | Can I avoid endoscopy because I am afraid of anesthesia? | Excluded | - | Excluded: individualized diagnostic avoidance question requiring clinician discussion. |
| 81 | POEM and post-procedure care | What should I eat after POEM? | Retained | 31 | Retained in the final 40-question set; directly addresses poem and post-procedure care. |
| 82 | Treatment overview and selection | Should I request esophagectomy early to prevent cancer? | Excluded | - | Excluded: extreme individualized treatment request not suitable for general public prompt set. |
| 83 | Treatment modalities | What is laparoscopic Heller myotomy? | Retained | 32 | Retained in the final 40-question set; directly addresses treatment modalities. |
| 84 | Disease mechanism | Is achalasia an autoimmune disease that should be treated with steroids? | Excluded | - | Excluded: misleading treatment premise and unsafe self-medication risk. |
| 85 | Treatment modalities | What is the difference between POEM and Heller myotomy? | Retained | 33 | Retained in the final 40-question set; directly addresses treatment modalities. |
| 86 | Diagnosis and testing | Can a blood test confirm achalasia? | Excluded | - | Excluded: diagnostic misconception covered by retained testing items. |
| 87 | Treatment modalities | What is pneumatic dilation for achalasia? | Retained | 34 | Retained in the final 40-question set; directly addresses treatment modalities. |
| 88 | Medication safety | Can I mix herbal remedies with nitrates for achalasia? | Excluded | - | Excluded: individualized drug-interaction and unsafe self-medication question. |
| 89 | Treatment modalities | Is pneumatic dilation safe? | Retained | 35 | Retained in the final 40-question set; directly addresses treatment modalities. |
| 90 | Diet and lifestyle | Which brand of blender is best for achalasia diets? | Excluded | - | Excluded: commercial/product-specific question outside clinical evaluation. |
| 91 | Treatment modalities | What is botulinum toxin injection for achalasia? | Retained | 36 | Retained in the final 40-question set; directly addresses treatment modalities. |
| 92 | Diet and lifestyle | Can I fast for a week to rest my esophagus? | Excluded | - | Excluded: unsafe dietary-restriction prompt. |
| 93 | Follow-up and long-term risk | Why do I still have swallowing difficulty after achalasia treatment? | Retained | 37 | Retained in the final 40-question set; directly addresses follow-up and long-term risk. |
| 94 | Differential diagnosis | Is achalasia the same as a hiatal hernia? | Excluded | - | Excluded: lower-priority differential variant; retained set focuses on GERD and cancer comparisons. |
| 95 | Follow-up and long-term risk | What symptoms after POEM or surgery should make me seek urgent medical care? | Retained | 38 | Retained in the final 40-question set; directly addresses follow-up and long-term risk. |
| 96 | Treatment overview and selection | Can I choose pneumatic dilation just because it is the cheapest treatment? | Excluded | - | Excluded: cost-only and oversimplified treatment-selection question. |
| 97 | Follow-up and long-term risk | Does achalasia increase the risk of esophageal cancer? | Retained | 39 | Retained in the final 40-question set; directly addresses follow-up and long-term risk. |
| 98 | Diagnosis and treatment decision | Can an online calculator decide my Eckardt score and choose treatment for me? | Excluded | - | Excluded: individualized decision-support prompt beyond general health education. |
| 99 | Diet and lifestyle | What should people with achalasia eat? | Retained | 40 | Retained in the final 40-question set; directly addresses diet and lifestyle. |
| 100 | Diet and lifestyle | Can drinking alcohol relax the sphincter and improve achalasia? | Excluded | - | Excluded: unsafe lifestyle/self-treatment premise. |
