# Supplementary material for: Public-facing health information on achalasia generated by large language models: a multidimensional comparative study
Source: Front Public Health. 2026 Sep 15;14:1962232. doi: 10.3389/fpubh.2026.1962232 (PMC13619586; doi:10.3389/fpubh.2026.1962232)
Supplement: Supplementary file 2 [file supplementary_file_2.docx]

**Structured records for the 40 questions by the six models**

**Q1. What is achalasia?**

**Model ChatGPT 5.5**

Answer text:

Achalasia is a rare disorder of the esophagus-the tube that carries food and liquid from your mouth to your stomach.

In achalasia, two main things happen:

The lower esophageal sphincter does not relax properly This is the muscular valve between the esophagus and stomach. Normally it opens when you swallow. In achalasia, it stays too tight.

The esophagus loses normal squeezing motion The muscles of the esophagus do not push food downward effectively.

As a result, food and liquids can have trouble passing into the stomach.

Common symptoms include:

Difficulty swallowing, especially with both solids and liquids

Regurgitation of undigested food or fluid

Chest pain or pressure

Heartburn-like symptoms

Coughing or choking, especially at night

Weight loss in more advanced cases

Achalasia is usually caused by damage to or loss of nerve cells in the esophagus that control muscle movement. The exact reason this happens is often unknown.

Diagnosis may involve tests such as:

Esophageal manometry, which measures muscle pressure and movement

Barium swallow X-ray, which shows how liquid moves through the esophagus

Upper endoscopy, to rule out other causes such as narrowing or cancer

Treatment does not usually "cure" the nerve problem, but it can help food pass into the stomach by weakening or opening the tight lower sphincter. Options include:

Pneumatic balloon dilation

Surgical myotomy, such as Heller myotomy

POEM, or peroral endoscopic myotomy

Botox injections in selected patients

Medications, though these are generally less effective

Achalasia is not the same as ordinary acid reflux, though it can sometimes feel similar. If someone has progressive trouble swallowing, especially both liquids and solids, they should be evaluated by a gastroenterologist.

A useful next step would be to discuss how achalasia is diagnosed and how it differs from GERD or esophageal cancer.

**Model Claude Opus 4.7**

Answer text:

Achalasia

Achalasia is a rare disorder of the esophagus (the muscular tube that carries food from the mouth to the stomach) that makes it difficult for food and liquid to pass into the stomach.

What Happens

The condition involves two main problems:

The lower esophageal sphincter (LES) - a muscular ring at the bottom of the esophagus - fails to relax properly to let food pass into the stomach.

The esophageal muscles lose their ability to contract in a coordinated way (peristalsis), so food isn't pushed downward effectively.

Cause

The exact cause is unknown, but it results from damage to or loss of nerve cells in the esophagus. Possible factors include:

Autoimmune response

Viral infection

Genetic predisposition

In rare cases, conditions like Chagas disease can cause similar symptoms

Symptoms

Dysphagia (difficulty swallowing solids and liquids)

Regurgitation of undigested food

Chest pain or discomfort

Heartburn

Weight loss

Coughing at night or aspiration of food into the lungs

Diagnosis

Esophageal manometry (measures muscle pressure) - the gold standard

Barium swallow X-ray - often shows a "bird's beak" appearance

Endoscopy - to rule out other conditions like cancer

Treatment

There is no cure, but treatments help manage symptoms:

Pneumatic dilation - stretching the LES with a balloon

Heller myotomy - surgery to cut the LES muscle

POEM (peroral endoscopic myotomy) - minimally invasive endoscopic procedure

Botulinum toxin injections - relaxes the sphincter (often used in patients who can't have surgery)

Medications - such as calcium channel blockers or nitrates (less effective)

Outlook

With proper treatment, most people experience significant symptom relief, though long-term monitoring is needed since achalasia slightly increases the risk of esophageal cancer.

If you suspect you have symptoms of achalasia, it's important to consult a gastroenterologist for evaluation.

**Model DeepSeek V4 Pro**

Answer text:

Achalasia is a rare esophageal motility disorder that makes it difficult for food and liquid to pass into the stomach. It happens when nerve cells in the esophagus (specifically in the myenteric plexus) degenerate for reasons that are still not fully understood, leading to two main problems:

Failure of the lower esophageal sphincter (LES) to relax during swallowing.

Loss of normal peristalsis (the rhythmic muscle contractions that push food down).

As a result, the LES remains tightly closed, and the esophagus above it can become dilated and filled with retained food or saliva.

Key symptoms

Progressive dysphagia - trouble swallowing both solids and liquids (usually starts with solids, but often progresses to liquids).

Regurgitation of undigested food or saliva, especially at night, which can lead to aspiration (food entering the lungs).

Chest pain or discomfort, which may be mistaken for heart pain.

Heartburn (though it's not caused by acid reflux but by retained food fermented in the esophagus).

Unintentional weight loss due to difficulty eating.

How is it diagnosed?

Because symptoms mimic those of GERD or esophageal cancer, proper testing is essential:

High-resolution manometry - the gold standard; it measures pressure in the esophagus and directly shows failed LES relaxation and absent peristalsis.

Barium swallow (esophagram) - often reveals a classic "bird's beak" narrowing at the LES and a dilated esophagus.

Upper endoscopy - mainly to rule out pseudoachalasia (e.g., a tumor at the gastroesophageal junction causing a similar picture).

Treatment options (and risk considerations)

No treatment can restore the lost nerves, so therapy aims to mechanically disrupt the tight LES.

Treatment How it works Key risks / considerations

Pneumatic dilation A balloon is inflated to tear the muscle fibers of the LES. Risk of esophageal perforation ( about 2-3 percent ) requiring urgent surgery. Often repeated. Provides good short-to-medium-term relief.

Laparoscopic Heller myotomy A surgeon cuts the LES muscle. Usually combined with a partial fundoplication to reduce postoperative reflux. Invasive surgery; risk of GERD in up to 10-30 percent of patients even with fundoplication.

Peroral endoscopic myotomy (POEM) An endoscopic procedure that creates a tunnel and cuts the inner muscle layer, similar to Heller but without external incisions. No anti-reflux wrap so reflux can be more common; risk of pneumothorax, bleeding, but perforation risk is low. Excellent symptom relief.

Botulinum toxin (Botox) injection Paralyzes the LES muscle temporarily. Effect lasts only about 6-12 months; repeat injections can cause scarring making future myotomy more difficult. Usually reserved for elderly or high-risk patients.

Medications (e.g., nifedipine, nitrates) Temporarily relax smooth muscle. Low efficacy, used only as a bridge when other options are not available.

Lifestyle optimization: Eat slowly, chew thoroughly, drink water with meals, and avoid eating close to bedtime. Sleeping with the head elevated can reduce nighttime regurgitation.

Potential long-term risks to be aware of

Aspiration pneumonia - from regurgitated material entering the lungs, especially at night.

Esophageal stasis complications - chronic food retention can lead to candida esophagitis or mucosal injury.

Increased risk of esophageal squamous cell carcinoma - studies show a small but real increase in risk in patients with long-standing, untreated achalasia (the stagnant food may cause chronic inflammation). While routine screening endoscopy is not universally recommended, any new or worsening dysphagia, weight loss, or chest pain should trigger prompt evaluation.

Have you or someone you know been dealing with swallowing difficulties, or are you looking into a specific treatment in more detail? I can walk you through what the diagnostic process looks like or compare two options side by side.

**Model Gemini 3.1 Pro Thinking**

Answer text:

Achalasia is a rare, chronic swallowing disorder that affects the esophagus (the tube that carries food from your mouth to your stomach).

In a healthy person, swallowing triggers a coordinated muscle contraction (peristalsis) that pushes food down the esophagus. At the bottom of the esophagus is a muscular ring called the lower esophageal sphincter (LES). This valve relaxes to let food into the stomach and tightens to prevent stomach acid from washing back up.

In someone with achalasia, two main things go wrong due to nerve damage in the esophagus:

Loss of peristalsis: The muscles in the esophagus lose their ability to contract and squeeze food down.

Failure of the LES to relax: The valve at the bottom of the esophagus fails to open properly, causing food and liquid to back up in the esophagus.

Common Symptoms

Dysphagia: A feeling that food or liquid is stuck in your chest after swallowing (the most common symptom).

Regurgitation of undigested food or saliva.

Heartburn or chest pain that may radiate to the back, neck, or arms.

Coughing at night (which can lead to choking or inhaling food into the lungs).

Unexplained weight loss due to difficulty eating.

Causes

The exact cause of achalasia is unknown. Researchers believe it may be an autoimmune condition, where the body's immune system mistakenly attacks the nerves in the muscular layers of the esophagus. It may also be triggered by a severe viral infection in some individuals.

Treatment Options

While there is no cure for achalasia, treatments are highly effective at relieving symptoms by forcing the lower esophageal sphincter open. These include:

Pneumatic dilation: A balloon is inserted into the esophagus and inflated to stretch the sphincter muscle.

Botox injections: Botulinum toxin is injected into the sphincter to paralyze and relax the muscle (usually a temporary fix).

Surgery (Heller myotomy): A surgeon cuts the muscle at the lower end of the esophagus to allow food to pass.

POEM (Peroral Endoscopic Myotomy): A newer, minimally invasive procedure where the surgeon uses an endoscope through the mouth to cut the inner esophageal muscles.

Potential Risk & Health Warning:The information provided here is for educational purposes only and should not be used as a substitute for professional medical advice, diagnosis, or treatment. Untreated achalasia can lead to severe malnutrition, pneumonia (from inhaling food), and a slightly increased risk of esophageal cancer. If you or someone you know is experiencing difficulty swallowing, it is critical to consult a gastroenterologist or medical professional for a proper evaluation.

Would you like to know more about how doctors diagnose this condition (such as using a barium swallow or esophageal manometry), or are you looking for information regarding a specific treatment method?

**Model Grok-4.3**

Answer text:

Achalasia is a rare disorder of the esophagus (the swallowing tube) in which the esophagus loses its ability to push food down into the stomach, and the lower esophageal sphincter (LES) - the valve at the bottom of the esophagus - fails to relax properly.

Key Features of Achalasia

Absent peristalsis: The normal wave-like muscle contractions that move food down the esophagus are lost or severely impaired.

Failure of LES relaxation: The sphincter that should open to let food enter the stomach stays tightly closed.

As a result, food and liquids accumulate in the esophagus, leading to progressive dilation (widening) of the esophagus over time.

Common Symptoms

Dysphagia (difficulty swallowing) - often for both solids and liquids

Regurgitation of undigested food or saliva (sometimes hours after eating)

Chest pain or discomfort (can mimic heartburn or even heart attack)

Heartburn or acid reflux-like symptoms

Unintentional weight loss

Nocturnal coughing or choking (from regurgitation while lying down)

In advanced cases: aspiration pneumonia or malnutrition

Causes

In most cases, achalasia is idiopathic (the exact cause is unknown). It is thought to result from the loss of inhibitory nerve cells (ganglion cells) in the myenteric plexus of the esophagus. Possible contributing factors include:

Autoimmune reaction

Viral infections (e.g., herpes viruses)

Genetic predisposition (rare familial forms exist)

There is also a rare form called pseudoachalasia, which is caused by a tumor or other disease mimicking achalasia.

Diagnosis

Barium swallow (classic "bird-beak" appearance)

High-resolution esophageal manometry (the gold-standard test - confirms absent peristalsis and impaired LES relaxation)

Upper endoscopy (to rule out cancer or pseudoachalasia)

Sometimes CT scan or endoscopic ultrasound

Treatment

There is no cure that restores normal esophageal function, but several effective treatments can relieve symptoms:

Pneumatic dilation - Balloon dilation of the LES

Heller myotomy - Surgical cutting of the LES muscle (often done laparoscopically, frequently combined with an anti-reflux procedure)

Peroral Endoscopic Myotomy (POEM) - A newer, minimally invasive endoscopic procedure that is now one of the most popular options

Botulinum toxin (Botox) injection - Temporary relief (usually used in elderly or frail patients)

Medications (nitrates, calcium channel blockers) - limited effectiveness and used rarely

Prognosis

With proper treatment, most patients can achieve good long-term symptom control, though many will need occasional repeat procedures. Lifelong follow-up is recommended because patients have a slightly increased risk of esophageal cancer (squamous cell carcinoma) later in life.

Would you like me to explain the differences between the main treatment options (POEM vs Heller myotomy vs pneumatic dilation) or tell you what to expect during the diagnostic workup?

**Model Qwen3-Max**

Answer text:

Achalasia is a rare esophageal motility disorder characterized by the inability of the lower esophageal sphincter (LES)-the muscular ring between the esophagus and the stomach-to relax properly during swallowing. This prevents food and liquid from passing smoothly into the stomach. Additionally, the normal coordinated contractions (peristalsis) of the esophageal body are often absent or impaired.

Key Features of Achalasia:

Failure of LES relaxation: The LES remains tightly closed even when swallowing.

Absent or abnormal peristalsis: The esophagus loses its ability to push food downward effectively.

Esophageal dilation: Over time, the esophagus above the LES may become enlarged due to food and liquid buildup.

Common Symptoms:

Difficulty swallowing (dysphagia) for both solids and liquids

Regurgitation of undigested food

Chest pain or discomfort (often mistaken for heart-related pain)

Weight loss (due to reduced food intake)

Nighttime coughing or aspiration (especially when lying down)

Causes:

The exact cause is not always known, but it's often linked to degeneration of nerve cells in the esophagus-particularly in the myenteric plexus. Possible contributing factors include:

Autoimmune processes

Viral infections (e.g., herpes simplex or measles virus triggering an immune response)

Genetic predisposition (rare familial cases)

Diagnosis:

High-resolution manometry (gold standard): Shows absent peristalsis and incomplete LES relaxation.

Barium swallow: Reveals a "bird's beak" appearance of the distal esophagus and esophageal dilation.

Endoscopy: Used to rule out other conditions like esophageal cancer (which can mimic achalasia, called "pseudoachalasia").

Treatment Options:

While there's no cure, treatments aim to reduce LES pressure and improve esophageal emptying:

Pneumatic dilation: Stretching the LES with a balloon.

Heller myotomy: Surgical cutting of the LES muscle (often combined with anti-reflux surgery).

Peroral endoscopic myotomy (POEM): A minimally invasive endoscopic procedure.

Botulinum toxin injection: Temporarily paralyzes the LES (short-term relief).

Medications: Nitrates or calcium channel blockers (less effective long-term).

Important Consideration:

Untreated achalasia increases the risk of aspiration pneumonia and, over decades, may slightly elevate the risk of esophageal cancer (squamous cell carcinoma), so regular monitoring is advised.

Would you like to know more about one specific treatment option or how achalasia is distinguished from other swallowing disorders?

**Q2. Is achalasia the same as acid reflux?**

**Model ChatGPT 5.5**

Answer text:

No. Achalasia and acid reflux/GERD are different conditions, though they can cause some overlapping symptoms like chest discomfort, regurgitation, or trouble swallowing.

Key difference

Achalasia is a swallowing/motility disorder:

The lower esophageal sphincter - the valve between the esophagus and stomach - doesn't relax properly

The esophagus also has trouble pushing food downward

Food and liquid can get stuck and come back up

Acid reflux/GERD is a reflux disorder:

The lower esophageal sphincter is often too weak or relaxes too much

Stomach acid flows back up into the esophagus

This causes heartburn, sour taste, irritation, and sometimes cough or hoarseness

Simple comparison

Condition Main problem Common symptoms

Achalasia Valve doesn't open well; esophagus doesn't move food normally Difficulty swallowing solids and liquids, regurgitation of undigested food, chest pain, weight loss

Acid reflux/GERD Acid comes back up from stomach Heartburn, sour/bitter taste, burning chest discomfort, symptoms after meals or lying down

Why they can be confused

Achalasia can cause regurgitation, but it is usually food or liquid that never made it into the stomach - not necessarily acid. GERD causes regurgitation of acidic stomach contents.

Also, some people develop reflux after achalasia treatment, such as pneumatic dilation or myotomy, because the tight valve is loosened.

When to get medical evaluation

You should talk with a healthcare professional, especially if you have:

Trouble swallowing, especially both solids and liquids

Food sticking in your chest

Unexplained weight loss

Recurrent vomiting or regurgitation

Chest pain

Symptoms not improving with acid-reducing medicine

Doctors may use tests such as upper endoscopy, barium swallow, and esophageal manometry to tell the difference.

Bottom line: achalasia is not the same as acid reflux. Achalasia is mainly a problem with esophageal movement and a valve that won't relax; reflux is stomach acid flowing backward. If you're having swallowing trouble, it's worth getting checked rather than assuming it's GERD.

**Model Claude Opus 4.7**

Answer text:

No, achalasia and acid reflux (GERD) are different conditions, though they can have some overlapping symptoms.

Achalasia

A rare motility disorder of the esophagus

The lower esophageal sphincter (LES) fails to relax, and the esophagus loses its ability to push food down

Food and liquid get stuck in the esophagus

Main symptoms: difficulty swallowing (dysphagia) for both solids and liquids, regurgitation of undigested food, chest pain, weight loss

Caused by damage to nerves in the esophagus

Acid Reflux (GERD)

A common condition where the LES is too weak or relaxes inappropriately

Stomach acid flows backward into the esophagus

Main symptoms: heartburn, sour taste, regurgitation of acidic fluid, sometimes cough or sore throat

Often related to diet, weight, hiatal hernia, or lifestyle factors

Key Differences

Feature Achalasia Acid Reflux

LES problem Won't relax Too loose

Swallowing Difficult Usually normal

Regurgitated material Undigested food Acidic stomach contents

Heartburn Less common Hallmark symptom

Treatment Procedures to open LES (e.g., Heller myotomy, POEM, balloon dilation) Acid-suppressing medications, lifestyle changes

Why the Confusion?

Both can cause regurgitation and chest discomfort, and achalasia is sometimes misdiagnosed as GERD initially. However, treating achalasia like reflux (with acid blockers) won't help.

If you're having persistent swallowing difficulty or regurgitation, see a doctor - diagnosis usually involves an endoscopy, barium swallow, and esophageal manometry.

**Model DeepSeek V4 Pro**

Answer text:

No, achalasia and acid reflux (gastroesophageal reflux disease, GERD) are not the same. They are distinct esophageal disorders with different causes, typical symptoms, and treatments-though they can sometimes be confused because both can cause heartburn.

Here's how they differ:

Feature Achalasia (motility disorder) Acid reflux (GERD)

What it is The lower esophageal sphincter (LES) fails to relax properly, and the esophagus loses its normal peristalsis (squeezing motion). Food and saliva get stuck. The LES is weak or relaxes too often, allowing stomach acid to flow backwards into the esophagus, causing irritation.

Main symptoms Dysphagia (trouble swallowing) to both solids and liquids, regurgitation of bland, undigested food, chest pain, weight loss. Heartburn can occur but is less acid-related. Heartburn (burning behind the chest), acid or bitter-tasting liquid coming up, regurgitation of acidic stomach contents, symptoms often worse when lying down or after meals.

What comes up Retained food and saliva (often not acidic, though fermentation can create some acid). Stomach acid and partially digested food.

Diagnosis High-resolution esophageal manometry is the gold standard - shows impaired LES relaxation and absent peristalsis. Barium swallow shows "bird's beak" narrowing. Typical symptoms; endoscopy may show esophagitis; pH monitoring can confirm acid exposure. Manometry usually shows a weak but relaxing LES and normal peristalsis.

Treatment Pneumatic dilation, Heller myotomy, or POEM (peroral endoscopic myotomy) to open the LES. Medication is rarely effective. Lifestyle changes, proton pump inhibitors (PPIs), H2 blockers, and sometimes anti-reflux surgery like fundoplication.

Complications Esophageal dilation, food impaction, aspiration pneumonia, weight loss. Very long-standing achalasia may slightly increase the risk of esophageal cancer. Esophagitis, strictures, Barrett's esophagus (a precancerous change), and possibly esophageal adenocarcinoma.

Why they sometimes get mixed up

Some people with achalasia report "heartburn," which can lead doctors to initially suspect GERD. In achalasia, the discomfort often comes from:

Food and saliva that sit in the esophagus and ferment, producing acid.

Esophageal spasms or pressure from the food-filled esophagus.

The muscle strain of a tight LES.

A patient who doesn't improve with standard acid-suppressing medications, and who has trouble swallowing liquids and solids, should be evaluated for a motility disorder like achalasia. Manometry is key - it tells them apart definitively.

Potential risk and optimisation

Risk of misdiagnosis: Many achalasia patients are initially treated for GERD for months or years, leading to delay in proper therapy and progressive esophageal damage. If you or someone you know has persistent dysphagia (especially to liquids) alongside heartburn, it's worth asking for a manometry study.

Optimisation: If you're already diagnosed with either condition, don't self-diagnose. A gastroenterologist can help rule out overlapping issues (e.g., achalasia can coexist with reflux in a condition called "reflux achalasia"). Always get a manometry if dysphagia is prominent.

Follow-up question to keep the conversation going: Are you asking because of symptoms you or someone else is experiencing? If so, could you share whether there's trouble swallowing both solids and liquids, and whether standard heartburn treatments have helped? That might give a clearer picture of what's going on.

**Model Gemini 3.1 Pro Thinking**

Answer text:

No, achalasia and acid reflux are not the same. In fact, regarding how the muscle at the bottom of your esophagus works, they are essentially opposites, even though they can share similar symptoms.

Here is a breakdown of the differences between the two conditions:

1. Achalasia (The valve is too tight)

Achalasia is a rare nerve disorder of the esophagus. In a healthy person, the lower esophageal sphincter (LES)-a muscular ring at the junction of the esophagus and stomach-relaxes to let food into the stomach.

The Mechanism: In achalasia, the nerves in the esophagus are damaged. The esophagus loses its ability to squeeze food down, and the LES fails to relax (open).

The Result: Food and liquids get backed up and stuck in the esophagus.

Key Symptoms: Difficulty swallowing both liquids and solids, regurgitation of undigested food, chest pain, and weight loss.

2. Acid Reflux / GERD (The valve is too loose)

Acid reflux, or Gastroesophageal Reflux Disease (GERD), is a very common condition.

The Mechanism: In this case, the LES is too relaxed or opens at the wrong times.

The Result: Because the valve doesn't close properly, stomach acid and digested food flow backward (reflux) up into the esophagus.

Key Symptoms: Heartburn (a burning sensation in the chest), a sour taste in the mouth, chronic cough, and sometimes regurgitation of acidic fluid.

Why are they easily confused?

Achalasia is frequently misdiagnosed as acid reflux in its early stages. This happens because:

Overlapping Symptoms: Both can cause chest pain, regurgitation, and a feeling of a lump in the throat.

"Heartburn" in Achalasia: People with achalasia often report "heartburn," but it is usually caused by food fermenting in the esophagus or esophageal muscle spasms, rather than stomach acid.

Potential Risks and Medical Recommendations

Risk of Misdiagnosis: If a patient has achalasia but is treated for acid reflux (e.g., given proton pump inhibitors like Omeprazole), their symptoms will not improve, and the esophagus can become stretched and severely damaged over time (megaesophagus).

Proper Testing: To distinguish between the two, a doctor will usually recommend specific tests, such as an Endoscopy (looking inside with a camera), a Barium Swallow (an X-ray test after drinking a chalky liquid), or Esophageal Manometry (a test that measures the muscle contractions and valve pressure in the esophagus).

Disclaimer: I am an AI, not a doctor. If you are experiencing symptoms, it is highly recommended that you consult a gastroenterologist for an accurate diagnosis.

Would you like to know more about the specific tests used to diagnose these conditions, or are you asking because you are experiencing certain symptoms?

**Model Grok-4.3**

Answer text:

No, achalasia is not the same as acid reflux (GERD). They are two completely different disorders that can sometimes cause overlapping symptoms like difficulty swallowing or chest discomfort, which is why they are occasionally confused.

Quick Comparison

Feature Achalasia Acid Reflux (GERD)

Main Problem The esophagus doesn't relax properly. The lower esophageal sphincter (LES) fails to open, and the esophagus loses its normal peristalsis (wave-like contractions). The LES is too loose or opens at the wrong times, allowing stomach acid to flow back up into the esophagus.

What Happens Food and saliva get trapped in the esophagus (like a bag that won't empty). Acid from the stomach comes back up and irritates the esophagus.

Typical Symptoms Progressive difficulty swallowing solids and liquids Regurgitation of undigested food/saliva (often at night) Chest pain Weight loss Feeling like food is stuck Heartburn (burning behind breastbone) Acid taste in mouth Regurgitation of sour liquid Worse after meals, lying down, or bending over

Cause Degeneration of nerves in the esophagus (often autoimmune or idiopathic). Weak LES, hiatal hernia, obesity, diet, pregnancy, etc.

Diagnosis Barium swallow (shows "bird-beak" sign), esophageal manometry (gold standard), endoscopy Endoscopy, pH monitoring, symptom response to PPI medication

Treatment Pneumatic dilation Heller myotomy or POEM surgery Botox injections Medications (limited effect) Lifestyle changes PPIs / H2 blockers Fundoplication surgery in severe cases

Key Point

In achalasia, the problem is failure to move food downward.

In GERD, the problem is too much acid moving upward.

It is possible for someone to have both conditions, or for achalasia to be misdiagnosed as severe reflux for years. If swallowing both solids and liquids is becoming progressively harder, or if you're regurgitating undigested food, that is much more suggestive of achalasia than simple acid reflux.

Would you like me to explain the diagnostic tests for achalasia, the newest treatment options (like POEM), or how doctors tell the two apart when symptoms overlap?

**Model Qwen3-Max**

Answer text:

No, achalasia and acid reflux (often associated with gastroesophageal reflux disease, or GERD) are not the same condition, although they can share some similar symptoms-like difficulty swallowing or a sensation of food getting stuck-which sometimes leads to confusion.

Key Differences:

Feature Achalasia Acid Reflux (GERD)

Cause Failure of the lower esophageal sphincter (LES) to relax properly + loss of esophageal peristalsis (due to nerve damage). Weak or inappropriately relaxing LES, allowing stomach acid to flow back into the esophagus.

Main Problem Esophagus can't move food into the stomach effectively. Stomach acid irritates the esophagus due to backward flow.

Typical Symptoms - Difficulty swallowing (solids and liquids)- Regurgitation of undigested food- Chest pain- Weight loss- No heartburn (usually) - Heartburn (burning chest pain)- Regurgitation of sour/acidic liquid- Worse after meals or lying down- May have trouble swallowing only in advanced cases

Diagnostic Test High-resolution esophageal manometry (gold standard), barium swallow, endoscopy Upper endoscopy, pH monitoring, sometimes manometry

Treatment Pneumatic dilation, Heller myotomy (surgery), POEM procedure, Botox injections Lifestyle changes, antacids, H2 blockers, PPIs; surgery (e.g., fundoplication) in severe cases

Important Note:

People with achalasia do not typically have heartburn, which is a hallmark of GERD.

However, some treatments for achalasia (like myotomy) can lead to reflux afterward because they weaken the LES.

If you're experiencing swallowing difficulties or persistent reflux symptoms, it's important to see a gastroenterologist for proper diagnosis-because treating achalasia like GERD (or vice versa) can delay appropriate care.

Would you like to know how doctors differentiate between the two in clinical practice?

**Q3. Is achalasia caused by stress or anxiety?**

**Model ChatGPT 5.5**

Answer text:

Usually, no - achalasia is not considered to be caused by stress or anxiety.

Achalasia is an esophageal motility disorder where the nerves that help move food through the esophagus and relax the lower esophageal sphincter don't work properly. The exact cause is still not fully understood, but medical sources describe it as likely related to loss/degeneration of nerve cells in the esophagus, with possible roles for autoimmune processes, viral infection, genetics, or rarely infections such as Chagas disease - not stress or anxiety. (mayoclinic.org)

That said, stress and anxiety can make symptoms feel worse. They may increase awareness of swallowing, chest tightness, reflux-like discomfort, or esophageal spasm sensations. Also, living with achalasia can itself cause anxiety, food avoidance, and reduced quality of life; studies have looked at higher depression/anxiety burden among people with achalasia. (pmc.ncbi.nlm.nih.gov)

A helpful way to think about it:

Stress/anxiety do not "cause" true achalasia.

They can worsen symptoms or make them more noticeable.

Achalasia symptoms should not be dismissed as anxiety, especially if there is trouble swallowing both solids and liquids, regurgitation of undigested food, chest pain, choking/coughing at night, or weight loss. Achalasia is typically diagnosed with tests such as upper endoscopy, barium swallow, and especially esophageal manometry. (merckmanuals.com)

If you or someone else has been told symptoms are "just stress," but swallowing is progressively difficult, it would be reasonable to ask a gastroenterologist about evaluation for achalasia or other esophageal motility disorders. Do you already have a confirmed achalasia diagnosis, or are you trying to figure out whether your symptoms could be anxiety-related?

**Model Claude Opus 4.7**

Answer text:

Achalasia and Stress/Anxiety

Short answer: No, achalasia is not caused by stress or anxiety, though stress can worsen symptoms.

What actually causes achalasia

Achalasia is a rare esophageal motility disorder caused by the progressive loss of nerve cells (ganglion cells) in the myenteric plexus of the esophagus. This nerve damage leads to:

Failure of the lower esophageal sphincter (LES) to relax

Loss of normal peristalsis in the esophagus

The underlying cause of this nerve degeneration isn't fully understood, but suspected factors include:

Autoimmune response (possibly triggered by viral infection in genetically susceptible people)

Genetic predisposition (rare familial forms exist)

Viral infections (herpes simplex virus has been studied)

Chagas disease (in endemic areas of Central/South America - caused by Trypanosoma cruzi)

The role of stress and anxiety

While stress doesn't cause achalasia, it can:

Aggravate symptoms like chest pain, regurgitation, and difficulty swallowing

Be commonly mistaken for the cause, since symptoms may flare during stressful periods

Develop as a result of living with achalasia (anxiety about eating in public, fear of choking, etc.)

Why this distinction matters

Achalasia requires medical treatment - typically pneumatic dilation, Heller myotomy, or POEM (peroral endoscopic myotomy). Managing stress alone won't reverse the underlying nerve damage, so a proper diagnosis (usually via esophageal manometry, barium swallow, and endoscopy) is essential.

If you or someone you know is experiencing persistent swallowing difficulties, regurgitation, or chest pain, it's worth seeing a gastroenterologist for evaluation.

**Model DeepSeek V4 Pro**

Answer text:

Achalasia is not directly caused by stress or anxiety. It's a primary esophageal motility disorder where the nerve cells in the esophageal wall (myenteric plexus) gradually degenerate, leading to failure of the lower esophageal sphincter to relax and loss of normal peristalsis. The exact cause is unknown, but it's thought to involve autoimmune or viral triggers in genetically susceptible individuals.

That said, stress and anxiety can make the symptoms worse - for example, they might increase the sensation of food sticking or heighten chest discomfort. However, they are not the root cause; even perfectly calm people can have achalasia.

If you're experiencing dysphagia (difficulty swallowing), regurgitation, or chest pain, I'd recommend seeing a gastroenterologist for proper testing (like high-resolution manometry), as early diagnosis can prevent complications like esophageal dilation or aspiration.

Want to know about the typical treatments that actually target the underlying mechanics of achalasia?

**Model Gemini 3.1 Pro Thinking**

Answer text:

No, achalasia is not caused by stress or anxiety.

Achalasia is a rare, progressive neurological disorder of the esophagus. It occurs when the nerve cells in the esophagus (specifically within the myenteric plexus) degenerate and die. This nerve damage prevents the esophagus from squeezing food down and prevents the lower esophageal sphincter (the valve between the esophagus and stomach) from relaxing to let food pass.

While the exact cause of this nerve degeneration remains unknown, medical researchers currently believe it is likely an autoimmune response-possibly triggered by a viral infection (such as the herpes simplex virus) or influenced by genetics-where the body's immune system mistakenly attacks its own nervous system in the esophagus.

The Connection Between Stress, Anxiety, and Achalasia: Even though stress and anxiety do not cause the disease, they are still highly relevant to the condition in two main ways:

Worsening of Symptoms: Stress and anxiety are well-known triggers for the gastrointestinal system. During periods of high stress, patients with achalasia often report that their symptoms-such as chest pain, regurgitation, esophageal spasms, and difficulty swallowing (dysphagia)-become significantly worse.

Result of the Disease: Living with a chronic, disruptive condition that makes eating difficult and unpredictable can, understandably, cause a great deal of anxiety and stress for the patient.

Potential Risk & Optimization Warning:Because anxiety can cause a feeling of a lump in the throat (globus sensation) and stress can cause mild esophageal spasms, early-stage achalasia is frequently misdiagnosed as anxiety, an eating disorder, or acid reflux (GERD). Relying on a psychological diagnosis for severe swallowing difficulties carries the risk of delaying proper treatment, which can lead to severe weight loss, malnutrition, or a dilated esophagus (megaesophagus). If you or someone you know is struggling to swallow, it is highly recommended to seek objective medical testing from a gastroenterologist.

(Disclaimer: I am an AI, not a doctor. Always consult with a qualified healthcare professional for medical diagnoses and advice.)

Would you like to know more about the specific medical tests used to definitively diagnose achalasia, or are you looking for information on how it is typically treated?

**Model Grok-4.3**

Answer text:

No, achalasia is not caused by stress or anxiety.

Achalasia is a rare esophageal motility disorder caused by the degeneration of specific nerve cells (inhibitory neurons) in the myenteric plexus of the esophagus. This leads to two main problems:

Failure of the lower esophageal sphincter (LES) to relax properly

Loss of normal peristalsis (the wave-like muscle contractions that move food down the esophagus)

What the evidence shows

Medical research has not established psychological factors like stress or anxiety as causal. The leading theories involve:

Autoimmune attack on the esophageal nerves (often supported by findings of inflammatory cells and autoantibodies)

Possible viral triggers (such as herpes simplex virus or other infections) in genetically susceptible individuals

Genetic factors (rare familial cases and associations with certain HLA types)

While some patients report that symptoms seemed to begin during a period of high stress, this is considered coincidental or a trigger for noticing existing symptoms rather than the root cause. Stress and anxiety can worsen many gastrointestinal conditions through the brain-gut axis, but they do not destroy the ganglion cells that characterize achalasia on pathology.

Important distinction

Stress and anxiety commonly cause functional esophageal symptoms (such as globus sensation, chest pain, or diffuse esophageal spasm), which are completely different from the structural nerve damage seen in achalasia. These can usually be distinguished through proper testing (high-resolution esophageal manometry is the gold standard).

If you're experiencing symptoms

The main symptoms of achalasia are:

Difficulty swallowing both solids and liquids (progressive)

Regurgitation of undigested food

Chest pain

Unexplained weight loss

Nocturnal coughing or aspiration

Recommendation: These symptoms always warrant medical evaluation. Achalasia is diagnosed with manometry, barium swallow, and endoscopy (to rule out pseudoachalasia caused by cancer). Early diagnosis matters because effective treatments exist (pneumatic dilation, Heller myotomy, POEM procedure, or Botox in some cases).

Disclaimer: This is general medical information based on current scientific understanding (American College of Gastroenterology and European guidelines). I am not a substitute for personalized medical advice.

If you're asking because you're having swallowing difficulties or have been diagnosed with achalasia, would you like information on current treatment options or how the condition is diagnosed?

**Model Qwen3-Max**

Answer text:

Achalasia is not caused by stress or anxiety. It is a neuromuscular disorder of the esophagus characterized by the failure of the lower esophageal sphincter (LES) to relax properly during swallowing, along with a loss of normal esophageal peristalsis (the coordinated muscle contractions that move food down the esophagus).

What Causes Achalasia?

The exact cause of achalasia is not fully understood, but it is believed to result from:

Degeneration of nerve cells in the esophageal wall-particularly in the myenteric plexus.

Possible autoimmune mechanisms, where the body's immune system mistakenly attacks these nerves.

In some cases, it may be associated with viral infections or genetic factors, though most cases are idiopathic (of unknown origin).

Role of Stress and Anxiety

While stress or anxiety do not cause achalasia, they can:

Worsen symptoms like chest pain, difficulty swallowing (dysphagia), or regurgitation.

Mimic or exacerbate functional esophageal disorders (e.g., globus sensation or functional dysphagia), which may be confused with achalasia.

It's important to distinguish achalasia from other conditions like GERD (gastroesophageal reflux disease) or esophageal spasm, which may have overlapping symptoms but different causes and treatments.

Diagnosis & Treatment

Diagnosis typically involves:

High-resolution manometry (gold standard)

Barium swallow study

Endoscopy

Treatment options include:

Pneumatic dilation

Heller myotomy (surgical)

Peroral endoscopic myotomy (POEM)

Botox injections (temporary relief)

If you're experiencing swallowing difficulties or chest discomfort, consult a gastroenterologist for proper evaluation-especially since untreated achalasia can lead to complications like aspiration or significant weight loss.

Would you like information on how to differentiate achalasia from anxiety-related swallowing issues?

**Q4. Can achalasia go away on its own?**

**Model ChatGPT 5.5**

Answer text:

Usually, no - achalasia does not go away on its own. It's typically a chronic esophageal motility disorder caused by loss/dysfunction of nerves that help the lower esophageal sphincter relax and the esophagus push food downward. Once those muscles/nerves are affected, they generally don't return to normal by themselves. Mayo Clinic states there is no cure in the sense of restoring normal esophageal muscle function, but symptoms can often be managed very effectively with treatment. (mayoclinic.org)

Treatment is usually aimed at opening or relaxing the lower esophageal sphincter so food and liquids can pass into the stomach more easily. Options may include pneumatic dilation, Botox injection, Heller myotomy, or POEM depending on age, health, achalasia subtype, and severity. (mayoclinic.org)

It's important not to ignore it: untreated achalasia can worsen over time, and in advanced cases may lead to significant esophageal dilation, aspiration, weight loss, or "end-stage" disease. Mayo notes that untreated achalasia can progress to severe disease that may even require esophagus removal in rare advanced cases. (mayoclinic.org)

See a gastroenterologist if you have trouble swallowing solids and liquids, food coming back up, unexplained weight loss, chest pain, nighttime coughing/choking, or recurrent "reflux" that doesn't respond to acid medications. Diagnosis is commonly confirmed with esophageal manometry, often along with endoscopy and/or barium swallow. (giejournal.org)

If you tell me your main symptoms and whether achalasia has been confirmed by manometry, I can help you understand what the usual next steps are.

**Model Claude Opus 4.7**

Answer text:

No, achalasia does not go away on its own. It's a chronic, progressive disorder of the esophagus where the lower esophageal sphincter fails to relax properly and the esophageal muscles lose their ability to move food downward (peristalsis).

Why it doesn't resolve spontaneously:

The nerve damage causing achalasia (degeneration of nerve cells in the esophageal wall) is permanent

Without treatment, symptoms typically worsen over time

The esophagus can become increasingly dilated and dysfunctional

Treatment is necessary to manage symptoms and prevent complications. Common options include:

Pneumatic dilation - a balloon stretches the sphincter

Heller myotomy - surgical cutting of the sphincter muscle

POEM (peroral endoscopic myotomy) - an endoscopic procedure to cut the muscle

Botox injections - temporary relief, often for those who can't have surgery

Medications (calcium channel blockers, nitrates) - limited effectiveness, usually short-term

Potential complications if untreated:

Severe weight loss and malnutrition

Aspiration pneumonia

Megaesophagus (severely dilated esophagus)

Slightly increased risk of esophageal cancer

While treatments can significantly relieve symptoms and improve quality of life, none completely cure the condition-follow-up care is usually needed long-term. If you or someone you know has symptoms suggesting achalasia (difficulty swallowing, regurgitation, chest pain, weight loss), it's important to see a gastroenterologist for evaluation.

**Model DeepSeek V4 Pro**

Answer text:

No, achalasia does not go away on its own. It's a chronic, progressive motility disorder of the esophagus, meaning the underlying problem-the failure of the lower esophageal sphincter to relax properly and the loss of normal peristalsis-persists and often worsens over time without treatment.

While some people may experience temporary fluctuations in symptom severity (for example, due to dietary changes or stress), the condition itself remains. Without intervention, the esophagus can gradually enlarge (megaesophagus) and lead to complications like aspiration pneumonia, weight loss, and significant discomfort.

The good news is that symptoms can be effectively managed with medical procedures such as pneumatic dilation, surgical myotomy (Heller myotomy), or peroral endoscopic myotomy (POEM). Botulinum toxin injections can offer temporary relief, but they are not a long-term solution. These treatments aim to weaken or cut the tight sphincter muscle, making swallowing easier, though they do not restore normal esophageal movement.

If you're experiencing symptoms like difficulty swallowing, regurgitation, or chest pain, it's important to see a gastroenterologist for proper testing (such as high-resolution manometry) and to discuss the best approach for your situation.

To help me better understand your question: Are you asking for yourself or someone else who is newly diagnosed? If so, I can walk you through the typical progression of the condition and what to expect from the various treatment options.

**Model Gemini 3.1 Pro Thinking**

Answer text:

No, achalasia does not go away on its own.

Achalasia is a chronic and progressive condition. It occurs when the nerves in the esophagus become damaged and degenerate over time. This nerve damage prevents the esophagus from squeezing food down and stops the lower esophageal sphincter (the valve between the esophagus and stomach) from relaxing to let food pass. Because the body cannot repair this specific nerve damage, the condition is permanent and will not resolve without medical intervention.

While there is no cure for achalasia, it is highly treatable, and medical procedures can effectively manage the symptoms and allow people to eat and swallow normally again.

Potential Risks of Untreated Achalasia: It is highly recommended not to wait for the condition to improve on its own. If left untreated, achalasia can lead to serious complications, including:

Severe malnutrition and weight loss: Due to the inability to keep food and liquids down.

Aspiration pneumonia: Food and liquids trapped in the esophagus can back up into the throat and be inhaled into the lungs, causing serious infections.

Megaesophagus: The esophagus can become severely stretched and enlarged over time, losing its shape completely.

Esophageal cancer: Long-term irritation of the esophagus slightly increases the risk of developing esophageal cancer later in life.

Disclaimer: I am an AI, not a doctor. If you suspect you or someone you know has achalasia, it is critical to consult a gastroenterologist for a proper diagnosis and treatment plan.

Common treatments include minimally invasive procedures like pneumatic dilation (stretching the esophageal sphincter with a balloon), Botox injections to relax the muscle, or surgeries like a Heller myotomy or POEM (Peroral Endoscopic Myotomy).

Would you like to know more about how these specific treatment options work, or are you looking for advice on how to manage eating while waiting for a doctor's appointment?

**Model Grok-4.3**

Answer text:

No, achalasia does not go away on its own.

Achalasia is a rare esophageal motility disorder in which the lower esophageal sphincter (LES) fails to relax properly and the normal peristaltic (wave-like) contractions of the esophagus are lost or severely impaired. It is caused by the progressive degeneration of inhibitory neurons in the myenteric plexus of the esophagus. Once these nerve cells are lost, the condition is not reversible without medical or surgical intervention.

Key Points

Spontaneous remission is extremely rare. There are only a handful of documented case reports of possible spontaneous improvement, usually in very early or atypical cases, and most experts consider true spontaneous cure to be exceptional.

In the vast majority of people, symptoms (difficulty swallowing solids and liquids, regurgitation, chest pain, weight loss, nocturnal cough, etc.) progress over time if left untreated.

Untreated achalasia increases the long-term risk of complications, including:

Malnutrition and significant weight loss

Aspiration pneumonia

Esophageal dilation (megaesophagus)

Esophagitis

Increased (though still low) risk of esophageal squamous cell carcinoma

Standard Management

Because it does not resolve spontaneously, treatment is recommended once the diagnosis is confirmed (usually by high-resolution manometry, barium swallow, and endoscopy). Current first-line options include:

Pneumatic dilation

Laparoscopic Heller myotomy (often with a fundoplication)

Peroral endoscopic myotomy (POEM) - currently the most common approach in many centers

Botulinum toxin injection (mainly for patients who are poor candidates for the above or as a bridge)

Medications (nitrates, calcium-channel blockers) are generally ineffective long-term.

Early treatment usually gives the best results and helps prevent irreversible esophageal dilation.

Important: If you or someone you know has symptoms suggestive of achalasia, see a gastroenterologist promptly for proper testing. Early diagnosis leads to better outcomes.

Would you like me to explain the diagnostic tests, compare the different treatment options, or discuss what to expect after treatment?

**Model Qwen3-Max**

Answer text:

Achalasia is a chronic esophageal motility disorder characterized by the failure of the lower esophageal sphincter (LES) to relax properly during swallowing and by absent or abnormal peristalsis in the esophageal body. It does not typically go away on its own.

Key Points:

Progressive condition: Achalasia usually worsens over time if left untreated. Symptoms like dysphagia (difficulty swallowing), regurgitation, chest pain, and weight loss tend to become more severe.

No spontaneous remission: There is no strong evidence that achalasia resolves spontaneously without medical or surgical intervention.

Treatment is necessary: While it can't be cured, effective treatments can significantly improve symptoms and quality of life. These include:

Pneumatic dilation (stretching the LES with a balloon)

Heller myotomy (surgical cutting of the LES muscle)

Peroral endoscopic myotomy (POEM)

Botulinum toxin injection (temporary relief, especially in older or high-risk patients)

Medications (e.g., nitrates or calcium channel blockers-less effective long-term)

Important Consideration:

Untreated achalasia can lead to complications such as megaesophagus (severe esophageal dilation), aspiration pneumonia, or significant malnutrition.

If you or someone you know has symptoms suggestive of achalasia, it's important to consult a gastroenterologist for proper diagnosis (often involving high-resolution manometry, barium swallow, and endoscopy) and management.

Would you like information on how achalasia is diagnosed or which treatment option might be best in specific situations?

**Q5. Is achalasia a type of cancer?**

**Model ChatGPT 5.5**

Answer text:

No. Achalasia is not a type of cancer.

Achalasia is a rare disorder of the esophagus where the lower esophageal sphincter does not relax properly and the esophagus has trouble moving food down into the stomach. This can cause symptoms such as:

Difficulty swallowing solids and liquids

Regurgitation of food or saliva

Chest discomfort

Weight loss

Heartburn-like symptoms

That said, achalasia can sometimes be confused with esophageal cancer, because both can cause trouble swallowing and weight loss. Also, people with long-standing achalasia may have a higher risk of esophageal cancer compared with the general population, but most people with achalasia do not develop cancer.

If someone has new or worsening trouble swallowing, unexplained weight loss, vomiting/regurgitation, or chest pain, they should be evaluated by a doctor-often with tests such as an endoscopy, barium swallow, or esophageal manometry.

A good next step would be to ask: Has achalasia been diagnosed by testing, or are you concerned because of symptoms like trouble swallowing?

**Model Claude Opus 4.7**

Answer text:

No, achalasia is not a type of cancer.

Achalasia is a rare esophageal motility disorder in which the muscles of the esophagus fail to move food properly into the stomach. It has two main features:

Failure of the lower esophageal sphincter (LES) to relax - the valve between the esophagus and stomach stays tight.

Loss of normal peristalsis - the wave-like muscle contractions that push food down are absent or abnormal.

This happens due to damage or loss of nerve cells in the esophagus, though the exact cause is usually unknown (idiopathic).

Common symptoms include:

Difficulty swallowing (dysphagia) - both solids and liquids

Regurgitation of undigested food

Chest pain or discomfort

Weight loss

Heartburn-like symptoms

Important note: While achalasia itself is not cancer, people with long-standing achalasia have a slightly increased risk of developing esophageal cancer (especially squamous cell carcinoma) due to chronic irritation and food stasis. Also, a condition called "pseudoachalasia" can mimic achalasia and is sometimes caused by a tumor near the LES - which is why doctors often perform an endoscopy to rule out cancer when diagnosing achalasia.

Treatments include medications, Botox injections, pneumatic dilation, or surgical procedures like Heller myotomy or POEM (peroral endoscopic myotomy).

If you or someone you know has symptoms suggestive of achalasia, it's important to consult a gastroenterologist for proper evaluation.

**Model DeepSeek V4 Pro**

Answer text:

No, achalasia is not a type of cancer.

Achalasia is a rare, chronic motility disorder of the esophagus - the tube that carries food from the mouth to the stomach. In achalasia, the lower esophageal sphincter (the valve at the bottom of the esophagus) fails to relax properly during swallowing, and the normal muscle contractions (peristalsis) of the esophagus are reduced or absent. This makes it difficult for food and liquids to pass into the stomach.

While achalasia itself is not cancerous, it's important to know that long-standing, untreated achalasia can slightly increase the risk of developing a specific type of esophageal cancer (squamous cell carcinoma) over many years. This is due to chronic inflammation caused by retained food and fluids. However, the vast majority of people with achalasia do not develop cancer - it's simply a risk factor that doctors monitor.

So, in short: achalasia is a functional, non-cancerous disorder, not a malignancy.

Would you like to know more about the common symptoms of achalasia or how it is typically treated?

**Model Gemini 3.1 Pro Thinking**

Answer text:

No, achalasia is not a type of cancer.

It is a rare, benign (non-cancerous) disorder of the esophagus. In a healthy person, the esophagus uses coordinated muscle contractions to push food down into the stomach, and a valve at the bottom (the lower esophageal sphincter) relaxes to let the food pass. In someone with achalasia, the nerves in the esophagus become damaged, meaning the muscles don't contract properly and the valve fails to open, making it very difficult to swallow food and liquids.

Potential Risk Warning and Medical Advice: While achalasia itself is not cancer, it is important to know that having long-standing, untreated achalasia can slightly increase your risk of developing esophageal cancer later in life. This increased risk is generally thought to be caused by chronic irritation and inflammation from food and acid pooling in the lower esophagus over many years.

Because of this risk, it is highly recommended that anyone diagnosed with achalasia stays in regular contact with a gastroenterologist. Effectively managing the condition (through medications, dilation, or surgery) and getting routine endoscopies can help monitor the health of your esophagus and catch any cellular changes early. (Please note: I am an AI, not a doctor, and you should always consult a healthcare professional for medical advice and diagnosis.)

Are you or a loved one currently experiencing symptoms like difficulty swallowing, or are you looking for information on how achalasia is typically treated?

**Model Grok-4.3**

Answer text:

No, achalasia is not a type of cancer.

What is achalasia?

Achalasia is a rare esophageal motility disorder (a problem with the nerves and muscles of the esophagus). In people with achalasia:

The lower esophageal sphincter (the valve at the bottom of the esophagus) fails to relax properly when you swallow.

The normal wave-like contractions (peristalsis) that push food down the esophagus are weakened or absent.

This causes food and liquids to get stuck in the esophagus, leading to symptoms such as:

Difficulty swallowing (dysphagia) - for both solids and liquids

Regurgitation of undigested food

Chest pain or discomfort

Unintended weight loss

Heartburn or a feeling of something stuck in the throat

It is a chronic neuromuscular condition, not a malignancy (cancer).

Important connection to cancer

While achalasia itself is not cancer, having long-standing achalasia does increase the risk of developing esophageal squamous cell carcinoma later in life (the risk is estimated to be 10-30 times higher than the general population, though the absolute risk remains relatively low). Because of this, doctors often recommend periodic surveillance (endoscopy) for patients who have had achalasia for many years.

How it's diagnosed and treated

Diagnosis usually involves:

Barium swallow (X-ray)

High-resolution esophageal manometry (the definitive test)

Upper endoscopy (EGD) to rule out cancer or other causes

Treatment focuses on relaxing or disrupting the lower esophageal sphincter:

Pneumatic dilation

Heller myotomy (often done with fundoplication)

Per-oral endoscopic myotomy (POEM)

Medications or botox injections (usually temporary)

Would you like me to explain the difference between achalasia and esophageal cancer in more detail, or describe what diagnostic tests are typically used to make sure it's not cancer?

**Model Qwen3-Max**

Answer text:

No, achalasia is not a type of cancer. It is a non-cancerous (benign) motility disorder of the esophagus.

What is achalasia?

Achalasia occurs when the lower esophageal sphincter (LES)-the muscular ring between the esophagus and the stomach-fails to relax properly during swallowing. Additionally, the normal wave-like muscle contractions (peristalsis) in the esophagus are reduced or absent. This makes it difficult for food and liquids to pass into the stomach.

Key features:

Not malignant: Achalasia is a functional disorder, not a tumor or cancer.

Symptoms: Difficulty swallowing (dysphagia), regurgitation of undigested food, chest pain, weight loss, and sometimes aspiration.

Cause: Often idiopathic (unknown cause), but may involve degeneration of nerve cells in the esophagus. In rare cases, it can be secondary to conditions like Chagas disease or tumors that mimic achalasia ("pseudoachalasia").

Important note:

While achalasia itself is not cancer, people with long-standing achalasia have a slightly increased risk of developing esophageal squamous cell carcinoma over time (though this is still rare). For this reason, regular monitoring may be recommended in some cases.

If you or someone you know has been diagnosed with achalasia, it's important to follow up with a gastroenterologist for proper management and surveillance.

Would you like to know more about how achalasia is diagnosed or treated?

**Q6. What symptoms are common in achalasia?**

**Model ChatGPT 5.5**

Answer text:

Common symptoms of achalasia include:

Difficulty swallowing - especially with both solids and liquids

Food or liquid coming back up into the mouth, especially after meals or when lying down

Chest pain or chest pressure

Heartburn-like symptoms

Coughing or choking, especially at night

Unintentional weight loss

Feeling that food is stuck in the chest or lower throat

Bad breath from retained food in the esophagus

Recurrent respiratory infections or aspiration, if food or liquid enters the airway

Achalasia happens when the lower esophageal sphincter does not relax properly and the esophagus has trouble moving food toward the stomach.

If someone has progressive swallowing difficulty, weight loss, chest pain, or repeated choking/coughing episodes, they should be evaluated by a healthcare professional. A related next step would be discussing how achalasia is diagnosed, such as with a barium swallow, endoscopy, or esophageal manometry.

**Model Claude Opus 4.7**

Answer text:

Common Symptoms of Achalasia

Achalasia is a rare esophageal motility disorder where the lower esophageal sphincter fails to relax properly and esophageal peristalsis is impaired. Common symptoms include:

Primary Symptoms

Dysphagia (difficulty swallowing) - typically for both solids and liquids; this is the hallmark symptom

Regurgitation of undigested food or saliva, especially when lying down

Chest pain or discomfort, often retrosternal, which may worsen after eating

Heartburn that doesn't respond to typical acid-reflux medications

Secondary Symptoms

Weight loss (often unintentional) due to difficulty eating

Difficulty belching

Sensation of food sticking in the chest or throat

Coughing or choking, particularly at night

Hiccups

Bad breath (halitosis) from food retention in the esophagus

Complications That May Develop

Aspiration pneumonia from regurgitated food entering the lungs

Nocturnal cough or respiratory symptoms

Malnutrition in advanced cases

Slightly increased long-term risk of esophageal cancer

Important Notes

Symptoms typically progress slowly over months to years, which often delays diagnosis

Patients may adopt compensatory behaviors like eating slowly, drinking lots of water with meals, or raising their arms/standing to help food pass

Symptoms can sometimes be mistaken for GERD, which can delay correct diagnosis

If you or someone you know is experiencing persistent swallowing difficulties, it's important to consult a healthcare provider for proper evaluation, which typically involves esophageal manometry, barium swallow, and endoscopy.

**Model DeepSeek V4 Pro**

Answer text:

Achalasia is a rare esophageal motility disorder where the lower esophageal sphincter (LES) fails to relax properly, and the esophageal muscles lose normal peristalsis. This leads to difficulty moving food into the stomach. The most common symptoms include:

Progressive dysphagia - Difficulty swallowing both solids and liquids (often starting with solids, then advancing to liquids). Patients may feel food sticking in the chest or throat.

Regurgitation - Undigested food or saliva coming back up, especially when lying down or bending over. This can happen hours after meals and may lead to coughing or aspiration (entry into the lungs).

Chest pain - Often described as a squeezing or pressure sensation behind the breastbone, sometimes mistaken for heart pain. It may occur during meals or spontaneously.

Heartburn - Not due to acid reflux, but from food debris fermenting in the esophagus, causing a burning sensation. (Note: Achalasia heartburn does not respond to typical acid-suppressing medications.)

Weight loss - Unintentional, because of reduced caloric intake or avoidance of eating.

Coughing or choking - Particularly at night, from aspiration of retained material.

Sensation of a lump in the throat (globus sensation).

Halitosis (bad breath) due to retained food.

Symptoms tend to worsen over time and can significantly impact quality of life. It's important to differentiate these from more common conditions like GERD or esophageal strictures.

Disclaimer: This is not medical advice. If you or someone you know is experiencing these symptoms, consult a healthcare professional for proper evaluation.

Would you also like to know about how achalasia is diagnosed, or the typical treatment options?

**Model Gemini 3.1 Pro Thinking**

Answer text:

Achalasia is a rare disorder of the esophagus where the nerves become damaged, preventing the esophagus from squeezing food down into the stomach and causing the lower esophageal sphincter (the valve between the esophagus and stomach) to fail to relax.

Because food and liquids have trouble passing into the stomach, people with achalasia typically experience the following common symptoms:

Difficulty swallowing (Dysphagia): This is the hallmark symptom. Patients often feel like food or liquids are stuck in their chest or throat. Unlike some other esophageal conditions, this difficulty usually occurs with both solids and liquids from early on.

Regurgitation: Bringing back up undigested food or saliva is very common. This often happens shortly after eating, or even hours later, and can be particularly troublesome at night when lying down flat.

Chest pain or discomfort: Often felt behind the breastbone (sternum). This pain can be a dull ache or severe, and it is sometimes mistaken for a heart attack.

Heartburn-like symptoms: Despite being a different condition, achalasia can cause a burning sensation in the chest, which frequently leads to it being misdiagnosed as Gastroesophageal Reflux Disease (GERD).

Unintentional weight loss: Because eating becomes difficult and uncomfortable, many people naturally eat less, leading to gradual weight loss.

Nighttime coughing or choking: When undigested food and saliva pool in the esophagus, it can spill over into the airways during sleep, causing coughing fits.

Difficulty belching: A feeling of a lump in the throat or an inability to relieve pressure through burping.

Potential Risks & Health Warning:

Symptom Overlap: The symptoms of achalasia (especially chest pain and difficulty swallowing) overlap significantly with other serious, potentially life-threatening conditions, including heart disease and esophageal cancer.

Complications: If left untreated, achalasia can lead to severe malnutrition, dehydration, and a high risk of aspiration pneumonia (a serious lung infection caused by inhaling regurgitated food).

Recommendation: This information is for educational purposes only. If you or someone you know is experiencing difficulty swallowing, unexplained weight loss, or persistent chest pain, it is critical to seek evaluation from a healthcare professional, such as a gastroenterologist, for a proper diagnosis.

Would you like to know more about the specific medical tests used to accurately diagnose achalasia, or are you more interested in the surgical and non-surgical treatment options available?

**Model Grok-4.3**

Answer text:

Common symptoms of achalasia include:

Dysphagia (difficulty swallowing): This is the hallmark symptom. People typically have trouble swallowing both solids and liquids. Food often feels like it is sticking in the lower chest or "not going down."

Regurgitation: Undigested food, saliva, or liquid comes back up, sometimes hours after eating. It frequently occurs at night and can lead to choking or coughing while lying down.

Chest pain or discomfort: Often described as pressure, squeezing, or burning behind the breastbone. It can be severe enough to be mistaken for heart-related pain (angina).

Weight loss: Gradual and unintentional, resulting from reduced food intake due to swallowing difficulties.

Heartburn or sour taste: Many patients experience reflux-like symptoms even though the lower esophageal sphincter fails to relax rather than being too loose.

Nocturnal cough or aspiration: Food or saliva can enter the lungs during sleep, causing coughing, wheezing, or recurrent lung infections.

Excessive salivation (sialorrhea) and a feeling of "something stuck" in the throat or chest.

Hiccups or frequent burping in some cases.

Symptoms usually develop slowly over months to years and tend to worsen progressively. Early on, people may compensate by eating more slowly, drinking more fluids with meals, or avoiding certain foods.

Important disclaimer: I am an AI language model, not a physician. These are the classically reported symptoms, but many other conditions (GERD, esophageal strictures, eosinophilic esophagitis, and even esophageal cancer) can cause overlapping symptoms. A proper diagnosis requires medical evaluation, typically with barium swallow, esophageal manometry, and endoscopy.

If you or someone you know is experiencing these symptoms, please see a gastroenterologist promptly.

Would you like me to explain how achalasia is diagnosed, what causes it, or what the current treatment options are?

**Model Qwen3-Max**

Answer text:

Achalasia is a rare esophageal motility disorder characterized by the failure of the lower esophageal sphincter (LES) to relax properly during swallowing and the absence of normal peristalsis in the esophageal body. Common symptoms include:

Dysphagia (difficulty swallowing) - This is the most common symptom and typically affects both solids and liquids, often worsening over time.

Regurgitation - Undigested food or saliva may be regurgitated, especially when lying down. This can sometimes lead to aspiration.

Chest pain - May occur due to esophageal distension or spasm; it can mimic cardiac pain.

Weight loss - Often occurs as a result of reduced food intake due to swallowing difficulties.

Heartburn - Though not caused by acid reflux in the typical sense, patients may report a burning sensation.

Nocturnal cough or choking - Caused by regurgitated material entering the airway during sleep.

It's important to note that symptoms usually develop gradually and progressively. Achalasia should be distinguished from other conditions like gastroesophageal reflux disease (GERD) or esophageal cancer, which can present with similar symptoms.

Diagnosis typically involves:

High-resolution esophageal manometry (gold standard)

Barium swallow study (showing a "bird's beak" appearance)

Endoscopy (to rule out mechanical obstruction or malignancy)

If you or someone you know is experiencing these symptoms, consultation with a gastroenterologist is recommended for proper evaluation.

Would you like to know more about diagnostic tests or treatment options for achalasia?

**Q7. I feel food stuck in my chest when swallowing. Could this be achalasia?**

**Model ChatGPT 5.5**

Answer text:

Feeling like food is stuck in your chest when swallowing can happen with achalasia, but it can also be caused by several other conditions-some common and some that need prompt evaluation.

Achalasia is possible, especially if you have:

Trouble swallowing both solids and liquids

Regurgitation of undigested food or saliva

Symptoms that have been gradually worsening

Chest discomfort after eating

Nighttime coughing/choking

Weight loss

Achalasia is an esophageal motility disorder where the lower esophageal sphincter doesn't relax properly and the esophagus doesn't move food down normally. Typical symptoms include dysphagia, regurgitation, chest pain, cough, and weight loss. (mayoclinic.org)

However, the sensation of food sticking can also be due to:

GERD/reflux-related narrowing or inflammation

Eosinophilic esophagitis - often associated with allergies/asthma/eczema

Esophageal stricture or ring

Esophageal spasm

A food impaction

Less commonly, a tumor or external compression

You should contact a doctor/gastroenterologist soon, especially if this is new, recurrent, or worsening. Evaluation often starts with upper endoscopy to look for inflammation, narrowing, rings, tumors, or food impaction; achalasia is typically confirmed with esophageal manometry, and a barium swallow can provide clues such as a dilated esophagus or "bird-beak" narrowing. (sysge.org)

Seek urgent care / ER now if:

Food is currently stuck and you can't swallow saliva

You're drooling, choking, or having trouble breathing

You have severe chest pain, sweating, shortness of breath, or pain radiating to arm/jaw/back

You're vomiting blood or have black stools

You have significant unexplained weight loss or progressive inability to swallow

Until you're assessed, consider small bites, chewing thoroughly, eating slowly, staying upright after meals, and avoiding tough/dry foods-but don't rely on this as a substitute for evaluation.

A helpful question: does this happen with solids only, or with both solids and liquids?

**Model Claude Opus 4.7**

Answer text:

I'm not able to diagnose you, but I can share information that may help you decide what to do next.

Achalasia is one possibility, but not the only one

Achalasia is a relatively rare disorder where the lower esophageal sphincter doesn't relax properly and the esophagus loses its normal muscle coordination. Symptoms often include:

Dysphagia (difficulty swallowing) for both solids and liquids

Sensation of food sticking in the chest

Regurgitation of undigested food

Chest pain or discomfort

Heartburn that doesn't respond to acid reflux medications

Gradual, unintentional weight loss

Symptoms that slowly worsen over months to years

Other causes of "food stuck" sensation

Far more common possibilities include:

GERD (acid reflux) and related esophagitis

Esophageal stricture (narrowing from chronic reflux)

Eosinophilic esophagitis (especially in younger adults with allergies/asthma)

Esophageal spasm

Schatzki ring or other structural issues

Functional dysphagia

Less commonly, tumors

When to seek prompt medical care

See a doctor soon - and don't just self-treat - especially if you have:

Trouble swallowing liquids as well as solids

Unintentional weight loss

Vomiting or regurgitation of food

Pain when swallowing

Anemia or signs of GI bleeding (black stools, vomiting blood)

Symptoms lasting more than a couple of weeks

What evaluation usually looks like

A gastroenterologist will typically recommend:

Upper endoscopy (EGD) - to look directly at the esophagus

Barium swallow - can show the classic "bird's beak" in achalasia

Esophageal manometry - the gold standard for diagnosing achalasia

The good news: most causes of this symptom are treatable, and even achalasia has effective treatments (pneumatic dilation, POEM procedure, Heller myotomy).

Would you like more detail on any of these conditions or what to expect from testing?

**Model DeepSeek V4 Pro**

Answer text:

It's completely understandable to feel concerned when something like this happens. Sensations of food "sticking" in the chest during swallowing (dysphagia) can be alarming, and achalasia is one of the possible underlying causes. However, I want to emphasize up front: I'm an AI, not a doctor, and I can't provide a diagnosis. You should see a gastroenterologist for proper evaluation.

Let me break down what achalasia is, how it might fit your experience, and what else could be causing that feeling.

What is achalasia?

Achalasia is a rare esophageal motility disorder in which the lower esophageal sphincter (the muscular valve where the esophagus meets the stomach) fails to relax properly during swallowing. At the same time, the normal wave-like contractions (peristalsis) that push food down the esophagus are reduced or absent. This can cause:

Food/liquid getting stuck - often felt behind the breastbone.

Regurgitation of undigested food or saliva, especially at night.

Chest discomfort or pain (sometimes mistaken for heart-related pain).

Slow, progressive difficulty swallowing both solids and liquids (a key red flag).

Unintentional weight loss and sometimes coughing or choking when lying down.

These symptoms often develop gradually over months or years, so if yours started suddenly, it might point toward something else.

Could it be achalasia? Possibly, but it's not the only explanation.

The sensation you described can be caused by achalasia, but many other conditions mimic it:

Gastroesophageal reflux disease (GERD) - acid reflux can cause inflammation, scarring, and narrowing of the esophagus (peptic stricture).

Eosinophilic esophagitis (EoE) - an allergic/immune condition that leads to chronic esophageal narrowing, often with impactions.

Esophageal strictures or rings - benign narrowings from chronic acid exposure, radiation, or other causes.

Esophageal cancer - especially if symptoms are rapidly progressive, accompanied by weight loss, and more common in older adults with risk factors (smoking, alcohol).

Other motility disorders - e.g., distal esophageal spasm, jackhammer esophagus, scleroderma-related dysmotility.

Functional dysphagia - a sensation of a lump in the throat without a physical blockage.

Because achalasia is rare (about 1 in 100,000 people), doctors typically rule out more common causes first. But persistent difficulty with both solids and liquids, along with nighttime regurgitation, would raise the suspicion.

What should you do?

The next step is a gastroenterology consultation. Typical workup may include:

Barium swallow test - to visualize the "bird's beak" narrowing of the lower esophagus, a classic sign of achalasia.

Upper endoscopy - to rule out tumors, inflammation, or strictures.

Esophageal manometry - the gold standard for diagnosing achalasia; it directly measures sphincter pressure and peristalsis.

In the meantime, you might find some relief by:

Eating slowly, chewing thoroughly, and taking smaller bites.

Drinking warm liquids with meals to help food pass.

Sleeping with your head elevated if you experience nighttime regurgitation.

A word on risk: untreated achalasia can lead to complications like esophageal dilation, aspiration pneumonia, and a slightly increased risk of esophageal cancer over many years. So getting it checked is important, but it's manageable-treatments (pneumatic dilation, myotomy, Botox injections) are quite effective.

Have you noticed any regurgitation of undigested food, unexplained weight loss, or coughing when lying flat? Your answer could help clarify which direction to explore when you speak with your doctor. (And again, please make that appointment-professional evaluation is essential here.)

**Model Gemini 3.1 Pro Thinking**

Answer text:

Disclaimer: I am an AI, not a doctor. The information provided below is for educational purposes and should not be used as a substitute for professional medical advice, diagnosis, or treatment. Always consult a healthcare professional regarding any medical concerns.

Yes, the sensation of food getting stuck in your chest when swallowing (medically known as dysphagia) is a primary hallmark symptom of achalasia.

Achalasia is a rare disorder in which the nerves in the esophagus become damaged. As a result, the muscles of the esophagus fail to squeeze food down properly, and the muscular ring at the bottom (the lower esophageal sphincter) fails to relax and open, causing food and liquids to back up into the chest.

However, while it could be achalasia, several other conditions can cause this exact same symptom.

Other Potential Causes

Gastroesophageal Reflux Disease (GERD): Chronic acid reflux can cause stomach acid to damage the esophagus, leading to scar tissue (peptic strictures) that narrow the swallowing tube.

Eosinophilic Esophagitis (EoE): An allergic/immune condition that causes inflammation in the esophagus, making it stiff and prone to food impaction.

Esophageal Spasms: Uncoordinated muscle contractions in the esophagus that can trap food and cause chest pain.

Esophageal Rings or Webs: Thin structures of tissue (like a Schatzki ring) that partially block the esophagus.

Hiatal Hernia: When part of the stomach pushes up into the chest cavity.

Potential Risk Warnings (Red Flag Symptoms)

Difficulty swallowing is a symptom that should never be ignored. You should seek urgent medical evaluation if you experience any of the following:

Inability to swallow saliva or liquids.

Chest pain, especially if it radiates to your jaw, back, or arm (this can be a cardiac emergency).

Unintentional weight loss.

Regurgitation of undigested food or vomiting blood.

Choking or coughing fits while eating.

Optimization & Next Steps

Schedule an appointment: You should see a primary care physician or a Gastroenterologist. They can perform tests like an Endoscopy, a Barium Swallow, or Esophageal Manometry (the gold standard for diagnosing achalasia) to find the root cause.

Symptom Diary: Start tracking what foods get stuck, the time of day, and whether it happens with solids, liquids, or both. This will be highly valuable data for your doctor.

Eating Habits: While waiting for your appointment, eat slowly, take small bites, chew your food thoroughly until it is almost liquid, and drink plenty of water with your meals.

To help me provide more tailored educational information, have you noticed if this issue happens more with solid foods, liquids, or both, and do you experience any accompanying symptoms like heartburn or a cough?

**Model Grok-4.3**

Answer text:

Yes, the sensation of food getting stuck in your chest when swallowing (esophageal dysphagia) can be a symptom of achalasia, but it is not the only possibility and requires professional evaluation.

What achalasia is

Achalasia is a rare esophageal motility disorder in which the lower esophageal sphincter (the valve between the esophagus and stomach) fails to relax properly, and the normal wave-like contractions (peristalsis) in the esophagus are weakened or absent. Food and liquids have trouble passing into the stomach, often causing:

Food or liquid feeling "stuck" in the chest or lower throat

Regurgitation of undigested food

Chest pain or discomfort

Difficulty swallowing both solids and liquids (a key clue)

Gradual weight loss in more advanced cases

These symptoms can develop slowly over months or years.

Other common causes

Many other conditions can produce the exact same "food stuck in chest" feeling, including:

Gastroesophageal reflux disease (GERD) with stricture or inflammation

Eosinophilic esophagitis

Schatzki ring or esophageal web

Esophageal spasm or other motility disorders

Peptic stricture

Rarely, more serious issues such as esophageal cancer or external compression

Because the symptom overlaps with multiple diagnoses-some benign, some serious-self-diagnosis is unreliable.

Important disclaimer: I am not a doctor. This is general medical information, not a diagnosis or medical advice. The only way to know whether you have achalasia (or something else) is through proper medical assessment, which may include a barium swallow study, endoscopy, and/or esophageal manometry (the definitive test for achalasia).

Recommended next step

Please see a physician-ideally a gastroenterologist-soon, especially if the problem is worsening, you're losing weight, having trouble with liquids as well as solids, regurgitating food, or experiencing chest pain. Early evaluation is important.

Have you noticed whether the problem occurs with both solids and liquids, how long this has been happening, or any other symptoms such as regurgitation, heartburn, or unintentional weight loss?

**Model Qwen3-Max**

Answer text:

Feeling like food is stuck in your chest when swallowing-known medically as dysphagia-can indeed be a symptom of achalasia, but it can also be caused by several other conditions. Let's break this down:

What is Achalasia?

Achalasia is a rare esophageal motility disorder where:

The lower esophageal sphincter (LES) fails to relax properly during swallowing.

The esophageal muscles lose their ability to push food down effectively (impaired peristalsis).

Common Symptoms of Achalasia:

Dysphagia (difficulty swallowing) for both solids and liquids (this is a key clue-many other causes affect only solids).

Sensation of food "sticking" or getting stuck in the chest.

Regurgitation of undigested food, especially when lying down.

Chest pain or discomfort (sometimes mistaken for heart pain).

Weight loss over time due to reduced food intake.

Nocturnal cough or aspiration (from regurgitated food entering the airway).

Other Possible Causes of Your Symptom:

GERD (Gastroesophageal Reflux Disease) - Can cause inflammation or strictures that narrow the esophagus.

Esophageal stricture - Often from chronic acid reflux or prior injury.

Esophageal ring or web (e.g., Schatzki ring).

Eosinophilic esophagitis (EoE) - An allergic/immune condition causing inflammation and narrowing.

Esophageal cancer - Less common, but important to rule out, especially with weight loss or progressive symptoms.

Hiatal hernia - May contribute to reflux and swallowing issues.

When to Seek Medical Evaluation:

You should see a doctor promptly if you have:

Persistent or worsening dysphagia.

Unintentional weight loss.

Regurgitation, especially at night.

Pain with swallowing (odynophagia).

Symptoms lasting more than a few days.

Diagnostic Tests for Achalasia:

If achalasia is suspected, your doctor may order:

High-resolution esophageal manometry (gold standard for diagnosing achalasia).

Barium swallow study (shows characteristic "bird's beak" narrowing).

Upper endoscopy (to rule out structural causes like tumors or strictures).

Important: While achalasia is not an emergency, persistent dysphagia always warrants medical evaluation to rule out serious conditions.

Would you like help preparing questions to ask your doctor, or would you like to describe more of your symptoms (e.g., duration, triggers, associated symptoms) so we can explore further?

**Q8. Can achalasia cause regurgitation of undigested food?**

**Model ChatGPT 5.5**

Answer text:

Yes. Achalasia can cause regurgitation of undigested food, often hours after eating.

In achalasia, the lower esophageal sphincter doesn't relax properly and the esophagus has impaired movement, so food and liquid can pool in the esophagus instead of passing normally into the stomach. Because the food may not have reached the stomach, it can come back up undigested, sometimes especially when lying down or bending over.

Other common symptoms can include:

Difficulty swallowing solids and liquids

Chest discomfort or pressure

Heartburn-like symptoms

Coughing, choking, or aspiration at night

Weight loss in some cases

That said, regurgitation of undigested food can also occur with other conditions, such as esophageal strictures, Zenker diverticulum, severe reflux, or motility disorders. If this is happening repeatedly-especially with trouble swallowing, weight loss, nighttime choking, or vomiting blood-it's important to see a clinician or gastroenterologist. Evaluation may include an upper endoscopy, barium swallow, and esophageal manometry, which is the key test for achalasia.

If you're experiencing this, a useful next step is to note when it happens after meals, whether solids/liquids are affected, and whether it occurs when lying down, then bring that information to a healthcare professional.

**Model Claude Opus 4.7**

Answer text:

Yes, achalasia commonly causes regurgitation of undigested food. This is one of the hallmark symptoms of the condition.

Why it happens: Achalasia is a disorder where the lower esophageal sphincter (LES) fails to relax properly, and the esophagus loses its normal coordinated muscle contractions (peristalsis). As a result, food and liquids cannot pass efficiently into the stomach and instead accumulate in the esophagus.

Characteristics of regurgitation in achalasia:

The regurgitated material is undigested food, because it never reached the stomach

It is typically not sour or bitter (unlike acid reflux), since it hasn't mixed with stomach acid

It may contain saliva and mucus

It often occurs hours after eating or when lying down

It can happen at night, sometimes leading to coughing, choking, or aspiration into the lungs

Other common symptoms of achalasia:

Dysphagia (difficulty swallowing solids and liquids)

Chest pain or discomfort

Weight loss

Heartburn-like sensation

Aspiration pneumonia (in severe cases)

Important note: Regurgitation of undigested food can also occur with other conditions such as Zenker's diverticulum, esophageal strictures, or gastroparesis, so proper evaluation by a healthcare provider is essential. Diagnosis of achalasia typically involves esophageal manometry, barium swallow study, and endoscopy.

If you or someone you know is experiencing these symptoms, it's important to consult a gastroenterologist for proper assessment and treatment.

**Model DeepSeek V4 Pro**

Answer text:

Yes, achalasia can definitely cause regurgitation of undigested food-in fact, it's one of the hallmark symptoms.

When you have achalasia, the lower esophageal sphincter fails to relax properly, and the esophagus loses its normal peristaltic waves. Food and liquid get stuck in the esophagus instead of moving down into the stomach. Because this material never reaches the stomach acid, it remains bland-tasting, non-bitter, and often undigested. Regurgitation tends to occur minutes to hours after meals, frequently when lying down, and can be spontaneous or triggered by bending over.

Why this matters from a risk perspective: Chronic regurgitation, especially while sleeping, can lead to aspiration pneumonitis or pneumonia, so patients are advised to sleep with the head elevated and avoid late-night meals. Untreated achalasia also carries a small but real long-term risk of esophageal cancer due to chronic stasis and inflammation.

Optimization / management: If you (or someone you're asking about) are experiencing these symptoms, it's important to get a proper evaluation. Definitive treatments like pneumatic dilation, laparoscopic Heller myotomy, or peroral endoscopic myotomy (POEM) can relieve the obstruction and dramatically reduce regurgitation. Early intervention helps prevent complications and improves quality of life.

Follow-up thought: Would you like to know how doctors differentiate achalasia from other conditions that cause regurgitation (like GERD or esophageal strictures), or are you curious about the typical diagnostic workup?

**Model Gemini 3.1 Pro Thinking**

Answer text:

Yes, absolutely. Achalasia is one of the classic causes of regurgitating undigested food.

Here is a breakdown of why this happens and what it means:

Why Achalasia Causes Regurgitation

Achalasia is a rare disorder of the esophagus (the tube that carries food from your mouth to your stomach). It causes regurgitation of undigested food due to two main failures in the digestive process:

Lack of Peristalsis: The muscles of the esophagus lose their ability to contract in a coordinated way (peristalsis) to push food downward.

Failure of the Sphincter: The lower esophageal sphincter (LES)-a muscular ring where the esophagus meets the stomach-fails to relax and open when you swallow.

Because the food cannot enter the stomach, it pools and collects in the esophagus. Over time, the esophagus becomes dilated (stretched). The trapped food, which never reaches the stomach acid to be digested, eventually comes back up into the mouth. Patients often describe this food as looking exactly as it did when they chewed it, sometimes hours after a meal.

Potential Risks and Complications

If you or someone you know is experiencing this symptom, it is important to be aware of the following health risks associated with untreated achalasia:

Aspiration Pneumonia: This is the most immediate danger. When undigested food comes back up, especially while lying down or sleeping, it can be accidentally inhaled into the lungs, leading to severe respiratory infections and lung damage.

Malnutrition and Weight Loss: Because a significant amount of food never reaches the stomach to be processed and absorbed, individuals with achalasia often experience unintended weight loss and nutritional deficiencies.

Esophageal Cancer Risk: Long-term pooling of food and chronic inflammation in the esophagus can slightly increase the risk of developing esophageal cancer over time.

Disclaimer: I am an AI, not a doctor. Because regurgitation of undigested food can lead to serious complications and can also be a symptom of other conditions (such as a Zenker's diverticulum or strictures), it requires professional medical evaluation.

To help me provide more tailored information, are you currently experiencing other symptoms alongside the regurgitation, such as chest pain, difficulty swallowing liquids, or coughing at night?

**Model Grok-4.3**

Answer text:

Yes, achalasia commonly causes regurgitation of undigested food.

Why this happens

Achalasia is an esophageal motility disorder in which two main problems occur:

The lower esophageal sphincter (LES) fails to relax properly.

Normal peristalsis (the wave-like muscular contractions that push food down the esophagus) is absent or severely impaired.

As a result, food and saliva accumulate in a progressively dilated esophagus rather than entering the stomach. Over time, the retained material can ferment or simply sit there until it is regurgitated-often hours after eating. The regurgitated material is typically undigested or only partially digested food, sometimes mixed with mucus or saliva. This is distinct from acid reflux (GERD), where the material is usually acidic and partially digested.

Typical pattern

Regurgitation often occurs at night or when lying down (leading to nighttime coughing or choking).

Patients may wake up with food or bitter-tasting liquid in their mouth or on their pillow.

It can lead to complications such as aspiration (food entering the lungs), recurrent pneumonia, or malnutrition.

Other common symptoms

Progressive difficulty swallowing both solids and liquids (dysphagia)

Chest pain or pressure

Unintentional weight loss

Heartburn-like sensation (even though it's not true acid reflux)

Feeling of food "sticking" in the chest

When to seek care

Regurgitation of undigested food is considered a "red flag" symptom. If you or someone you know is experiencing this along with swallowing difficulties, it warrants prompt medical evaluation. Diagnosis usually involves:

Barium swallow (classic "bird-beak" appearance)

High-resolution esophageal manometry (the definitive test)

Upper endoscopy to rule out pseudoachalasia or cancer

Important disclaimer: This is general medical information, not personalized medical advice. Only a qualified physician can diagnose or treat achalasia.

Would you like me to explain the current treatment options (such as pneumatic dilation, Heller myotomy, or POEM procedure) or what diagnostic tests typically involve?

**Model Qwen3-Max**

Answer text:

Yes, achalasia can cause regurgitation of undigested food-and this is actually one of its classic symptoms.

Why does this happen?

Achalasia is a motility disorder of the esophagus characterized by:

Failure of the lower esophageal sphincter (LES) to relax during swallowing.

Loss of peristalsis in the esophageal body.

As a result:

Food and liquids cannot pass efficiently into the stomach.

They accumulate in the esophagus, especially after meals.

Over time, the esophagus may dilate and act like a storage pouch.

When the person lies down or bends over, this retained material can passively regurgitate back into the mouth-often undigested and sometimes hours after eating.

Key features of regurgitation in achalasia:

Typically not acidic (unlike GERD), because the food hasn't reached the stomach.

May occur at night, leading to coughing, choking, or aspiration pneumonia.

Often described as "spitting up" old food without nausea or retching.

Clinical significance:

Regurgitation of undigested food, especially when combined with progressive dysphagia (to both solids and liquids), chest pain, and weight loss, strongly suggests achalasia and warrants further evaluation (e.g., high-resolution manometry, barium swallow, or endoscopy).

Important: New-onset regurgitation in older adults should prompt evaluation to rule out other causes, including esophageal cancer, which can mimic achalasia ("pseudoachalasia").

Would you like to know how achalasia is diagnosed or what treatment options are available?

**Q9. Can achalasia cause chest pain?**

**Model ChatGPT 5.5**

Answer text:

Yes. Achalasia can cause chest pain, and it's a fairly common symptom.

Achalasia is a disorder where the esophagus has trouble moving food down into the stomach and the lower esophageal sphincter does not relax properly. This can lead to:

Chest pain or pressure

Trouble swallowing, especially with solids and liquids

Regurgitation of food or fluid

Heartburn-like symptoms

Coughing or choking, especially at night

Weight loss in some cases

The chest pain from achalasia may feel like squeezing, pressure, burning, or spasms behind the breastbone. It can sometimes be mistaken for acid reflux, esophageal spasm, or even heart-related chest pain.

Important: If chest pain is new, severe, occurs with shortness of breath, sweating, nausea, fainting, pain radiating to the arm/jaw/back, or you have heart disease risk factors, it should be treated as possible cardiac chest pain and evaluated urgently.

If you already have achalasia and are having chest pain, it's worth discussing with a gastroenterologist. Treatments such as pneumatic dilation, Heller myotomy, POEM, Botox injection, or medications may help depending on the type and severity of achalasia.

A good next step would be: Have you already been diagnosed with achalasia, or are you trying to figure out whether your chest pain could be from it?

**Model Claude Opus 4.7**

Answer text:

Yes, Achalasia Can Cause Chest Pain

Chest pain is a recognized symptom of achalasia, affecting approximately 40-60 percent of patients with this condition.

Characteristics of Achalasia-Related Chest Pain

Location: Typically retrosternal (behind the breastbone)

Quality: Often described as squeezing, cramping, or pressure-like

Timing: May occur during or after meals, but can also happen spontaneously, including at night

Duration: Can last from minutes to hours

Triggers: Eating, drinking cold liquids, or stress

Why It Happens

The chest pain in achalasia is thought to result from:

Esophageal spasms - uncoordinated, forceful contractions

Food retention and esophageal distension

Increased pressure from the failure of the lower esophageal sphincter (LES) to relax

Inflammation of the esophageal lining from retained food and stasis

Other Common Symptoms

Chest pain usually accompanies other classic achalasia symptoms:

Dysphagia (difficulty swallowing solids and liquids)

Regurgitation of undigested food

Weight loss

Heartburn

Nocturnal coughing or aspiration

Important Note

Because chest pain can also indicate cardiac problems, any new or severe chest pain should be evaluated by a healthcare provider to rule out heart-related causes first. Interestingly, chest pain in achalasia may actually improve over time, even without treatment, while swallowing difficulties tend to worsen.

If you're experiencing these symptoms, consult a gastroenterologist for proper evaluation, which may include manometry, endoscopy, or a barium swallow study.

**Model DeepSeek V4 Pro**

Answer text:

Yes, achalasia can definitely cause chest pain. It's actually one of the hallmark symptoms, alongside difficulty swallowing (dysphagia) and regurgitation.

Why does it happen? Achalasia is a rare disorder where the lower esophageal sphincter (the muscular valve between the esophagus and stomach) fails to relax properly, and the esophageal muscles lose their normal rhythmic contractions (peristalsis). This leads to:

Food and liquid backing up in the esophagus

Esophageal distension and muscle spasms

Increased pressure behind the stuck valve

The resulting pain is typically retrosternal (behind the breastbone), can be squeezing, burning, or pressure-like, and sometimes radiates to the back, neck, or arms. Because of its location and character, it's not uncommon for achalasia-related chest pain to be mistaken for a heart attack - which is why anyone with unexplained chest pain should still seek urgent medical evaluation to rule out cardiac causes.

Important note: While I can provide general information, I'm not a doctor. If you're experiencing chest pain, especially if it's new, severe, or accompanied by shortness of breath, sweating, or nausea, please treat it as a potential emergency and get checked immediately.

A related follow-up: Have you been diagnosed with achalasia and are now having chest pain, or are you trying to understand a symptom that might point to a diagnosis? Knowing that can help me share more specific information about what to expect or what tests (like manometry or a barium swallow) are typically used.

**Model Gemini 3.1 Pro Thinking**

Answer text:

Yes, achalasia can absolutely cause chest pain. In fact, it is one of the most common and prominent symptoms of the condition.

Achalasia is a rare disorder where the nerves in the esophagus become damaged, preventing the lower esophageal sphincter (the valve between the esophagus and the stomach) from relaxing to let food pass. It also paralyzes the normal muscle contractions (peristalsis) of the esophagus.

Here is why achalasia causes chest pain and what it typically feels like:

Esophageal Spasms: As the esophagus struggles to push food down through the closed valve, the esophageal muscles can go into severe, uncoordinated spasms, which can be intensely painful.

Stretching and Dilation: Because food and liquid cannot empty into the stomach, they back up and pool in the esophagus. Over time, this causes the esophagus to stretch and become inflamed, leading to a heavy, aching pain.

Heartburn-like Sensations: The retained food can ferment, causing an acidic environment that mimics severe acid reflux or heartburn, even though it isn't true stomach acid reflux.

Characteristics of the Pain: The pain is usually felt behind the breastbone (sternum) and can radiate to the back, neck, jaw, or arms. It can happen after eating or randomly wake a person up in the middle of the night.

Important Medical Risk Warning

Chest pain caused by achalasia can feel almost exactly like a heart attack. It is virtually impossible to tell the difference between severe esophageal spasms and a cardiac event based on symptoms alone.

Immediate Action: If you or someone else is experiencing sudden, severe chest pain-especially if it radiates to the arm or jaw, or is accompanied by shortness of breath, dizziness, or sweating-seek emergency medical attention immediately to rule out a heart attack. Cardiac issues must always be ruled out before attributing chest pain to the esophagus.

Optimization Advice: If cardiac issues have been ruled out and achalasia is suspected, a consultation with a gastroenterologist is highly recommended. They can perform tests like an esophageal manometry (the gold standard for diagnosing achalasia) or an upper endoscopy.

Would you like to know more about the other warning signs of achalasia, or the medical tests used to formally diagnose it?

**Model Grok-4.3**

Answer text:

Yes, achalasia can cause chest pain.

Chest pain is actually one of the most common symptoms of achalasia, often occurring in up to 40-60 percent of patients. It is frequently described as:

Retrosternal (behind the breastbone)

Pressure-like, squeezing, or burning

Sometimes radiating to the back, neck, or arms

Occurring at rest, during meals, or at night

Why does achalasia cause chest pain?

The pain is usually due to:

Esophageal spasm or dysmotility - The esophagus tries to push food forward against a lower esophageal sphincter (LES) that fails to relax properly. This can generate high-pressure contractions.

Food retention and esophageal distension - Stagnant food and saliva cause stretching of the esophageal wall.

Acid or fermenting material - Retained food can ferment and produce lactic acid or other irritants.

Visceral hypersensitivity - Many patients with achalasia have heightened sensitivity in the esophagus.

Important distinctions

This chest pain can closely mimic cardiac angina or even acute coronary syndrome. For this reason, doctors often recommend that patients (especially those over 40 or with risk factors) rule out heart disease first with an EKG, stress test, or cardiology evaluation before attributing the pain solely to achalasia.

Other typical symptoms that often accompany the chest pain

Dysphagia (difficulty swallowing solids and liquids)

Regurgitation of undigested food or saliva

Heartburn-like symptoms

Unexplained weight loss

Nocturnal cough or choking

Next steps if you're experiencing this

If you have been diagnosed with achalasia and have chest pain, effective treatments (pneumatic dilation, Heller myotomy, POEM, or botox injections) often improve or resolve the chest pain in many patients. Medications such as nitrates, calcium channel blockers, or PDE-5 inhibitors are sometimes used temporarily for pain relief.

Would you like me to explain the current treatment options for achalasia-related chest pain, or tell you how doctors typically differentiate this pain from cardiac causes?

**Model Qwen3-Max**

Answer text:

Yes, achalasia can cause chest pain.

Achalasia is a motility disorder of the esophagus characterized by:

Failure of the lower esophageal sphincter (LES) to relax properly during swallowing

Absent or impaired peristalsis (coordinated muscle contractions) in the esophageal body

This leads to difficulty swallowing (dysphagia), regurgitation of undigested food, and sometimes chest pain. The chest pain associated with achalasia is often described as:

A dull, pressure-like, or squeezing sensation behind the sternum (retrosternal)

May mimic cardiac chest pain (angina), leading to initial misdiagnosis

Can occur after eating or even at rest

Sometimes severe enough to wake patients from sleep

The exact mechanism of chest pain in achalasia isn't fully understood but may involve:

Esophageal spasm or distension due to retained food/fluid

Increased intraluminal pressure in the esophagus

Visceral hypersensitivity

Important: Because chest pain can also be a sign of serious cardiac conditions (like heart attack), it's crucial to rule out cardiac causes first, especially in adults with risk factors for heart disease.

Diagnosis of achalasia typically involves:

High-resolution esophageal manometry (gold standard)

Barium swallow (showing "bird's beak" tapering)

Endoscopy (to exclude mechanical obstruction like tumors)

If you or someone is experiencing new or unexplained chest pain, seek prompt medical evaluation to determine the cause.

Would you like information on treatment options for achalasia or how it differs from other esophageal disorders like GERD?

**Q10. Can achalasia cause weight loss?**

**Model ChatGPT 5.5**

Answer text:

Yes. Achalasia can cause weight loss, especially when swallowing becomes difficult enough that a person eats less or avoids food.

Achalasia is a disorder where the esophagus has trouble moving food down into the stomach, and the lower esophageal sphincter does not relax properly. This can lead to:

Difficulty swallowing solids and/or liquids

Regurgitation of undigested food

Chest discomfort or pain

Coughing or choking, especially at night

Reduced food intake

Fear of eating because meals are uncomfortable or embarrassing

Over time, these can result in unintentional weight loss.

That said, unexplained weight loss should always be evaluated by a clinician, because other conditions-including esophageal cancer, strictures, severe reflux complications, infections, or other gastrointestinal diseases-can sometimes cause similar symptoms. This is especially important if weight loss is rapid, progressive, or associated with trouble swallowing.

Seek medical care promptly if you have:

Difficulty swallowing that is worsening

Unintentional weight loss

Vomiting or regurgitation

Blood in vomit or stool

New swallowing problems after age 50

Chest pain, especially if severe or cardiac symptoms are possible

A doctor may evaluate achalasia with tests such as upper endoscopy, barium swallow, and esophageal manometry.

Have you been diagnosed with achalasia already, or are you having swallowing symptoms and weight loss now?

**Model Claude Opus 4.7**

Answer text:

Yes, achalasia commonly causes weight loss. It's one of the hallmark symptoms of the condition.

Why achalasia causes weight loss:

Achalasia is a disorder where the lower esophageal sphincter (LES) fails to relax properly, and the esophagus loses its normal muscle coordination. This makes it difficult for food and liquids to pass into the stomach. As a result:

Difficulty swallowing (dysphagia) - both solids and liquids

Regurgitation of undigested food

Chest pain or discomfort while eating

Fear of eating due to discomfort, leading to reduced intake

Inadequate nutrition because food doesn't reach the stomach efficiently

Characteristics of the weight loss:

Usually gradual and progressive

Can range from mild to significant (10+ pounds or more)

Often accompanied by signs of malnutrition in advanced cases

When to be concerned:

Rapid or severe unintentional weight loss - especially in older adults - warrants prompt evaluation, because conditions like esophageal cancer (or "pseudoachalasia" from a tumor near the LES) can mimic achalasia.

Treatment helps:

Once achalasia is treated (with options like pneumatic dilation, Heller myotomy, POEM procedure, or Botox injections), most people regain the ability to eat normally and recover lost weight.

If you or someone you know is experiencing unexplained weight loss with swallowing difficulties, it's important to see a gastroenterologist for evaluation, which typically includes an upper endoscopy, esophageal manometry, and/or barium swallow study.

**Model DeepSeek V4 Pro**

Answer text:

Yes, achalasia can definitely cause weight loss. In fact, unintentional weight loss is one of the hallmark symptoms, often occurring alongside difficulty swallowing (dysphagia) and regurgitation of undigested food.

How achalasia leads to weight loss:

The lower esophageal sphincter fails to relax, and peristalsis (the muscular contractions that move food downward) is absent. This makes it physically difficult for food and liquids to pass into the stomach.

Patients may unconsciously reduce their food intake to avoid the distressing sensation of food sticking in the chest, pain, or coughing/regurgitation that often follows meals.

Over time, even small deficits add up, leading to gradual but sometimes significant weight loss and malnutrition.

Potential complications to watch for:

Malnutrition and vitamin/mineral deficiencies (e.g., iron, B12, calcium).

Aspiration pneumonia from regurgitated food/liquid entering the airway, especially at night.

Electrolyte imbalances if fluid intake is also compromised.

What can be done: Weight loss from achalasia is reversible once effective treatment restores esophageal emptying. Options include pneumatic balloon dilation, laparoscopic Heller myotomy, and peroral endoscopic myotomy (POEM). Nutritional support (pureed/soft foods, eating slowly, drinking liquids with meals) and sometimes temporary dietary adjustments are part of the pre-treatment plan.

If you or someone you know is experiencing these symptoms, it's important to consult a gastroenterologist for diagnosis (usually via manometry and barium swallow). Would you like to know more about how achalasia is diagnosed or the pros and cons of the different treatment approaches?

**Model Gemini 3.1 Pro Thinking**

Answer text:

Yes, achalasia can definitely cause significant weight loss. In fact, it is one of the hallmark symptoms of the condition.

Achalasia is a rare disorder that affects the esophagus (the tube that carries food from your mouth to your stomach). In people with achalasia, the nerves in the esophagus become damaged. This causes two main problems: the esophagus loses its ability to squeeze food down, and the muscular ring at the bottom of the esophagus (the lower esophageal sphincter) fails to relax and let food into the stomach.

Here is why this leads to weight loss:

Difficulty Swallowing (Dysphagia): Because food and liquids cannot easily pass into the stomach, eating becomes a slow, uncomfortable, and sometimes painful process.

Reduced Food Intake: To avoid the discomfort, coughing, or choking associated with meals, many people with achalasia unintentionally (or intentionally) start eating much less. They may also switch to liquid diets, which often lack sufficient calories.

Regurgitation: Food often collects in the esophagus and is brought back up (regurgitated) before it ever reaches the stomach to be digested and absorbed.

Potential Risk & Optimization Warning:Unexplained weight loss and difficulty swallowing are serious symptoms. While they point toward conditions like achalasia, they can also be warning signs for other severe gastrointestinal issues, including esophageal cancer. If you or someone you know is experiencing these symptoms, it is highly recommended to consult a healthcare provider or a gastroenterologist as soon as possible for a proper diagnosis (often involving a barium swallow, endoscopy, or esophageal manometry).

Disclaimer: I am an AI, not a doctor. This information is for educational purposes and should not replace professional medical advice.

Are you or a loved one currently experiencing difficulty swallowing alongside weight loss, or are you just looking into this condition for general research?

**Model Grok-4.3**

Answer text:

Yes, achalasia commonly causes weight loss.

Why it happens

Achalasia is a rare esophageal motility disorder in which the lower esophageal sphincter (LES) fails to relax properly and the normal peristaltic (wave-like) contractions of the esophagus are impaired or absent. As a result, food and liquids have difficulty passing into the stomach and often sit in the esophagus. This leads to:

Progressive dysphagia (difficulty swallowing), first with solids and later with liquids as well.

Regurgitation of undigested food.

Chest pain or a feeling of food "sticking."

Reduced oral intake because eating becomes slow, uncomfortable, or frightening.

Because patients eat significantly less (and sometimes avoid eating altogether), unintentional weight loss is very common. In many cases, weight loss is one of the presenting symptoms that prompts medical evaluation.

How common and how severe?

Most patients with achalasia experience some degree of weight loss.

In advanced or long-standing cases, the weight loss can be dramatic (10-20+ pounds or more) and may lead to malnutrition.

Rapid or significant weight loss is also one of the "alarm" features that doctors use to differentiate achalasia from other benign swallowing disorders and to prompt earlier investigation for possible pseudoachalasia (a rare cancer-related mimic).

Other associated symptoms

Heartburn or chest pain (can be mistaken for reflux)

Nocturnal regurgitation or coughing/choking at night

Aspiration pneumonia in severe cases

Malnutrition, dehydration, and vitamin deficiencies if untreated

Bottom line

Unexplained difficulty swallowing + weight loss is a classic presentation of achalasia. If you or someone you know is experiencing these symptoms, it should be evaluated promptly by a gastroenterologist. Diagnosis usually involves barium swallow, esophageal manometry, and upper endoscopy.

Would you like me to explain the diagnostic tests, current treatment options (including POEM, Heller myotomy, Botox, or dilation), or what dietary strategies can help while awaiting treatment?

**Model Qwen3-Max**

Answer text:

Yes, achalasia can cause weight loss-and it's actually a common symptom of the condition.

Why does achalasia lead to weight loss?

Achalasia is a disorder of the esophagus where:

The lower esophageal sphincter (LES) fails to relax properly during swallowing.

The esophageal muscles lose their ability to push food down (impaired peristalsis).

As a result:

Difficulty swallowing (dysphagia) occurs for both solids and liquids.

People may eat less due to discomfort, fear of choking, or regurgitation.

Food can get stuck in the esophagus, leading to reduced caloric intake.

Over time, this can result in unintentional weight loss, sometimes significant.

In fact, weight loss of 5-10 percent of body weight is frequently reported in patients with long-standing or severe achalasia.

Other associated symptoms include:

Regurgitation of undigested food

Chest pain or pressure

Heartburn (though not typical acid reflux)

Coughing, especially at night

Aspiration pneumonia (in advanced cases)

When to seek help:

Unintentional weight loss combined with dysphagia warrants prompt medical evaluation to confirm the diagnosis (often via esophageal manometry, barium swallow, or endoscopy) and rule out other conditions like esophageal cancer.

Treatment options (e.g., pneumatic dilation, Heller myotomy, or POEM procedure) can significantly improve swallowing and help restore weight.

Would you like an overview of how achalasia is diagnosed or treated?

**Q11. Can achalasia cause coughing or choking at night?**

**Model ChatGPT 5.5**

Answer text:

Yes. Achalasia can cause coughing, choking, gagging, or "waking up suffocating" at night, especially when lying flat.

Achalasia makes it hard for food and liquid to pass from the esophagus into the stomach. Material can pool in the esophagus and later come back up - particularly during sleep - leading to:

Regurgitation of undigested food or liquid

Coughing or choking at night

Aspiration - small amounts entering the airway/lungs

Wheezing, hoarseness, sore throat, or recurrent chest infections

Symptoms that can be mistaken for acid reflux, though the regurgitated material may not be acidic

This can be concerning because nighttime aspiration may increase the risk of aspiration pneumonia.

What may help temporarily: avoid eating for several hours before bed, sleep with the head of the bed elevated, and avoid lying down soon after meals. But these measures do not treat the underlying problem.

You should discuss this with a gastroenterologist, especially if you have difficulty swallowing, food sticking, weight loss, repeated nighttime choking, or recurrent respiratory symptoms. Diagnosis often involves tests such as esophageal manometry, barium swallow, and endoscopy.

If you are actively choking, having trouble breathing, chest pain, blue lips, confusion, or signs of pneumonia such as fever and shortness of breath, seek urgent medical care.

Have you already been diagnosed with achalasia, or are you having nighttime choking along with trouble swallowing?

**Model Claude Opus 4.7**

Answer text:

Yes, achalasia can definitely cause coughing and choking at night, and this is actually a fairly common symptom.

Why it happens: Achalasia impairs the lower esophageal sphincter's ability to relax, causing food, liquid, and saliva to accumulate in the esophagus instead of passing into the stomach. When you lie down to sleep, this retained material can:

Regurgitate back up into the throat

Be aspirated into the airways (lungs)

Trigger coughing, choking, or a sensation of suffocation

Common nighttime symptoms include:

Waking up coughing or choking

Finding saliva, undigested food, or fluid on your pillow

Sour or bitter taste in the mouth on waking

Shortness of breath at night

Recurrent respiratory infections or pneumonia from aspiration

What can help:

Avoid eating for 3-4 hours before bedtime

Elevate the head of the bed (using bed risers or a wedge pillow, not just extra pillows)

Sleep on your left side, which some find reduces regurgitation

Stay well-hydrated during the day

Seek treatment for the underlying achalasia (options include pneumatic dilation, Heller myotomy, POEM procedure, or botulinum toxin injections)

When to seek prompt medical attention: If you're experiencing frequent nighttime choking, recurrent chest infections, unexplained weight loss, or worsening difficulty swallowing, it's important to see a gastroenterologist. Aspiration pneumonia is a serious complication that can develop from untreated achalasia.

If you haven't been formally diagnosed, these symptoms warrant evaluation, as similar symptoms can occur with GERD, esophageal strictures, or other conditions.

**Model DeepSeek V4 Pro**

Answer text:

Yes, achalasia can absolutely cause nighttime coughing or choking. In fact, these are classic symptoms of the condition — especially when it’s untreated or progressing.

Why it happens

Achalasia prevents the lower esophageal sphincter from relaxing properly, and the esophagus loses its normal squeezing motion (peristalsis). Food, liquid, and saliva build up in the esophagus instead of emptying into the stomach. When you lie flat to sleep, gravity no longer helps keep that retained material down. It can silently flow back up into the throat and spill into the airway (silent aspiration), triggering a cough or a frightening choking sensation. The coughing is often a protective reflex to clear the airway and may be dry or wet-sounding.

Potential risks to be aware of

Aspiration pneumonia: Stomach and esophageal contents entering the lungs can cause infection. This is a serious complication, especially if choking episodes become frequent.

Chronic lung irritation: Even small amounts of aspirated material can lead to persistent cough, wheezing, or asthma-like symptoms.

Sleep disruption: The choking/coughing can jolt you awake repeatedly, leading to poor sleep quality.

Steps you can take (optimization and risk mitigation)

1. Elevate the head of your bed by 6–8 inches (using blocks under the legs, not just extra pillows) , a wedge pillow is an alternative, but the whole upper body should be raised.

2. Avoid eating or drinking (except sips of water) for 3-4 hours before bedtime to minimize residual esophageal volume.

3. Eat slowly and chew thoroughly, and take small sips of water with meals to help food pass.

4. Consider the “chin-tuck” or “head-turn” swallowing technique (though these are more for pharyngeal dysphagia, some people find sipping water and turning the head helps - a speech therapist can advise).

5. Definitive treatment matters most: The above measures are temporary. Achalasia usually requires a procedure to open the tight sphincter -like pneumatic balloon dilation, peroral endoscopic myotomy (POEM), or laparoscopic Heller myotomy. Botox injections are an option for some, but the effect is temporary. Reducing esophageal stasis stops the regurgitation that causes nighttime symptoms.

6. Monitor for signs of lung infection: Fever, chills, productive cough with colored sputum, shortness of breath - seek medical attention promptly.

One important note - if you have been diagnosed with achalasia and are now coughing or choking at night, don’t ignore it. Talk to your gastroenterologist. They may need to reassess esophageal retention (e.g., via timed barium esophagram or endoscopy) and adjust your treatment plan.

Have you noticed these symptoms yourself, or is this about someone you know? Knowing a bit more might help me point you toward the most useful next step - like what a diagnostic journey looks like, or which specialists to see first.

**Model Gemini 3.1 Pro Thinking**

Answer text:

Yes, achalasia can absolutely cause coughing or choking at night. In fact, nighttime regurgitation is one of the classic and most distressing symptoms of the condition.

Why This Happens

In a normal digestive system, the lower esophageal sphincter (a muscular valve at the bottom of the esophagus) opens to let food and liquid into the stomach, and then closes to prevent it from coming back up.

In someone with achalasia, this valve fails to relax, and the esophagus loses its normal squeezing ability (peristalsis). As a result, food, liquids, and saliva pool in the esophagus. When you lie down flat to sleep, you lose the assistance of gravity. The pooled contents can easily flow back up into the throat. If these contents spill over into your windpipe (trachea), your body will instantly trigger a violent coughing or choking reflex to clear the airway.

Potential Risks

Aspiration Pneumonia: The biggest danger of nighttime choking in achalasia is "aspiration"-breathing food particles or stomach acid into the lungs. This can lead to aspiration pneumonia, a serious and potentially life-threatening lung infection.

Sleep Deprivation: Frequent waking due to coughing fits can lead to severe fatigue and a poor quality of life.

Lung Damage: Chronic micro-aspiration over time can lead to long-term lung scarring or asthma-like symptoms.

Optimization and Management Suggestions

While medical or surgical treatment (such as pneumatic dilation, Heller myotomy, or POEM) is usually required to treat the root cause of achalasia, there are steps you can take to manage nighttime symptoms:

Elevate the Head of the Bed: Use a wedge pillow or bed risers to elevate the top of your bed by 6 to 8 inches. Propping yourself up with just standard pillows usually isn't enough and bends your body in a way that can actually worsen pressure on the esophagus.

Timing of Meals: Stop eating and drinking at least 3 to 4 hours before going to bed to give your esophagus as much time as possible to empty.

Dietary Adjustments: Eat softer, easy-to-swallow foods, chew your food very thoroughly, and sip warm liquids during meals to help wash food down.

Disclaimer: I am an AI, not a doctor. If you are experiencing nighttime choking or coughing, it is highly recommended that you consult a gastroenterologist or a medical professional promptly to prevent serious respiratory complications.

Are you currently experiencing these symptoms and looking for ways to manage them, or are you trying to understand a recent achalasia diagnosis?

**Model Grok-4.3**

Answer text:

Yes, achalasia can cause coughing or choking at night.

Why This Happens

Achalasia is a disorder where the esophagus loses its normal ability to propel food downward (aperistalsis) and the lower esophageal sphincter (LES) fails to relax properly. As a result, food, liquids, and saliva accumulate in the esophagus instead of entering the stomach.

At night, when you lie down:

Gravity no longer helps keep contents in the esophagus or stomach.

Retained material (especially undigested food, liquids, or thick saliva) can regurgitate or overflow from the dilated esophagus into the throat.

This overflow can spill into the larynx (voice box) or be inhaled into the trachea, triggering coughing, choking, or a sensation of gasping awake.

Many patients describe waking up suddenly with a choking feeling, coughing fits, or even aspiration (food/liquid entering the lungs).

This is sometimes called nocturnal regurgitation or nocturnal aspiration. In more advanced or long-standing achalasia, the esophagus can become massively dilated (sigmoid esophagus), making nighttime symptoms even more pronounced.

How Common Is It?

Nocturnal coughing, choking, or aspiration symptoms are reported by a significant percentage of people with achalasia-often 30-60 percent in various studies. It is one of the "alarm" symptoms that can lead to complications such as:

Recurrent chest infections or aspiration pneumonia

Chronic cough

Disrupted sleep and fatigue

What You Should Do

See a doctor promptly (gastroenterologist or esophageal motility specialist). These symptoms can indicate significant esophageal retention and increased risk of aspiration.

Diagnostic tests usually include:

High-resolution esophageal manometry (the gold-standard test)

Barium swallow (often shows the classic "bird-beak" appearance and retained column)

Upper endoscopy

Sometimes a pH/impedance study or modified barium swallow to assess aspiration risk

Treatment Can Help

Effective treatment of achalasia (pneumatic dilation, Heller myotomy, POEM, or Botox in selected cases) usually improves or resolves the nighttime coughing and choking by improving esophageal emptying. Lifestyle measures while awaiting treatment include:

Eating the last meal at least 3-4 hours before bedtime

Elevating the head of the bed 6-8 inches

Sleeping on your left side or semi-upright if symptoms are severe

Bottom line: Yes - nocturnal coughing and choking are well-recognized symptoms of achalasia and should be taken seriously as they signal poor esophageal clearance and possible aspiration risk.

Follow-up question for you: How long have you (or the person you're asking about) been experiencing these nighttime symptoms, and are there any other achalasia symptoms such as difficulty swallowing solids/liquids, regurgitation of undigested food, chest pain, or unexplained weight loss? This information can help gauge severity.

**Model Qwen3-Max**

Answer text:

Yes, achalasia can cause coughing or choking at night, and this is a relatively common symptom-especially in more advanced cases.

Why This Happens:

Achalasia is a motility disorder of the esophagus characterized by:

Failure of the lower esophageal sphincter (LES) to relax during swallowing.

Loss of peristalsis in the esophageal body.

As a result, food and liquids accumulate in the esophagus instead of passing into the stomach. When a person lies down (e.g., at night), this retained material can reflux passively into the throat or even be aspirated into the airways, leading to:

Nocturnal coughing

Choking episodes

Waking up gasping or coughing

Recurrent aspiration pneumonia (in severe cases)

Importantly, this is not acid reflux like in GERD-there's usually little acid involved because the LES remains tightly closed. Instead, it's undigested food or saliva causing the irritation.

Clinical Significance:

Nighttime coughing or choking in a patient with known or suspected achalasia may indicate:

Disease progression

Significant esophageal dilation

Increased risk of aspiration

What to Do:

If someone with achalasia experiences these symptoms, they should consult a gastroenterologist. Management strategies may include:

Elevating the head of the bed

Avoiding eating close to bedtime

Esophageal emptying techniques (e.g., drinking warm water before bed)

Definitive treatment (e.g., pneumatic dilation, Heller myotomy, or POEM)

Would you like guidance on distinguishing achalasia-related cough from other causes like GERD or asthma?

**Q12. Can achalasia make swallowing both liquids and solids difficult?**

**Model ChatGPT 5.5**

Answer text:

Yes. Achalasia can make swallowing both liquids and solids difficult, and that pattern is actually a classic clue.

In achalasia, the lower esophageal sphincter doesn't relax properly and the esophagus has impaired coordinated movement, so food and drink can have trouble passing into the stomach. Because it's a motility problem rather than just a physical blockage, people often have trouble with:

Liquids

Solids

Sometimes both from early on

Other possible symptoms include regurgitation of undigested food or liquid, chest discomfort, coughing/choking at night, heartburn-like symptoms, and weight loss.

By contrast, difficulty swallowing solids first that later progresses to liquids can sometimes suggest a narrowing or obstruction, which needs evaluation.

If you're having new, worsening, or persistent swallowing trouble-especially with weight loss, vomiting, blood, anemia, or choking-you should see a healthcare professional. Diagnosis often involves tests such as a barium swallow, upper endoscopy, and esophageal manometry.

A good next step would be to tell me: is the swallowing difficulty with liquids and solids from the beginning, or did it start with solids first?

**Model Claude Opus 4.7**

Answer text:

Yes, achalasia can make swallowing both liquids and solids difficult. This is actually one of the hallmark features that helps distinguish it from other swallowing disorders.

Why both are affected:

Achalasia is a motility disorder of the esophagus where:

The lower esophageal sphincter (LES) fails to relax properly to let food pass into the stomach

The esophagus loses its normal coordinated muscle contractions (peristalsis) needed to push food downward

Because the problem is mechanical/functional rather than a physical narrowing, both liquids and solids get held up.

Clinical significance:

This pattern is important diagnostically. In contrast:

Mechanical obstructions (like strictures or tumors) typically cause difficulty with solids first, with liquid problems developing later as the narrowing worsens

Achalasia and other motility disorders often cause difficulty with both from early on, sometimes with liquids being just as troublesome as solids

Other common symptoms of achalasia:

Regurgitation of undigested food

Chest pain or discomfort

Heartburn (that doesn't respond well to acid-reducing medications)

Weight loss

Coughing or choking, especially when lying down

Feeling of food "sticking" in the chest

If someone is experiencing persistent difficulty swallowing, they should see a healthcare provider. Diagnosis typically involves tests like esophageal manometry, barium swallow, and upper endoscopy. Treatment options include pneumatic dilation, surgical myotomy (Heller myotomy), POEM (peroral endoscopic myotomy), or sometimes Botox injections.

**Model DeepSeek V4 Pro**

Answer text:

Yes, achalasia typically causes difficulty swallowing both solids and liquids, and this is one of its hallmark features.

In many other esophageal disorders, trouble swallowing starts with solids and only later affects liquids. Achalasia is different because the underlying problem-failure of the lower esophageal sphincter (LES) to relax properly, combined with loss of normal esophageal peristalsis-can make liquids just as hard (or even harder) to swallow than solids, especially in the early stages.

Why both are affected

Liquids: Because the LES does not open normally, fluids can pool in the esophagus instead of entering the stomach. Gravity helps somewhat, but the lack of peristalsis means the bolus isn't pushed through efficiently. Many patients report that water or thin liquids cause more discomfort or regurgitation early on, while thicker substances may pass more slowly but with an urge to "chase" with water.

Solids: As the esophagus becomes progressively dilated and floppy, solid foods (especially dry or bulky items like bread, meat, or rice) get stuck at the LES and can lead to a sensation of food "hanging up" in the chest, often relieved only by regurgitation.

Typical symptom pattern

Dysphagia (difficulty swallowing) for both liquids and solids from relatively early in the disease.

Regurgitation of undigested food or saliva, especially when lying down, which can cause nocturnal coughing or aspiration (risk of pneumonia).

Chest discomfort/pain from esophageal spasm or stretching.

Gradual weight loss if intake is limited.

Important note

Because achalasia symptoms can slowly progress over years, some people adapt by eating slowly, drinking a lot of water during meals, or avoiding certain foods, which may mask the severity. However, untreated achalasia can lead to complications like megaesophagus, malnutrition, and chronic aspiration. Diagnosis typically involves barium swallow, high-resolution manometry (the gold standard), and endoscopy to rule out pseudoachalasia (e.g., from a tumor).

Treatment options (pneumatic dilation, Heller myotomy, or POEM) are aimed at disrupting the tight LES to allow better passage, and these can significantly improve swallowing for both solids and liquids.

Follow-up question to keep the conversation going: Would you like a more detailed overview of how achalasia is diagnosed, or are you more interested in the pros and cons of the different treatment approaches?

**Model Gemini 3.1 Pro Thinking**

Answer text:

Yes, achalasia can absolutely make swallowing both liquids and solids difficult. In fact, this is one of the classic, hallmark symptoms of the condition.

Here is a breakdown of why this happens:

The Mechanism: Achalasia is a rare disorder caused by nerve damage in the esophagus (the tube that carries food from the mouth to the stomach). Because of this nerve damage, the esophagus loses its normal muscular contractions (peristalsis) that push food down. Furthermore, the muscular valve at the bottom of the esophagus (the lower esophageal sphincter) fails to relax and open, essentially creating a barricade.

Solids vs. Liquids: With mechanical blockages of the esophagus (such as a benign stricture or a tumor), people typically have trouble swallowing solid foods first, and only have trouble with liquids much later as the blockage grows. In achalasia, however, the impairment is primarily functional (movement-based), so patients usually experience difficulty swallowing both solids and liquids right from the onset of symptoms.

Other common symptoms that often accompany this include regurgitation of undigested food or saliva, chest pain, heartburn, and unintentional weight loss.

Potential Risk Warning & Optimization:Dysphagia (difficulty swallowing) is a serious symptom that can lead to severe complications, including malnutrition, severe dehydration, and aspiration pneumonia (inhaling food, liquid, or saliva into the lungs). Because I am an AI and not a doctor, I strongly advise that anyone experiencing difficulty swallowing seek prompt medical evaluation from a healthcare provider, such as a gastroenterologist.

Would you like to know more about the specific tests doctors use to diagnose achalasia, or the current treatment options available to help manage it?

**Model Grok-4.3**

Answer text:

Yes, achalasia typically makes swallowing both solids and liquids difficult.

Achalasia is an esophageal motility disorder in which the lower esophageal sphincter (LES) fails to relax properly and the normal wave-like contractions (peristalsis) in the esophagus are absent or severely impaired. Because the problem is functional rather than mechanical, patients usually experience dysphagia (difficulty swallowing) for both solids and liquids from the outset.

Key characteristics of achalasia-related swallowing problems:

Both solids and liquids are affected, often early in the disease.

Many people report that liquids are sometimes more difficult than solids, which is the opposite of mechanical obstructions (such as peptic strictures or tumors), where solids usually cause problems first.

Food or liquid often feels like it "sticks" in the chest or comes back up (regurgitation of undigested material).

Symptoms tend to be progressive and can lead to chest pain, weight loss, nighttime coughing, or aspiration.

This pattern of dysphagia to both consistencies is one of the classic clinical clues that points toward a motility disorder like achalasia rather than a structural blockage.

Important: While these are the typical features, symptoms can vary between individuals. Difficulty swallowing is never normal and should be evaluated by a physician (usually a gastroenterologist). Diagnosis typically involves barium swallow, esophageal manometry, and endoscopy to confirm achalasia and rule out pseudoachalasia or other conditions.

Would you like information on how achalasia is diagnosed, current treatment options (such as pneumatic dilation, Heller myotomy, or POEM), or what symptoms you should discuss with a doctor?

**Model Qwen3-Max**

Answer text:

Yes, achalasia can make swallowing both liquids and solids difficult, although the pattern may vary depending on the stage and severity of the condition.

Achalasia is a motility disorder of the esophagus caused by the failure of the lower esophageal sphincter (LES) to relax properly during swallowing, along with a loss of normal peristalsis (coordinated muscle contractions) in the esophageal body. This leads to food and liquids backing up in the esophagus instead of passing into the stomach.

Key points:

Early stages: Patients often report difficulty swallowing solids more than liquids.

As the disease progresses: Both solids and liquids become increasingly difficult to swallow. In fact, difficulty with liquids (especially when lying down or drinking cold liquids) is a hallmark that helps distinguish achalasia from mechanical obstructions like strictures or tumors (which typically affect solids first and spare liquids until later).

Regurgitation of undigested food, chest pain, weight loss, and nighttime coughing are also common symptoms.

If someone experiences progressive dysphagia to both solids and liquids-particularly if liquids are affected early-it should raise suspicion for achalasia and warrant further evaluation (e.g., high-resolution manometry, barium swallow, or endoscopy).

Would you like an overview of how achalasia is diagnosed or treated?

**Q13. How is achalasia diagnosed?**

**Model ChatGPT 5.5**

Answer text:

Achalasia is diagnosed by combining symptoms, tests that rule out blockage/cancer, and a motility test that confirms the disorder.

1) When doctors suspect it

Achalasia is suspected when a person has:

Trouble swallowing both solids and liquids

Regurgitation of undigested food or saliva

Chest discomfort, heartburn-like symptoms, cough/aspiration, or weight loss

"GERD" symptoms that do not improve with acid-suppressing medication

2) Upper endoscopy - to rule out other causes

Most patients get an upper endoscopy (EGD). This lets the doctor look for:

A tumor, stricture, inflammation, or other mechanical obstruction

Food/saliva retained in the esophagus

A tight or "puckered" gastroesophageal junction

This step is important because pseudoachalasia-achalasia-like symptoms caused by cancer or another obstruction-can mimic true achalasia. Guidelines recommend endoscopy to assess the gastroesophageal junction and gastric cardia in patients with suspected achalasia. (mdcalc.com)

3) Barium swallow / esophagram - to show poor emptying

A barium esophagram, often a timed barium swallow, involves swallowing contrast while X-rays track how it moves. Findings that support achalasia include:

Dilated esophagus

Narrowing at the lower end, often called a "bird-beak" appearance

Poor emptying of barium

Reduced or absent normal esophageal contractions

A barium study is very helpful, but by itself it usually does not definitively confirm achalasia. (mdcalc.com)

4) High-resolution esophageal manometry - the gold-standard test

The diagnosis is usually confirmed with high-resolution manometry (HRM). A thin catheter is passed through the nose into the esophagus to measure pressure while you swallow.

Achalasia is characterized by:

Poor relaxation of the lower esophageal sphincter

Absent or abnormal peristalsis - the normal wave-like movement of the esophagus

HRM also classifies achalasia into subtypes, which helps guide treatment:

Type I: minimal esophageal pressurization, failed peristalsis

Type II: failed peristalsis with panesophageal pressurization

Type III: spastic/premature contractions

High-resolution manometry is considered the current gold standard for confirming achalasia and identifying the subtype. (sysge.org)

In short

The usual diagnostic pathway is:

Symptoms to upper endoscopy to rule out obstruction to barium swallow to assess emptying to high-resolution manometry to confirm achalasia and subtype it.

If you're asking because you or someone else has swallowing trouble-especially with both solids and liquids, weight loss, or regurgitation-it's worth seeing a gastroenterologist promptly.

**Model Claude Opus 4.7**

Answer text:

Diagnosing Achalasia

Achalasia is typically diagnosed through a combination of clinical evaluation and specialized tests. Here are the main diagnostic approaches:

Initial Assessment

Medical history: Symptoms like progressive difficulty swallowing (dysphagia) for both solids and liquids, regurgitation, chest pain, and weight loss

Physical examination

Key Diagnostic Tests

1. Esophageal Manometry (Gold Standard)

Measures pressure and muscle contractions in the esophagus

Reveals the hallmark findings:

Failure of the lower esophageal sphincter (LES) to relax during swallowing

Absent or abnormal peristalsis in the esophageal body

High-resolution manometry (HRM) can classify achalasia into three subtypes (Types I, II, III) using the Chicago Classification

2. Barium Swallow (Esophagram)

Shows the characteristic "bird's beak" appearance - a dilated esophagus narrowing at the LES

May reveal:

Esophageal dilation

Poor emptying

Air-fluid levels

"Sigmoid" esophagus in advanced cases

3. Upper Endoscopy (EGD)

Primarily used to rule out other conditions, especially:

Esophageal cancer (pseudoachalasia)

Strictures

Eosinophilic esophagitis

May show a dilated esophagus with retained food/fluid and a tight LES

Additional Tests (When Needed)

Timed barium esophagram: Measures esophageal emptying over 1-5 minutes

EndoFLIP (Functional Lumen Imaging Probe): Assesses LES distensibility

CT scan: If malignancy is suspected

Differential Diagnosis

Important to distinguish achalasia from:

Pseudoachalasia (often from tumors)

GERD

Esophageal strictures

Other motility disorders

If you're experiencing symptoms suggestive of achalasia, it's important to consult a gastroenterologist for proper evaluation.

**Model DeepSeek V4 Pro**

Answer text:

Achalasia is diagnosed through a combination of clinical assessment and specialized tests, with high-resolution esophageal manometry (HREM) as the gold standard. Because its symptoms-progressive dysphagia to solids and liquids, regurgitation, chest pain, and weight loss-can mimic other conditions, a systematic approach is essential to avoid misdiagnosis.

Key Diagnostic Steps

Clinical History & Symptom Assessment

Characteristic dysphagia for both solids and liquids, often worse with emotional stress.

Use of the Eckardt score to quantify symptom severity (weight loss, dysphagia, retrosternal pain, regurgitation).

Risk tip: Early achalasia can be mistaken for GERD, leading to prolonged antacid trials and delayed definitive diagnosis. If symptoms don't respond to high-dose proton pump inhibitors, achalasia must be considered.

High-Resolution Esophageal Manometry (HREM)

Directly measures esophageal peristalsis and lower esophageal sphincter (LES) relaxation.

Chicago Classification v4.0 criteria:

Elevated median integrated relaxation pressure (IRP) (typically greater than 15 mmHg with supine swallows).

Absent peristalsis (100 percent failed swallows) in type I; absent peristalsis + panesophageal pressurization in type II; premature/spastic contractions in type III.

Optimization: HREM with concurrent impedance can detect bolus clearance and exclude functional obstruction when IRP is borderline. Ensure patients stop medications that affect motility (e.g., nitrates, calcium channel blockers, opioids) 24-48 hours before testing.

Barium Swallow (Timed Barium Esophagram)

Classic "bird's beak" appearance at the esophagogastric junction, a dilated esophagus, and poor emptying.

Timed upright films at 1, 2, and 5 minutes quantify retention (e.g., greater than 5 cm barium column at 5 minutes is diagnostic).

Complementary value: Highly suggestive in advanced cases, but may be normal in early disease; not a substitute for manometry.

Upper Endoscopy (EGD)

Rule out pseudoachalasia (malignant stricture at the GEJ, often from adenocarcinoma of the cardia).

Findings: retained food/saliva without mechanical obstruction, tight LES that "pops" open with insufflation.

Critical risk: Any atypical features (older age greater than 60, abrupt onset, significant weight loss, smoking history) warrant endoscopic biopsy, cross-sectional imaging (CT/PET-CT), and/or endoscopic ultrasound (EUS) to exclude malignancy, which can present with manometry identical to primary achalasia.

Adjunctive Tests

CT scan of chest/abdomen: rule out pseudoachalasia, extrinsic compression.

EndoFLIP (functional lumen imaging probe): measures esophagogastric junction distensibility; increasingly used when manometry is equivocal or to guide therapy.

Diagnostic Pitfalls & Risk Optimization

Pseudoachalasia: Accounts for up to 5 percent of manometric achalasia patterns. Always exclude in older patients or those with red-flag symptoms.

Prior fundoplication: Can mimic achalasia manometrically; surgical history is key.

Incomplete manometry: Variability in IRP measurement can be reduced with multiple position testing (upright swallows) and solid/drink challenges if liquid swallows are normal.

Recommended Approach

A practical diagnostic algorithm:

All patients with dysphagia to EGD to exclude structural lesions.

If EGD normal to HREM.

If HREM consistent with achalasia to add timed barium swallow to establish baseline emptying and rule out advanced disease.

If greater than 60 years, abrupt symptoms, or weight loss to cross-sectional imaging and EUS even if manometry suggests primary achalasia.

What specific aspect of achalasia would you like to explore next-treatment options (pneumatic dilation, POEM, Heller myotomy), long-term follow-up risks (like esophageal cancer), or dietary/lifestyle modifications while waiting for intervention?

**Model Gemini 3.1 Pro Thinking**

Answer text:

Disclaimer: I am an AI, not a doctor. The information provided below is for educational purposes and should not replace professional medical advice. If you or someone you know is experiencing swallowing difficulties, please consult a healthcare provider, specifically a gastroenterologist.

Achalasia is a rare disorder that makes it difficult for food and liquid to pass from the swallowing tube (esophagus) into the stomach. This happens because the nerves in the esophagus become damaged, preventing the esophageal muscles from squeezing properly and the lower esophageal sphincter (LES) from relaxing.

Because its symptoms (like difficulty swallowing, heartburn, and regurgitation) mimic other conditions like GERD, achalasia is typically diagnosed using a combination of the following three tests:

1. High-Resolution Esophageal Manometry (The Gold Standard)

This is the most definitive test for diagnosing achalasia.

How it works: A thin, flexible tube containing pressure sensors is passed through the nose, down the esophagus, and into the stomach. You will be asked to swallow small sips of water.

What it shows: It measures the strength and coordination of the muscle contractions in the esophagus and evaluates whether the lower esophageal sphincter properly relaxes during a swallow.

2. Barium Swallow (Esophagram)

This is an imaging test used to visualize the shape and function of the esophagus.

How it works: You drink a thick, chalky liquid containing barium, which coats the inside of your digestive tract. X-ray images are then taken in real-time.

What it shows: In patients with achalasia, the X-ray typically reveals a dilated (widened) esophagus that abruptly narrows at the bottom near the stomach, creating a classic "bird's beak" or "rat tail" appearance. It also shows the delayed emptying of the barium into the stomach.

3. Upper Endoscopy (EGD)

While manometry confirms the diagnosis, an endoscopy is crucial for ruling out other dangerous conditions.

How it works: While you are under sedation, a doctor inserts a thin, flexible tube with a light and camera (endoscope) down your throat to look directly at the lining of your esophagus and stomach.

What it shows: It is primarily used to ensure that a tumor or a stricture (scar tissue) isn't causing a physical blockage that mimics achalasia (a condition known as pseudoachalasia).

Potential Risks and Patient Optimization Suggestions

Potential Risks of Diagnostic Procedures:

Manometry: Can cause temporary discomfort, gagging, a sore throat, or a minor nosebleed during tube insertion.

Endoscopy: Very safe, but carries rare risks associated with sedation, or slight risks of bleeding, infection, or esophageal perforation (a tear in the esophageal wall).

Barium Swallow: Involves exposure to a very low, generally safe amount of ionizing radiation. Barium can also cause temporary constipation.

Optimization & Best Practices for Patients:

Pre-test Fasting: It is absolutely critical to follow your doctor's fasting instructions strictly before these tests. Because achalasia prevents food from clearing the esophagus, undigested food can remain there for hours or days, increasing the risk of aspiration (inhaling food into the lungs) during an endoscopy or manometry.

Symptom Diary: Keep a log of what foods/liquids are hardest to swallow, when you experience regurgitation, and if you have chest pain. This helps the gastroenterologist tailor their approach.

Liquid Diet Prep: If you have severe swallowing difficulties, your doctor may recommend a liquid diet for 24-48 hours prior to the procedure to ensure the esophagus is clear.

Would you like to know about the current treatment options (such as Botox injections, pneumatic dilation, or surgery) available for managing achalasia once it is diagnosed?

**Model Grok-4.3**

Answer text:

Achalasia is diagnosed through a combination of clinical history, imaging studies, endoscopic evaluation, and most importantly, esophageal manometry.

1. Clinical Symptoms (Initial Clue)

Doctors typically suspect achalasia when a patient reports:

Progressive dysphagia (difficulty swallowing) to both solids and liquids

Regurgitation of undigested food or saliva (often at night)

Chest pain or heartburn-like pain

Unintentional weight loss

Nocturnal cough or recurrent aspiration pneumonia

2. Diagnostic Tests (in order of typical use)

A. Barium Swallow (Esophagram) - Often the first imaging test

Classic finding: "Bird-beak" or "rat-tail" appearance at the gastroesophageal junction

Dilated, tortuous esophagus above the narrowing

Delayed emptying of barium into the stomach (sometimes hours later)

Sensitivity is good but not perfect ( about 90 percent ).

B. Upper Endoscopy (EGD)

Performed to rule out pseudoachalasia (especially cancer at the cardia or infiltrating tumors).

Typical findings: dilated esophagus, retained food/saliva, and a tight lower esophageal sphincter (LES) that the scope can usually pass through with gentle pressure.

Normal endoscopy does not rule out achalasia.

C. High-Resolution Esophageal Manometry (HRM) - The Gold Standard This is the definitive test required to confirm the diagnosis and classify the subtype.

Key findings on HRM:

Absent peristalsis (no normal swallowing waves) in the esophageal body

Impaired relaxation of the lower esophageal sphincter (elevated integrated relaxation pressure - IRP)

Chicago Classification v4.0 divides it into three subtypes:

Type I (classic): no pressurization

Type II (best prognosis with treatment): panesophageal pressurization

Type III (spastic): premature/spastic contractions

D. Additional Tests (used selectively)

Functional Lumen Imaging Probe (FLIP): Measures esophageal distensibility; increasingly used, especially when manometry is inconclusive.

CT scan or Endoscopic Ultrasound (EUS): If pseudoachalasia (cancer) is strongly suspected.

Timed Barium Esophagram: Helps assess severity and can be used to follow treatment response.

Typical Diagnostic Pathway

Patient presents with dysphagia to Barium swallow

Suggestive of achalasia to Upper endoscopy (to exclude malignancy)

Endoscopy compatible to High-resolution manometry for confirmation and subtyping

Manometry confirms to Treatment planning (pneumatic dilation, Heller myotomy, POEM, or Botox depending on age, subtype, and comorbidities)

Important: Because early achalasia can mimic GERD, many patients are initially misdiagnosed and treated for reflux for months or years. Any patient with dysphagia to liquids should raise suspicion for a motility disorder.

Would you like me to explain the differences between the three Chicago Classification subtypes and how they influence treatment choices?

**Model Qwen3-Max**

Answer text:

Achalasia is diagnosed through a combination of clinical evaluation and specific diagnostic tests that assess esophageal motility and anatomy. The key diagnostic methods include:

1. High-Resolution Esophageal Manometry (HRM) - Gold Standard

Measures pressure and coordination of muscle contractions in the esophagus.

Classic findings in achalasia:

Absent peristalsis in the esophageal body.

Incomplete or absent relaxation of the lower esophageal sphincter (LES) during swallowing.

Elevated resting LES pressure (though not always present).

HRM also helps classify achalasia into subtypes (Type I, II, or III) per the Chicago Classification, which can guide treatment.

2. Barium Swallow (Esophagram)

A radiographic test where the patient swallows barium while X-ray images are taken.

Typical findings:

"Bird's beak" tapering at the LES.

Dilated esophagus with retained barium.

Lack of peristalsis; barium may remain in the esophagus for minutes.

3. Upper Endoscopy (Esophagogastroduodenoscopy, EGD)

Primarily used to rule out other conditions (e.g., esophageal cancer, stricture, or pseudoachalasia).

May show:

Dilated esophagus with retained food/fluid.

Tight LES that resists passage of the endoscope ("pop" sign).

Biopsies are usually normal but help exclude malignancy or eosinophilic esophagitis.

4. Timed Barium Esophagogram (Optional but Useful)

Used to assess treatment response by measuring how much barium remains in the esophagus after 1, 2, and 5 minutes.

Note: Diagnosis should not rely solely on endoscopy or symptoms. Manometry is essential for confirmation.

Red Flags Prompting Further Workup

Rapid weight loss, age greater than 50 at onset, or anemia may suggest pseudoachalasia (e.g., due to a tumor), warranting CT imaging or endoscopic ultrasound.

Would you like to know how the different subtypes of achalasia influence treatment choices?

**Q14. What tests are needed to confirm achalasia?**

**Model ChatGPT 5.5**

Answer text:

To confirm achalasia, the key test is high-resolution esophageal manometry (HRM). Other tests are usually done to support the diagnosis and rule out mimics.

Main tests used

Upper endoscopy - EGD

Looks inside the esophagus and stomach.

Helps rule out cancer, strictures, eosinophilic esophagitis, or other mechanical blockages that can mimic achalasia.

May show retained food/saliva, a dilated esophagus, or a tight/puckered lower esophageal sphincter, but endoscopy alone usually does not confirm achalasia.

Barium swallow / esophagram

You swallow contrast while X-rays are taken.

Classic findings include a dilated esophagus, delayed emptying, and a narrowed "bird-beak" appearance at the gastroesophageal junction.

A timed barium esophagram is especially useful to measure how well the esophagus empties and can serve as a baseline before treatment.

High-resolution esophageal manometry - confirmatory/gold-standard test

Measures pressure and muscle contractions in the esophagus.

Confirms achalasia by showing impaired relaxation of the lower esophageal sphincter plus absent or abnormal peristalsis.

Also classifies achalasia into Type I, II, or III, which helps guide treatment choice. Current ACG and ASGE guidance identify HRM as the criterion/gold-standard diagnostic test. (giboardreview.com)

Sometimes used if results are unclear

FLIP - functional lumen imaging probe

Often performed during endoscopy in specialized centers.

Measures how well the esophagogastric junction opens/distends.

Can help when manometry or other tests are inconclusive.

Typical diagnostic sequence

Usually:

EGD to rule out blockage or cancer to barium esophagram to assess anatomy/emptying to HRM to confirm and subtype achalasia.

If you have rapid weight loss, new symptoms at older age, vomiting blood, black stools, or progressive trouble swallowing, evaluation should be more urgent because pseudoachalasia from malignancy needs to be excluded.

A good next step would be to ask your gastroenterologist whether you've had high-resolution manometry, because that is usually the decisive test.

**Model Claude Opus 4.7**

Answer text:

Tests to Confirm Achalasia

Achalasia is typically diagnosed using a combination of the following tests:

1. Esophageal Manometry (Gold Standard)

The definitive test for diagnosis

Measures pressure and muscle contractions in the esophagus

Confirms three hallmark findings:

Failure of the lower esophageal sphincter (LES) to relax with swallowing

Absence of normal peristalsis (esophageal contractions)

Often elevated LES resting pressure

High-resolution manometry (HRM) classifies achalasia into three subtypes (Types I, II, III) using the Chicago Classification, which helps guide treatment

2. Barium Swallow (Esophagram)

X-ray study after drinking barium contrast

Classic findings include:

"Bird's beak" appearance (narrowing at the LES)

Dilated esophagus above the narrowing

Delayed emptying of barium

Sometimes a sigmoid-shaped esophagus in advanced cases

Timed barium swallow can quantify emptying

3. Upper Endoscopy (EGD)

Essential to rule out pseudoachalasia (mimics caused by cancer at the gastroesophageal junction or other obstruction)

Evaluates the esophageal mucosa

May show retained food/saliva and a dilated esophagus

Allows biopsy if suspicious lesions are present

4. Additional/Adjunctive Tests

EndoFLIP (Functional Lumen Imaging Probe) - measures distensibility of the LES; increasingly used

CT scan - if pseudoachalasia or malignancy is suspected

pH monitoring - occasionally used to differentiate from GERD

Typical Diagnostic Approach

Endoscopy first (rule out obstruction/malignancy)

Barium swallow (suggests diagnosis)

Manometry (confirms diagnosis and subtypes it)

Would you like more details about any specific test or about treatment options once achalasia is confirmed?

**Model DeepSeek V4 Pro**

Answer text:

I'm an AI assistant, not a doctor, so I can't give you personal medical advice. But I can definitely explain the tests that are typically used to confirm achalasia based on current medical guidelines. Always discuss your situation with a gastroenterologist.

Achalasia is a rare motility disorder where the lower esophageal sphincter (LES) fails to relax properly and the esophagus loses normal peristalsis. The diagnosis is usually made with a combination of tests, but high-resolution esophageal manometry is considered the gold standard for confirmation.

Here's the typical workup, in the order it often happens:

1. Upper Endoscopy (EGD)

Purpose: Rule out other causes, especially pseudoachalasia (a tumor at the gastroesophageal junction that can mimic achalasia).

The doctor looks for retained food, a dilated esophagus, and resistance when passing the scope through the LES.

Biopsies may be taken if there's any suspicion of malignancy.

A normal endoscopy doesn't exclude achalasia; it's mainly to make sure there's no mechanical obstruction.

2. Barium Swallow (Esophagram / Timed Barium Swallow)

Purpose: Anatomical and functional screening.

You swallow a barium solution, and X-rays are taken.

Classic signs:

Smooth, tapered narrowing at the LES ("bird's beak" appearance).

Dilated, sometimes tortuous esophagus above.

Delayed emptying of barium into the stomach.

A timed barium swallow (taking X-rays at set intervals, e.g., 1, 2, and 5 minutes) can quantify emptying and assess severity.

It's helpful but not 100 percent diagnostic - some early achalasia can have a normal barium swallow.

3. High-Resolution Esophageal Manometry (HRM)

Purpose: Definitively confirm achalasia and classify its subtype.

A thin catheter is passed through the nose into the esophagus. It measures pressure patterns when you swallow.

According to the Chicago Classification (v4.0), achalasia requires:

Elevated integrated relaxation pressure (IRP) of the LES (usually greater than 15 mmHg, machine-dependent).

Absent peristalsis (100 percent failed swallows) in the esophageal body.

The three subtypes (Type I, II, III) are distinguished by pressurization patterns, which helps guide treatment.

HRM is the gold standard because it directly shows the motility defect. False negatives are rare but can happen if there's esophageal dilation leading to "catheter folding" - experienced centers account for this.

4. Additional Imaging (Selected Cases)

CT scan or endoscopic ultrasound (EUS) - If pseudoachalasia is suspected (e.g., older age, rapid weight loss, symptoms less than 6 months), to look for a hidden tumor or lymphadenopathy compressing the LES.

Functional Lumen Imaging Probe (FLIP) - An emerging tool that measures esophagogastric junction distensibility during endoscopy. It can sometimes detect early achalasia even when manometry is equivocal, though it's not yet the primary diagnostic test in most guidelines.

Potential Risks and Practical Notes

Manometry can cause gagging, nasal discomfort, or minor nosebleed. It's very safe but requires a cooperative patient.

Endoscopy carries the tiny risks of sedation and, rarely, perforation.

Barium swallow involves low radiation exposure and could cause constipation from the barium; not recommended if there's suspicion of perforation (use water-soluble contrast instead).

Since achalasia is rare, sometimes symptoms are misinterpreted as GERD, leading to delays. A careful test sequence is key.

Do you have a specific test you're scheduled for, or are you trying to understand the process for a case? I'm happy to clarify what to expect during a manometry or how the subtypes change treatment decisions.

**Model Gemini 3.1 Pro Thinking**

Answer text:

Disclaimer: I am an AI, not a doctor. The following information is for educational purposes and should not be used as a substitute for professional medical advice, diagnosis, or treatment. Always consult with a healthcare provider regarding medical symptoms.

Achalasia is a rare disorder that makes it difficult for food and liquid to pass from your swallowing tube (esophagus) into your stomach. To confirm a diagnosis of achalasia and rule out other conditions that cause similar symptoms, doctors typically rely on three main tests:

1. Esophageal Manometry (High-Resolution Manometry)

What it is: This is the gold standard test for diagnosing achalasia.

How it works: A thin, flexible tube containing pressure sensors is passed through your nose, down your esophagus, and into your stomach. You will be asked to swallow small sips of water.

What it confirms: It measures the rhythmic muscle contractions of the esophagus when you swallow, the coordination and force exerted by the esophageal muscles, and how well the lower esophageal sphincter (LES) relaxes or fails to relax.

2. Upper Endoscopy (EGD - Esophagogastroduodenoscopy)

What it is: A visual examination of the inside of your esophagus, stomach, and the first part of your small intestine.

How it works: While you are under sedation, a doctor inserts a flexible tube equipped with a light and camera (endoscope) down your throat.

What it confirms: While it cannot definitively diagnose achalasia on its own, it is crucial for ruling out other serious conditions, such as esophageal cancer (which can mimic achalasia, known as pseudoachalasia), strictures, or inflammation. It may also reveal retained food or a dilated esophagus, which are signs of achalasia.

3. Barium Swallow (Esophagram)

What it is: An imaging test that uses a special contrast dye.

How it works: You drink a thick, chalky liquid containing barium, which coats the inside of your digestive tract. X-rays are then taken as the liquid moves down your esophagus.

What it confirms: It provides a silhouette of the esophagus. In a patient with achalasia, the X-ray often shows a dilated (widened) esophagus that abruptly narrows at the bottom, creating a classic "bird's beak" appearance where the sphincter fails to open.

Potential Risks & Best Practice (Optimization) Suggestions

Potential Risks:

Manometry: Can cause temporary discomfort, a gagging sensation, or minor nosebleeds during tube insertion.

Endoscopy: Carries standard risks associated with mild sedation (grogginess, rare breathing issues) and a very small risk of esophageal perforation.

Barium Swallow: Involves a low dose of radiation. Constipation can occur afterward if you do not drink enough water to flush out the barium.

Patient "Optimization" (Best Practices):

See a Specialist: If you suspect achalasia, try to get a referral to a gastroenterologist who specializes in motility disorders. They have the most experience interpreting High-Resolution Manometry results.

Preparation: Strictly follow fasting instructions before these tests (usually fasting for 8+ hours). Because achalasia causes food to be retained in the esophagus, your doctor may recommend a liquid diet for a day or two before an endoscopy to ensure a clear view and prevent aspiration (inhaling food into the lungs).

Next Step: Are you currently experiencing swallowing difficulties that prompted this question, or would you like to know more about the available treatment options (like surgery or balloon dilation) once achalasia is officially diagnosed?

**Model Grok-4.3**

Answer text:

To confirm a diagnosis of achalasia, a motility disorder where the esophagus fails to relax properly and the lower esophageal sphincter (LES) does not open normally, doctors typically follow a stepwise approach. No single test is 100 percent diagnostic on its own; a combination is used.

1. Initial Screening Tests (Usually done first)

Barium swallow (esophagram): Classic finding is a "bird-beak" or "rat-tail" appearance at the gastroesophageal junction with a dilated, tortuous esophagus above it and delayed emptying of barium. This is often the first test that raises strong suspicion for achalasia.

Upper endoscopy (EGD): Performed to rule out pseudoachalasia (especially cancer at the cardia or infiltrating tumors that mimic achalasia). In true achalasia, the esophagus may look dilated with retained food/fluid, but the LES usually allows passage of the scope with mild resistance. Biopsies are taken if any suspicious areas are seen.

2. Confirmatory Test (Gold Standard)

High-Resolution Esophageal Manometry (HRM): This is the definitive test required to confirm achalasia. Key diagnostic findings (Chicago Classification v4.0):

Absent or failed peristalsis in the esophageal body (100 percent of swallows ineffective).

Impaired LES relaxation - elevated integrated relaxation pressure (IRP greater than 15 mmHg in most systems).

Subtypes are then classified:

Type I (classic): no pressurization

Type II (best prognosis with treatment): panesophageal pressurization

Type III (spastic): premature contractions

3. Additional/Supportive Tests (often used)

Timed Barium Esophagram (TBE): More quantitative version of the barium swallow. Measures height of the barium column at 1 and 5 minutes. Very useful both for diagnosis and to assess treatment response later.

Functional Lumen Imaging Probe (FLIP): Sometimes used during endoscopy. Measures esophageal distensibility and can detect reduced LES distensibility even when manometry is equivocal.

CT chest/abdomen or Endoscopic Ultrasound (EUS): Occasionally performed if there is concern for malignancy or pseudoachalasia (especially in older patients with rapid weight loss and short duration of symptoms).

Typical Diagnostic Pathway

Patient presents with dysphagia (to solids and liquids), regurgitation, chest pain, or weight loss.

Barium swallow to suggestive of achalasia.

Endoscopy to rules out cancer or mechanical obstruction.

High-resolution manometry to confirms diagnosis and determines subtype.

Important: Manometry is essential before any definitive treatment (pneumatic dilation, Heller myotomy, or peroral endoscopic myotomy - POEM), because the subtype influences treatment choice and expected success rates.

Would you like me to explain the differences between the three manometric subtypes of achalasia, or outline the pros and cons of the main treatment options based on subtype?

**Model Qwen3-Max**

Answer text:

To confirm a diagnosis of achalasia, a combination of clinical evaluation and specific diagnostic tests is typically used. Achalasia is a primary esophageal motility disorder characterized by failure of the lower esophageal sphincter (LES) to relax during swallowing and absent or abnormal peristalsis in the esophageal body.

The key diagnostic tests include:

1. High-Resolution Esophageal Manometry (HRM) - Gold Standard

Purpose: Measures pressure and coordination of muscle contractions in the esophagus.

Findings in achalasia:

Incomplete LES relaxation (integrated relaxation pressure [IRP] greater than 15 mmHg)

Absent peristalsis in the esophageal body

May show elevated resting LES pressure

HRM also allows classification into subtypes (I, II, III) per the Chicago Classification, which can guide treatment.

2. Barium Swallow (Esophagram)

Purpose: Radiologic imaging to assess esophageal structure and function.

Classic findings:

"Bird's beak" tapering at the LES

Dilated esophagus with retained barium

Lack of peristalsis ("smooth" contractions or aperistalsis)

Useful when manometry is unavailable or to evaluate for complications (e.g., megaesophagus).

3. Upper Endoscopy (Esophagogastroduodenoscopy, EGD)

Purpose: Primarily to rule out mimics, especially pseudoachalasia (e.g., due to gastric or esophageal cancer obstructing the LES).

Findings in achalasia:

Food/liquid retention in a dilated esophagus

Tight LES that may require gentle passage with the scope

Normal mucosa (no inflammation or stricture unless from stasis)

Not diagnostic alone, but essential to exclude mechanical obstruction or malignancy.

Additional Considerations:

Timed barium esophagram: Sometimes used to monitor treatment response.

Endoscopic ultrasound (EUS): If pseudoachalasia is suspected (e.g., to detect submucosal tumors or lymphadenopathy).

Chest X-ray: May show a widened mediastinum or air-fluid level in a dilated esophagus-but not diagnostic.

Diagnostic Approach Summary:

Start with EGD to rule out mechanical obstruction.

Confirm with high-resolution manometry.

Use barium swallow for supportive evidence or pre-treatment planning.

Important: Always exclude malignancy (pseudoachalasia) before confirming primary achalasia, especially in older patients or those with alarm symptoms (weight loss, anemia, rapid onset).

Would you like guidance on interpreting manometry results or differentiating achalasia subtypes?

**Q15. Can gastroscopy alone diagnose achalasia?**

**Model ChatGPT 5.5**

Answer text:

Usually, no. Gastroscopy/endoscopy alone cannot definitively diagnose achalasia.

Gastroscopy can raise suspicion for achalasia if it shows findings such as retained food/saliva in the esophagus, a dilated esophagus, or a tight/puckered gastroesophageal junction. But it is mainly used to rule out other causes of swallowing difficulty-especially mechanical obstruction, stricture, cancer, or "pseudoachalasia." (gi.org)

The confirmatory/gold-standard test is generally high-resolution esophageal manometry, which measures esophageal muscle contractions and whether the lower esophageal sphincter relaxes properly. (giejournal.org)

A typical diagnostic workup may include:

Upper endoscopy/gastroscopy - to exclude blockage or malignancy and look for clues.

Barium swallow / timed barium esophagram - may show a dilated esophagus with "bird-beak" narrowing.

High-resolution manometry - confirms achalasia and classifies the subtype. (gi.org)

So, if your gastroscopy looked suggestive of achalasia, the next usual step is esophageal manometry unless your gastroenterologist has a specific reason otherwise.

If you're having progressive difficulty swallowing, weight loss, vomiting/regurgitation, anemia, or symptoms that are worsening quickly, it's important to follow up promptly with a gastroenterologist.

**Model Claude Opus 4.7**

Answer text:

Can Gastroscopy Alone Diagnose Achalasia?

No, gastroscopy alone cannot definitively diagnose achalasia, though it plays an important supporting role in the diagnostic process.

Role of Gastroscopy (Upper Endoscopy)

Gastroscopy is typically performed as part of the workup, but its main purposes are:

Excluding other conditions that mimic achalasia (especially pseudoachalasia from tumors at the gastroesophageal junction)

Identifying complications (esophagitis, candidiasis, retained food)

Ruling out malignancy

Endoscopic findings suggestive of achalasia:

Dilated esophagus with retained food/saliva

Tight lower esophageal sphincter (LES) that requires gentle pressure to traverse (but still passable)

Normal-appearing esophageal mucosa

Importantly, endoscopy can appear completely normal in up to 40 percent of achalasia cases, particularly in early disease.

Gold Standard: High-Resolution Esophageal Manometry

Definitive diagnosis requires high-resolution manometry (HRM), which demonstrates:

Impaired LES relaxation (elevated integrated relaxation pressure)

Absent peristalsis in the esophageal body

Manometry also classifies achalasia into subtypes I, II, and III (Chicago Classification), which guides treatment.

Complete Diagnostic Workup

A standard evaluation includes:

Barium esophagram - may show the classic "bird's beak" tapering and dilated esophagus

Upper endoscopy (gastroscopy) - to exclude mechanical obstruction/malignancy

High-resolution manometry - confirms the diagnosis

Bottom Line

Gastroscopy is necessary but not sufficient. If achalasia is suspected based on symptoms (dysphagia to solids and liquids, regurgitation, weight loss, chest pain), manometry should be performed to confirm the diagnosis-even if endoscopy looks normal.

**Model DeepSeek V4 Pro**

Answer text:

No, gastroscopy (upper endoscopy) alone cannot definitively diagnose achalasia.

Why not?

Achalasia is a motility disorder of the esophagus-its hallmark is failed relaxation of the lower esophageal sphincter and absent peristalsis, which are functional abnormalities that endoscopy cannot measure.

Gastroscopy is structural, not functional: It shows the inner lining (mucosa) and can spot masses, strictures, or inflammation, but it cannot quantify pressure or muscle contraction.

False reassurance: Up to 40 percent of achalasia patients may have a normal-appearing esophagus on endoscopy in early stages.

Pseudoachalasia: Malignant stricture (e.g., gastroesophageal junction tumor) can mimic achalasia both in symptoms and endoscopic appearance. A biopsy during gastroscopy is crucial to rule this out.

What gastroscopy can do (clues, but not diagnosis)

Suggest achalasia if it finds a dilated esophagus with retained saliva/food, tight LES giving a "pop" on passage, or candida esophagitis from stasis.

Exclude mechanical obstruction (tumor, peptic stricture, rings).

Obtain biopsies to look for malignancy or eosinophilic esophagitis.

What is actually needed for diagnosis

High-resolution esophageal manometry (HRM) - the gold standard, shows impaired LES relaxation and absent peristalsis.

Timed barium swallow - complementary, may show a "bird's beak" narrowing and delayed esophageal emptying.

Important: If you or someone you're asking for is being evaluated for dysphagia, always pursue manometry even if endoscopy is normal. Achalasia can be missed otherwise.

Is there a particular symptom or testing step you'd like to dive deeper into-like how timed barium swallow compares to manometry, or what treatment options follow diagnosis?

**Model Gemini 3.1 Pro Thinking**

Answer text:

Disclaimer: I am an AI, not a doctor. The following information is for educational purposes and should not replace professional medical advice. Always consult a gastroenterologist or healthcare provider for an accurate diagnosis.

No, gastroscopy (upper endoscopy) alone cannot definitively diagnose achalasia.

While gastroscopy is a crucial part of the diagnostic process, its primary role is to rule out other conditions rather than to confirm achalasia itself. Here is a breakdown of why this is the case and how achalasia is actually diagnosed:

The Role of Gastroscopy in Achalasia

Achalasia is a motility disorder where the nerves in the esophagus are damaged, causing the esophageal muscles to lose their ability to squeeze (peristalsis) and the lower esophageal sphincter (LES) to fail to relax. During a gastroscopy, a doctor might see supportive signs of achalasia, such as:

A dilated (widened) esophagus.

Retained food or liquid in the esophagus despite fasting.

A tight lower esophageal sphincter that offers mild resistance but eventually "pops" open when the endoscope is gently pushed through.

However, in the early stages of achalasia, the esophagus may look completely normal during a gastroscopy. Therefore, a normal gastroscopy does not rule out achalasia.

Why is Gastroscopy Required Then?

Gastroscopy is absolutely essential to rule out mechanical obstructions or malignancies. Some cancers (like esophageal or stomach cancer at the gastroesophageal junction) can mimic the symptoms and x-ray findings of achalasia-a condition known as "pseudoachalasia." Endoscopy allows the doctor to visually inspect the tissue and take biopsies to ensure cancer is not the underlying cause.

How is Achalasia Definitively Diagnosed?

To confirm achalasia, doctors rely on tests that evaluate the function and movement of the esophagus:

High-Resolution Esophageal Manometry (Gold Standard): A thin tube is passed through the nose down into the stomach. It measures the rhythmic muscle contractions of the esophagus when you swallow and evaluates how well the LES relaxes. This test is required to definitively diagnose and classify the specific subtype of achalasia.

Barium Swallow (Esophagram): The patient drinks a chalky liquid (barium) while X-rays are taken. In achalasia, this often reveals a dilated esophagus that narrows sharply at the bottom, creating a classic "bird's beak" appearance.

Potential Risks & Clinical Optimization

Risk of Misdiagnosis: Relying solely on gastroscopy can lead to missing early-stage achalasia (resulting in delayed treatment) or confusing true achalasia with pseudoachalasia (tumor).

Diagnostic Optimization: The best practice for a patient presenting with progressive difficulty swallowing (dysphagia) to both solids and liquids is a multi-step approach: start with a gastroscopy to rule out structural damage/cancer, followed by a barium swallow and esophageal manometry to evaluate motility.

Would you like to know more about the treatment options for achalasia once a diagnosis has been confirmed via manometry?

**Model Grok-4.3**

Answer text:

No, gastroscopy (upper endoscopy) alone cannot reliably diagnose achalasia.

Why gastroscopy is insufficient

Gastroscopy is an important initial test in patients with swallowing difficulties, but it has significant limitations for diagnosing achalasia:

Findings are often non-specific or absent: In early achalasia, the endoscopy may appear completely normal. In more advanced cases, it may show a dilated esophagus, retained food/saliva, or resistance when passing the scope through the lower esophageal sphincter (LES), but these signs are not diagnostic.

It cannot evaluate motility: Achalasia is fundamentally a motility disorder (failure of LES relaxation + absent esophageal peristalsis). Gastroscopy cannot measure pressures or contractions.

Main role is exclusion: Its primary value is to rule out mimicking conditions such as esophageal cancer, strictures, or pseudoachalasia (a cancer at the gastroesophageal junction that mimics achalasia).

Standard diagnostic pathway

The definitive diagnosis of achalasia requires:

Clinical suspicion (progressive dysphagia to solids and liquids, regurgitation, chest pain, weight loss).

Barium esophagram - often shows the classic "bird-beak" or "rat-tail" appearance.

High-resolution esophageal manometry (HRM) - this is the gold standard. It confirms the diagnosis and classifies the subtype (Type I, II, or III), which guides treatment.

Endoscopy - performed to exclude malignancy/pseudoachalasia before treatment.

In selected cases, endoscopic ultrasound or CT may be added if pseudoachalasia is strongly suspected.

Bottom line

A normal gastroscopy does not rule out achalasia, and abnormal findings on gastroscopy are not sufficient to confirm it. Manometry is required for a definitive diagnosis.

Would you like me to explain the different types of achalasia (Chicago classification), the manometry findings, or the current treatment options (POEM, Heller myotomy, pneumatic dilation)?

**Model Qwen3-Max**

Answer text:

Gastroscopy (also known as upper endoscopy) alone is not sufficient to definitively diagnose achalasia, although it can raise strong suspicion and help rule out other conditions.

Why gastroscopy isn't enough:

Achalasia is a motility disorder of the esophagus characterized by:

Failure of the lower esophageal sphincter (LES) to relax during swallowing

Absence or impairment of esophageal peristalsis

These are functional abnormalities that cannot be directly visualized with standard endoscopy.

What gastroscopy may show in achalasia:

A dilated esophagus with retained food or fluid

A "tight" or non-relaxing LES that the endoscope passes through with resistance ("pop" sign)

No mechanical obstruction or mucosal abnormality (helps exclude tumors or strictures)

However, these findings are not specific-they can also occur in pseudoachalasia (e.g., due to esophageal or gastric cancer), Chagas disease, or other causes.

Gold standard for diagnosis:

High-resolution manometry (HRM) is the gold standard. It demonstrates:

Absent or impaired peristalsis in the esophageal body

Inadequate LES relaxation (measured by integrated relaxation pressure, IRP)

Often elevated baseline LES pressure

Additional supportive tests:

Barium esophagogram: Shows a "bird's beak" tapering of the distal esophagus, esophageal dilation, and poor emptying.

Endoscopic ultrasound (EUS): May be used if pseudoachalasia is suspected to evaluate for underlying malignancy.

Clinical takeaway:

While gastroscopy is an important initial step to exclude structural causes (like malignancy), achalasia should not be diagnosed on endoscopy alone. Manometry is essential for confirmation.

Next step: If achalasia is suspected based on symptoms (dysphagia to solids and liquids, regurgitation, chest pain) and endoscopic findings, refer the patient for high-resolution manometry.

Would you like guidance on interpreting manometry results or differentiating achalasia subtypes?

**Q16. What is the role of high-resolution esophageal manometry in achalasia?**

**Model ChatGPT 5.5**

Answer text:

High-resolution esophageal manometry HRM is the key diagnostic test for achalasia. Its role is to confirm that the problem is a primary esophageal motility disorder-rather than reflux, stricture, cancer, or another cause of dysphagia-and to classify the achalasia subtype, which can influence treatment planning.

Main roles of HRM in achalasia:

Confirms the diagnosis

Achalasia is defined manometrically by impaired relaxation of the esophagogastric junction/lower esophageal sphincter plus absent or abnormal esophageal peristalsis.

Guidelines describe HRM as the criterion standard/gold-standard test for diagnosing achalasia. (sysge.org)

Classifies achalasia into subtypes HRM identifies three clinically important subtypes based on esophageal body pressure patterns:

Type I/classic achalasia: failed peristalsis with minimal pressurization.

Type II achalasia: failed peristalsis with panesophageal pressurization.

Type III/spastic achalasia: impaired EGJ relaxation with premature/spastic contractions.

This classification comes from HRM-based Chicago Classification systems and is now central to achalasia evaluation. (pmc.ncbi.nlm.nih.gov)

Guides treatment choice Subtype matters:

Type II generally responds very well to pneumatic dilation, laparoscopic Heller myotomy, or POEM.

Type III often requires a longer myotomy, so POEM is frequently favored because it allows extension of the myotomy proximally into the spastic segment.

Type I may respond less robustly than type II but is still treated with LES-disrupting therapy.

Distinguishes achalasia from related disorders HRM helps differentiate achalasia from:

Esophagogastric junction outflow obstruction EGJOO

Distal esophageal spasm

Absent contractility, such as in scleroderma-pattern esophagus

Opioid-related esophageal dysfunction

Chicago Classification v4.0 emphasizes more rigorous HRM protocols, including different body positions and provocative maneuvers, to reduce overdiagnosis of borderline disorders such as EGJOO. (pmc.ncbi.nlm.nih.gov)

Complements, but does not replace, endoscopy and barium esophagram Endoscopy is important to exclude pseudoachalasia, malignancy, stricture, or mechanical obstruction. Barium esophagram can show classic "bird-beak" narrowing or delayed emptying. But HRM is what physiologically confirms achalasia. (sysge.org)

Bottom line: HRM is central in achalasia because it confirms the diagnosis, defines the subtype, helps exclude mimics, and guides the most appropriate therapy.

Would you like a quick table comparing type I, II, and III achalasia and their preferred treatments?

**Model Claude Opus 4.7**

Answer text:

Role of High-Resolution Esophageal Manometry (HRM) in Achalasia

High-resolution manometry is the gold standard for diagnosing achalasia and plays several critical roles in its management.

1. Diagnostic Confirmation

HRM definitively diagnoses achalasia by demonstrating the two cardinal features:

Impaired relaxation of the lower esophageal sphincter (LES) - elevated integrated relaxation pressure (IRP greater than 15 mmHg)

Absent peristalsis in the esophageal body

It is typically performed after endoscopy and barium swallow have excluded mechanical obstruction (pseudoachalasia).

2. Subtype Classification (Chicago Classification)

HRM uniquely allows classification into three clinically relevant subtypes:

Type Manometric Finding Treatment Response

Type I (classic) Absent peristalsis, no pressurization Intermediate response

Type II Panesophageal pressurization in at least 20 percent swallows Best response to all therapies

Type III (spastic) Premature/spastic contractions in at least 20 percent swallows Worst response; may favor POEM

3. Guiding Treatment Selection

Subtype influences therapy choice:

Type II to excellent outcomes with pneumatic dilation, Heller myotomy, or POEM

Type III to POEM preferred (allows longer myotomy to address spastic segment)

Type I to surgical myotomy or POEM generally favored over dilation

4. Prognostic Value

Subtype predicts long-term symptom relief and helps set patient expectations.

5. Post-Treatment Assessment

HRM can evaluate persistent or recurrent symptoms after intervention, assessing residual LES pressure and the need for further therapy.

6. Differentiating Related Disorders

HRM distinguishes achalasia from:

EGJ outflow obstruction (peristalsis preserved)

Distal esophageal spasm and jackhammer esophagus (normal LES relaxation)

Limitations

Cannot reliably distinguish primary achalasia from pseudoachalasia (malignancy-related) - endoscopy/imaging required

May yield equivocal results requiring adjunctive testing (e.g., FLIP planimetry, timed barium esophagram)

Bottom line: HRM is essential not only for diagnosis but also for phenotyping achalasia into subtypes that directly guide individualized therapy and predict outcomes.

**Model DeepSeek V4 Pro**

Answer text:

High-resolution esophageal manometry (HRM) is the gold-standard diagnostic tool for achalasia and plays a central role far beyond simply confirming the diagnosis. Its main contributions are:

1. Primary Diagnosis

According to the Chicago Classification v4.0, HRM establishes the diagnosis of achalasia by demonstrating two essential findings:

Impaired lower esophageal sphincter (LES) relaxation: median integrated relaxation pressure (IRP) greater than upper limit of normal (typically greater than 15 mmHg in the supine position, depending on the system).

Absent peristalsis: 100 percent failed swallows (failed peristalsis) in the esophageal body.

These criteria differentiate achalasia from other motor disorders like distal esophageal spasm or hypercontractile esophagus.

2. Subtype Classification

HRM is unique in its ability to classify achalasia into three clinically meaningful subtypes based on the pattern of esophageal body pressurization during swallows. The Chicago Classification v4.0 defines them as:

Type I (classic achalasia): No significant pressurization within the esophageal body (integrated relaxation pressure is elevated, but panesophageal pressurization does not occur).

Type II (achalasia with esophageal pressurization): at least 20 percent of swallows show panesophageal pressurization ( greater than 30 mmHg isodobaric contour extending from the upper to the lower esophageal sphincter).

Type III (spastic achalasia): at least 20 percent of swallows exhibit premature (spastic) contractions (distal latency less than 4.5 s), with or without panesophageal pressurization.

This subtyping cannot be achieved by barium swallow or endoscopy alone.

3. Predicting Treatment Response and Guiding Therapy

The subtype directly influences prognosis and treatment selection:

Type II shows the best response to any form of LES disruption (pneumatic dilation, Heller myotomy, POEM). It is the most common subtype and has excellent outcomes across therapies.

Type I has an intermediate prognosis; treatment is still effective but may require a more aggressive approach (e.g., larger balloon diameter).

Type III is the most refractory to standard LES-targeted therapies. Because spastic contractions often involve the esophageal body, these patients derive greater benefit from per-oral endoscopic myotomy (POEM) with an extended myotomy (covering the spastic segment) or from a long surgical myotomy. Pneumatic dilation alone often fails to relieve dysphagia.

Thus, HRM phenotype directly steers the choice between pneumatic dilation, Heller myotomy, and POEM, and defines the extent of myotomy.

4. Detection of Early or Variant Forms

HRM can identify esophagogastric junction outflow obstruction (EGJOO), a manometric pattern with elevated IRP but preserved peristalsis. While not achalasia, a subset of these patients may progress, and manometry helps decide if further workup (e.g., timed barium esophagogram, CT/EUS) is needed to exclude mechanical obstruction or pseudoachalasia.

5. Follow-up and Post-treatment Assessment

Although timed barium esophagogram is the primary tool for following achalasia treatment, HRM can be used to:

Objectively document reduction in IRP after therapy.

Detect incomplete myotomy or disease progression (e.g., recurrence of spastic features).

Investigate persistent symptoms when imaging results are equivocal.

Potential Pitfalls and Optimisation

Inadequate study quality: Poor catheter positioning, too few valid swallows, or artifacts can lead to misclassification. At least 10 supine liquid swallows are recommended; adjunctive solid swallows or rapid drink challenge can unmask latent outflow obstruction.

Pseudoachalasia: Malignant infiltration at the gastro-esophageal junction can mimic achalasia on HRM. Red flags (older age, abrupt weight loss, short symptom duration) warrant cross-sectional imaging and endoscopic ultrasound.

Software and diagnostic thresholds: IRP cut-offs vary slightly between systems (Sierra/Sandhill vs. ManoScan); always reference system-specific norms and the latest Chicago Classification version.

Interpretation in context: The manometric diagnosis must always be integrated with symptoms and complementary tests (endoscopy, timed barium esophagogram) to avoid over-treating isolated manometric findings.

In summary, HRM is irreplaceable for diagnosing achalasia, stratifying it into prognostic subtypes, and personalising invasive therapy-ultimately improving symptom relief and reducing the need for repeat interventions.

Would you like to discuss how the choice between pneumatic dilation, Heller myotomy, and POEM might change based on a specific subtype, or are you interested in the technical steps of a rapid drink challenge during manometry?

**Model Gemini 3.1 Pro Thinking**

Answer text:

Disclaimer: The following information is for educational purposes only and should not be considered professional medical advice. Always consult with a qualified healthcare provider or gastroenterologist for medical diagnosis and treatment decisions.

High-resolution esophageal manometry (HRM) is currently considered the gold standard for diagnosing achalasia. It represents a significant technological leap over conventional manometry by using a catheter with numerous, closely spaced pressure sensors (typically 36) to create a highly detailed, color-coded topographical map of esophageal pressure.

Here is a breakdown of the critical roles HRM plays in the management of achalasia:

1. Definitive Diagnosis

Achalasia is characterized by two main physiological defects: the failure of the lower esophageal sphincter (LES) to relax during swallowing, and the absence of normal peristalsis (muscle contractions) in the esophageal body. HRM precisely measures the Integrated Relaxation Pressure (IRP). An abnormally high IRP confirms that the LES is not relaxing properly, which is the hallmark of achalasia. It also definitively proves the absence of normal peristaltic waves.

2. Subtyping via the Chicago Classification

This is perhaps the most important clinical role of HRM. By analyzing the pressure patterns, HRM classifies achalasia into three distinct subtypes using the Chicago Classification system. This is crucial because each subtype responds differently to specific treatments:

Type I (Classic Achalasia): Characterized by absent peristalsis and no significant pressurization within the esophagus.

Type II (Achalasia with Pan-esophageal Pressurization): Characterized by absent peristalsis but features simultaneous pressurization of the entire esophagus when swallowing. This subtype generally has the best response to treatment.

Type III (Spastic Achalasia): Characterized by absent normal peristalsis but accompanied by premature, spastic contractions. This is the most difficult subtype to treat.

3. Guiding Treatment Selection

Because HRM identifies the specific subtype, it directly dictates the surgical or endoscopic approach:

Type I and II: Often respond well to Pneumatic Dilation (PD) or Laparoscopic Heller Myotomy (LHM).

Type III: Traditional LHM often fails because the spastic contractions occur higher up in the esophagus than the surgery reaches. For Type III, POEM (Peroral Endoscopic Myotomy) is heavily favored because the surgeon can cut a longer segment of the esophageal muscle to address the spasms.

4. Post-Treatment Monitoring

If a patient experiences recurrent dysphagia (difficulty swallowing) or chest pain after treatment, HRM can be repeated to determine if the LES is failing to relax again, if spasms have returned, or if the symptoms are due to a new issue, such as severe acid reflux caused by the initial surgery.

Potential Risks and Limitations of HRM

While generally very safe, HRM does carry some risks and limitations:

Patient Discomfort: The passing of the catheter through the nose and into the stomach can cause gagging, coughing, a sore throat, and minor nasal bleeding.

Aspiration Risk: Patients with achalasia often have retained food and liquid in their esophagus. Placing a catheter can induce vomiting, leading to a risk of aspiration.

Catheter Placement Failure: In advanced achalasia, the esophagus can become massively dilated and tortuous (a "sigmoid esophagus"). The catheter may curl in the esophagus and fail to pass through the LES into the stomach.

Rare Complications: Esophageal or gastric perforation is exceptionally rare but theoretically possible, especially if there is an underlying structural abnormality.

Optimization and Clinical Best Practices

To ensure an accurate and safe HRM study in suspected achalasia patients:

Dietary Preparation: Because achalasia patients retain food, a standard 8-hour fast is often insufficient. Best practice may require a clear liquid diet for 24-48 hours before the procedure to ensure the esophagus is clear of debris.

Prior Imaging: A timed Barium Swallow X-ray or endoscopy should ideally be performed before manometry. This allows the physician to rule out a physical blockage (like a tumor, known as "pseudoachalasia") and identify if a tortuous sigmoid esophagus is present, which alters how the manometry catheter is safely placed.

Medication Management: Medications that affect esophageal motility (such as calcium channel blockers, nitrates, or opioids) should generally be paused prior to the study, as they can artificially alter pressure readings and mask the diagnosis.

Would you like to know more about how the specific treatments (like POEM or Heller Myotomy) are performed, or how they compare in terms of recovery and outcomes?

**Model Grok-4.3**

Answer text:

High-resolution esophageal manometry (HRM) is the gold-standard diagnostic test for achalasia.

Primary Role

HRM definitively confirms the diagnosis of achalasia and classifies it into clinically relevant subtypes. It has largely replaced conventional manometry because it provides greater accuracy, reproducibility, and detailed pressure topography through 36 closely spaced pressure sensors that generate color-contour plots (esophageal pressure topography).

Key Diagnostic Criteria (Chicago Classification v4.0)

HRM establishes the diagnosis when both of the following are present:

Elevated median Integrated Relaxation Pressure (IRP) - usually greater than 15 mmHg (threshold varies slightly by catheter and lab), indicating impaired relaxation of the lower esophageal sphincter (LES) and esophagogastric junction (EGJ).

Absent peristalsis (100 percent failed swallows) in the esophageal body.

Subtyping (Most Important Clinical Contribution)

HRM divides achalasia into three subtypes that have strong prognostic and therapeutic implications:

Subtype Manometric Pattern Clinical Features Best Treatment Response

Type I Classic achalasia (no contractility) Dilated esophagus, retained food Good response to myotomy/dilation

Type II Panesophageal pressurization ( greater than 20 percent swallows) Highest success rate Best prognosis; excellent response to any therapy (especially POEM or pneumatic dilation)

Type III Spastic contractions (premature/spastic swallows) Chest pain predominant Poorest prognosis; POEM preferred

Type II is the most common and has the highest treatment success rate ( about 90-95 percent ), while Type III often requires a longer myotomy (typically POEM).

Additional Clinical Roles

Differential diagnosis: Distinguishes achalasia from other motility disorders (e.g., EGJ outflow obstruction, distal esophageal spasm, jackhammer esophagus) and helps identify pseudoachalasia (e.g., due to malignancy).

Pre-procedural planning: Guides choice and extent of intervention (pneumatic dilation, laparoscopic Heller myotomy, or peroral endoscopic myotomy - POEM). For example, Type III patients often do better with POEM than standard Heller myotomy.

Post-treatment assessment: Can be used when symptoms persist after therapy to evaluate adequacy of myotomy or identify complications such as reflux or incomplete myotomy.

Research and physiologic insights: Provides detailed information about esophageal body contractility, EGJ distensibility, and pressurization patterns.

Limitations

HRM is invasive and requires patient cooperation.

It provides limited information about esophageal anatomy or mucosal disease (complementary tests such as timed barium esophagram, FLIP, or endoscopy are often used together).

In summary, HRM is not only essential for making the diagnosis of achalasia but has become indispensable for personalized treatment decisions through its ability to subtype the disease and predict outcomes.

Would you like me to explain the Chicago Classification criteria in more technical detail, compare HRM with FLIP or timed barium esophagram, or discuss how manometric subtype influences the choice between POEM, Heller myotomy, and pneumatic dilation?

**Model Qwen3-Max**

Answer text:

High-resolution esophageal manometry (HRM) plays a central and indispensable role in the diagnosis, classification, and management of achalasia. It is considered the gold standard for diagnosing this motility disorder.

Key Roles of HRM in Achalasia:

Definitive Diagnosis:

Achalasia is characterized by failure of the lower esophageal sphincter (LES) to relax and absent or abnormal peristalsis in the esophageal body.

HRM objectively measures:

Incomplete LES relaxation (measured by integrated relaxation pressure [IRP]-typically elevated greater than 15 mmHg).

Aperistalsis (lack of coordinated contractions in the esophageal body).

Chicago Classification:

HRM enables classification of achalasia into three main subtypes (per the Chicago Classification v4.0):

Type I (classic): Minimal contractility in the esophageal body.

Type II: Pan-esophageal pressurization ( at least 20 percent of swallows)-most responsive to therapy.

Type III (spastic): Premature or spastic contractions-more challenging to treat.

This classification guides treatment decisions and predicts therapeutic outcomes.

Differentiation from Mimics:

HRM helps distinguish achalasia from other conditions like:

Esophageal outflow obstruction (e.g., due to stricture or tumor).

Functional dysphagia.

Jackhammer esophagus or diffuse esophageal spasm.

This is critical because treatments differ significantly.

Pre-Therapeutic Assessment:

Before interventions like pneumatic dilation, peroral endoscopic myotomy (POEM), or surgical Heller myotomy, HRM confirms the diagnosis and subtype.

It also assesses esophageal pressurization patterns that may influence procedural choice.

Post-Treatment Evaluation (less common but useful):

In cases of persistent or recurrent symptoms, HRM can evaluate for:

Incomplete myotomy.

Development of reflux or other motility disorders.

Clinical Workflow Example:

A patient presents with dysphagia to solids and liquids, regurgitation, and chest pain.

Endoscopy rules out mechanical obstruction.

HRM is performed to shows elevated IRP and absent peristalsis to confirms achalasia.

Subtype determined to guides decision toward POEM (especially for Type II/III) or pneumatic dilation.

Note: HRM should be interpreted alongside clinical findings, barium esophagography (which may show "bird's beak" tapering), and endoscopy to exclude pseudoachalasia (e.g., from malignancy).

Would you like a comparison of HRM findings across achalasia subtypes or guidance on interpreting an HRM tracing?

**Q17. What does a barium swallow show in achalasia?**

**Model ChatGPT 5.5**

Answer text:

In achalasia, a barium swallow classically shows:

Dilated esophagus above the lower esophageal sphincter

Smooth tapering narrowing at the gastroesophageal junction, often called a "bird-beak" or "rat-tail" appearance

Poor or absent peristalsis in the esophageal body

Delayed emptying of barium into the stomach

Sometimes an air-fluid level or retained food/fluid in a markedly dilated esophagus

The key exam phrase is: "bird-beak narrowing of the distal esophagus with proximal esophageal dilatation."

Would you like a quick comparison of barium swallow findings in achalasia vs esophageal cancer/pseudoachalasia?

**Model Claude Opus 4.7**

Answer text:

A barium swallow in achalasia typically shows several characteristic findings:

Classic Findings

"Bird's beak" or "rat-tail" appearance - The hallmark sign: smooth, symmetrical, tapered narrowing at the lower esophageal sphincter (LES) due to failure of relaxation.

Dilated esophagus - Proximal esophageal dilatation above the narrowed segment, which can become massive in advanced disease ("sigmoid" or "megaesophagus" appearance).

Delayed emptying of barium - Contrast pools above the LES and empties slowly into the stomach.

Absent or abnormal peristalsis - Loss of normal primary peristaltic waves; tertiary (non-propulsive) contractions may be seen.

Air-fluid level - Often visible in the dilated esophagus on upright films, with absence of a gastric air bubble.

Retained food/debris - Mottled appearance within the esophagus from residual contents.

Additional Features

Loss of the normal gastric air bubble

In advanced disease: tortuous, sigmoid-shaped esophagus

Timed barium swallow can quantify emptying - useful for assessing severity and treatment response (measuring barium column height at 1, 2, and 5 minutes)

Clinical Note

While barium swallow is suggestive, high-resolution esophageal manometry is the gold standard for diagnosis, and endoscopy is required to exclude pseudoachalasia (e.g., malignancy at the gastroesophageal junction).

**Model DeepSeek V4 Pro**

Answer text:

In achalasia, a barium swallow (esophagram) typically shows a characteristic set of findings reflecting impaired lower esophageal sphincter (LES) relaxation and absent peristalsis:

"Bird's beak" sign: The distal esophagus tapers smoothly to a tight, beak-like narrowing at the gastroesophageal junction (GEJ) due to a non-relaxing LES that fails to open.

Dilated esophagus: The esophageal body becomes progressively widened, often to a massive degree, and may appear flaccid or tortuous (megaesophagus in advanced cases).

Air-fluid level: In the upright position, retained food, liquid, and secretions create a horizontal air-fluid level in the dilated esophagus.

Delayed emptying: Barium fails to pass into the stomach promptly, often requiring gravity or prolonged observation to trickle through the narrowed segment.

Aperistalsis: On dynamic fluoroscopy, normal primary peristaltic waves are absent; instead, simultaneous, non-propulsive tertiary contractions may be seen (especially in earlier stages or vigorous achalasia).

Smooth mucosal appearance: The narrowed segment has smooth contours, distinguishing it from malignant strictures.

Clinical note: While still widely used, barium studies primarily reveal structural and functional consequences; high-resolution esophageal manometry is the gold standard for diagnosis.

Do you want me to explain how achalasia subtypes (e.g., Type I, II, III) differ on manometry and how that guides treatment?

**Model Gemini 3.1 Pro Thinking**

Answer text:

A barium swallow (esophagram) is a key diagnostic imaging test used to evaluate swallowing disorders. In a patient with achalasia-a rare condition where the esophagus loses its ability to squeeze food down and the lower esophageal sphincter (LES) fails to relax-a barium swallow typically reveals several classic, highly distinct features.

Here is what a barium swallow typically shows in someone with achalasia:

The "Bird's Beak" (or "Rat-Tail") Deformity: This is the most famous hallmark of achalasia. As the barium reaches the lower esophagus, it funnels down into a smooth, symmetrical, and sharp tapering point where the LES remains tightly closed. It looks remarkably like the beak of a bird.

Esophageal Dilation: Above the narrowed LES, the body of the esophagus often appears significantly widened (dilated). In advanced or late-stage achalasia, it can become massively dilated and take on a twisted or "sigmoid" appearance (megaesophagus).

Absent Peristalsis (Aperistalsis): During the real-time fluoroscopy (video X-ray) part of the exam, the radiologist will observe a lack of normal, coordinated muscle contractions (peristalsis) in the lower two-thirds of the esophagus. The barium simply falls by gravity rather than being actively pushed down.

Delayed Emptying: The barium liquid pools in the dilated esophagus and empties very slowly into the stomach, often requiring the hydrostatic pressure of a large column of fluid to force a small amount through the unrelaxed sphincter.

Air-Fluid Level: Because food, saliva, and liquids are retained in the esophagus, an upright X-ray will often show a distinct horizontal line where the resting fluid meets the trapped air above it.

Potential Risk & Health Notice:Please note that while a barium swallow is an excellent screening tool, it is not always 100 percent definitive on its own, especially in early achalasia where the esophagus may not yet be dilated. This information is for educational purposes. If you or someone you know is experiencing difficulty swallowing (dysphagia), regurgitation, or chest pain, it is critical to consult a gastroenterologist or physician for a proper clinical evaluation.

Would you like to know about how achalasia is treated (such as POEM or pneumatic dilation), or would you prefer to learn about esophageal manometry, which is the "gold standard" test used to confirm this diagnosis?

**Model Grok-4.3**

Answer text:

A barium swallow (esophagram) is one of the most characteristic and useful initial tests for diagnosing achalasia.

Classic Findings on Barium Swallow in Achalasia

"Bird-beak" or "Rat-tail" appearance

The most iconic sign.

The distal esophagus tapers smoothly and symmetrically to a narrow point at the lower esophageal sphincter (LES), resembling the beak of a bird.

This reflects the failure of the LES to relax.

Dilated (megaesophagus) and tortuous esophagus

The body of the esophagus is often markedly dilated above the narrowing.

In longstanding cases, it can become sigmoid-shaped (tortuous).

Absent or markedly reduced peristalsis

Normal primary peristaltic waves are absent.

You may see tertiary (non-propulsive) contractions or a completely atonic esophagus.

Delayed emptying / barium column

Barium often pools in the esophagus and empties very slowly into the stomach.

The height of the retained barium column is increased (especially on upright films).

"Champagne glass" sign (sometimes described)

The appearance of the contrast column above the bird-beak tapering.

Additional Notes

Early achalasia: The esophagus may not be significantly dilated yet; the bird-beak tapering and poor LES relaxation may be the only clues.

Vigorous achalasia: May show prominent tertiary contractions that can mimic diffuse esophageal spasm.

Sensitivity: A good barium swallow has about 90-95 percent sensitivity for achalasia when the classic bird-beak is present. However, it is not 100 percent specific - pseudoachalasia (e.g., from a tumor at the gastroesophageal junction) can look very similar.

Typical Radiologist Report Phrasing

"There is smooth, tapered narrowing of the distal esophagus with a bird-beak appearance, associated esophageal dilation, absent primary peristalsis, and delayed emptying of barium into the stomach. These findings are highly suggestive of achalasia."

Gold-standard confirmatory test after a suggestive barium swallow is high-resolution esophageal manometry, which definitively shows absent peristalsis and impaired LES relaxation (elevated integrated relaxation pressure).

Follow-up question: Would you like me to also describe the typical manometry findings in achalasia, or explain how to differentiate achalasia from pseudoachalasia on imaging?

**Model Qwen3-Max**

Answer text:

A barium swallow (also called an esophagram) is a key diagnostic imaging test for achalasia, and it typically shows several characteristic findings:

Classic Radiographic Features of Achalasia on Barium Swallow:

Dilated esophagus - The esophagus above the lower esophageal sphincter (LES) is often markedly dilated due to impaired emptying.

"Bird's beak" or "rat tail" tapering - Smooth, symmetric narrowing at the gastroesophageal junction caused by failure of the LES to relax.

Absence of peristalsis - The esophageal body shows little to no primary peristaltic contractions during swallowing.

Barium retention - Contrast material remains in the esophagus for an extended period because it cannot pass easily into the stomach.

Air-fluid level - A visible air-fluid level may be seen in the dilated esophagus on upright films.

These findings reflect the underlying pathophysiology of achalasia: loss of inhibitory neurons in the myenteric plexus, leading to failure of LES relaxation and aperistalsis of the esophageal body.

Note: While barium swallow is highly suggestive, the diagnosis of achalasia is confirmed with high-resolution esophageal manometry, which demonstrates incomplete LES relaxation and absent peristalsis.

Would you like to know how this differs from other esophageal motility disorders like diffuse esophageal spasm or pseudoachalasia?

**Q18. Why do doctors need to rule out esophageal cancer before diagnosing achalasia?**

**Model ChatGPT 5.5**

Answer text:

Doctors need to rule out esophageal or gastroesophageal-junction cancer before diagnosing achalasia because some cancers can mimic achalasia. This mimic is called pseudoachalasia or secondary achalasia.

In true achalasia, the problem is usually a primary nerve/muscle motility disorder: the lower esophageal sphincter does not relax properly and the esophagus loses normal peristalsis. But a tumor near the lower esophagus, gastroesophageal junction, or gastric cardia can produce a very similar picture by physically narrowing the outlet, infiltrating nerves, or stiffening the junction. Symptoms and even barium swallow or manometry findings can overlap. Guidelines therefore recommend upper endoscopy to evaluate the gastroesophageal junction and gastric cardia in suspected achalasia to exclude pseudoachalasia. (mdcalc.com)

The reason this matters is that the treatments are completely different. Achalasia treatments-pneumatic dilation, Heller myotomy, POEM, botulinum toxin-aim to open or weaken the lower esophageal sphincter. If the real cause is cancer, those treatments may delay cancer diagnosis and do not address the underlying tumor. Pseudoachalasia is uncommon, but it is important because it is often caused by malignancy and may be advanced by the time it is recognized. (aoe.amegroups.org)

Doctors are especially cautious if there are "red flags," such as:

Older age at symptom onset, often over 50-60

Rapidly progressive dysphagia, especially over months rather than years

Significant unintentional weight loss

Difficulty passing the endoscope through the gastroesophageal junction

A short history of severe symptoms

Evidence of a mass, irregular narrowing, or abnormal tissue on imaging/endoscopy

The usual workup may include upper endoscopy with biopsies, barium esophagram, and high-resolution manometry. If suspicion remains despite a normal-looking endoscopy, doctors may add CT scan or endoscopic ultrasound, because some tumors can be submucosal or outside the esophageal lining and may not be obvious at first glance.

So, in short: doctors rule out cancer first because cancer can masquerade as achalasia, and missing it could lead to the wrong treatment and a dangerous delay in cancer care. If you're asking because of your own symptoms or test results, I can help you interpret what findings usually point more toward achalasia versus pseudoachalasia.

**Model Claude Opus 4.7**

Answer text:

Doctors need to rule out esophageal cancer before diagnosing achalasia because of a condition called pseudoachalasia, where a tumor mimics the clinical, radiographic, and manometric features of true achalasia.

Key Reasons:

1. Symptom Overlap Both conditions present with:

Progressive dysphagia (difficulty swallowing)

Regurgitation

Weight loss

Chest pain

2. Similar Diagnostic Findings A tumor at the gastroesophageal (GE) junction can cause:

Esophageal dilation above the obstruction

A "bird's beak" appearance on barium swallow

Elevated lower esophageal sphincter (LES) pressure

Impaired LES relaxation on manometry

3. Pseudoachalasia Causes

Adenocarcinoma of the gastric cardia (most common culprit)

Esophageal squamous cell carcinoma

Metastatic tumors infiltrating the myenteric plexus

4. Treatment Implications The treatments for achalasia are very different from cancer treatment:

Achalasia treatments (pneumatic dilation, Heller myotomy, POEM, Botox) would be inappropriate and potentially harmful in cancer

Missing a cancer diagnosis delays curative treatment

Procedures like myotomy on an undiagnosed tumor could worsen outcomes

5. Red Flags Suggesting Malignancy Suspicion is higher when:

Patient is over 55 years old

Symptom duration is short (less than 6-12 months)

Significant, rapid weight loss

Difficulty passing the endoscope through the GE junction

How It's Ruled Out

Upper endoscopy (EGD) with careful inspection of the GE junction and cardia

Biopsies of suspicious areas

CT scan or endoscopic ultrasound (EUS) if pseudoachalasia is suspected despite a normal endoscopy

This is why endoscopy is considered mandatory in the workup of any suspected achalasia case.

**Model DeepSeek V4 Pro**

Answer text:

Doctors must exclude esophageal cancer before diagnosing achalasia because cancer at the gastro-esophageal junction can perfectly mimic the symptoms, radiological findings, and even the high-resolution manometry pattern of primary achalasia. This condition is called pseudoachalasia (or secondary achalasia), and missing it would delay life-saving cancer treatment while exposing the patient to risky procedures that don't address the malignancy.

Here's why this step is non-negotiable:

1. Cancer can infiltrate the nerves that control the esophagus Achalasia results from degeneration of the myenteric (Auerbach's) plexus, which normally coordinates peristalsis and lower esophageal sphincter (LES) relaxation. An adenocarcinoma of the gastric cardia or distal esophagus, or a squamous cell carcinoma, can directly invade that same nerve network or cause a tight, non-relaxing stricture that produces nearly identical manometry-absent peristalsis and an LES that fails to relax fully when swallowing.

2. The symptoms overlap heavily Both conditions cause progressive dysphagia, regurgitation, chest pain, and weight loss. While achalasia typically develops over years and pseudoachalasia over weeks to months, the timeline alone isn't reliable. Some early cancers are indolent, and an elderly patient might not notice slow progression until it's advanced.

3. Imaging can be misleading A barium swallow may show the classic "bird's beak" taper at the LES in achalasia-but an infiltrative cancer can create an identical contour. Even a CT scan can miss a flat, infiltrating tumor (linitis plastica type) that thickens the wall without a distinct mass.

4. The diagnostic gold standard for achalasia isn't enough by itself High-resolution manometry defines achalasia by demonstrating failed peristalsis and impaired LES relaxation. But it cannot see the cause-whether neurodegeneration or a microscopic tumor infiltrating the plexus. Therefore, every patient with suspected achalasia must have an upper endoscopy with careful inspection of the cardia and biopsies. In achalasia, the mucosa looks smooth, and the scope passes through the tight LES with a "pop." In pseudoachalasia, there may be a subtle mucosal irregularity, ulcer, or resistance at the cardia; biopsies (including deep or endoscopic ultrasound-guided sampling) are taken to rule out malignancy.

5. The consequences of misdiagnosis are severe If pseudoachalasia is mistaken for true achalasia, a patient might undergo pneumatic dilation or surgical myotomy-treatments that won't stop the cancer. Worse, those interventions can mask symptoms temporarily while the tumor grows, spread, and become incurable. Conversely, a prompt cancer diagnosis leads to appropriate staging, chemotherapy, radiation, or resection.

Bottom line: The standard workup for dysphagia always begins with an upper endoscopy to exclude cancer, even before manometry. Achalasia is a diagnosis of exclusion only after cancer and other structural mimics have been ruled out.

A related follow-up: Would you like to know what subtle clues on endoscopy or EUS (endoscopic ultrasound) help differentiate pseudoachalasia from true achalasia when no obvious mass is seen?

**Model Gemini 3.1 Pro Thinking**

Answer text:

Doctors must strictly rule out esophageal cancer before diagnosing achalasia because a malignant tumor can perfectly mimic the symptoms and clinical test results of achalasia-a phenomenon known in medicine as "pseudoachalasia."

Here is a detailed breakdown of why this distinction is so critical:

1. Overlapping Symptoms

Both achalasia (a rare nerve disorder where the esophagus loses its ability to squeeze food down and the lower valve fails to relax) and esophageal cancer present with nearly identical warning signs:

Progressive dysphagia (difficulty swallowing liquids and solids).

Regurgitation of undigested food.

Significant, unintended weight loss.

Chest pain or discomfort after eating.

2. The Trap of "Pseudoachalasia"

Cancer occurring at the gastroesophageal (GE) junction (where the esophagus meets the stomach) can cause pseudoachalasia in two ways:

Mechanical Obstruction: A tumor can physically narrow the passageway, acting just like a tight, non-relaxing esophageal sphincter.

Nerve Infiltration: Malignant cells can invade and destroy the myenteric plexus-the exact same nerve network that degenerates in primary achalasia. When this happens, even esophageal manometry (the gold-standard motility test for achalasia) might show results that are indistinguishable from primary achalasia.

3. Severe Consequences of Misdiagnosis

Treating cancer as if it were achalasia can lead to catastrophic outcomes:

Delayed Cancer Treatment: Esophageal cancer is aggressive. The time lost treating a patient for achalasia allows the cancer to grow and spread, potentially turning a treatable tumor into a terminal one.

Dangerous Interventions: Standard treatments for achalasia include pneumatic dilation (using a balloon to tear the sphincter muscle) or a Heller myotomy (surgically cutting the muscle). Performing these procedures on a malignant tumor heavily increases the risk of esophageal perforation (a life-threatening tear) and can potentially seed cancer cells into the surrounding tissue.

How Doctors Differentiate the Two

To ensure they are dealing with primary achalasia and not a hidden tumor, gastroenterologists will typically perform an Upper Endoscopy (EGD). This allows them to visually inspect the mucosal lining of the esophagus and stomach and take biopsies of any suspicious tissue. In some cases, an Endoscopic Ultrasound (EUS) or a CT scan is also used to look at the deeper layers of the esophageal wall and surrounding lymph nodes.

Potential Risk Warning & Health Disclaimer: The information provided here is for educational purposes only and should not be used as a substitute for professional medical advice, diagnosis, or treatment. Esophageal symptoms like difficulty swallowing or weight loss are "red flag" symptoms. If you or someone you know is experiencing these, it is critical to consult a gastroenterologist or primary care physician immediately for a comprehensive evaluation.

Would you like to know more about how diagnostic tests, like esophageal manometry or upper endoscopies, are specifically performed to confirm these conditions?

**Model Grok-4.3**

Answer text:

Doctors must rule out esophageal cancer (specifically pseudoachalasia) before diagnosing primary (idiopathic) achalasia because the two conditions can look nearly identical on initial presentation and testing, but their treatments and prognoses are completely different.

Why the symptoms and tests overlap

Both achalasia and esophageal cancer (particularly tumors at the gastroesophageal junction) cause progressive dysphagia to solids and liquids, regurgitation of undigested food, chest pain, weight loss, and sometimes heartburn.

On barium swallow, both can show the classic "bird-beak" tapering of the lower esophagus.

High-resolution manometry can show absent peristalsis and impaired lower esophageal sphincter relaxation (the Chicago Classification criteria for achalasia) in both conditions.

Because manometry and barium studies cannot reliably distinguish between them, a structural lesion must be actively excluded.

Pseudoachalasia

This term describes a secondary achalasia-like syndrome caused by malignancy. The tumor (most commonly adenocarcinoma of the cardia or distal esophagus, but also lymphoma, metastatic cancer, or even benign tumors) infiltrates the myenteric plexus or mechanically obstructs the junction. It produces the same motility pattern as idiopathic achalasia on manometry.

Key red-flag features that raise suspicion for pseudoachalasia:

Age greater than 50-55 years

Short duration of symptoms ( less than 6-12 months)

Rapid progression

Significant weight loss ( greater than 10-15 lbs / greater than 5-7 kg)

In these patients the risk that the "achalasia" is actually paraneoplastic or infiltrative is substantially higher.

Diagnostic workflow (standard of care)

Guidelines from the American College of Gastroenterology and international motility societies recommend:

Upper endoscopy (EGD) with biopsies - mandatory in essentially all new cases to visualize and sample the gastroesophageal junction and cardia. A normal-appearing cardia does not completely rule out malignancy; some tumors are submucosal.

If suspicion remains high (red flags above or abnormal endoscopy), further testing includes:

Endoscopic ultrasound (EUS) with fine-needle aspiration

CT or PET-CT

Sometimes repeat endoscopy after a period of time

Only after a thorough negative work-up for mechanical obstruction or malignancy is a diagnosis of idiopathic achalasia considered secure.

Risk of missing the cancer

If cancer is mistakenly treated as achalasia (e.g., with pneumatic dilation, Heller myotomy, or peroral endoscopic myotomy), the cancer is left untreated. This can lead to:

Delayed diagnosis by months

Disease progression to incurable stages

Worsened prognosis (esophageal cancer has poor survival once advanced)

Additionally, patients with long-standing achalasia do carry a modestly increased lifetime risk of squamous cell esophageal cancer due to chronic food stasis and inflammation, but that is a separate, much slower process.

Bottom line: Ruling out malignancy is a safety step required to avoid treating cancer with the wrong tools. Endoscopy is quick, low-risk, and highly effective at preventing this diagnostic error.

Would you like a clear comparison of the diagnostic tests (manometry vs. endoscopy vs. EUS), or information on current treatment options once cancer has been safely excluded?

**Model Qwen3-Max**

Answer text:

Doctors need to rule out esophageal cancer before diagnosing achalasia because the two conditions can present with very similar symptoms, and esophageal cancer can sometimes mimic achalasia-a phenomenon known as "pseudoachalasia."

Key Reasons:

Overlapping Symptoms: Both achalasia and esophageal cancer commonly cause:

Dysphagia (difficulty swallowing), often for both solids and liquids

Regurgitation of undigested food

Weight loss

Chest pain or discomfort

Pseudoachalasia: In some cases, a tumor (most commonly at the gastroesophageal junction or in the distal esophagus) can infiltrate or compress the nerves of the esophagus, leading to impaired lower esophageal sphincter (LES) relaxation and absent peristalsis-mimicking the manometric findings of true achalasia. This is called pseudoachalasia.

Diagnostic Similarities on Testing:

High-resolution manometry (HRM), the gold standard for diagnosing achalasia, may show classic features (e.g., absent peristalsis, failed LES relaxation) in both true achalasia and pseudoachalasia.

Barium swallow may show a "bird's beak" tapering in both conditions.

Critical Implications for Treatment:

Achalasia is managed with pneumatic dilation, Heller myotomy, or POEM (peroral endoscopic myotomy)-all of which involve disrupting the LES.

If an underlying malignancy is misdiagnosed as achalasia and these interventions are performed, it could delay cancer treatment and potentially worsen outcomes.

How Do Doctors Rule Out Cancer?

Upper Endoscopy: To visually inspect for tumors, strictures, or mucosal abnormalities. Biopsies are taken if suspicious lesions are seen (though pseudoachalasia may not show obvious mucosal changes).

Endoscopic Ultrasound (EUS): Helps detect submucosal or extrinsic masses not visible on standard endoscopy.

CT Scan or PET-CT: Used if there's high suspicion of malignancy (e.g., rapid symptom onset, significant weight loss, age greater than 50).

Clinical Red Flags: Rapid progression, age over 50, significant weight loss, or lack of typical achalasia history should raise suspicion for pseudoachalasia.

Summary:

Because esophageal cancer can masquerade as achalasia, and because the treatments are vastly different, it is essential to exclude malignancy-especially in atypical or high-risk cases-before confirming a diagnosis of primary achalasia.

Would you like to know more about how pseudoachalasia is distinguished from true achalasia in clinical practice?

**Q19. Can a normal gastroscopy rule out achalasia?**

**Model ChatGPT 5.5**

Answer text:

No. A normal gastroscopy/upper endoscopy cannot reliably rule out achalasia.

Gastroscopy is important because it can look for other causes of swallowing problems-such as cancer, strictures, rings, severe esophagitis, or "pseudoachalasia"-but achalasia is primarily a motility disorder, so the lining can look normal, especially early on. Guidelines describe endoscopy, barium esophagram, and esophageal manometry as complementary tests; achalasia is confirmed with high-resolution esophageal manometry, which is considered the diagnostic standard. (sysge.org)

If symptoms fit-especially difficulty swallowing both solids and liquids, regurgitation of undigested food/liquid, chest pressure, unexplained weight loss, or "reflux" that doesn't respond to acid suppressants-then further testing is usually appropriate even if the gastroscopy was normal. A timed barium swallow/esophagram can show classic signs such as esophageal dilation or "bird-beak" narrowing, but manometry is the key test for confirming the diagnosis and subtype. (europepmc.org)

Bottom line: a normal gastroscopy can make a tumor/stricture less likely, but it does not exclude achalasia. If your symptoms are suggestive, ask your gastroenterologist about high-resolution esophageal manometry and possibly a timed barium swallow.

Seek prompt medical review if you have progressive swallowing difficulty, weight loss, vomiting/regurgitation at night, aspiration/coughing, anemia, or new symptoms after age about 50, because those raise concern for obstruction or pseudoachalasia.

Would you like to share what symptoms led to the gastroscopy-solids only vs solids and liquids, regurgitation, weight loss, reflux treatment response?

**Model Claude Opus 4.7**

Answer text:

Can a Normal Gastroscopy Rule Out Achalasia?

No, a normal gastroscopy (upper endoscopy) cannot reliably rule out achalasia.

Why Endoscopy Has Limitations for Achalasia

Achalasia is a motility disorder caused by failure of the lower esophageal sphincter (LES) to relax and loss of peristalsis. Endoscopy primarily assesses structural/mucosal abnormalities, not motor function.

In early or mild achalasia, endoscopic findings are often normal because:

The esophagus may not yet be dilated

There may be no retained food or saliva

The mucosa typically looks normal

The LES may still allow scope passage with mild resistance

Studies suggest endoscopy misses up to 30-40 percent of achalasia cases, particularly early-stage disease.

What Endoscopy Can Suggest

Findings that raise suspicion include:

Dilated esophagus with retained food/saliva

"Tight" or puckered LES requiring some pressure to traverse

Secondary changes (candidiasis, stasis esophagitis)

But the main role of endoscopy is to exclude pseudoachalasia (e.g., malignancy at the GE junction) rather than to confirm or rule out achalasia.

Gold Standard for Diagnosis

High-resolution esophageal manometry (HRM) is the definitive test. It identifies:

Failed LES relaxation (elevated integrated relaxation pressure)

Absent peristalsis

Subtype classification (I, II, III) per the Chicago Classification

A timed barium esophagram is also helpful, showing delayed emptying and the classic "bird's beak" appearance.

Bottom Line

If clinical suspicion for achalasia is high (progressive dysphagia to both solids and liquids, regurgitation, weight loss), a normal gastroscopy should prompt further workup with manometry and/or barium swallow rather than reassurance.

**Model DeepSeek V4 Pro**

Answer text:

No, a normal gastroscopy cannot reliably rule out achalasia - especially in its early stages. Here's why, when to be cautious, and what to do next.

Why a normal gastroscopy can miss achalasia

Achalasia is a motility disorder of the esophagus, not a mucosal or structural lesion. The core problem is:

Impaired relaxation of the lower esophageal sphincter (LES)

Absent normal peristalsis in the esophageal body

Gastroscopy (EGD) is designed to inspect the mucosal lining and detect masses, strictures, or inflammation. In early achalasia, the esophagus may not yet be dilated, and the LES may offer no significant resistance to the scope. Therefore the exam can appear completely normal.

What an EGD can find in more advanced achalasia

Later in the disease, achalasia may produce visible findings, but even these are not always present:

Dilated, atonic esophagus (sometimes with retained food/fluid)

Tight LES with a "pop" sensation when the scope passes through

Absence of any mechanical obstruction or tumor

The crucial point: a normal-appearing esophagus and cardia during EGD does not equal normal motility.

The real role of gastroscopy in suspected achalasia

The primary value of a normal EGD is to exclude pseudoachalasia - a mechanical obstruction (most commonly a tumor at the gastroesophageal junction) that mimics true achalasia. So, paradoxically, a normal EGD increases the suspicion for primary achalasia when symptoms are suggestive.

How achalasia is actually diagnosed

Definitive diagnosis requires an esophageal motility test, specifically high-resolution esophageal manometry (HRM). A barium swallow study can also show classic findings like a smooth "bird's beak" narrowing at the LES and a dilated esophagus with poor emptying.

Risk of relying on a normal gastroscopy

Delaying manometry can lead to progressive esophageal dilation (megaesophagus), aspiration, weight loss, and a much more difficult treatment course. The optimized approach is: if clinical symptoms point to achalasia (e.g., dysphagia to both solids and liquids, regurgitation, retrosternal pain), proceed to manometry even after a normal EGD.

Have you or someone you know been experiencing difficulty swallowing both solids and liquids? If so, what other tests (like a barium swallow or manometry) have you discussed with your doctor? I'm here to help you understand the next steps.

**Model Gemini 3.1 Pro Thinking**

Answer text:

Disclaimer: I am an AI, not a doctor. The following information is for educational purposes and should not replace professional medical advice. If you are experiencing swallowing difficulties, please consult a gastroenterologist or a qualified healthcare provider.

The short answer is no, a completely normal gastroscopy (upper endoscopy) cannot rule out achalasia.

Here is a breakdown of why this is the case, the role of a gastroscopy, and how achalasia is actually diagnosed:

1. Why a Normal Gastroscopy Doesn't Rule Out Achalasia

Achalasia is a motility (movement) disorder of the esophagus, characterized by the absence of normal swallowing contractions and the failure of the lower esophageal sphincter (LES) to relax.

Structure vs. Function: A gastroscopy is a visual test. It is fantastic for seeing structural issues like ulcers, tumors, strictures, or inflammation. However, it cannot evaluate how well the esophageal muscles are functioning or measuring pressures.

The "Push-Through" Effect: Even though the LES does not relax properly in achalasia, a gastroenterologist can often easily push the endoscope through the tight sphincter, especially because the patient is usually sedated.

Early Stages: In the early stages of achalasia, the esophagus may look visually completely normal. Only in advanced stages might the doctor see a dilated esophagus or retained food/saliva.

2. Why Perform a Gastroscopy at All?

If it can't diagnose achalasia, why do doctors order it? A gastroscopy is a crucial step to rule out other serious conditions, specifically "pseudoachalasia." Tumors at the junction of the esophagus and stomach, severe strictures, or conditions like eosinophilic esophagitis (EoE) can cause symptoms that perfectly mimic achalasia. The endoscopy ensures there is no mechanical blockage or cancer causing the swallowing issues.

3. How is Achalasia Actually Diagnosed?

If you have symptoms like dysphagia (difficulty swallowing), regurgitation, or chest pain, and your gastroscopy is normal, your doctor should consider the following tests:

High-Resolution Esophageal Manometry (HREM): This is the gold standard for diagnosing achalasia. A thin tube containing pressure sensors is passed through the nose and into the stomach. As you swallow small sips of water, the machine measures the muscle contractions of your esophagus and the relaxation of your LES.

Barium Swallow (Esophagram): You drink a chalky liquid while X-rays are taken in real-time. In achalasia, this often reveals a dilated esophagus that tapers down to a point at the stomach, known as the classic "bird's beak" appearance.

Optimization/Next Step Suggestion: If a patient has ongoing swallowing issues (especially with both solids and liquids) and a normal gastroscopy, it is highly recommended to advocate for motility testing (manometry) to avoid a delayed diagnosis of a functional disorder.

To help me provide more tailored information, could you share if you are asking this because you (or someone you know) are still experiencing difficulty swallowing despite a clear endoscopy? Has a doctor mentioned ordering a manometry or barium swallow?

**Model Grok-4.3**

Answer text:

No, a normal gastroscopy (upper endoscopy) cannot reliably rule out achalasia.

Why gastroscopy is often normal or non-diagnostic in achalasia

In early or well-compensated achalasia, the esophagus may look completely normal on endoscopy.

The lower esophageal sphincter (LES) usually appears closed, but this is not a reliable diagnostic sign. Many patients with achalasia have a normal-appearing gastroesophageal junction.

Classic endoscopic findings (dilated esophagus, retained food/saliva, tight LES that doesn't open well with air insufflation, or "pop" when passing the scope through the LES) are only present in more advanced cases. Their absence does not exclude the disease.

Studies show that endoscopy has relatively low sensitivity for achalasia (often missing 30-50 percent of cases, especially type II and early type I).

What actually rules achalasia in or out

The gold-standard tests are:

High-Resolution Manometry (HRM) - This is the definitive test. It directly measures the impaired relaxation of the LES and the loss of normal esophageal peristalsis. Chicago Classification v4.0 is used to diagnose and subtype achalasia (Types I, II, III).

Barium esophagram (timed barium swallow) - Often very helpful. It can show the classic "bird-beak" appearance, delayed emptying, and esophageal dilation even when endoscopy looks normal.

Endoscopy is still important - mainly to exclude pseudoachalasia (e.g., cancer at the cardia mimicking achalasia) and to look for complications (esophagitis, candidiasis, stasis, etc.).

Bottom line

A completely normal gastroscopy report does not rule out achalasia. If you have suggestive symptoms (dysphagia to solids and liquids, regurgitation of undigested food, chest pain, weight loss, or long-standing heartburn that doesn't respond to PPIs), you should proceed to esophageal manometry even if your endoscopy was normal.

Next step / question for you: What symptoms are you experiencing, and how long have they been going on? This will help clarify whether manometry or a barium swallow should be the next test.

**Model Qwen3-Max**

Answer text:

A normal gastroscopy (upper endoscopy)cannot reliably rule out achalasia.

Why?

Achalasia is a motility disorder of the esophagus characterized by:

Failure of the lower esophageal sphincter (LES) to relax during swallowing

Absence of normal peristalsis in the esophageal body

These are functional abnormalities, not structural ones. In early or even classic achalasia, the esophageal lining may appear entirely normal during endoscopy. The endoscopist might notice:

A dilated esophagus with retained food or fluid

Resistance when passing the scope through the LES ("pop" sign)

Pseudodiverticula or mild mucosal inflammation from stasis

However, none of these findings are always present, and their absence doesn't exclude achalasia.

Gold Standard for Diagnosis

The definitive test for achalasia is high-resolution esophageal manometry (HRM), which shows:

Absent peristalsis in the esophageal body

Impaired LES relaxation (e.g., elevated integrated relaxation pressure, or IRP)

Additional supportive tests:

Barium esophagogram: May show "bird's beak" tapering, esophageal dilation, and poor emptying

Endoscopy: Primarily used to exclude mechanical obstruction (e.g., tumor, stricture) or other conditions like eosinophilic esophagitis

Clinical Takeaway

If achalasia is suspected based on symptoms (e.g., dysphagia to solids and liquids, regurgitation, chest pain, weight loss), a normal endoscopy should not stop the workup. Proceed to manometry and/or barium swallow.

Would you like guidance on interpreting manometry findings or differentiating achalasia from other esophageal motility disorders?

**Q20. How is achalasia different from gastroesophageal reflux disease?**

**Model ChatGPT 5.5**

Answer text:

Achalasia and GERD can feel similar-both can cause regurgitation, chest discomfort, and "heartburn-like" symptoms-but they are very different disorders.

Feature Achalasia GERD

Main problem The esophagus doesn't move food down normally, and the lower esophageal sphincter often fails to relax, so food/liquid backs up. Stomach acid/content refluxes upward because the anti-reflux barrier is weak or opens inappropriately.

Typical key symptom Difficulty swallowing-often both solids and liquids, usually progressive. Heartburn and sour/acid regurgitation, often after meals or lying down.

Regurgitation Often undigested food or saliva, sometimes hours after eating; may happen at night. Usually acidic/sour fluid or stomach contents.

Response to acid reducers Often poor or incomplete; may be mistaken for "refractory GERD." Often improves with lifestyle changes, antacids, H2 blockers, or proton-pump inhibitors.

Weight loss More concerning/common, especially if swallowing worsens. Less typical unless severe or due to food avoidance.

Diagnosis Best confirmed with esophageal manometry; barium swallow and endoscopy often help. Often diagnosed clinically; may use endoscopy or pH monitoring if unclear or persistent.

Treatment focus Open/relax the lower esophageal sphincter: pneumatic dilation, Heller myotomy, POEM, botulinum toxin in selected cases. Reduce reflux and acid exposure: lifestyle changes, acid-suppressing meds, sometimes anti-reflux surgery.

Achalasia is an esophageal motility disorder characterized by abnormal/absent peristalsis and insufficient relaxation of the lower esophageal sphincter, while GERD is primarily a reflux disorder involving movement of gastric contents back into the esophagus. (sysge.org)

A practical clue: trouble swallowing both liquids and solids, regurgitation of undigested food, nighttime coughing/choking, or weight loss points more toward achalasia than typical GERD. Merck notes that nocturnal regurgitation of undigested food can occur in achalasia and may lead to cough or aspiration. (merckmanuals.com)

Another clue: if someone has been treated for GERD but symptoms persist-especially with progressive swallowing difficulty-achalasia should be considered. The ACG achalasia guideline specifically notes that incorrect GERD diagnosis can delay achalasia diagnosis. (sysge.org)

Seek medical evaluation promptly if you have progressive swallowing trouble, food sticking, vomiting/regurgitation at night, unexplained weight loss, anemia, black stools, or chest pain-especially because other conditions can mimic both GERD and achalasia.

Would you like a symptom-by-symptom checklist to help decide when achalasia should be suspected instead of GERD?

**Model Claude Opus 4.7**

Answer text:

Achalasia vs. Gastroesophageal Reflux Disease (GERD)

While both conditions affect the esophagus and can cause overlapping symptoms, they are fundamentally different disorders.

Achalasia

What it is: A rare motility disorder where the lower esophageal sphincter (LES) fails to relax, and the esophagus loses its ability to move food downward (peristalsis).

Key features:

Cause: Degeneration of nerves in the esophagus (often idiopathic/autoimmune)

LES pressure: Abnormally high and won't open properly

Main symptoms:

Difficulty swallowing (dysphagia) for both solids and liquids

Regurgitation of undigested food

Chest pain, weight loss

Nocturnal coughing/aspiration

Diagnosis: Esophageal manometry (gold standard), barium swallow showing "bird-beak" appearance

Treatment: Pneumatic dilation, Heller myotomy, POEM (peroral endoscopic myotomy), or Botox injection

GERD

What it is: A common condition where stomach contents flow back up into the esophagus due to a weak or inappropriately relaxing LES.

Key features:

Cause: Weak LES, hiatal hernia, obesity, dietary factors

LES pressure: Abnormally low or relaxes too often

Main symptoms:

Heartburn (burning chest pain after meals)

Acid regurgitation (sour/bitter taste)

Worse when lying down or bending over

Chronic cough, hoarseness

Diagnosis: Clinical symptoms, endoscopy, pH monitoring

Treatment: Lifestyle changes, antacids, H2 blockers, PPIs, or fundoplication surgery

Quick Comparison

Feature Achalasia GERD

LES function Won't relax (tight) Too relaxed (weak)

Direction of problem Food can't go down Acid comes up

Dysphagia Prominent, for solids AND liquids Mild, mostly solids if present

Heartburn Uncommon Hallmark symptom

Treatment with PPIs Ineffective Effective

Important note: Achalasia is sometimes misdiagnosed as GERD initially because regurgitation can mimic acid reflux. Persistent dysphagia or symptoms unresponsive to PPIs should prompt further evaluation.

**Model DeepSeek V4 Pro**

Answer text:

Achalasia and gastroesophageal reflux disease (GERD) are both esophageal disorders, but they are nearly opposites in their underlying mechanisms, symptoms, and management. Here's a breakdown of the key differences.

1. Core mechanism (what goes wrong)

Achalasia is a motility disorder.

The lower esophageal sphincter (LES) fails to relax properly due to degeneration of inhibitory neurons in the esophageal wall.

The esophageal body also loses peristalsis (coordinated squeezing). Food and liquid can't pass easily into the stomach - they accumulate, causing gradual esophageal dilation.

GERD is a reflux disorder.

The LES is too weak or relaxes inappropriately, allowing stomach acid and contents to flow back (reflux) into the esophagus.

Peristalsis is usually normal, but chronic acid exposure can damage the lining (esophagitis, Barrett's esophagus).

2. Cardinal symptoms

Achalasia GERD

Progressive dysphagia (difficulty swallowing) to both solids and liquids from early on. Heartburn (retrosternal burning), often after meals or when lying down.

Regurgitation of undigested, non-acidic food, often hours after meals. Can lead to nocturnal cough or aspiration. Sour/bitter-tasting acid regurgitation; may be worse at night.

Chest pain or discomfort (sometimes mimicking heart pain). Chest pain can occur, but it's typically a burning sensation.

Weight loss (unintentional) due to difficulty eating. Weight gain is possible; no direct restriction of intake.

No heartburn initially, though some patients describe a sensation of "chest fullness." Heartburn is a hallmark (though not all GERD patients have it).

Symptoms are relentlessly progressive if untreated. Symptoms wax and wane, often linked to triggers.

3. Diagnostic findings

Barium swallow X-ray:

Achalasia: classic "bird's beak" narrowing at the LES with a dilated, tortuous esophagus above; delayed emptying of barium.

GERD: may show free reflux of barium, hiatal hernia, or complications like stricture; esophageal motility is normal.

High-resolution esophageal manometry (gold standard for achalasia):

Achalasia: elevated integrated relaxation pressure (IRP), absent peristalsis, and often panesophageal pressurization.

GERD: manometry is typically normal, or may show a hypotensive LES and ineffective motility, but these alone don't diagnose GERD.

Ambulatory pH monitoring:

Achalasia: generally normal acid exposure (unless complicated by stasis or superimposed reflux).

GERD: abnormal distal esophageal acid exposure time ( percent time pH less than 4) is the defining feature.

4. Treatment approaches (diametrically opposite)

Achalasia aims to disrupt the tight LES to allow passage:

Pneumatic balloon dilation

Heller myotomy (surgical cutting of the LES muscle)

Peroral endoscopic myotomy (POEM)

Botulinum toxin injection (temporary, often for frail patients)

Pharmacologic relaxants (nitrates, calcium channel blockers) are rarely effective long term.

GERD aims to reinforce a weak LES and reduce acid:

Lifestyle changes (elevate head of bed, avoid triggers)

Proton pump inhibitors (PPIs), H2 blockers

Fundoplication (surgical wrap) to augment the LES barrier

Risk warning: Achalasia treatments that lower the LES can sometimes lead to iatrogenic GERD (post-myotomy reflux), requiring long-term acid suppression or even fundoplication at the time of surgery. Chronic achalasia also carries a small risk of esophageal cancer (squamous cell carcinoma) due to stasis and irritation, while chronic GERD is linked to adenocarcinoma (Barrett's esophagus).

5. Can they be confused?

Yes, early stages can overlap. Some achalasia patients describe "heartburn" from food stasis and fermentation (not acid). Others develop reflux after treatment. Conversely, severe GERD with stricture can cause dysphagia. This is why objective testing (manometry, pH monitoring, endoscopy) is crucial when the diagnosis isn't clear.

A follow-up question for you: Are you asking out of personal curiosity, studying for a medical exam, or trying to understand a specific patient scenario? Knowing that would let me tailor the next step - for instance, a comparison table for differentials, a deeper dive into manometry patterns, or guidance on when to suspect one over the other.

**Model Gemini 3.1 Pro Thinking**

Answer text:

While both achalasia and gastroesophageal reflux disease (GERD) affect the esophagus (the tube connecting your throat to your stomach) and share some overlapping symptoms, they are fundamentally opposite mechanical problems.

To put it simply: Achalasia is a problem of food not being able to go down, while GERD is a problem of stomach contents coming back up.

Here is a detailed breakdown of how they differ:

1. The Core Mechanism: The "Door" to the Stomach

Both conditions revolve around the Lower Esophageal Sphincter (LES), a muscular ring at the bottom of the esophagus that acts like a valve or a door.

Achalasia: The LES fails to relax (the door stays tightly shut) when you swallow. Additionally, the esophagus loses its normal muscle contractions (peristalsis) needed to push food down. Food and liquid back up in the esophagus.

GERD: The LES is unusually weak or relaxes when it shouldn't (the door swings open too easily). This allows stomach acid and partially digested food to flow backward into the esophagus.

2. Primary Symptoms

Because the mechanics are different, the nature of the symptoms differs significantly:

Achalasia Symptoms:

Dysphagia: Difficulty swallowing both liquids and solids (feels like food is stuck in the chest).

Regurgitation: Bringing up undigested, bland-tasting food or liquid that never made it to the stomach.

Weight loss (due to inability to eat normally).

GERD Symptoms:

Heartburn: A burning sensation in the chest caused by stomach acid irritating the esophageal lining.

Regurgitation: Bringing up sour, bitter, or acidic stomach contents.

Chronic cough or hoarseness (especially in the morning).

3. Differences in Treatment

Because the conditions are essentially mechanical opposites, their treatments aim for opposite results:

Achalasia Treatment: The goal is to weaken or open the overly tight LES. This is done through procedures like balloon dilation (stretching the valve), Botox injections (paralyzing the muscle to relax it), or surgery (Heller myotomy or POEM) to cut the muscle fibers of the LES.

GERD Treatment: The goal is to strengthen or tighten the LES and reduce stomach acid. This is achieved through medication (antacids, PPIs), lifestyle changes, or in severe cases, anti-reflux surgery (like fundoplication) to wrap the top of the stomach around the LES to reinforce it.

Potential Risk Warnings & Optimization Suggestions

Diagnostic Risk (Misdiagnosis): Achalasia is a rare disease, and in its early stages, the chest pain and regurgitation it causes are very frequently misdiagnosed as GERD.

The Danger: Taking acid-reducing medications (like PPIs) will not cure achalasia. If achalasia is left untreated for years, the esophagus can stretch massively (megaesophagus), leading to a complete loss of function that may eventually require the surgical removal of the esophagus.

When to see a doctor: If you have been diagnosed with GERD but do not respond to standard antacid or PPI medications, or if you consistently feel like food is physically getting stuck in your chest, you must seek a re-evaluation from a gastroenterologist. A test called esophageal manometry is the gold standard for definitively distinguishing between the two.

Lifestyle & Management Optimizations:

If you suspect GERD: Optimize your routine by avoiding eating within 3 hours of bedtime, elevating the head of your bed by 6-8 inches, and identifying dietary triggers (like caffeine, alcohol, spicy foods, and chocolate).

If you suspect Achalasia: While awaiting medical treatment, optimize swallowing by chewing food into a near-liquid consistency, eating very slowly, and drinking plenty of warm liquids with meals to help wash food down via gravity.

Disclaimer: I am an AI, not a doctor. If you are experiencing difficulty swallowing, chest pain, or chronic regurgitation, please consult a healthcare professional for an accurate diagnosis.

To help me provide more tailored information, could you share what specific symptoms you (or the person you are asking for) are experiencing, or are you just researching the differences out of general curiosity?

**Model Grok-4.3**

Answer text:

Achalasia and GERD (Gastroesophageal Reflux Disease) are two distinct esophageal disorders that can cause overlapping symptoms like difficulty swallowing and regurgitation, but they have fundamentally opposite problems with the lower esophageal sphincter (LES) and esophageal motility.

Key Differences

Feature Achalasia GERD

Primary Problem Failure of the LES to relax + loss of esophageal peristalsis (propulsive contractions) Excessive or inappropriate relaxation of the LES, allowing stomach acid to reflux

Motility Absent or severely impaired peristalsis (the esophagus doesn't "push" food down) Peristalsis is usually normal

LES Pressure Typically high resting pressure with incomplete relaxation Normal or low resting pressure

Main Symptoms Progressive dysphagia to both solids and liquids from the beginning, regurgitation of undigested food (often hours later), chest pain, weight loss, nocturnal choking Heartburn (burning sensation), acid regurgitation, sour taste, worse after meals or lying down. Dysphagia usually occurs only later if complications (stricture) develop

Underlying Cause Degeneration of inhibitory neurons in the esophageal myenteric plexus (often autoimmune or idiopathic) Weak anti-reflux barrier (hiatal hernia, obesity, transient LES relaxations, diet, medications)

Risk Profile High risk of food retention, aspiration pneumonia, esophageal dilation (megaesophagus), and increased risk of esophageal squamous cell cancer Risk of erosive esophagitis, Barrett's esophagus, and esophageal adenocarcinoma

Diagnostic Findings - High-resolution manometry: absent peristalsis + impaired LES relaxation (Chicago Classification)- Barium swallow: "bird-beak" appearance- Endoscopy often normal early on - Endoscopy may show erosions or Barrett's- 24-hour pH-impedance monitoring shows abnormal acid exposure- Manometry usually normal

Treatment Goal Disrupt or weaken the non-relaxing LES (pneumatic dilation, Heller myotomy, POEM procedure, Botox) Strengthen the anti-reflux barrier and reduce acid (PPIs, lifestyle changes, fundoplication surgery)

Why They Are Sometimes Confused

Both can cause chest pain and regurgitation, and some patients with long-standing achalasia develop secondary reflux after treatment (because the LES has been intentionally weakened). There is also a rare condition called pseudoachalasia (usually due to malignancy) that can mimic achalasia but behaves differently.

Important Clinical Note

The treatments are essentially opposite: weakening the LES helps achalasia but would worsen GERD. This is why accurate diagnosis-particularly with high-resolution esophageal manometry-is critical before any invasive procedure.

Would you like me to explain the diagnostic testing process (especially manometry), typical treatment outcomes, or how doctors differentiate the two when symptoms overlap?

**Model Qwen3-Max**

Answer text:

Achalasia and gastroesophageal reflux disease (GERD) are both disorders of the esophagus, but they differ significantly in their underlying mechanisms, symptoms, diagnostic findings, and treatments.

1. Pathophysiology

Achalasia: A motility disorder caused by degeneration of the myenteric plexus (nerve cells in the esophageal wall), leading to:

Failure of the lower esophageal sphincter (LES) to relax during swallowing.

Absent or ineffective peristalsis in the esophageal body.

Result: Food/liquid cannot pass efficiently into the stomach, causing esophageal dilation over time.

GERD: A mechanical and functional disorder where the LES is too relaxed or incompetent, allowing stomach contents (including acid) to reflux back into the esophagus.

Often associated with hiatal hernia, obesity, or transient LES relaxations.

Leads to mucosal irritation/inflammation (esophagitis).

2. Symptoms

Symptom Achalasia GERD

Dysphagia Progressive, for solids AND liquids Usually for solids; may occur with liquids if stricture present

Regurgitation Undigested food, often without acid taste, worse when lying down Acidic/bitter taste, especially after meals or at night

Heartburn Uncommon or atypical Classic symptom

Chest pain Possible (due to esophageal spasm) Common (from acid irritation)

Weight loss Common (due to chronic dysphagia) Rare (unless severe complications)

Aspiration/pneumonia Possible (from retained food) Possible (from acid reflux)

Key distinction: In achalasia, dysphagia affects both solids and liquids from early on; in GERD, dysphagia (if present) usually develops later and is more solid-predominant.

3. Diagnostic Tests

High-resolution manometry (HRM):

Achalasia: Shows absent peristalsis + incomplete LES relaxation (e.g., elevated integrated relaxation pressure [IRP]).

GERD: Typically normal motility; may show hypotensive LES.

Barium swallow:

Achalasia: "Bird's beak" tapering at LES, dilated esophagus, retained barium.

GERD: May show reflux of contrast, hiatal hernia, but normal esophageal emptying.

Endoscopy:

Achalasia: May show retained food, dilated esophagus; no mucosal inflammation (unless secondary).

GERD: May reveal esophagitis, erosions, Barrett's esophagus.

pH monitoring:

GERD: Confirms abnormal acid exposure.

Achalasia: Not typically used; acid exposure is usually low.

4. Treatment

Achalasia:

Goal: Reduce LES pressure to allow food passage.

Options: Pneumatic dilation, Heller myotomy (surgical), POEM (peroral endoscopic myotomy), or botulinum toxin injection.

Proton pump inhibitors (PPIs) are NOT effective.

GERD:

Goal: Reduce acid production/reflux.

Options: Lifestyle changes, PPIs, H2 blockers; surgery (e.g., Nissen fundoplication) for refractory cases.

Caution: Misdiagnosing achalasia as GERD and treating with PPIs can delay proper care and lead to worsening malnutrition or aspiration.

Summary Table

Feature Achalasia GERD

LES function Fails to relax Too loose / inappropriate relaxation

Peristalsis Absent Normal

Primary symptom Dysphagia (solids + liquids) Heartburn, regurgitation

Response to PPIs None Often improves

Manometry Diagnostic gold standard Usually normal

If you're evaluating a patient with dysphagia, high-resolution manometry is critical to distinguish between these conditions-especially before starting long-term acid suppression therapy.

Would you like a comparison of how these conditions appear on imaging or endoscopy?

**Q21. How is achalasia different from esophageal cancer?**

**Model ChatGPT 5.5**

Answer text:

Achalasia and esophageal cancer can feel similar because both can cause trouble swallowing, but they are very different conditions.

Feature Achalasia Esophageal cancer

What it is A motility/nerve disorder: the esophagus doesn't push food down normally, and the lower esophageal sphincter doesn't relax well. A malignant tumor growing in the esophagus.

Cause of blockage feeling Functional narrowing/spasm at the lower esophageal sphincter; no tumor is required. Physical narrowing from a cancerous mass or tumor infiltration.

Typical swallowing pattern Often difficulty swallowing both solids and liquids from early on. Often starts with solids first, then progresses to liquids as narrowing worsens.

Progression Usually slow, over months to years. Often progressive and may worsen more quickly.

Common symptoms Regurgitation of undigested food, chest discomfort, coughing/choking at night, weight loss can occur. Progressive dysphagia, weight loss, chest/back pain, hoarseness, vomiting, bleeding/black stools in some cases.

Diagnosis Endoscopy to rule out cancer, then esophageal manometry is the key test; barium swallow may show "bird-beak" narrowing. Upper endoscopy with biopsy is required to confirm cancer; CT/PET/endoscopic ultrasound may stage it.

Treatment Pneumatic dilation, Heller myotomy, POEM procedure, botulinum toxin in selected patients; not treated with chemo/radiation. Depends on stage: surgery, chemotherapy, radiation, immunotherapy/targeted therapy, stents, palliative care.

Relationship between them Achalasia is not cancer, but long-standing achalasia can increase risk of esophageal cancer. Cancer can sometimes mimic achalasia, called pseudoachalasia.

Achalasia is a disorder of esophageal movement caused by nerve dysfunction, leading to impaired peristalsis and poor relaxation of the lower esophageal sphincter. Esophageal cancer begins in the lining cells of the esophagus and may form a tumor that narrows the swallowing passage. (mayoclinic.org)

A key clinical clue is that achalasia commonly causes trouble with both liquids and solids, while cancer more classically starts with solids and later affects liquids-but this is not reliable enough to diagnose without testing. Because symptoms overlap, guidelines and expert sources emphasize endoscopy to exclude malignancy when someone has dysphagia. (bestpractice.bmj.com)

Red flags that should be evaluated promptly include new or worsening trouble swallowing, unintentional weight loss, vomiting, blood in stool or vomit, anemia, persistent chest pain, hoarseness, or symptoms starting later in life. Esophageal cancer diagnosis typically requires endoscopy and biopsy. (cancer.org)

Bottom line: achalasia is usually a benign motility disorder; esophageal cancer is a malignant growth. But because cancer can mimic achalasia-and achalasia can increase long-term cancer risk-persistent swallowing problems should be evaluated by a gastroenterologist.

Have you or someone else been diagnosed with achalasia already, or are you trying to understand symptoms like trouble swallowing?

**Model Claude Opus 4.7**

Answer text:

Achalasia vs. Esophageal Cancer

While both conditions can cause difficulty swallowing (dysphagia), they are fundamentally different diseases.

Achalasia

A benign motility disorder of the esophagus.

Cause: Loss of nerve cells in the esophagus, causing the lower esophageal sphincter (LES) to fail to relax and the esophagus to lose peristalsis

Nature: Functional problem - no tumor or growth

Onset: Gradual, often over years

Dysphagia pattern: Difficulty with both solids AND liquids from the start

Other symptoms: Regurgitation of undigested food, chest pain, heartburn, slow weight loss

Age: Typically 25-60 years

Prognosis: Manageable; not life-threatening with treatment

Esophageal Cancer

A malignant tumor of the esophagus (squamous cell carcinoma or adenocarcinoma).

Cause: Abnormal cell growth, often linked to smoking, alcohol, GERD/Barrett's esophagus, obesity

Nature: Structural obstruction from a tumor

Onset: Rapidly progressive (weeks to months)

Dysphagia pattern: Starts with solids, then progresses to liquids

Other symptoms: Rapid weight loss, anemia, bleeding, hoarseness, persistent cough

Age: Typically greater than 55 years

Prognosis: Often poor, especially if diagnosed late

Key Diagnostic Differences

Feature Achalasia Esophageal Cancer

Barium swallow "Bird's beak" tapering at LES; dilated esophagus Irregular, asymmetric narrowing ("apple core")

Endoscopy Normal mucosa; LES tight but passable Visible mass, ulceration, or stricture

Manometry Absent peristalsis, failure of LES relaxation Usually normal motility

Biopsy Normal Malignant cells confirm diagnosis

Important Overlap

Pseudoachalasia: Tumors at the gastroesophageal junction can mimic achalasia, so cancer must be ruled out - especially in older patients with rapid symptom onset and weight loss.

Long-standing achalasia slightly increases the risk of developing squamous cell carcinoma of the esophagus.

Bottom line: Achalasia is a nerve/muscle problem causing functional obstruction, while esophageal cancer is a structural obstruction from a malignant tumor. Endoscopy with biopsy is essential to distinguish them.

**Model DeepSeek V4 Pro**

Answer text:

Here is the comparison in English, keeping the same level of detail and structure:

Achalasia vs. Esophageal Cancer: Key Differences

While both conditions can cause dysphagia (difficulty swallowing), they are fundamentally different in nature, diagnosis, and treatment. Below is a side-by-side comparison.

Aspect Achalasia Esophageal Cancer

Nature Benign, functional motility disorder Malignant tumor

Cause Failure of the lower esophageal sphincter (LES) to relax and loss of esophageal peristalsis; often linked to degeneration of inhibitory neurons in the esophageal myenteric plexus. Exact trigger unknown. Malignant transformation of cells, linked to smoking, heavy alcohol use, chronic reflux (Barrett's esophagus to adenocarcinoma), hot beverages, nitrosamines, HPV infection, etc.

Dysphagia Pattern Difficulty with both liquids and solids from early on; paradoxically, liquids may be more difficult initially. Patients often need to drink water, arch their back, or raise their arms to help food pass. Typically starts with solids and progresses to liquids; steadily worsening dysphagia.

Other Symptoms Regurgitation of undigested food (especially at night), chest pain, heartburn, slow weight loss, halitosis, nocturnal cough/aspiration. Rapid, unintentional weight loss, odynophagia (painful swallowing), hoarseness (recurrent laryngeal nerve involvement), black/tarry stools (melena) or hematemesis, palpable supraclavicular lymph node (Virchow's node).

Gold Standard Diagnostic Test High-Resolution Manometry (HRM) - shows impaired LES relaxation and absent peristalsis. Upper Endoscopy + Biopsy - direct visualization and histopathological confirmation of malignant cells.

Radiological Features (Barium Swallow) Dilated esophagus with a smooth, symmetrical "bird's beak" narrowing at the gastro-esophageal junction; lack of peristaltic waves. Irregular/filling defect or asymmetric rigid stricture; mucosal destruction. CT/PET-CT can show mass and metastases.

Endoscopic Appearance Dilated esophagus with intact, normal-appearing mucosa; tight LES that opens with gentle pressure. Fungating, ulcerated, or infiltrative mass; friable, easily bleeding tissue with loss of normal mucosal pattern.

Treatment No cure; aims to reduce LES pressure. Options: pneumatic balloon dilation, laparoscopic Heller myotomy, POEM (Per-Oral Endoscopic Myotomy), botulinum toxin injection (temporary). Surgery (esophagectomy), chemotherapy, radiation therapy, targeted therapy, and immunotherapy. Early-stage disease can be potentially curable.

Prognosis Not directly life-threatening, but complications include recurrent aspiration pneumonia and severe esophagitis. Risk of esophageal squamous cell carcinoma is elevated (up to 10-50 x ) in long-standing achalasia ( greater than 10-15 years). Depends heavily on staging at diagnosis; late-stage esophageal cancer has a poor prognosis with low 5-year survival.

Critical Risk Note

Patients with achalasia do have an increased risk of developing esophageal squamous cell carcinoma, especially after 10-15 years of untreated or inadequately treated disease. This does not mean achalasia turns into cancer, but regular endoscopic surveillance is strongly recommended.

If you or someone you know has progressive dysphagia - particularly with unintentional weight loss, black stools, or chest pain - it is vital to seek medical evaluation immediately rather than attributing it to a benign condition. A gastroenterologist will usually start with an upper endoscopy.

Next Step

Would you like me to explain the POEM procedure in more detail, or would you prefer a deeper dive into how high-resolution manometry is performed and what results to expect? Let me know and I'll continue.

**Model Gemini 3.1 Pro Thinking**

Answer text:

Disclaimer: I am an AI, not a doctor. The following information is for educational purposes and should not be used for self-diagnosis. If you or someone you know is experiencing swallowing difficulties, please consult a healthcare professional immediately.

While both achalasia and esophageal cancer primarily affect the esophagus and share common symptoms-most notably dysphagia (difficulty swallowing) and weight loss-they are fundamentally very different diseases.

Here is a breakdown of the key differences between the two:

1. The Nature of the Disease

Achalasia is a benign (non-cancerous) nerve and muscle disorder. It occurs when the nerves in the esophagus become damaged. This causes two problems: the esophageal muscles lose their ability to push food down (peristalsis), and the lower esophageal sphincter (the valve between the esophagus and stomach) fails to relax to let food pass.

Esophageal Cancer is a malignant disease where abnormal cells mutate and grow out of control, forming a tumor in the lining of the esophagus. This tumor physically obstructs the passage of food.

2. The Pattern of Symptoms

How the swallowing difficulty presents is often the biggest clinical clue:

Achalasia: People typically experience difficulty swallowing both solids and liquids at the same time from the onset of the disease. The symptoms can sometimes fluctuate from day to day and are often accompanied by the regurgitation of undigested food, heartburn-like chest pain, and a feeling that food is stuck in the chest.

Esophageal Cancer: The difficulty swallowing is usually progressive. It typically begins with hard, solid foods (like meat or bread). As the tumor grows and narrows the esophagus further, the difficulty progresses to soft foods, and eventually, to liquids. Unintentional, rapid weight loss is very prominent, and patients may also experience vomiting blood or black, tarry stools.

3. The Link Between the Two

It is important to note that having achalasia is a recognized risk factor for developing esophageal cancer later in life. Because food sits in the esophagus and ferments, it causes chronic inflammation. Over many years (often decades), this chronic irritation can cause cellular changes that may lead to cancer.

Potential Risks & Clinical "Best Practices" (Diagnostics)

Because the symptoms overlap, a doctor cannot diagnose either condition based on symptoms alone. Misdiagnosing early-stage esophageal cancer as a benign motility disorder poses a severe risk to patient survival.

To definitively differentiate the two, gastroenterologists rely on a specific diagnostic workflow:

Upper Endoscopy (EGD): This is usually the first step. A camera is used to look inside the esophagus. If there is a tumor (cancer), the doctor will see it and take a biopsy. In achalasia, the esophagus might look dilated and retain food, but there will be no tumor.

Esophageal Manometry: This is the "gold standard" for diagnosing achalasia. A thin tube measures the muscle contractions and pressure in the esophagus to prove the nerves/muscles are misfiring.

Barium Swallow (Esophagram): The patient drinks a contrast liquid while x-rays are taken. Achalasia often shows a classic "bird's beak" appearance at the bottom of the esophagus, whereas cancer will show an irregular stricture (narrowing).

When to seek immediate care (Red Flags): If you experience progressive inability to swallow, unexplained weight loss, black stools, or coughing up blood, you should be evaluated by a physician without delay.

Would you like to know more about the specific treatment options available for achalasia, or are you looking for information on the risk factors that contribute to esophageal cancer?

**Model Grok-4.3**

Answer text:

Achalasia and esophageal cancer are two distinct conditions that can cause similar symptoms like difficulty swallowing (dysphagia) and weight loss, but they differ fundamentally in nature, cause, behavior, and treatment.

Quick Definitions

Achalasia is a rare benign esophageal motility disorder. It occurs when nerves in the esophagus degenerate, causing two main problems: (1) the lower esophageal sphincter (LES) fails to relax properly, and (2) the esophageal body loses its normal wave-like contractions (peristalsis). Food and liquids have trouble entering the stomach and tend to sit in the esophagus.

Esophageal cancer is a malignant tumor that grows in the lining of the esophagus. The two main types are squamous cell carcinoma (often upper/middle esophagus) and adenocarcinoma (usually lower esophagus, often arising from Barrett's esophagus).

Key Differences

Aspect Achalasia Esophageal Cancer

Nature Non-cancerous functional disorder Malignant (cancerous) tumor

Cause Degeneration of inhibitory neurons (often autoimmune or idiopathic; sometimes secondary to Chagas disease) DNA mutations driven by risk factors (smoking, heavy alcohol, chronic GERD, obesity, Barrett's esophagus, HPV in some cases)

Typical Age Usually 25-60 years Usually greater than 55-60 years

Symptom Pattern Dysphagia to both solids and liquids from early on; regurgitation of undigested food/liquid (often at night), chest pain, heartburn-like symptoms Progressive dysphagia (usually solids first, then liquids); odynophagia (painful swallowing), hoarseness, anemia, black stools, rapid weight loss

Onset Usually gradual over months to years Can be more rapid once advanced

Pathophysiology Failure of LES relaxation + aperistalsis Physical mass obstructing and/or invading the esophagus

Risk of Cancer Slightly increased long-term risk ( about 0.1-0.5 percent per year) due to food stasis and chronic irritation It is cancer; can spread (metastasize)

Diagnosis High-resolution manometry (gold standard), barium swallow ("bird-beak" appearance), endoscopy to rule out cancer Endoscopy with biopsy (definitive), CT/PET scans, endoscopic ultrasound

Treatment Pneumatic dilation, Heller myotomy, POEM (peroral endoscopic myotomy), Botox injections, sometimes medications Depends on stage: endoscopic resection, esophagectomy, chemotherapy, radiation, immunotherapy, stenting for palliation

Prognosis Chronic but manageable; quality of life can be good with treatment Poor if advanced (5-year survival less than 20 percent for metastatic disease); much better if caught very early

Important Overlap: Pseudoachalasia

A small but critical point is that cancer at the gastroesophageal junction can mimic achalasia (called pseudoachalasia). This is why doctors usually perform endoscopy (and sometimes endoscopic ultrasound or CT) in anyone newly diagnosed with achalasia, especially if they are:

Over age 50-55

Have rapid onset of symptoms

Have significant unintentional weight loss

Summary

Achalasia is a nerve/muscle coordination problem without a tumor. Esophageal cancer is an actual malignant growth. While both can make swallowing difficult, cancer tends to be more aggressive, occurs in older patients with specific risk factors, and requires completely different treatment.

This is general medical information and not a substitute for professional care. Swallowing problems should always be evaluated by a gastroenterologist.

Would you like me to explain the diagnostic process (especially manometry vs endoscopy), treatment options for achalasia, risk factors for esophageal cancer, or how to differentiate the two on a barium swallow?

**Model Qwen3-Max**

Answer text:

Achalasia and esophageal cancer are two distinct conditions affecting the esophagus, differing significantly in cause, pathology, symptoms, diagnosis, and treatment.

1. Definition & Pathophysiology

Achalasia: A motility disorder caused by degeneration of the myenteric (Auerbach's) plexus in the esophageal wall, leading to:

Failure of the lower esophageal sphincter (LES) to relax during swallowing.

Absent or abnormal peristalsis in the esophageal body. It is not cancerous-it's a functional/neuromuscular disorder.

Esophageal Cancer: A malignant tumor arising from the esophageal lining. Two main types:

Adenocarcinoma (often in the distal esophagus, linked to chronic GERD/Barrett's esophagus).

Squamous cell carcinoma (often in the proximal/mid esophagus, linked to smoking/alcohol). Involves uncontrolled cellular growth that can invade locally or metastasize.

2. Symptoms (Overlap & Differences)

Both can cause:

Dysphagia (difficulty swallowing), initially for solids, then liquids.

Regurgitation of undigested food.

Weight loss (more common/severe in cancer).

Key differences:

Achalasia:

Symptoms develop gradually over years.

Chest pain or discomfort may occur.

No systemic signs like fatigue or anemia (unless malnourished).

Esophageal Cancer:

Symptoms often progress more rapidly (weeks to months).

May present with odynophagia (painful swallowing), hoarseness, cough, or hematemesis (vomiting blood).

Systemic symptoms: significant weight loss, fatigue, anorexia.

3. Diagnostic Findings

Achalasia:

High-resolution manometry: Gold standard-shows absent peristalsis + incomplete LES relaxation.

Barium swallow: "Bird's beak" tapering at LES, dilated esophagus with retained barium.

Endoscopy: Rules out mechanical obstruction; may show retained food but no mass.

Esophageal Cancer:

Endoscopy with biopsy: Reveals a mass, ulceration, or stricture; histology confirms malignancy.

CT/PET scans: Assess local invasion and metastasis.

EUS (Endoscopic ultrasound): Evaluates depth of tumor invasion and lymph nodes.

Important: Achalasia slightly increases the risk of esophageal squamous cell carcinoma (due to chronic stasis and irritation), so long-standing achalasia requires monitoring.

4. Treatment

Achalasia: Aimed at reducing LES pressure:

Pneumatic dilation

Heller myotomy (surgical)

Peroral endoscopic myotomy (POEM)

Botulinum toxin injection (temporary)

Esophageal Cancer: Depends on stage:

Early stage: Endoscopic resection or surgery (esophagectomy)

Locally advanced: Chemoradiation plus or minus surgery

Metastatic: Palliative chemotherapy, immunotherapy, stenting

Summary Table

Feature Achalasia Esophageal Cancer

Nature Benign motility disorder Malignant neoplasm

Onset Gradual (years) Progressive (weeks-months)

Dysphagia Solids to liquids Often starts with solids

Weight loss Mild/moderate Often severe

Manometry Abnormal (diagnostic) Usually normal (unless advanced)

Endoscopy No mass; possible food retention Mass, ulcer, stricture

Biopsy Normal Malignant cells

If someone presents with new-onset dysphagia-especially if older than 50 or with alarm symptoms (weight loss, bleeding)-esophageal cancer must be ruled out first before diagnosing achalasia.

Would you like to know how doctors differentiate them in clinical practice using specific tests?

**Q22. Can achalasia be misdiagnosed as reflux disease?**

**Model ChatGPT 5.5**

Answer text:

Yes. Achalasia can be misdiagnosed as reflux disease/GERD, especially early on, because both can cause heartburn-like discomfort, chest pain, regurgitation, cough, or a sour/bitter taste.

The key difference is that in achalasia, the main problem is usually food and liquid not moving properly through the esophagus because the lower esophageal sphincter doesn't relax normally. In GERD, the main problem is acid refluxing back up from the stomach.

Clues that "reflux" might actually be achalasia include:

Difficulty swallowing - especially if it affects both solids and liquids

Regurgitation of undigested food, particularly hours after eating or when lying down

Symptoms that don't improve with PPIs like omeprazole, pantoprazole, esomeprazole, etc.

Progressive worsening over months/years

Weight loss, avoiding meals, or taking a long time to eat

Nighttime choking/coughing from retained food or fluid

The American College of Gastroenterology guideline notes that achalasia commonly presents with dysphagia, regurgitation, chest pain, and sometimes weight loss, and that an incorrect GERD diagnosis can delay achalasia diagnosis. Diagnosis is typically evaluated with upper endoscopy, barium esophagram, and confirmed with high-resolution esophageal manometry, the gold-standard test. (sysge.org)

This confusion is well recognized in studies: achalasia is "often misdiagnosed as refractory GERD," and patients may have similar reflux-like symptoms even though the underlying disorder is different. (pmc.ncbi.nlm.nih.gov) A 2023 study of 300 achalasia patients found substantial diagnostic delay and reported GERD as a common false diagnosis. (academic.oup.com)

If you or someone you know has been treated for reflux but has trouble swallowing, regurgitates undigested food, or isn't responding to acid suppression, it would be reasonable to ask a gastroenterologist whether testing for achalasia or another esophageal motility disorder is appropriate.

Seek urgent care if there is inability to swallow liquids, dehydration, vomiting blood, black stools, severe chest pain, or significant unintentional weight loss.

Would you like me to help you compare your symptoms with typical GERD vs achalasia warning signs?

**Model Claude Opus 4.7**

Answer text:

Yes, achalasia is frequently misdiagnosed as gastroesophageal reflux disease (GERD), and this is actually one of the most common diagnostic errors associated with the condition. Studies suggest diagnosis is often delayed by several years, with many patients initially treated for GERD before achalasia is identified.

Why the Confusion Happens

Overlapping symptoms:

Both can cause heartburn or burning chest discomfort

Both can cause regurgitation (though the mechanism differs)

Both can cause chest pain

Both can cause difficulty swallowing

Misleading features of achalasia:

Early-stage achalasia may present primarily with heartburn-like symptoms

Fermentation of retained food in the esophagus can produce acidic-tasting material that mimics acid reflux

Patients may initially respond partially to dietary changes or positional measures

Key Distinguishing Features

Features that should raise suspicion for achalasia rather than GERD:

Dysphagia for both solids and liquids (GERD typically causes solid-food dysphagia only if a stricture develops)

Regurgitation of undigested food, sometimes hours after eating or at night

Poor or no response to proton pump inhibitors

Weight loss

Need to use maneuvers (drinking water, raising arms, standing) to help food pass

Why It Matters

Misdiagnosis is problematic because:

PPIs don't treat achalasia

Delayed diagnosis allows progressive esophageal dilation

Long-standing achalasia increases esophageal cancer risk

Confirming the Diagnosis

If achalasia is suspected, the gold standard is high-resolution esophageal manometry, supported by barium swallow (showing the classic "bird's beak" appearance) and endoscopy to rule out other causes (pseudoachalasia from malignancy).

If you or someone you know has had persistent reflux-like symptoms unresponsive to standard treatment, especially with swallowing difficulties or regurgitation, it's worth discussing further evaluation with a gastroenterologist.

**Model DeepSeek V4 Pro**

Answer text:

Yes, absolutely. Achalasia is frequently misdiagnosed as gastroesophageal reflux disease (GERD) - in fact, it's a common pitfall in clinical practice. The two conditions can present with very similar symptoms, but the underlying problem and treatment are completely different.

How the confusion happens

Overlapping symptoms:

Heartburn / chest pain - In GERD, it's caused by acid refluxing into the esophagus. In achalasia, it's often due to stasis of food and liquids, which ferment and produce lactic acid, causing a burning sensation that feels identical to acid reflux.

Regurgitation - In GERD it's typically sour/acidic fluid; in achalasia it's bland, undigested food (often hours after a meal) which patients may describe as "vomiting" or "wet burps."

Dysphagia (difficulty swallowing) - Can occur in both, but in achalasia it's progressive for both solids and liquids from the start, whereas in GERD it's usually a late complication (e.g., from a stricture).

Why misdiagnosis persists:

Many doctors assume all heartburn = acid reflux, especially in younger patients.

A trial of proton pump inhibitors (PPIs) often provides partial relief in achalasia (by reducing the acidity of the stagnant fluid), which reinforces a false diagnosis of GERD.

Non-specialists may not immediately jump to manometry - the gold standard for achalasia - when symptoms initially sound like reflux.

Potential dangers of misdiagnosis

Delay in effective treatment (such as pneumatic dilation, Heller myotomy, or POEM), allowing the esophagus to progressively dilate and lose function.

Unnecessary long-term PPI use with no real benefit.

Increased risk of aspiration pneumonia, weight loss, and in advanced stages, massive esophageal dilation (megaesophagus) which can make definitive treatment less effective.

Key differentiating clues

Feature Typical GERD Achalasia

Difficulty swallowing For solids, later stages; may not be prominent Early, progressive, for solids and liquids

Regurgitation Acidic, shortly after meals Bland, undigested food, often hours later

Weight loss Uncommon unless severe dysphagia Common in untreated achalasia

Response to PPIs Usually good for heartburn Partial or temporary at best

Barium swallow May show reflux, possible stricture "Bird's beak" narrowing at LES, dilated esophagus

Esophageal manometry Normal or weak peristalsis, normal LES relaxation Absent peristalsis, failed LES relaxation (definitive)

Best practice recommendations

If a patient is diagnosed with GERD but their symptoms (especially dysphagia or regurgitation of undigested food) persist despite adequate PPI therapy, the next step should be esophageal manometry, not just an endoscopy. Endoscopy can rule out tumors (pseudoachalasia) but cannot reliably diagnose the motility disorder itself.

Important risk note: Performing an upper endoscopy is safe, but therapeutic dilation of the LES (for a supposed stricture) without knowing the patient has achalasia could theoretically cause perforation if the LES is hypertonic and the diagnosis is missed. Always confirm with manometry first if achalasia is suspected.

Do you have a specific case or symptom timeline in mind? Or would you like me to explain how the diagnostic tests (like high-resolution manometry) actually work to tell the two conditions apart?

**Model Gemini 3.1 Pro Thinking**

Answer text:

Yes, achalasia is very frequently misdiagnosed as Gastroesophageal Reflux Disease (GERD). In fact, GERD is the most common initial misdiagnosis for patients who actually have achalasia. Because GERD is incredibly common and achalasia is quite rare, doctors naturally suspect reflux first.

Here is a breakdown of why this misdiagnosis happens, the risks involved, and how the two are distinguished.

Why They Are Confused (Symptom Overlap)

The symptoms of achalasia and GERD can look strikingly similar on the surface:

Regurgitation: Both conditions cause food and liquid to come back up. In GERD, it comes up from the stomach. In achalasia, the food never made it to the stomach; it gets trapped in the esophagus and comes back up undigested.

Heartburn/Chest Pain: In GERD, stomach acid burns the esophagus. In achalasia, the trapped food ferments and produces lactic acid, which causes a burning sensation that feels exactly like heartburn.

Difficulty Swallowing (Dysphagia): Severe GERD can cause esophageal strictures (narrowing) that make swallowing difficult. Achalasia fundamentally prevents swallowing because the esophageal muscles are paralyzed and the lower valve won't open.

The Core Difference (The Paradox)

Physiologically, the two conditions are exact opposites:

GERD happens because the Lower Esophageal Sphincter (LES)-the valve between the esophagus and stomach-is too loose or relaxes when it shouldn't, letting stomach contents wash upward.

Achalasia happens because the nerve endings in the esophagus degenerate. The LES becomes too tight and fails to relax, preventing food and liquid from passing down into the stomach.

Potential Risks of Misdiagnosis

Ineffective Treatment: Patients misdiagnosed with GERD are usually prescribed Proton Pump Inhibitors (PPIs) like omeprazole. These will do nothing to treat achalasia, prolonging the patient's suffering.

Esophageal Damage: If achalasia goes undiagnosed for years, the esophagus can stretch out from holding trapped food and liquid, eventually becoming a "megaesophagus." At this severe stage, the esophagus may need to be surgically removed.

Aspiration Pneumonia: Trapped food regurgitated at night can easily be inhaled into the lungs, causing severe respiratory infections.

How to Get the Correct Diagnosis

If a patient has "GERD" that does not respond to acid-blocking medications, or if they have progressive difficulty swallowing both solids and liquids, a gastroenterologist will usually order specific tests:

High-Resolution Esophageal Manometry: This is the "gold standard" for diagnosing achalasia. A thin tube is passed through the nose into the esophagus to measure the muscle contractions and valve pressure while the patient swallows water.

Barium Swallow (Esophagram): The patient drinks a chalky liquid while X-rays are taken. In achalasia, the esophagus looks dilated and narrows to a "bird's beak" shape at the bottom.

Upper Endoscopy (EGD): A camera is used to look inside the esophagus to rule out mechanical blockages like tumors or severe strictures.

Disclaimer: I am an AI, not a doctor. If you or someone you know is experiencing persistent swallowing difficulties or regurgitation, it is highly recommended to consult a gastroenterologist for a proper medical evaluation.

Are you or someone you know currently experiencing these types of symptoms, or navigating a situation where standard acid reflux treatments aren't working?

**Model Grok-4.3**

Answer text:

Yes, achalasia is frequently misdiagnosed as gastroesophageal reflux disease (GERD) or reflux disease, sometimes for years.

Why the Misdiagnosis Happens

Achalasia is a rare esophageal motility disorder in which the lower esophageal sphincter (LES) fails to relax properly and the esophagus loses its normal peristalsis. This leads to food and saliva accumulating in the esophagus. Common symptoms include:

Dysphagia (difficulty swallowing solids and liquids)

Regurgitation of undigested food or saliva (often at night)

Chest pain or discomfort

Heartburn-like sensation

Weight loss (in later stages)

Coughing or choking at night

Many of these overlap with GERD symptoms. In particular, the regurgitation and "heartburn" sensation in achalasia are often interpreted as acid reflux. Patients may be treated with proton pump inhibitors (PPIs) for months or even years without improvement.

Key Reasons for Confusion

Regurgitation is misinterpreted as reflux: In achalasia the material coming back up is usually undigested food mixed with saliva, not acid. Patients still describe it as "heartburn" or "reflux."

Some patients have true secondary reflux: Stagnant food in the esophagus can ferment and produce lactic acid or even allow some gastric acid to reflux, creating a mixed picture.

Early or "vigorous" achalasia can show simultaneous contractions on manometry that mimic other motility disorders.

Upper endoscopy can look normal or show mild esophagitis, leading doctors to assume it's GERD.

Barium swallow may be misinterpreted: The classic "bird-beak" appearance may be subtle or missed if the radiologist is not specifically looking for it.

How to Differentiate the Two

The gold-standard test to distinguish them is high-resolution esophageal manometry, which definitively shows absent peristalsis and failure of LES relaxation in achalasia. Other helpful tests include:

Timed barium esophagram (shows poor emptying)

Endoscopy with possible biopsy (to rule out pseudoachalasia from cancer)

pH-impedance monitoring (can show that many "reflux" episodes are actually non-acidic regurgitations)

Studies show that up to 30-50 percent of achalasia patients are initially treated for GERD, and the average delay from symptom onset to correct diagnosis can be 2-5 years (sometimes much longer).

Bottom Line

If someone has persistent swallowing difficulty (especially to both solids and liquids), regurgitation of undigested food, or heartburn that does not respond well to PPIs, achalasia should be seriously considered and manometry should be performed before labeling it as refractory GERD.

Would you like me to explain the key diagnostic tests (manometry, barium swallow, pH monitoring) in more detail, or describe how the main treatments for achalasia (POEM, Heller myotomy, pneumatic dilation) compare?

**Model Qwen3-Max**

Answer text:

Yes, achalasia can be misdiagnosed as gastroesophageal reflux disease (GERD)-especially in its early stages-because the symptoms can overlap and mimic each other.

Why Misdiagnosis Occurs:

Similar Symptoms:

Both conditions can cause dysphagia (difficulty swallowing), regurgitation, and chest pain.

Patients with achalasia may report a sensation of food "sticking" or "coming back up," which can be mistaken for acid reflux.

Atypical Presentation:

Early achalasia may present with vague symptoms like heartburn or nocturnal regurgitation, leading clinicians to initially suspect GERD.

Unlike typical GERD, however, achalasia-related regurgitation often involves undigested food (not acidic stomach contents) and occurs without acid exposure.

Inadequate Initial Testing:

If diagnosis relies only on symptom history or empirical proton pump inhibitor (PPI) trials (common in GERD), achalasia may be overlooked.

PPIs usually do not improve symptoms in true achalasia, but a partial or placebo response can delay correct diagnosis.

Key Differences to Help Distinguish:

Feature Achalasia GERD

Regurgitated material Undigested food, bland taste Acidic, sour/bitter taste

Response to PPIs Minimal or none Often improves

Dysphagia For both solids and liquids (progressive) Usually solids only, intermittent

Weight loss Common (due to chronic dysphagia) Uncommon unless severe

Manometry findings Absent peristalsis + failed LES relaxation Normal motility; low LES pressure possible

Diagnostic Gold Standard:

High-resolution esophageal manometry (HRM) is essential to confirm achalasia (showing aperistalsis and incomplete lower esophageal sphincter [LES] relaxation).

Barium swallow may show a "bird's beak" tapering and esophageal dilation.

Endoscopy is often normal or shows retained food-but rules out mechanical obstruction.

Clinical Tip: If a patient diagnosed with GERD has progressive dysphagia to liquids, no response to PPIs after 8-12 weeks, or unintentional weight loss, consider evaluating for achalasia or other esophageal motility disorders.

Would you like guidance on when to refer a patient for motility testing or how to interpret manometry results?

**Q23. What are the main treatments for achalasia?**

**Model ChatGPT 5.5**

Answer text:

The main treatments for achalasia aim to open or relax the lower esophageal sphincter (LES) so food and liquid can pass into the stomach more easily. They do not cure the underlying nerve problem, but they often relieve swallowing symptoms substantially.

Main treatment options

Pneumatic dilation

An endoscopic procedure where a balloon is inflated across the LES to stretch/disrupt the tight muscle.

Often effective, but may need repeat treatments.

Small risk of esophageal perforation.

Common first-line option for appropriate patients. (mayoclinic.org)

Laparoscopic Heller myotomy - usually with partial fundoplication

Minimally invasive surgery that cuts the LES muscle to reduce the obstruction.

A partial fundoplication is often added to reduce reflux after the muscle is cut.

Durable and well-established, especially for patients fit for surgery. (asge.org)

POEM - peroral endoscopic myotomy

An endoscopic "inside-the-esophagus" myotomy that cuts the tight LES muscle without external incisions.

Increasingly used and can be particularly helpful for certain achalasia subtypes, such as spastic/type III achalasia.

Reflux after POEM is a common consideration, so follow-up and acid suppression may be needed. (asge.org)

Botulinum toxin injection

Botox is injected into the LES during endoscopy to temporarily relax the muscle.

Usually reserved for older or medically fragile patients who are not good candidates for dilation, POEM, or surgery.

Benefit often wears off, so repeat injections may be needed. (gi.org)

Medications

Drugs such as nitrates or calcium-channel blockers can relax the LES somewhat.

They are generally less effective and are usually used only when procedural treatments are not possible or as a temporary measure.

Esophagectomy

Rarely needed.

Considered only in advanced, end-stage achalasia with severe esophageal dilation or failure of other therapies. (gi.org)

How doctors choose among them

Choice depends on factors like:

Achalasia subtype on high-resolution manometry

Age and surgical risk

Symptom severity

Prior treatments

Local expertise

Risk of reflux

Patient preference

For many otherwise healthy patients, the major definitive options are pneumatic dilation, laparoscopic Heller myotomy, or POEM. Guidelines emphasize that these procedures are best done by experienced specialists, ideally at centers that treat achalasia frequently. (mayoclinic.org)

If you want, I can also give you a side-by-side comparison of POEM vs Heller myotomy vs pneumatic dilation.

**Model Claude Opus 4.7**

Answer text:

Main Treatments for Achalasia

Achalasia is a rare esophageal motility disorder where the lower esophageal sphincter (LES) fails to relax properly. Treatment focuses on reducing LES pressure to improve swallowing, as there's no cure that restores normal muscle function.

1. Pneumatic Dilation (PD)

A balloon is inflated at the LES to stretch and tear muscle fibers

Performed endoscopically

Often requires repeat procedures

Risk of esophageal perforation ( about 2 percent )

2. Surgical Myotomy (Heller Myotomy)

Laparoscopic cutting of LES muscle fibers

Usually combined with a partial fundoplication (Dor or Toupet) to prevent reflux

High long-term success rate ( about 90 percent )

Considered gold standard surgical option

3. Peroral Endoscopic Myotomy (POEM)

Newer, minimally invasive endoscopic procedure

Myotomy performed through a submucosal tunnel

Excellent short- and medium-term results

Higher rates of post-procedure GERD

Particularly effective for type III (spastic) achalasia

4. Botulinum Toxin Injection

Injected into the LES via endoscopy

Temporarily relaxes the sphincter

Effects wear off in 6-12 months

Reserved for elderly or poor surgical candidates

5. Pharmacologic Therapy

Calcium channel blockers (e.g., nifedipine) or nitrates (e.g., isosorbide dinitrate)

Taken before meals

Limited efficacy and side effects

Used only when other options aren't feasible

6. Esophagectomy

Reserved for end-stage achalasia (megaesophagus) when other treatments fail

Major surgery with significant morbidity

Choosing Treatment

The choice depends on:

Achalasia subtype (I, II, or III on high-resolution manometry)

Patient age and surgical risk

Local expertise

Patient preference

For most patients, POEM, laparoscopic Heller myotomy, and pneumatic dilation offer comparable effectiveness as first-line therapies. Lifelong follow-up is recommended due to recurrence risk and slightly increased esophageal cancer risk.

**Model DeepSeek V4 Pro**

Answer text:

Achalasia is a rare esophageal motility disorder where the lower esophageal sphincter (LES) fails to relax properly and peristalsis is absent. Treatments aim to relieve the obstruction at the LES, improve swallowing, and prevent complications. The main options fall into three categories: endoscopic, surgical, and pharmacological. I'll outline each with their typical use, risks, and optimization suggestions.

1. Endoscopic treatments

These are minimally invasive and often first-line for many patients.

Pneumatic dilation (PD) A balloon is passed through the mouth and inflated at the LES to forcefully disrupt the muscle fibers.

Effectiveness: about 70-90 percent symptom relief initially, but may need repeat dilations over time.

Risks: Esophageal perforation ( about 2-5 percent of procedures, lower with experienced operators).

Optimization: Using graded, sequential balloon diameters and post-procedure contrast study to rule out leak. Offers a short procedure, no incisions.

Peroral endoscopic myotomy (POEM) An endoscope is inserted through the mouth, and a submucosal tunnel is created to cut the LES muscle from inside. No external incisions.

Effectiveness: greater than 90 percent success at 1-2 years; particularly favored for type III achalasia.

Risks: Mainly post-procedure gastroesophageal reflux disease (GERD) - up to 40-60 percent because the LES is thoroughly cut without an anti-reflux wrap. Also bleeding, mucosal perforation, pneumothorax.

Optimization: Patient selection (obese, high-risk surgical patients, type III). Post-POEM long-term PPI use often needed. Consider a fundoplication-structured closure (transoral incisionless fundoplication) in trial settings to reduce reflux.

Botulinum toxin injection (Botox) Botulinum toxin is injected into the LES to paralyze muscle contraction.

Effectiveness: Quick symptom relief, but effect wears off in 3-12 months. Repeat injections may become less effective and can cause scarring.

Risks: Make subsequent myotomy more difficult due to fibrosis. Generally reserved for elderly or high-surgical-risk patients not candidates for definitive therapy.

Optimization: Use only as a bridge or in palliation. Avoid in younger patients who would otherwise be surgical candidates.

2. Surgical treatment

Laparoscopic Heller myotomy with partial fundoplication The LES muscle is cut via small abdominal incisions, and a partial wrap (Dor or Toupet) is added to minimize reflux.

Effectiveness: about 85-95 percent long-term symptom control; considered the gold standard with durable results.

Risks: Reflux development (10-30 percent even with wrap), perforation ( less than 1 percent ), post-operative dysphagia (if wrap too tight).

Optimization: Intraoperative endoscopy to confirm complete myotomy and check for mucosal integrity. Length of myotomy should extend 2-3 cm onto the gastric cardia. Robotic-assisted approach may enhance precision.

3. Pharmacological therapy

Calcium channel blockers (e.g., nifedipine) or nitrates (isosorbide dinitrate) can reduce LES pressure temporarily.

Use: Only when other therapies are not feasible, or as a short-term bridge.

Risks: Often poorly tolerated (headache, hypotension, edema), incomplete relief, and waning effect.

Optimization: Not recommended as definitive treatment; consider only in palliative settings.

4. Adjunctive and lifestyle measures

Eating smaller, soft meals, thoroughly chewing, and drinking water with meals.

Sleeping with the head elevated if reflux is present.

Regular follow-up (endoscopy, manometry, timed barium swallow) to rule out late complications like esophageal stricture or early carcinoma ( about 1 percent lifetime risk, especially in long-standing untreated achalasia).

Key risk warnings across all modalities:

Early diagnosis and treatment reduce risk of megaesophagus.

Undiagnosed esophageal cancer can mimic pseudoachalasia; always rule out malignancy with endoscopy and biopsy, especially in older patients with rapid symptom onset.

POEM's higher reflux risk demands lifelong acid suppression and surveillance for Barrett's esophagus.

Repeated Botox can complicate future myotomy.

Optimization in decision-making: A multidisciplinary approach (gastroenterologist, surgeon, motility specialist) tailors therapy based on achalasia subtype (Chicago classification), age, surgical risk, and patient's willingness to accept reflux risk.

Would you like a deeper dive into a particular treatment - for example, comparing POEM vs. Heller myotomy in younger patients, or how achalasia subtypes influence the choice?

**Model Gemini 3.1 Pro Thinking**

Answer text:

Achalasia is a rare swallowing disorder in which the esophagus loses its ability to squeeze food down, and the lower esophageal sphincter (LES) fails to relax completely to let food into the stomach. While there is no definitive cure to restore normal esophageal muscle function, treatments are highly effective at relieving symptoms by forcing open or relaxing the LES.

Here are the main treatments for achalasia, ranging from minimally invasive procedures to surgery:

1. Peroral Endoscopic Myotomy (POEM)

How it works: This is an advanced, minimally invasive endoscopic procedure. A gastroenterologist or surgeon inserts an endoscope through the mouth and down the esophagus. They create a small tunnel in the esophageal lining and cut the muscle of the lower esophageal sphincter from the inside.

Pros: No external incisions, fast recovery, and highly effective.

2. Laparoscopic Heller Myotomy

How it works: This is a minimally invasive surgical procedure performed through a few small incisions in the abdomen. The surgeon cuts the muscle at the lower end of the esophagus and the top of the stomach to allow food to pass.

Pros: Long-lasting results. It is often combined with a partial fundoplication (wrapping the top of the stomach around the LES) to prevent acid reflux later on.

3. Pneumatic Dilation

How it works: An endoscope is passed into the stomach while the patient is sedated. A specially designed balloon is positioned at the LES and inflated to forcefully tear the muscle fibers of the sphincter, stretching it open.

Pros: Outpatient procedure, very effective, though nearly a third of patients may need the procedure repeated within 5 years.

4. Botulinum Toxin (Botox) Injections

How it works: Botox is injected directly into the sphincter muscle via an endoscope to paralyze and relax the muscle.

Pros: Extremely safe and quick.

Cons: The effects are temporary (usually lasting 6 to 12 months). It is generally reserved for elderly patients or those who are not healthy enough for surgery or dilation.

5. Medications

How it works: Muscle relaxants such as calcium channel blockers (e.g., nifedipine) or long-acting nitrates can be taken before eating to help relax the lower esophageal sphincter.

Cons: They are the least effective treatment, often cause side effects (like headaches or low blood pressure), and are usually only used as a temporary bridge to definitive treatment.

Potential Risks and Optimization Suggestions

Risk of Acid Reflux (GERD): Because all structural treatments (POEM, Heller Myotomy, Dilation) destroy the barrier that prevents stomach acid from backing up, severe acid reflux is a common side effect.

Optimization: If undergoing a Heller Myotomy, ensure a partial fundoplication (like a Dor or Toupet fundoplication) is performed simultaneously to recreate a physical anti-reflux barrier. For POEM patients, lifelong treatment with Proton Pump Inhibitors (PPIs) and regular endoscopic monitoring is often recommended.

Risk of Esophageal Perforation: Pneumatic dilation carries a small risk (around 1-3 percent ) of tearing a hole in the esophagus, which is a medical emergency requiring immediate surgery.

Optimization: Dilation should be performed by a highly experienced gastroenterologist, and patients should be evaluated carefully; prior treatments (like multiple Botox injections) can cause scarring that makes dilation or myotomy more difficult and riskier.

Disclaimer: I am an AI, not a doctor. Any medical decisions regarding the diagnosis or treatment of achalasia should be made in consultation with a qualified gastroenterologist or thoracic surgeon.

Are you asking this for general knowledge, or are you looking for information to help prepare for an upcoming consultation with a doctor?

**Model Grok-4.3**

Answer text:

The main treatments for achalasia focus on reducing pressure in the lower esophageal sphincter (LES) to improve the passage of food and liquids into the stomach. There is no cure that restores normal esophageal peristalsis, so all therapies are palliative.

1. First-Line / Most Common Treatments

Treatment Type How it works Success rate (symptom relief) Durability Best suited for

Pneumatic Dilation (PD) Endoscopic procedure Balloon dilation tears the LES muscle 70-90 percent initial 1-5 years (often needs repeat) Most patients (especially older)

Heller Myotomy Surgery (usually laparoscopic) Cuts the LES muscle fibers 85-95 percent Excellent (long-term) Younger, fit patients

POEM (Peroral Endoscopic Myotomy) Advanced endoscopic Tunnel created in esophagus to cut LES 90-95 percent Excellent Most patients, especially spastic types

POEM and laparoscopic Heller myotomy are generally considered the most effective long-term options today.

2. Other Treatments

Botulinum toxin (Botox) injection

Injected directly into the LES during endoscopy.

Success rate: about 60-80 percent initially, but effects usually last only 3-6 months.

Mainly used for frail, elderly patients or as a bridge to definitive therapy.

Medications (limited role)

Nitrates (e.g., isosorbide dinitrate) or calcium channel blockers (e.g., nifedipine).

Only modestly effective and often limited by side effects.

Peroral Endoscopic Myotomy (POEM) variants

Standard POEM, Z-POEM, or G-POEM in selected cases.

Esophagectomy (very rare)

Reserved for end-stage disease with a massively dilated, sigmoid-shaped esophagus that no longer functions.

3. Emerging / Investigational

EndoFLIP-guided treatments (to better tailor dilation or myotomy).

Newer endoscopic techniques and stents (still not standard).

Current Treatment Algorithm (2023-2025 guidelines)

Confirm diagnosis with high-resolution manometry, barium swallow, and endoscopy.

For most fit patients: Discuss POEM vs. Laparoscopic Heller Myotomy with partial fundoplication.

For older/frail patients or those who prefer non-surgical options: Pneumatic dilation or Botox.

Type III (spastic) achalasia usually responds best to POEM.

Important considerations:

All definitive treatments (PD, Myotomy, POEM) carry a risk of gastroesophageal reflux afterward, so many patients need long-term proton pump inhibitors.

Treatment choice is highly individualized based on age, achalasia subtype (I, II, or III), esophageal anatomy, and patient preferences.

Would you like me to explain the differences between POEM and Heller myotomy in more detail, or discuss what to expect after treatment (diet, follow-up, success rates by subtype)?

**Model Qwen3-Max**

Answer text:

Achalasia is a rare esophageal motility disorder characterized by the failure of the lower esophageal sphincter (LES) to relax properly during swallowing and the absence of normal peristalsis in the esophageal body. The goal of treatment is to reduce LES pressure and improve esophageal emptying, thereby relieving symptoms like dysphagia, regurgitation, chest pain, and weight loss.

The main treatment options for achalasia include:

1. Pneumatic Dilation (PD)

Description: A balloon is inserted endoscopically into the LES and inflated to disrupt the muscle fibers.

Effectiveness: Successful in about 60-90 percent of patients initially; may require repeat procedures.

Risks: Risk of esophageal perforation ( about 2-5 percent ), which is a surgical emergency.

Best for: Patients who are not candidates for surgery or prefer a non-surgical approach.

2. Heller Myotomy (Surgical Myotomy)

Description: Laparoscopic (or sometimes robotic) division of the LES muscle fibers.

Often combined with a partial fundoplication (e.g., Dor or Toupet) to prevent gastroesophageal reflux.

Effectiveness: greater than 90 percent success rate in experienced centers; durable long-term relief.

Risks: General surgical risks, potential for postoperative GERD if anti-reflux procedure is inadequate.

Best for: Younger, healthy patients seeking long-term solution.

3. Peroral Endoscopic Myotomy (POEM)

Description: A minimally invasive endoscopic technique where a submucosal tunnel is created, and the LES muscle is cut from within.

Effectiveness: Comparable to Heller myotomy ( greater than 90 percent success); especially effective for type III (spastic) achalasia.

Risks: Higher rates of post-procedure GERD (up to 20-40 percent in some studies), though often manageable.

Advantages: No external incisions, shorter recovery, suitable for complex cases.

Best for: Patients with all types of achalasia, particularly those with tortuous esophagus or prior failed treatments.

4. Botulinum Toxin (Botox) Injection

Description: Endoscopic injection of Botox into the LES to inhibit acetylcholine release and relax the sphincter.

Effectiveness: Provides short-term relief (weeks to months); symptom recurrence is common.

Risks: Minimal immediate risk; repeated injections may cause fibrosis and complicate future surgery.

Best for: Elderly or frail patients not suitable for definitive therapy, or as a diagnostic/therapeutic trial.

5. Pharmacologic Therapy (Limited Role)

Includes calcium channel blockers (e.g., nifedipine) or nitrates (e.g., isosorbide dinitrate).

Effectiveness: Modest and temporary; rarely used today due to side effects and poor efficacy.

Role: Generally reserved for patients who cannot undergo other interventions.

Choosing a Treatment

Treatment selection depends on:

Patient age and comorbidities

Achalasia subtype (I, II, or III based on high-resolution manometry)

Local expertise and available resources

Patient preference regarding invasiveness and long-term outcomes

Type II achalasia typically responds best to all interventions, while Type III may benefit more from POEM.

Note: All patients should undergo baseline high-resolution manometry and upper endoscopy before treatment. Long-term follow-up is essential to monitor for complications like reflux or megaesophagus.

Would you like a comparison table of these treatments or guidance on how to choose between POEM and Heller myotomy?

**Q24. Can medicine cure achalasia?**

**Model ChatGPT 5.5**

Answer text:

Medicine usually cannot cure achalasia.

Achalasia is a disorder where the lower esophageal sphincter doesn't relax properly and the esophagus has impaired movement, making it hard for food and liquid to pass into the stomach. Because the underlying nerve dysfunction generally does not reverse, treatment is aimed at relieving the obstruction and improving swallowing, not truly curing the condition.

Medication options-such as nitrates or calcium-channel blockers-may temporarily relax the sphincter, but they are typically less effective, short-acting, and can cause side effects like headache, dizziness, or low blood pressure. They are usually reserved for people who cannot undergo procedures.

More effective treatments include:

Pneumatic dilation: stretching the lower esophageal sphincter with a balloon

Laparoscopic Heller myotomy: surgical cutting of the sphincter muscle, often with an anti-reflux procedure

POEM - peroral endoscopic myotomy: endoscopic cutting of the sphincter muscle

Botulinum toxin injection: can help temporarily, especially in older or high-risk patients, but effects often wear off

These treatments can provide long-term symptom relief, but achalasia often requires ongoing follow-up, because symptoms or reflux can occur later.

If you have trouble swallowing, regurgitation, chest discomfort, weight loss, or food sticking, it's important to see a gastroenterologist for testing such as endoscopy, barium swallow, and esophageal manometry.

A good next step would be to ask your doctor which type of achalasia you have, since treatment choice often depends on manometry findings.

**Model Claude Opus 4.7**

Answer text:

Can Medicine Cure Achalasia?

Short answer: No, medications cannot cure achalasia, but they can sometimes help manage symptoms temporarily.

Why Medications Don't Cure It

Achalasia is caused by permanent damage to the nerves in the esophagus, leading to:

Failure of the lower esophageal sphincter (LES) to relax

Loss of normal esophageal muscle contractions

Once these nerves are damaged, they cannot be regenerated with current medical therapy.

Medications That May Help Symptoms

These are typically used only when other treatments aren't options:

Calcium channel blockers (e.g., nifedipine) - relaxes the LES

Nitrates (e.g., isosorbide dinitrate) - taken before meals

Botulinum toxin (Botox) injections - into the LES, effects last 6-12 months

PDE-5 inhibitors (e.g., sildenafil) - occasionally used off-label

Limitations: These provide only partial, temporary relief and often have side effects (headaches, low blood pressure).

More Effective Treatments

Definitive treatments target the tight sphincter mechanically:

Pneumatic dilation - balloon stretches the LES

Heller myotomy - surgical cutting of LES muscle fibers

POEM (Peroral Endoscopic Myotomy) - endoscopic version of myotomy

These offer long-term symptom relief in most patients, though achalasia is technically managed rather than "cured" - the underlying nerve damage remains.

Bottom Line

There is no medical or surgical cure for achalasia, but procedures like POEM, Heller myotomy, or pneumatic dilation can provide excellent, long-lasting symptom control. A gastroenterologist can help determine the best option for your situation.

**Model DeepSeek V4 Pro**

Answer text:

No, there is currently no medicine that can cure achalasia. Medications can only help temporarily relieve symptoms (mainly difficulty swallowing) by relaxing the lower esophageal sphincter (LES), but they cannot repair the underlying nerve damage, restore normal esophageal peristalsis, or stop disease progression.

Medications sometimes used for symptom relief (not a cure)

Calcium channel blockers (e.g., nifedipine) or nitrates (e.g., isosorbide dinitrate) - Taken before meals to relax the LES. They often become less effective over time and can cause side effects (headaches, low blood pressure, dizziness).

Botulinum toxin (Botox) injection - A local injection into the LES during endoscopy temporarily paralyzes the muscle. Relief typically lasts 6-12 months and repeat injections may be less effective; it also causes scarring that can make later definitive treatments (like myotomy) more difficult.

Other off-label options (e.g., sildenafil) - Sometimes tried but evidence is limited.

Why medicine cannot cure achalasia

Achalasia is caused by progressive loss of nerve cells in the esophageal wall (myenteric plexus). No oral or injectable drug can regenerate these nerves or permanently correct the dysfunctional motility. That's why definitive treatments are procedural, aiming to mechanically disrupt the tight LES:

Pneumatic (balloon) dilation

Heller myotomy (laparoscopic or robotic)

Peroral endoscopic myotomy (POEM)

These are not medicines, but they offer lasting symptom relief for most patients.

Important note: This information is for educational purposes only and does not replace a specialist's assessment. Achalasia is a chronic condition that requires individualized management by a gastroenterologist or surgeon. Medication may still play a role as a temporary bridge, especially for patients who are not candidates for procedures.

Would you like me to explain the differences between these procedural treatments and which might be most appropriate for someone with achalasia?

**Model Gemini 3.1 Pro Thinking**

Answer text:

No, medicine cannot cure achalasia. In fact, there is currently no cure for the underlying cause of achalasia, which is the progressive degeneration of the nerve cells in the esophagus.

Treatments for achalasia are entirely focused on managing the symptoms by relaxing or weakening the lower esophageal sphincter (the valve between the esophagus and stomach) so that food and liquids can pass through more easily.

Here is how medicine and other treatments fit into managing the condition:

The Role of Medication

Medications are generally considered the least effective treatment for achalasia and are usually only prescribed for patients who are not candidates for surgery or endoscopic procedures, or as a temporary bridge until a more permanent procedure can be done.

How they work: Muscle relaxants, such as calcium channel blockers (e.g., nifedipine) or long-acting nitrates (e.g., isosorbide), are taken before meals to help the lower esophageal sphincter relax.

Limitations: They do not work for everyone, their effectiveness tends to wear off over time, and they often cause uncomfortable side effects like severe headaches, dizziness, and low blood pressure.

More Effective Treatment Options

To achieve long-term relief from achalasia symptoms, doctors typically recommend procedural or surgical interventions:

Botox (Botulinum Toxin) Injections: Injected directly into the esophageal sphincter via an endoscope to paralyze the muscle and force it to relax. This is effective but temporary, usually wearing off within 6 to 12 months.

Pneumatic Dilation: A special balloon is inserted through an endoscope and inflated at the sphincter to stretch and tear the muscle fibers, keeping the valve open.

Surgical Myotomy (Heller Myotomy): A minimally invasive surgery where the muscle at the lower end of the esophagus is cut to allow food to pass.

Peroral Endoscopic Myotomy (POEM): A newer, less invasive procedure where the surgeon uses an endoscope inserted through the mouth to cut the esophageal muscle from the inside.

Potential Risks and Medical Disclaimer

This information is for educational purposes and does not constitute medical advice. Relying solely on medications for achalasia when you are a candidate for other procedures can lead to poor nutrition, severe weight loss, and the progressive dilation (stretching) of the esophagus, which can eventually lead to end-stage achalasia (a condition that may require complete removal of the esophagus). If you or a loved one are experiencing symptoms of achalasia, it is crucial to consult with a gastroenterologist or a thoracic surgeon to discuss the safest and most effective treatment plan.

Would you like to know more about the differences in recovery time and success rates between the surgical options like POEM and Heller Myotomy?

**Model Grok-4.3**

Answer text:

No, there is no medicine that can cure achalasia.

What is Achalasia?

Achalasia is a rare esophageal motility disorder in which the lower esophageal sphincter (LES) fails to relax properly and the normal peristalsis (wave-like muscle contractions) of the esophagus is lost. It is caused by the degeneration of inhibitory neurons in the esophageal myenteric plexus. It is generally considered a chronic, progressive neurologic condition, not an infection or simple inflammatory disease.

Current Treatment Landscape (2025)

Treatment Type Can it cure? What it does Typical Duration of Benefit

Medications (nitrates, calcium channel blockers, PDE-5 inhibitors) No Temporarily relaxes the LES Hours to days

Botulinum toxin (Botox) injection No Blocks acetylcholine to reduce LES pressure 3-6 months (often less)

Pneumatic dilation No Mechanically disrupts the LES muscle 1-5+ years (many need repeats)

POEM (Peroral Endoscopic Myotomy) No Cuts the LES muscle from inside Excellent long-term relief in most patients

Heller Myotomy (laparoscopic surgery) No Cuts the LES muscle from outside Very good long-term results

Peroral endoscopic myotomy + fundoplication No Same as POEM but with anti-reflux measure Best current balance of efficacy/side effects

Why Medications Don't Cure It

The underlying problem is loss of inhibitory ganglion cells in the esophagus. No currently available drug can regenerate those neurons.

Medications only provide symptomatic, temporary relief by reducing LES pressure. They are usually used only in patients who are too frail for procedures or while waiting for definitive treatment.

Promising Research Areas (not yet curative)

Stem-cell therapy and neuronal regeneration approaches

Gene therapy targeting the lost inhibitory neurons

Better immunomodulatory treatments (some cases may have autoimmune components)

Improved long-acting LES-relaxing agents

These are still experimental and not available as standard care.

Bottom Line

Definitive treatments are procedural (POEM, Heller myotomy, or repeated dilations), not pharmacologic. Medications can help mild cases or serve as a bridge, but they do not cure achalasia.

Recommendation: See a gastroenterologist who specializes in esophageal motility disorders (ideally at a high-volume center). They will confirm the diagnosis with manometry, barium swallow, and endoscopy, then discuss the best procedural option for your age and overall health.

Follow-up question for you: Have you already had high-resolution manometry and been diagnosed with achalasia, or are you still in the diagnostic phase? This will help me give you more targeted information.

**Model Qwen3-Max**

Answer text:

Achalasia is a chronic esophageal motility disorder characterized by the failure of the lower esophageal sphincter (LES) to relax properly during swallowing and the absence of normal peristalsis in the esophageal body. Unfortunately, there is no medication that can cure achalasia. However, certain medicines may help temporarily relieve symptoms in some patients-especially those who are not candidates for more definitive treatments.

Medications Used (Symptomatic Relief Only):

Calcium channel blockers (e.g., nifedipine):

May reduce LES pressure by relaxing smooth muscle.

Often used short-term or in elderly/frail patients.

Nitrates (e.g., isosorbide dinitrate):

Also act to lower LES pressure.

Limited by side effects like headaches and tolerance development.

Why Medications Aren't Curative:

Achalasia involves irreversible loss of inhibitory neurons (particularly nitric oxide-producing neurons) in the myenteric plexus of the esophagus.

Drugs cannot restore these lost neurons or reestablish normal esophageal motility.

Definitive Treatments (More Effective):

Pneumatic dilation: Stretches and disrupts the LES muscle fibers.

Heller myotomy (surgical or laparoscopic): Cuts the LES muscle to allow easier passage of food.

Peroral endoscopic myotomy (POEM): A minimally invasive endoscopic technique with high success rates.

Botulinum toxin injection: Temporarily paralyzes the LES (effects last weeks to months); often used as a bridge or in high-risk patients.

Important: Untreated achalasia can lead to complications like megaesophagus, aspiration pneumonia, or significant weight loss. Early intervention with definitive therapy is generally recommended.

If you or someone you know has achalasia, consultation with a gastroenterologist specializing in motility disorders is essential to determine the best treatment path.

Would you like a comparison of the success rates or risks of the different procedural options?

**Q25. Which treatment is best for achalasia?**

**Model ChatGPT 5.5**

Answer text:

There isn't one "best" treatment for all achalasia-choice depends mainly on achalasia subtype, your age/health, prior treatments, reflux risk, and local expertise.

In general:

Type III achalasia:POEM - peroral endoscopic myotomy - is often preferred because it can treat the longer spastic segment of the esophagus better. Recent SAGES guidance favors POEM over Heller myotomy with fundoplication for subtype III. (sages.org)

Type I or Type II achalasia:POEM, laparoscopic Heller myotomy with fundoplication, and pneumatic dilation are all effective options. Current guidance supports shared decision-making based on patient factors and center expertise. (sages.org)

POEM vs Heller myotomy: Both are highly effective. POEM is less invasive and especially useful for type III, but it has a higher risk of post-procedure acid reflux and may require long-term acid-suppression medication. Heller myotomy with fundoplication includes an anti-reflux procedure, so reflux risk may be lower. (sages.org)

Pneumatic dilation: A good non-surgical option, especially for some type I/II patients, but it may require repeat treatments and may be less durable than myotomy in some patients. SAGES conditionally recommends POEM over pneumatic dilation in adults, while noting PD can still be appropriate in selected cases. (sages.org)

Botox injection: Usually reserved for people who are poor candidates for surgery/endoscopic myotomy, because its benefit is often temporary. The ASGE guideline lists botulinum toxin as an option but generally alongside more definitive therapies like POEM, pneumatic dilation, and Heller myotomy. (asge.org)

Bottom line: If you are fit for a procedure, the "best" treatment is often POEM for type III, and POEM or Heller myotomy with fundoplication for type I/II. Pneumatic dilation is also reasonable in selected type I/II cases. You should ideally be evaluated at a center that offers all three-POEM, Heller myotomy, and pneumatic dilation-so the recommendation isn't biased by what is available.

If you know your manometry result-type I, II, or III-I can help compare the options more specifically.

**Model Claude Opus 4.7**

Answer text:

Treatment for Achalasia

There is no single "best" treatment for achalasia - the optimal choice depends on the patient's age, overall health, achalasia subtype (I, II, or III), and local expertise. However, here's an evidence-based overview:

First-Line Treatments (Most Effective)

1. Laparoscopic Heller Myotomy (LHM) with partial fundoplication

Long-standing gold standard

Success rate: about 85-90 percent at 5 years

Adds fundoplication (Dor or Toupet) to prevent reflux

Best for: younger patients, type I and II achalasia

2. Peroral Endoscopic Myotomy (POEM)

Newer, incisionless endoscopic procedure

Comparable efficacy to LHM ( about 90 percent short-term success)

Treatment of choice for type III (spastic) achalasia

Higher rate of post-procedure GERD (no anti-reflux wrap)

3. Pneumatic Balloon Dilation (PD)

Graded dilation (30 to 35 to 40 mm balloon)

Effective, less invasive, but often requires repeat sessions

Best for: type II achalasia, older patients

Risk of perforation ( about 2 percent )

Comparable Efficacy

Major trials (e.g., European Achalasia Trial) show LHM, POEM, and pneumatic dilation have similar long-term outcomes when performed at experienced centers.

Second-Line / Alternative Treatments

Botulinum toxin injection - for elderly or high surgical risk patients; effect wears off in 6-12 months

Pharmacologic therapy (nitrates, calcium channel blockers) - limited efficacy; for those unfit for procedures

Practical Recommendations by Subtype

Type Preferred Treatment

Type I LHM or POEM

Type II LHM, POEM, or pneumatic dilation (all effective)

Type III POEM (allows longer myotomy)

Bottom Line

For most patients: LHM with fundoplication or POEM

For type III achalasia: POEM

For poor surgical candidates: botulinum toxin or pneumatic dilation

Treatment is palliative - it relieves symptoms but does not cure the underlying motility disorder. Lifelong follow-up is recommended.

Would you like more detail on a specific treatment or subtype?

**Model DeepSeek V4 Pro**

Answer text:

For achalasia, there is no single "best" treatment for everyone - the choice depends on the subtype (based on high-resolution manometry), surgical risk, age, and personal preference. However, here is a summary of the most effective options according to current guidelines (e.g., ACG 2020):

Main treatment options

Treatment Mechanism Efficacy Risks / Considerations

Pneumatic dilation Balloon stretches the LES Good short-term; long-term success about 50-70 percent (type II best). Often repeatable. Perforation risk (1-3 percent ), post-procedural reflux in some. Not ideal for type III.

Laparoscopic Heller myotomy (LHM) + partial fundoplication Surgical cutting of LES muscle; wrap reduces reflux Excellent long-term ( greater than 80 percent at 5-10 years), especially type I/II. Invasive, requires general anesthesia, recovery time. Very low perforation risk in experienced hands. Reflux if no fundoplication.

Peroral endoscopic myotomy (POEM) Endoscopic myotomy without external incisions Very high success across all subtypes; superior for type III (spastic). Short hospital stay. High GERD incidence (20-50 percent by pH study) - no fundoplication. May require long-term PPI. Rarely, bleeding or pneumothorax.

Botulinum toxin injection Paralyzes LES Rapid relief but temporary (6-12 months); efficacy wanes. Reserved for patients not candidates for more definitive treatments. Scarring may complicate future myotomy.

Pharmacologic (nifedipine, nitrates) Smooth muscle relaxants Weak, short-term, not recommended as sole therapy. Only for temporary symptom control.

What does "best" mean?

For most healthy patients with type I or II achalasia, either LHM with fundoplication or pneumatic dilation is considered first-line; POEM is a reasonable option, especially if the patient accepts long-term GERD surveillance.

For type III (spastic) achalasia, POEM is often preferred because it allows a longer myotomy tailored to the spastic segment.

For elderly/frail patients, botulinum toxin or pneumatic dilation may be safer than surgery, even if less durable.

Risk awareness & optimization

Perforation is a serious complication of pneumatic dilation - always ensure an experienced operator and immediate surgical backup.

GERD after POEM is common and can lead to Barrett's esophagus in a minority (though actual progression risk appears low). Regular upper endoscopy is recommended for those with objective reflux.

Multidisciplinary evaluation (gastroenterologist + foregut surgeon) yields the highest success and lowest complication rates.

No treatment restores normal esophageal peristalsis; symptom relief is the primary goal. Monitoring for recurrence (e.g., timed barium esophagram or routine symptom scores) is important.

Have you had a high-resolution manometry to determine your achalasia subtype (I, II, or III)? That's the key factor guiding which approach would be optimal for you.

**Model Gemini 3.1 Pro Thinking**

Answer text:

Disclaimer: I am an AI, not a doctor. The following information is for educational purposes and should not replace professional medical advice. Always consult with a gastroenterologist or a thoracic surgeon to determine the best treatment plan for your specific case.

There is no single "best" treatment for achalasia. Because achalasia cannot be cured, the goal of all treatments is to relax or open the lower esophageal sphincter (LES) so food and liquid can pass into the stomach.

The most appropriate treatment depends on several factors, including the patient's age, overall health, surgical risk, personal preference, and the specific subtype of achalasia (Types I, II, or III), which is usually determined by a test called high-resolution manometry.

Here is a breakdown of the primary treatment options, along with their potential risks

**Model Grok-4.3**

Answer text:

There is no single "best" treatment for achalasia - the optimal choice depends on your age, overall health, esophageal anatomy (especially sigmoid or dilated esophagus), symptom severity, and personal preferences. Current guidelines (AGA 2020, ASGE, and European guidelines) consider the following as first-line options:

Current Best Treatments (Ranked by Efficacy and Durability)

Treatment Long-term Success Rate Durability Invasiveness Best For Main Drawbacks

Peroral Endoscopic Myotomy (POEM) 85-95 percent Excellent (5-10+ yrs) Minimally invasive (endoscopic) Most patients, especially younger, Type III, or failed prior treatments Higher risk of reflux (30-50 percent ), needs expertise

Laparoscopic Heller Myotomy (LHM) + fundoplication 85-90 percent Excellent Surgical Patients who prefer surgery or have hiatal hernia More invasive, longer recovery

Pneumatic Dilation (PD) 70-90 percent (often needs repeat sessions) Moderate Endoscopic (outpatient) Older patients, frail, or those wanting least invasive option 30-50 percent need repeat procedures, about 2-5 percent perforation risk

Botulinum toxin injection 60-80 percent at 6 months Poor (3-6 months) Endoscopic Elderly, very high surgical risk, bridge to definitive therapy Temporary, less effective long-term

2024-2025 Recommendations (Evidence-Based)

Most patients (especially less than 65 and fit): POEM is now often considered the preferred first-line therapy in high-volume centers because it is highly effective, durable, and allows tailored myotomy length (particularly good for Type III achalasia).

Younger patients ( less than 40-50): Many experts still favor Laparoscopic Heller Myotomy with partial fundoplication because it has the lowest long-term reflux rate when done by experienced surgeons.

Older or higher-risk patients: Pneumatic dilation or POEM (if available).

Very frail/elderly: Botox as initial treatment.

Important Modern Considerations

Subtypes matter (Chicago Classification v4.0):

Type II to best prognosis with any treatment

Type III to POEM is clearly superior

All definitive treatments (POEM, surgery, dilation) carry a significant risk of gastroesophageal reflux afterward. You will likely need lifelong proton-pump inhibitors or further anti-reflux procedures.

Treatment should be done at a high-volume center - outcomes are dramatically better with experienced operators.

What should you do next?

See a gastroenterologist who specializes in esophageal motility disorders (ideally at a center that offers all three major options: POEM, Heller myotomy, and pneumatic dilation). They will usually recommend:

Confirm diagnosis with high-resolution manometry, barium esophagram, and endoscopy.

Discuss the pros/cons tailored to your specific situation.

Would you like me to explain the differences between POEM and Heller myotomy in more detail, or tell you what questions you should ask your doctor before choosing a treatment?

**Model Qwen3-Max**

Answer text:

Achalasia is a rare esophageal motility disorder characterized by failure of the lower esophageal sphincter (LES) to relax during swallowing and absence of normal esophageal peristalsis. While there is no cure, several effective treatments aim to reduce LES pressure and improve esophageal emptying. The "best" treatment depends on patient factors such as age, overall health, symptom severity, disease stage, and personal preference.

Main Treatment Options:

Pneumatic Dilation (PD)

A balloon is inserted endoscopically and inflated in the LES to disrupt muscle fibers.

Success rate: about 70-90 percent in experienced centers.

Risk: Esophageal perforation ( about 2-5 percent ).

Often requires repeat procedures over time.

Best for older patients or those who are not surgical candidates.

Heller Myotomy (Surgical Myotomy)

Laparoscopic Heller myotomy (LHM) involves cutting the LES muscle fibers.

Often combined with a partial fundoplication (e.g., Dor or Toupet) to prevent reflux.

Long-term success: about 80-90 percent .

Preferred for younger, healthy patients due to durability.

Requires general anesthesia and surgical expertise.

Peroral Endoscopic Myotomy (POEM)

Minimally invasive endoscopic procedure that creates a myotomy through the mouth.

Comparable efficacy to LHM ( about 85-95 percent success).

Shorter recovery than surgery; no external incisions.

Higher risk of post-procedure gastroesophageal reflux disease (GERD) ( about 20-40 percent ).

Increasingly used as first-line therapy in specialized centers.

Botulinum Toxin (Botox) Injection

Injected endoscopically into the LES to block acetylcholine release.

Provides short-term relief (weeks to months).

Useful for frail, elderly, or high-risk patients.

Not durable; repeated injections lose effectiveness.

Pharmacologic Therapy (e.g., nitrates, calcium channel blockers)

Rarely used today due to limited efficacy and side effects.

May offer temporary symptom relief when other options aren't feasible.

Current Guidelines & Recommendations:

American College of Gastroenterology (ACG) and European guidelines consider pneumatic dilation, laparoscopic Heller myotomy, and POEM as primary interventions.

POEM and LHM are preferred for long-term outcomes in eligible patients.

Shared decision-making is essential-discuss risks (especially GERD after myotomy/POEM) and benefits.

Important: All patients should undergo pre-treatment evaluation with high-resolution manometry and upper endoscopy to confirm diagnosis and rule out mimics (e.g., esophageal cancer).

Would you like a comparison table of these options or guidance on how to choose based on a specific patient profile?

**Q26. Can achalasia be treated without surgery?**

**Model ChatGPT 5.5**

Answer text:

Yes. Achalasia can be treated without traditional surgery, but the best option depends on your achalasia subtype, age, overall health, symptom severity, and local expertise.

Important: Achalasia usually isn't "cured" in the sense of restoring normal esophageal nerve function. Treatments aim to open or relax the lower esophageal sphincter so food and liquid can pass into the stomach more easily. (uptodate.com)

Non-surgical or less-invasive options

Pneumatic balloon dilation

An endoscopic procedure where a balloon is inflated at the lower esophageal sphincter to stretch/disrupt the tight muscle.

Often considered an effective non-surgical treatment, especially for some patients with type I or II achalasia.

It may need repeat treatments and carries a small risk of esophageal perforation. (asge.org)

Botulinum toxin - "Botox" injection

Injected endoscopically into the lower esophageal sphincter to relax it.

Usually reserved for people who are older, frail, or not good candidates for dilation, POEM, or surgery.

Relief is often temporary, and repeat injections are commonly needed. (asge.org)

Medications

Drugs such as nitrates or calcium-channel blockers can relax the sphincter somewhat.

They are generally the least effective option and are usually used only when procedures are not possible or as a short-term bridge.

Side effects can include low blood pressure, headaches, dizziness, and swelling. (pmc.ncbi.nlm.nih.gov)

POEM - peroral endoscopic myotomy

This is done through an endoscope passed through the mouth, so there are no external incisions.

It is not "traditional surgery," but it is still an invasive procedure that cuts the tight muscle from inside the esophagus.

It is often especially useful for type III/spastic achalasia, but it can increase the risk of acid reflux afterward. (ncbi.nlm.nih.gov)

So, do you need surgery?

Not always. Many people can be treated with pneumatic dilation, Botox, medications, or POEM. However, for durable long-term relief, gastroenterologists often compare pneumatic dilation, POEM, and laparoscopic Heller myotomy based on the individual case. Heller myotomy is the classic surgical option; POEM is endoscopic; dilation and Botox are endoscopic non-surgical options. (asge.org)

You should see a gastroenterologist-ideally one who specializes in esophageal motility-because treatment choice often depends on high-resolution manometry results and achalasia subtype.

Seek urgent care if you have inability to swallow liquids, dehydration, significant weight loss, vomiting blood, black stools, chest pain that could be cardiac, fever, or aspiration/choking episodes.

A good next step would be to ask your GI doctor: "What type of achalasia do I have-type I, II, or III-and which non-surgical options are appropriate for me?"

**Model Claude Opus 4.7**

Answer text:

Non-Surgical Treatments for Achalasia

Yes, achalasia can be treated without surgery, though the effectiveness varies by approach. Here are the main non-surgical options:

Pneumatic Dilation

A balloon is inserted via endoscopy and inflated to stretch and tear the lower esophageal sphincter (LES)

Success rate: 50-90 percent , though many patients need repeat procedures

Minimally invasive but carries a small risk of esophageal perforation (1-3 percent )

Botulinum Toxin (Botox) Injection

Injected into the LES during endoscopy to relax the muscle

Provides relief for about 6-12 months

Best suited for older patients or those who can't tolerate other procedures

Requires repeat injections as effects wear off

Medications

Calcium channel blockers (e.g., nifedipine) or nitrates (e.g., isosorbide dinitrate) taken before meals

Generally the least effective option

Often used when other treatments aren't possible

Side effects include headaches and low blood pressure

POEM (Peroral Endoscopic Myotomy)

Technically a procedure rather than traditional surgery - performed entirely through an endoscope

The LES muscle is cut from inside the esophagus

High success rate ( about 90 percent ), comparable to surgical myotomy

Increasingly considered a first-line option

Choosing a Treatment

The best option depends on:

Your age and overall health

Severity and subtype of achalasia

Availability of experienced providers

Personal preference

Pneumatic dilation and POEM generally offer the most durable non-surgical results, while Heller myotomy (surgical) remains the traditional gold standard.

It's important to discuss these options with a gastroenterologist who specializes in esophageal disorders to determine which approach is right for your specific situation.

**Model DeepSeek V4 Pro**

Answer text:

Yes, achalasia can be managed without traditional open surgery, but it's important to understand that non-surgical treatments are usually palliative (symptom-relieving) rather than curative for the underlying problem. The goal is to reduce the pressure at the lower esophageal sphincter (LES) so food and liquid can pass into the stomach. Below are the main non-surgical options, their effectiveness, and potential risks.

1. Pneumatic Dilation (Balloon Dilation)

What it is: A balloon is passed endoscopically to the LES and inflated to forcefully stretch and tear the muscle fibers. This is a non-surgical, endoscopic procedure.

Effectiveness:

Provides excellent symptom relief in 70-85 percent of patients, often lasting several years.

May be repeated if symptoms return.

Risks & Considerations:

Esophageal perforation (about 2-5 percent risk) - requires immediate medical attention and sometimes surgery.

GERD afterward in about 15-35 percent of patients, usually manageable with acid-suppressing medication.

Not recommended for patients with severe cardiac or pulmonary comorbidities who would not tolerate a possible perforation.

2. Botulinum Toxin (Botox) Injection

What it is: Botox is injected directly into the LES via endoscopy, blocking nerve signals that keep the muscle contracted, thereby relaxing it temporarily.

Effectiveness:

Initial relief in 70-90 percent of patients, but effect wanes after 3-12 months.

Repeated injections often become less effective due to antibody formation or fibrosis.

Risks & Considerations:

Temporary solution - best for elderly patients, those unfit for more invasive procedures, or as a bridge to definitive treatment.

Can cause scaring at the LES, potentially making future myotomy (surgical or POEM) more difficult.

Low risk of heartburn or chest pain.

3. Pharmacological Therapy (Oral Medications)

What it is: Calcium channel blockers (e.g., nifedipine) or long-acting nitrates (e.g., isosorbide dinitrate) taken before meals to relax the LES smooth muscle.

Effectiveness:

Modest and short-lived - only 20-50 percent of patients achieve noticeable improvement.

Often used as a temporary measure for mild disease or when other options are contraindicated.

Risks & Considerations:

Side effects: headache, dizziness, hypotension, and peripheral edema - can be intolerable for many.

Not a long-term solution due to declining efficacy and side effects.

Optimization tip: Sublingual nifedipine is sometimes used "on demand" for mild, intermittent dysphagia, but regular monitoring of blood pressure is required.

4. Peroral Endoscopic Myotomy (POEM) - Is It "Surgery"?

POEM is an endoscopic procedure (no external incisions) where a submucosal tunnel is created and the LES muscle is cut internally. While it is invasive, it is not open or laparoscopic surgery and many patients consider it a middle ground. Technically, it's a non-surgical myotomy.

Effectiveness:

Comparable to surgical Heller myotomy, with symptom improvement in greater than 90 percent of patients.

Shorter recovery, less pain, no visible scars.

Risks & Considerations:

GERD is more common after POEM (up to 40 percent ) because no anti-reflux wrap is performed.

Requires specialized expertise; not available everywhere.

May not be suitable for patients with certain esophageal motility disorders.

Key Optimization & Risk Mitigation Points

Tailor treatment to the patient: Age, comorbidities, esophageal diameter, and type of achalasia (I, II, III) influence the choice. For example, type II achalasia responds best to all treatments, while type III often requires myotomy.

Monitor for iatrogenic GERD: Any LES-relaxing treatment can cause reflux. Proton pump inhibitors (PPIs) should be prescribed liberally after dilation, Botox, or POEM.

Avoid repeated Botox in surgical candidates: It can lead to tissue fibrosis, increasing the difficulty of later myotomy.

Early intervention: Delaying definitive treatment can result in massive esophageal dilation (megaesophagus) where non-surgical options become less effective and esophagectomy may be the only option.

Important disclaimer: This information is for educational purposes only and does not replace professional medical advice. Achalasia is a progressive condition; treatment decisions require a thorough evaluation by a gastroenterologist and sometimes a multidisciplinary team.

Would you like me to explain how the type of achalasia (based on manometry) influences which non-surgical treatment is most appropriate?

**Model Gemini 3.1 Pro Thinking**

Answer text:

Yes, achalasia can be treated without traditional surgery. While there is no absolute cure for achalasia, the goal of all treatments is to relax or force open the lower esophageal sphincter (LES) so that food and liquid can empty into the stomach more easily.

Here are the primary non-surgical and minimally invasive treatment options:

1. Minimally Invasive Endoscopic Procedures

While these are performed in a hospital or clinic setting and require sedation, they do not involve external incisions:

Pneumatic Dilation: This is the most common non-surgical treatment. A gastroenterologist passes an endoscope into the esophagus and inflates a specially designed balloon at the level of the LES. This tears the muscle fibers to widen the opening. It is highly effective, though the procedure may need to be repeated over the years if symptoms return.

Botulinum Toxin (Botox) Injections: Botox is injected directly into the esophageal sphincter via an endoscope. This paralyzes the muscle, allowing it to relax and let food pass. It is very safe, but the effects are temporary (usually lasting 6 to 12 months). It is typically recommended for older adults or those who are not good candidates for more invasive procedures.

Peroral Endoscopic Myotomy (POEM): While sometimes classified as an endoscopic surgery, POEM involves no external incisions. A doctor uses an endoscope inserted through the mouth to cut the muscle on the inside of the esophagus. It offers long-term relief comparable to traditional surgery (Heller myotomy) but with a faster recovery time.

2. Medications

Medications are generally the least effective treatment for achalasia and are usually reserved for patients who cannot undergo pneumatic dilation, POEM, or surgery.

Muscle Relaxants: Drugs such as calcium channel blockers (e.g., nifedipine) or long-acting nitrates (e.g., isosorbide dinitrate) can be taken before meals to help relax the lower esophageal sphincter.

3. Lifestyle and Dietary Management

While not a treatment to fix the underlying muscle problem, modifying how you eat can significantly help manage symptoms:

Chewing food very thoroughly and eating slowly.

Drinking plenty of water with meals to help "wash" food down.

Eating smaller, more frequent meals.

Avoiding eating near bedtime to reduce the risk of regurgitation and aspiration.

Potential Risks and Medical Disclaimer

Please note that I am an AI, not a doctor, and this information should not replace professional medical advice.

Pneumatic Dilation Risks: There is a small risk (about 2-5 percent ) of tearing (perforating) the esophagus, which requires emergency surgery to repair.

POEM Risks: The most common long-term side effect of POEM is the development of gastroesophageal reflux disease (GERD), as the sphincter is permanently loosened.

Medication Risks: Muscle relaxants can cause significant side effects, including severe headaches, dizziness, swelling in the feet, and dangerously low blood pressure.

Next Steps: Because the effectiveness of these treatments depends heavily on a patient's age, overall health, and the specific subtype of achalasia they have, it is highly recommended to consult with a gastroenterologist or a motility specialist.

Would you like to know more about how doctors diagnose the specific "type" of achalasia to determine which of these treatments will work best?

**Model Grok-4.3**

Answer text:

Yes, achalasia can be treated without traditional surgery.

Several effective non-surgical or minimally invasive endoscopic options exist. Treatment choice depends on the patient's age, overall health, achalasia type (especially Type II responds best), and symptom severity. Here's a breakdown of the main approaches:

1. Endoscopic Treatments (No external incisions)

Pneumatic Balloon Dilation (Most common first-line non-surgical option) A specially designed balloon is passed through an endoscope and inflated at the lower esophageal sphincter (LES) to partially disrupt the muscle.

Success rate: 70-90 percent after 1-3 sessions.

Duration: Many patients get good relief for years, though some need repeat dilations.

Risks: Esophageal perforation (2-5 percent ), bleeding, and subsequent gastroesophageal reflux (GERD).

Usually performed as an outpatient procedure under sedation.

Peroral Endoscopic Myotomy (POEM) An advanced endoscopic technique where the doctor creates a tunnel in the esophageal lining, cuts the LES muscle from the inside, and then closes the entry point.

Success rate: greater than 90 percent in most studies, with excellent long-term results.

Considered non-surgical by most patients and many physicians because it is done entirely through the mouth with no skin incisions.

Risks: Higher chance of post-procedure reflux compared to some surgical techniques (often managed with proton-pump inhibitors).

Botox (Botulinum Toxin) Injection Injected directly into the LES during endoscopy to temporarily paralyze the muscle.

Best for elderly patients, those with significant comorbidities, or as a bridge to other therapies.

Effect typically lasts 3-6 months (sometimes up to 12).

Drawbacks: Repeated injections become less effective over time and can cause scarring that makes future procedures more difficult.

2. Medications (Least invasive, limited effectiveness)

Calcium channel blockers (e.g., nifedipine) or nitrates (e.g., isosorbide dinitrate) taken before meals.

These provide only partial, short-term relief for mild cases or patients who cannot undergo procedures.

Common side effects include headaches, low blood pressure, and dizziness.

3. Supportive Measures

Dietary changes (soft or liquid foods, eating slowly, drinking with meals, upright posture after eating)

Weight management and treatment of any co-existing conditions

Important Considerations & Risks

No treatment is perfect. Even the best non-surgical options carry risks (perforation, reflux, incomplete relief). Long-term success is generally highest with either POEM or well-performed pneumatic dilation.

Botox is the least durable option.

Early diagnosis and treatment at a center experienced in esophageal motility disorders usually yields the best outcomes.

Regular follow-up is necessary; achalasia is a chronic condition.

Strong recommendation: These are general medical facts, not personalized advice. Diagnosis usually requires high-resolution manometry, barium swallow, and endoscopy. Please consult a gastroenterologist who specializes in esophageal motility disorders (often at a major medical center) to determine which option is safest and most effective for your specific situation.

Would you like me to explain the differences between POEM and laparoscopic Heller myotomy, or tell you what questions to ask your doctor at the appointment?

**Model Qwen3-Max**

Answer text:

Yes, achalasia can often be treated without surgery, especially in early or mild cases. While surgery (such as Heller myotomy) is a definitive treatment option, several non-surgical approaches are available and commonly used:

1. Pneumatic Dilation

A balloon is inserted endoscopically into the lower esophageal sphincter (LES) and inflated to stretch and tear the muscle fibers.

Effective in 50-90 percent of patients, though may need repeating.

Risk: Small chance of esophageal perforation ( about 2-5 percent ).

2. Botulinum Toxin (Botox) Injection

Injected endoscopically into the LES to temporarily paralyze the muscle and reduce pressure.

Provides short-term relief (weeks to months), especially useful for older or high-risk patients.

Less effective long-term compared to dilation or surgery.

3. Medications

Calcium channel blockers (e.g., nifedipine) or nitrates (e.g., isosorbide dinitrate) can help relax the LES.

Generally less effective than other options and used when other treatments aren't feasible.

Often limited by side effects (e.g., headache, low blood pressure).

4. Peroral Endoscopic Myotomy (POEM)

Note: While technically minimally invasive and endoscopic, POEM is still considered a surgical procedure by many guidelines-but it avoids external incisions.

Highly effective with outcomes similar to laparoscopic Heller myotomy.

Choosing a Treatment

The best approach depends on:

Patient age and overall health

Severity of symptoms

Esophageal anatomy (e.g., presence of megaesophagus)

Access to specialized centers

Patient preference

Non-surgical options like pneumatic dilation or Botox are often tried first in patients who are not ideal surgical candidates or who wish to avoid surgery initially.

Important: Achalasia is a chronic condition with no cure, but treatments aim to relieve symptoms and improve quality of life. Untreated achalasia can lead to complications like aspiration, malnutrition, or esophageal cancer (rarely).

Would you like guidance on which treatment might be most appropriate based on specific patient factors (e.g., age, symptom duration, test results)?

**Q27. What is POEM for achalasia?**

**Model ChatGPT 5.5**

Answer text:

POEM stands for Peroral Endoscopic Myotomy. It is a minimally invasive endoscopic procedure used to treat achalasia, a disorder in which the esophagus has trouble moving food into the stomach because the lower esophageal sphincter (LES) does not relax properly.

In POEM, a gastroenterologist passes an endoscope through the mouth into the esophagus, creates a tunnel within the esophageal wall, and cuts part of the abnormal muscle layer - a myotomy - to help the LES relax and allow food and liquids to pass more easily into the stomach.

Why it's used for achalasia

It can improve swallowing difficulty, regurgitation, chest discomfort, and food sticking.

It is especially useful for certain achalasia subtypes, including type III/spastic achalasia, because the myotomy can be extended higher up the esophagus if needed.

It avoids external incisions because it is done entirely through the mouth.

How it compares with other treatments Other options include:

Pneumatic dilation: stretching the LES with a balloon.

Laparoscopic Heller myotomy: surgical cutting of the LES muscle, usually combined with an anti-reflux procedure.

Botulinum toxin injection: temporary relaxation of the LES, often used in patients who are not good candidates for more definitive therapy.

Main risks and side effects

Acid reflux/GERD is relatively common after POEM because the LES is loosened and, unlike Heller myotomy, POEM usually does not include an anti-reflux wrap.

Temporary chest pain, sore throat, or bloating may occur.

Less commonly: bleeding, infection, perforation, or complications related to anesthesia.

Bottom line: POEM is an incision-free endoscopic treatment for achalasia that cuts the tight esophageal muscle to improve swallowing. It is generally considered an effective, durable treatment, but patients often need follow-up for reflux afterward.

A good next step would be to ask a gastroenterologist whether your achalasia subtype - type I, II, or III - makes POEM the best option compared with Heller myotomy or pneumatic dilation.

**Model Claude Opus 4.7**

Answer text:

POEM for Achalasia

What is POEM?

POEM (Per-Oral Endoscopic Myotomy) is a minimally invasive endoscopic procedure used to treat achalasia, a disorder where the lower esophageal sphincter (LES) fails to relax, making swallowing difficult.

How POEM Works:

Performed under general anesthesia via endoscopy (through the mouth)

A small incision is made in the inner lining (mucosa) of the esophagus

The endoscope is tunneled through the submucosal layer

The muscle fibers of the LES and lower esophagus are cut (myotomy)

The mucosal entry point is closed with clips

No external incisions are needed

Advantages:

Scarless (no skin incisions)

Shorter hospital stay (1-2 days)

Faster recovery

High success rate ( greater than 90 percent )

Can treat all subtypes of achalasia, especially Type III (spastic) achalasia

Disadvantages:

Higher rate of post-procedure GERD/reflux ( about 30-50 percent )

Requires specialized expertise

No anti-reflux procedure can be added (unlike surgery)

Best Treatment for Achalasia

There is no single "best" treatment - it depends on patient factors, achalasia subtype, age, and local expertise. Current evidence supports:

First-Line Treatments (most effective):

Treatment Best For Success Rate

Laparoscopic Heller Myotomy + Dor/Toupet Fundoplication Type I & II, younger patients about 90 percent

Pneumatic Dilation (graded) Type II, older patients about 70-85 percent

POEM Type III (spastic), sigmoid esophagus, failed prior therapy greater than 90 percent

Second-Line / Less Durable:

Botulinum toxin injection - for elderly/high surgical risk patients; effects last 6-12 months

Medications (nitrates, calcium channel blockers) - least effective; for those unfit for procedures

Current Recommendations (Guidelines)

According to ACG, SAGES, and ISDE guidelines:

Type I & II achalasia: Laparoscopic Heller myotomy, POEM, or pneumatic dilation are all acceptable

Type III achalasia: POEM is preferred (longer myotomy possible)

Sigmoid/end-stage esophagus: POEM or esophagectomy in severe cases

Failed prior treatment: POEM is often the best salvage option

Bottom Line:

POEM and laparoscopic Heller myotomy have comparable efficacy, but POEM has more reflux while Heller has the advantage of an added anti-reflux wrap. The "best" treatment is individualized - based on achalasia type, patient health, and surgeon expertise.

Would you like more details on recovery, complications, or comparison between POEM and Heller myotomy?

**Model DeepSeek V4 Pro**

Answer text:

Peroral Endoscopic Myotomy (POEM) is a minimally invasive endoscopic procedure used to treat achalasia, a rare esophageal motility disorder where the lower esophageal sphincter (LES) fails to relax properly, making it difficult for food and liquids to pass into the stomach.

How POEM Works for Achalasia

The core problem in achalasia is the hypertensive, non-relaxing LES and often abnormal esophageal body peristalsis.

POEM directly targets the LES by creating a surgical myotomy (cutting the circular muscle fibers) from inside the esophagus, without external incisions.

By dividing the muscle, the LES pressure is permanently reduced, allowing passive emptying of the esophagus by gravity.

The Procedure (Simplified Steps)

Submucosal entry: An endoscope is inserted through the mouth into the esophagus. A small incision is made in the esophageal mucosa to access the submucosal space.

Tunnel creation: A submucosal tunnel is carefully created down past the gastro-esophageal junction (GEJ) into the upper stomach (cardia) using dissection and CO2 insufflation.

Myotomy: A specialized endoscopic knife cuts the inner circular muscle layer along the tunnel, extending 2-3 cm onto the gastric side to fully divide the LES.

Closure: The mucosal entry point is closed with endoscopic clips or sutures. The rest of the tunnel heals spontaneously.

Why POEM is Considered Effective

Minimally invasive: No thoracic or abdominal incisions; faster recovery than surgical (Heller) myotomy.

Precise myotomy length: The endoscopist can tailor the length of the muscle cut to the patient's anatomy.

Rapid symptom relief: Most patients experience significant improvement in dysphagia within days.

Short hospital stay: Often discharged after 1-2 days.

Risks and Considerations

Gastroesophageal reflux disease (GERD): Because the anti-reflux barrier is divided, up to 40-60 percent of patients may develop abnormal acid exposure, although not all develop severe symptoms. Proton pump inhibitors are routinely prescribed.

Perforation or leak: Although rare, full-thickness injury or mucosal tear can occur; most are managed endoscopically.

Bleeding, infection, pneumothorax/pneumoperitoneum: CO2 is used to minimize complications, but tracking these is part of standard monitoring.

Need for experienced centers: POEM is technically demanding; outcomes improve with high-volume operators.

Comparison with Laparoscopic Heller Myotomy (LHM)

Both relieve dysphagia effectively, but POEM may offer a shorter recovery and less post-operative pain.

LHM is usually combined with a partial fundoplication to reduce reflux; no fundoplication is added during POEM, which is why post-POEM GERD can be more common.

Choice depends on patient factors, achalasia subtype (POEM works well for type III, which has spastic features), and local expertise.

Who is a Candidate?

Typically, patients with confirmed achalasia who are fit for anesthesia and have no contraindications like severe esophageal varices, prior esophageal surgery that obliterated the submucosal plane, or uncorrectable coagulopathy.

Follow-up for you: Are you interested in how POEM compares to pneumatic dilation for achalasia, or would you like to know more about post-procedure diet and lifestyle adjustments?

**Model Gemini 3.1 Pro Thinking**

Answer text:

POEM stands for Peroral Endoscopic Myotomy. It is a state-of-the-art, minimally invasive procedure used to treat achalasia, a rare swallowing disorder where the muscles of the esophagus fail to push food down, and the lower esophageal sphincter (the valve between the esophagus and stomach) fails to relax.

By performing POEM, doctors can permanently relax this valve, allowing food and liquids to pass normally into the stomach.

How the POEM Procedure Works

Unlike traditional surgery, POEM does not require any cuts on the outside of the body. The entire procedure is performed through the mouth using an endoscope (a flexible tube with a camera).

Incision: The doctor inserts the endoscope through the mouth and into the esophagus, making a small incision in the innermost layer of the esophageal lining (mucosa).

Tunneling: A pathway (submucosal tunnel) is created within the wall of the esophagus to reach the tight muscle of the lower esophageal sphincter.

Myotomy (Cutting the muscle): The doctor uses a specialized tool to cut the tight muscle fibers of the lower esophagus and the top of the stomach, releasing the tension.

Closure: The original incision in the esophageal lining is closed using endoscopic clips, which eventually pass naturally through the digestive tract.

Advantages of POEM

Minimally Invasive: No external scars or skin incisions.

Fast Recovery: Most patients stay in the hospital for only a day or two and experience less pain compared to traditional laparoscopic surgery (Heller myotomy).

High Success Rate: It provides excellent long-term relief from swallowing difficulties for the vast majority of patients.

Potential Risks and Considerations

While POEM is highly effective and generally safe, it is important to be aware of the potential medical risks:

Gastroesophageal Reflux Disease (GERD): Because the valve is permanently loosened, stomach acid can easily splash back up. This is the most common side effect, occurring in a significant number of patients. It is usually manageable with daily acid-blocking medications (Proton Pump Inhibitors or PPIs).

Bleeding or Infection: As with any surgical procedure, there is a small risk of bleeding or infection at the surgical site.

Esophageal Perforation: There is a rare risk of a deeper tear in the esophageal wall that may require further intervention.

Optimization and Post-Procedure Care

If you or someone you know is considering or recovering from POEM, here are some best practices to optimize recovery:

Dietary Progression: Patients must strictly adhere to a liquid diet immediately after the procedure, slowly transitioning to soft foods, and eventually back to a normal diet over a few weeks.

Proactive Acid Management: Taking prescribed antacids or PPIs faithfully is crucial to prevent esophageal inflammation or damage from post-procedure acid reflux.

Long-term Monitoring: Regular follow-ups with a gastroenterologist, potentially including routine endoscopies or pH monitoring, are highly recommended to monitor for silent acid reflux.

Disclaimer: I am an AI, not a doctor. This information is for educational purposes and should not replace professional medical advice.

Would you like to know how POEM compares to other treatments for achalasia, such as the Laparoscopic Heller Myotomy or Pneumatic Dilation?

**Model Grok-4.3**

Answer text:

POEM for Achalasia stands for Peroral Endoscopic Myotomy.

It is a modern, minimally invasive endoscopic procedure used to treat achalasia, a rare esophageal motility disorder in which the lower esophageal sphincter (LES) fails to relax properly, making it difficult for food and liquids to pass into the stomach.

How POEM Works

The procedure is performed entirely through the mouth (peroral) using a flexible endoscope.

The doctor creates a small incision in the mucosal lining of the esophagus.

A tunnel is carefully made between the mucosal layer and the muscular layer of the esophagus.

The surgeon then cuts (myotomy) the inner circular muscle fibers of the LES and the lower esophagus, typically extending 6-10 cm up the esophagus and 2-3 cm onto the stomach.

The mucosal incision is closed with endoscopic clips.

This effectively weakens the non-relaxing sphincter, allowing food to pass more easily while preserving the outer longitudinal muscle fibers.

Advantages of POEM

No external incisions (scarless)

Very effective at relieving dysphagia (success rates about 90-95 percent in most studies)

Can treat all types of achalasia, including type III (spastic) achalasia, where other treatments are often less effective

Shorter recovery time compared to traditional Heller myotomy surgery

Can be performed on patients who have previously failed other treatments (balloon dilation, Botox, or surgical myotomy)

Comparison with Other Treatments

Treatment Approach Effectiveness Durability Main Risk

POEM Endoscopic Very High Excellent Reflux (GERD) about 30-50 percent

Heller Myotomy Laparoscopic surgery Very High Excellent Reflux (with fundoplication)

Pneumatic Balloon Dilation Endoscopic Good Moderate Perforation ( about 2-5 percent )

Botox Injection Endoscopic Moderate Short-term Needs repeated treatments

Main Side Effect

The most common long-term side effect is gastroesophageal reflux disease (GERD) because the procedure permanently disrupts the LES. Many patients need to take proton pump inhibitors (PPIs) afterward.

Would you like me to explain the differences between POEM and Heller myotomy in more detail, or go over what patients can expect before, during, and after the procedure?

**Model Qwen3-Max**

Answer text:

POEM stands for Peroral Endoscopic Myotomy. It is a minimally invasive endoscopic procedure used to treat achalasia, a disorder of the esophagus characterized by impaired relaxation of the lower esophageal sphincter (LES) and loss of normal esophageal peristalsis.

How POEM Works:

During POEM, an endoscope is inserted through the mouth into the esophagus. The surgeon then:

Creates a small incision in the inner lining (mucosa) of the esophagus.

Tunnels underneath the mucosa through the submucosal layer to reach the muscle layer.

Performs a myotomy-cutting the circular muscle fibers of the LES and extending it into the gastric cardia-thereby reducing pressure and allowing food/liquid to pass more easily into the stomach.

Closes the initial mucosal incision with endoscopic clips.

Advantages of POEM:

No external incisions (fully endoscopic)

Shorter hospital stay (often 1-2 days)

Rapid recovery

High success rates ( greater than 90 percent symptom improvement in many studies)

Comparable or superior efficacy to traditional surgical myotomy (e.g., Heller myotomy)

Potential Risks/Complications:

Gastroesophageal reflux disease (GERD) - occurs in a subset of patients post-POEM; often managed with proton pump inhibitors (PPIs).

Mucosal perforation (rare, usually manageable endoscopically)

Bleeding or infection (uncommon)

Pneumomediastinum or subcutaneous emphysema (usually self-limiting)

Patient Selection:

POEM is typically offered to patients with:

Confirmed achalasia (types I, II, or III based on high-resolution manometry)

Failed prior treatments (e.g., pneumatic dilation or botulinum toxin injection)

No severe comorbidities that increase procedural risk

It is now considered a first-line therapy for many patients with achalasia, especially type II, due to its efficacy and minimally invasive nature.

Would you like a comparison between POEM, Heller myotomy, and pneumatic dilation for achalasia?

**Q28. Who is suitable for POEM?**

**Model ChatGPT 5.5**

Answer text:

If you mean POEM = peroral endoscopic myotomy, it is usually suitable for people with achalasia or certain esophageal motility/spasm disorders that cause significant swallowing problems.

People who are often good candidates for POEM

POEM may be appropriate for:

Adults with confirmed achalasia

Especially if symptoms include trouble swallowing solids and liquids, regurgitation, chest pain, choking/coughing, or weight loss.

Diagnosis is usually confirmed with high-resolution esophageal manometry, often plus endoscopy and barium swallow.

Achalasia type III - spastic achalasia

POEM is often favored for type III because the endoscopic approach can allow a longer, tailored myotomy along the spastic segment. Current SAGES guidance favors POEM over Heller myotomy with fundoplication for subtype III. (sages.org)

Achalasia type I or II

POEM, laparoscopic Heller myotomy with fundoplication, and pneumatic dilation can all be effective options; choice depends on patient preference, anatomy, reflux risk, and local expertise. (sages.org)

People who prefer a less invasive, incision-free approach

POEM is done through the mouth using an endoscope, without skin incisions. (hopkinsmedicine.org)

Patients who have failed other treatments

Such as botulinum toxin injection, pneumatic dilation, or sometimes prior Heller myotomy, depending on anatomy and specialist assessment.

Selected patients with other spastic esophageal disorders

Such as diffuse esophageal spasm or jackhammer/nutcracker esophagus when symptoms are severe and medications have not helped. (hopkinsmedicine.org)

Children or older adults in selected cases

Age alone is usually not the deciding factor; overall health, anesthesia risk, and diagnosis matter more. Johns Hopkins notes that both children and adults can undergo POEM. (hopkinsmedicine.org)

People who may not be ideal candidates

POEM may be less suitable, or needs extra caution, if someone has:

Severe uncontrolled acid reflux or Barrett's esophagus, because POEM can increase reflux risk.

Large hiatal hernia, where Heller myotomy with fundoplication may be preferred.

Advanced "sigmoid" or very dilated esophagus, depending on severity.

Serious medical problems making general anesthesia unsafe.

Active esophageal infection, ulceration, tumor, or uncertain diagnosis.

Bleeding disorders or inability to stop certain blood thinners, unless safely managed.

Symptoms caused by something other than achalasia, such as cancer, stricture, eosinophilic esophagitis, or severe reflux-related narrowing.

Key trade-off

The main advantage of POEM is that it is highly effective and minimally invasive. The main downside is post-procedure reflux, because POEM does not include an anti-reflux fundoplication. Many patients need acid-suppressing medication such as a proton-pump inhibitor afterward. SAGES notes POEM with appropriate PPI use is a reasonable option compared with Heller myotomy plus fundoplication in adults with achalasia. (sages.org)

Bottom line

A suitable POEM candidate is typically someone with manometry-confirmed achalasia-especially type III-or another severe spastic esophageal motility disorder, whose symptoms significantly affect eating or quality of life, and who can tolerate general anesthesia and accept the possibility of long-term reflux treatment.

A good next step is to ask a gastroenterologist or esophageal motility specialist: "What achalasia subtype do I have, and how does my reflux risk compare with POEM versus Heller myotomy?"

**Model Claude Opus 4.7**

Answer text:

POEM (Per-Oral Endoscopic Myotomy)

POEM is a minimally invasive endoscopic procedure primarily used to treat achalasia and other esophageal motility disorders.

Ideal Candidates Include:

Primary Indications

Achalasia (all subtypes: Type I, II, and III)

Particularly effective for Type III (spastic) achalasia, where it's often considered first-line

Spastic esophageal disorders:

Diffuse esophageal spasm (DES)

Jackhammer esophagus

Hypertensive lower esophageal sphincter (LES)

Patient Profiles Suited for POEM

Patients who failed previous treatments (pneumatic dilation, botulinum toxin injection, or Heller myotomy)

Those who prefer a less invasive alternative to laparoscopic Heller myotomy

Patients with sigmoid-shaped esophagus (advanced achalasia)

Pediatric and elderly patients (good safety profile)

Patients with prior abdominal surgeries (avoids the abdominal approach)

Contraindications / Less Suitable Candidates

Absolute Contraindications

Severe coagulopathy that cannot be corrected

Inability to tolerate general anesthesia

Severe esophageal mucosal disease (extensive ulceration, candidiasis)

Relative Contraindications

Significant esophageal varices

Prior extensive mediastinal radiation

Prior endoscopic mucosal resection in the planned tunnel area

Severe pulmonary disease

Significant pre-existing GERD (since POEM doesn't include an anti-reflux procedure)

Pre-Procedure Evaluation Typically Requires

High-resolution esophageal manometry (confirms diagnosis)

Upper endoscopy (EGD)

Barium esophagram

Eckardt score assessment

Would you like more details on a specific aspect of POEM, such as the procedure itself, recovery, or comparison with other treatments?

**Model DeepSeek V4 Pro**

Answer text:

POEM (Peroral Endoscopic Myotomy) is a minimally invasive endoscopic procedure used primarily to treat achalasia and some other severe esophageal motility disorders. Determining suitability is a careful medical decision, made after comprehensive testing.

People who are typically suitable for POEM

Confirmed diagnosis of achalasia - especially Type I and Type II on high-resolution manometry. These types show the best outcomes with POEM.

Type III (spastic) achalasia may also be a candidate, but requires more detailed muscular mapping; the spastic segment can be targeted with a longer myotomy.

Other motility disorders (after medication/other treatments fail) - such as jackhammer esophagus, distal esophageal spasm, or esophagogastric junction outflow obstruction (EGJOO) - when manometry confirms the diagnosis and symptoms are severe.

Patients who did not respond to, or cannot tolerate, other first-line therapies (e.g., pneumatic dilation, Botox injection).

Those able to tolerate general anesthesia and a brief hospital stay (usually 1-3 days).

No serious anatomical or bleeding risks (see below).

People who are typically not suitable (contraindications)

Major esophageal anatomical changes: large esophageal or paraesophageal hernias, significant strictures, or a history of extensive esophageal surgery that distorts normal layers (relative contraindication).

Uncorrectable bleeding disorders or severe coagulopathy that cannot be reversed.

Esophageal varices or portal hypertension (makes the tunnel unsafe).

Severe esophagitis or untreated infection (should be treated first).

Advanced megaesophagus with sigmoid shape - POEM may still be offered, but success rates drop and alternative surgery (esophagectomy) is sometimes considered.

Severe cardiopulmonary disease making the procedure or its possible complications (pneumothorax, pneumomediastinum) too risky.

Severe reflux disease unprepared for post-procedural monitoring (POEM intentionally cuts the lower esophageal sphincter without an anti-reflux wrap, so reflux can worsen - careful patient selection and lifelong follow-up are critical).

How suitability is decided

Candidates undergo a full work-up:

High-resolution esophageal manometry (to confirm diagnosis and classify subtype)

Barium swallow/timed barium esophagram (to assess anatomy and emptying)

Upper endoscopy (to rule out tumors or strictures and evaluate esophageal anatomy)

Medical clearance for anesthesia

The final decision is made by a multidisciplinary team (gastroenterologist, surgeon, anesthesiologist). Patient preference, age, comorbidities, and risk of post-procedural reflux are all weighed.

Important note

POEM is a specialized procedure. While minimally invasive, it carries risks like perforation, bleeding, infection, post-POEM gastroesophageal reflux (up to 50 percent of patients, often manageable with PPIs), and transient air-related complications. Long-term reflux monitoring is part of routine care.

Follow-up question to continue the conversation: Would you like to know how POEM is actually performed step-by-step, or how its outcomes compare with the classic surgical treatment, laparoscopic Heller myotomy?

**Model Gemini 3.1 Pro Thinking**

Answer text:

Assuming you are referring to POEM (Peroral Endoscopic Myotomy), which is a minimally invasive endoscopic procedure used to treat swallowing disorders, suitability depends heavily on the patient's specific esophageal condition.

Here is a breakdown of who is generally a suitable candidate for the POEM procedure, as well as those who might not be:

Who is Highly Suitable for POEM?

Patients with Achalasia: POEM is primarily used to treat achalasia, a rare disorder where the esophagus fails to move food into the stomach, and the lower esophageal sphincter (LES) fails to relax. It is highly effective for all three types of achalasia (Type I, II, and III).

Note: POEM is often considered the preferred treatment for Type III achalasia, as the endoscopist can make a longer, customized incision in the esophageal muscle than is typically possible with traditional surgery (Heller myotomy).

Patients with Spastic Esophageal Disorders: Individuals suffering from conditions like Jackhammer esophagus (hypercontractile esophagus) or diffuse esophageal spasm (DES) are also excellent candidates.

Patients Who Failed Previous Treatments: People who have previously undergone pneumatic dilation, Botox injections, or even a laparoscopic Heller myotomy, but are still experiencing severe symptoms, can often be successfully treated with POEM.

Patients Preferring a Minimally Invasive Approach: Because POEM is performed entirely through the mouth using an endoscope, there are no incisions on the chest or abdomen, often leading to a faster recovery and less post-operative pain.

Who is NOT Suitable for POEM? (Contraindications)

Certain conditions make POEM unsafe or ineffective. These generally include:

Severe pulmonary (lung) or cardiovascular disease that makes general anesthesia high-risk.

Severe blood clotting disorders (coagulopathy).

A history of esophageal radiation therapy or esophageal cancer.

Severe, untreated erosive esophagitis.

Portal hypertension with esophageal varices (often related to severe liver disease).

Previous interventions that have caused severe scarring in the submucosal layer of the esophagus.

Potential Risks and Optimization Suggestions

Risk of GERD: The most common side effect of POEM is the development of Gastroesophageal Reflux Disease (GERD). Because the esophageal valve is permanently opened, stomach acid can splash up.

Optimization: Before considering POEM, a patient must undergo a comprehensive workup. This should include High-Resolution Manometry (HRM) to precisely diagnose the motility disorder, an upper endoscopy (EGD) to rule out cancer or severe inflammation, and a timed barium esophagram. Post-procedure, patients are often prescribed daily proton pump inhibitors (PPIs) to manage and prevent acid reflux.

Disclaimer: I am an AI, not a doctor. This information is for educational purposes. Anyone considering POEM should consult with a gastroenterologist or thoracic surgeon who specializes in esophageal motility disorders to discuss their specific case.

Are you asking for yourself or a loved one who has recently been diagnosed with a swallowing disorder, or are you researching medical procedures for a different purpose?

**Model Grok-4.3**

Answer text:

POEM (Peroral Endoscopic Myotomy) - Who is Suitable?

POEM is a highly effective, minimally invasive endoscopic procedure used primarily to treat esophageal motility disorders, especially Achalasia.

Ideal / Suitable Candidates for POEM

Condition Suitability for POEM Notes

Achalasia (All types: I, II, III) Excellent (First-line in many centers) Type II has best results, Type III also responds very well

Esophagogastric Junction Outflow Obstruction (EGJOO) Very Good Especially if confirmed on FLIP or manometry

Diffuse Esophageal Spasm (DES) Good When medications fail

Jackhammer (Hypercontractile) Esophagus Good to Moderate Best for patients with obstruction at the EGJ

Failed prior treatments (Failed Heller Myotomy, Botox, Pneumatic Dilation) Very Good POEM is often preferred after failed Heller myotomy

Type III Achalasia ("Spastic Achalasia") One of the best indications POEM is often superior to Heller myotomy here

Patient Profile Typically Suitable

Age: Usually 18-80 years (can be done in adolescents and carefully selected elderly)

Symptoms: Significant dysphagia, regurgitation, chest pain, weight loss

Failed or refused medical therapy (nitrates, calcium channel blockers, PDE-5 inhibitors)

Willing to undergo lifelong GERD management (high rate of reflux after POEM)

Good performance status (can tolerate general anesthesia and endoscopy)

No severe cardiopulmonary disease

Relative or Absolute Contraindications

Severe pulmonary disease (cannot tolerate CO2 insufflation)

Uncorrectable coagulopathy or platelet count less than 50,000

Advanced esophageal cancer or pseudoachalasia

Prior extensive esophageal surgery (relative - case by case)

Very advanced age + frailty ( greater than 85-90 with comorbidities)

Active esophageal infection or severe esophagitis

Hiatal hernia greater than 3-4 cm (relative, increases post-POEM reflux risk)

Bottom Line - Best Candidates:

Classic Achalasia (especially Type II and III)

Patients who have failed pneumatic dilation or Heller myotomy

Young patients who want a definitive, one-time procedure (instead of repeated dilations)

Patients with spastic disorders (Type III, DES, Jackhammer)

Would you like me to also explain:

How POEM compares to Heller Myotomy and Pneumatic Dilation?

Success rates and long-term outcomes?

What tests are needed before deciding on POEM?

Just tell me what you'd like to know next.

**Model Qwen3-Max**

Answer text:

POEM (Peroral Endoscopic Myotomy) is a minimally invasive endoscopic procedure primarily used to treat achalasia, a disorder of the esophagus that affects the ability to swallow due to failure of the lower esophageal sphincter (LES) to relax properly.

Who is suitable for POEM?

Ideal candidates for POEM typically include:

Patients diagnosed with achalasia

Confirmed by high-resolution manometry (showing absent peristalsis and impaired LES relaxation).

Often supported by barium swallow and endoscopy findings.

Patients who have not responded adequately to less invasive treatments, such as:

Pneumatic dilation

Botulinum toxin injection

Pharmacologic therapy

Patients of various ages, including elderly individuals, provided they are medically fit for anesthesia and endoscopy.

Those with different achalasia subtypes (Type I, II, or III), though Type II often shows the best response.

Patients who prefer a minimally invasive alternative to surgical myotomy (e.g., Heller myotomy), especially if they want to avoid external incisions.

Individuals with recurrent or persistent symptoms after prior interventions (e.g., failed Heller myotomy or repeated dilations).

Contraindications or cautionary considerations:

Severe cardiopulmonary disease that increases anesthesia risk

Uncorrected coagulopathy

Active esophageal infection or perforation

Prior extensive esophageal surgery that may distort anatomy

Suspected or confirmed esophageal cancer (must be ruled out before POEM)

Note: All patients should undergo thorough pre-procedure evaluation, including endoscopy, imaging, and manometry, to confirm diagnosis and rule out mimics like pseudoachalasia (which can be caused by malignancy).

Would you like a comparison between POEM and other treatment options like Heller myotomy or pneumatic dilation?

**Q29. What are the risks of POEM?**

**Model ChatGPT 5.5**

Answer text:

If by POEM you mean peroral endoscopic myotomy for achalasia or another esophageal motility disorder, the main risks are:

Common / important risks

Acid reflux / GERD: This is one of the biggest long-term risks. POEM cuts the lower esophageal sphincter muscle, which helps swallowing but can make reflux more likely. Some patients need long-term acid-suppressing medicine, and follow-up testing/endoscopy may be recommended. Studies and GI guidance note that objective reflux after POEM can be fairly common, even when symptoms are mild or absent. (asge.org)

Esophagitis: Inflammation or irritation of the esophagus from reflux; rarely, chronic uncontrolled reflux can contribute to strictures or Barrett's esophagus.

Chest/throat/abdominal pain, bloating, nausea after the procedure, usually temporary.

Bleeding from the cut/tunnel area.

Infection or fever, including mediastinitis, though serious infection is uncommon in experienced centers.

Perforation or leak: A tear or leak in the esophagus or stomach lining may require endoscopic repair, antibiotics, drainage, or rarely surgery.

Air/gas-related complications: Air can escape into surrounding spaces, causing pneumoperitoneum, pneumomediastinum, subcutaneous emphysema, or pneumothorax; many cases are managed conservatively, but some need decompression.

Aspiration or anesthesia-related risks, since POEM is done under general anesthesia.

Failure to improve symptoms or symptom recurrence: Some people may need repeat treatment, dilation, or another procedure later.

Difficulty completing the procedure: For example, if the esophagus is very narrow or anatomy is difficult. (mskcc.org)

How serious is the risk overall?

In expert hands, POEM is generally considered effective and relatively safe, with long-term clinical effectiveness reported above 90 percent in some series; however, reflux remains the most notable tradeoff. (asge.org)

Red flags after POEM

Seek urgent medical care if you develop fever, worsening chest or abdominal pain, shortness of breath, vomiting blood, black stools, inability to swallow liquids, severe vomiting, or signs of dehydration.

Risk varies a lot based on your diagnosis, age, anatomy, prior treatments, reflux history, and the experience of the center. Are you asking about POEM for achalasia, esophageal spasm, or G-POEM for gastroparesis?

**Model Claude Opus 4.7**

Answer text:

Risks and Complications of POEM (Per-Oral Endoscopic Myotomy)

POEM is a minimally invasive endoscopic procedure used primarily to treat achalasia and other esophageal motility disorders. While generally safe, it carries several potential risks:

Common/Minor Complications

Insufflation-related issues (from CO2 used during the procedure):

Subcutaneous emphysema

Pneumomediastinum

Pneumoperitoneum

Pneumothorax

Mucosal injury or perforation during tunnel creation

Minor bleeding within the submucosal tunnel

Chest or abdominal pain post-procedure

Sore throat

Significant Complications

Gastroesophageal reflux disease (GERD) - the most common long-term issue, occurring in 20-60 percent of patients (higher than with surgical Heller myotomy because no anti-reflux wrap is performed)

Esophagitis and potential Barrett's esophagus with chronic reflux

Significant bleeding requiring intervention

Leak from mucosal defect leading to mediastinitis

Aspiration pneumonia

Pleural effusion

Rare but Serious Complications

Tension capnoperitoneum requiring needle decompression

Full-thickness perforation

Sepsis or abscess formation

Cardiac arrhythmias

Death (very rare, less than 0.1 percent )

Long-Term Concerns

Treatment failure or symptom recurrence (5-10 percent of cases)

Need for repeat intervention (POEM, dilation, or surgery)

Persistent reflux requiring long-term PPI therapy

Risk-Reducing Factors

Experienced operators at high-volume centers have significantly lower complication rates

Routine post-procedure imaging or endoscopy can detect issues early

Long-term acid suppression and reflux monitoring are typically recommended

Overall, POEM has a major adverse event rate of approximately 1-3 percent , making it comparable in safety to laparoscopic Heller myotomy, though with a higher rate of postoperative reflux.

Would you like more information about how POEM compares to other achalasia treatments, or about managing post-POEM reflux?

**Model DeepSeek V4 Pro**

Answer text:

POEM (Peroral Endoscopic Myotomy) is a minimally invasive endoscopic procedure used primarily to treat achalasia and certain other esophageal motility disorders. While it has a high success rate and avoids external incisions, it carries specific risks that patients and clinicians should understand.

Immediate and early risks (procedural / within days)

Bleeding

Can occur from the mucosal entry site or during myotomy. Usually minor but sometimes requires endoscopic intervention.

Perforation

Full-thickness esophageal or gastric perforation, potentially leading to pneumothorax, pneumomediastinum, or pneumoperitoneum. Often managed conservatively, but severe cases may need surgery.

Mucosal injury / leak

Inadvertent mucosal breach or inadequate closure of the entry site can cause an esophageal leak, leading to mediastinitis or sepsis.

Infection

Mediastinitis, abscess formation, or systemic infection if a leak is present or extensive dissection occurs.

Subcutaneous emphysema / capnoperitoneum

CO2 insufflation can dissect into tissues, causing temporary facial/neck swelling or abdominal distension; usually benign but can be alarming and rarely compromises ventilation.

Cardiac arrhythmias / vagal response

Instrument manipulation near the esophagus may precipitate bradycardia or other arrhythmias.

Late risks (weeks to months)

Gastroesophageal reflux disease (GERD)

The most common long-term complication. POEM inherently divides the lower esophageal sphincter without a concomitant anti-reflux procedure; up to 40-60 percent of patients may develop abnormal acid exposure, and some require lifelong PPIs or even subsequent fundoplication.

Post-procedure dysphagia

Although treatment aims to relieve dysphagia, some patients experience persistent or recurrent dysphagia due to incomplete myotomy, fibrosis, or severe reflux-induced stricture.

Esophageal stricture

Scarring at the myotomy site or at the mucosal closure can lead to stricture requiring dilation.

Transient chest discomfort / pain

Common after the procedure; usually resolves spontaneously.

Rare but serious risks

Nerve injury (e.g., vagus nerve branches) - may affect gastrointestinal motility or heart rate.

Pleural injury leading to pleural effusion, often managed with drainage.

Fistula formation - abnormal connection between the esophagus and mediastinum or pleura.

Need for conversion to open surgery - if endoscopic closure fails or there is uncontrolled bleeding/perforation.

Risk mitigation and optimization

Appropriate patient selection: Not all achalasia subtypes respond equally; type III may benefit from a longer myotomy.

Pre-procedure planning: High-resolution manometry and timed barium esophagram help guide myotomy length.

Experienced operator: Volume and expertise dramatically reduce perforation and leak rates.

Meticulous closure: Reliable clipping/suturing of the mucosal entry site is crucial.

Anti-reflux strategy: Patients should be counselled about GERD risk; some centers perform a same-session transoral fundoplication (e.g., TIF) in selected cases (POEM+F).

Post-procedure monitoring: Early detection of leaks via imaging or endoscopic check; acid suppression for at least a few months and possibly longer.

Disclaimer: This information is for educational purposes and is not a substitute for professional medical advice. Treatment decisions must be made in consultation with a gastroenterologist or surgeon.

As a next step, would you like me to compare POEM with laparoscopic Heller myotomy regarding their respective risk profiles, or explain which achalasia subtypes might benefit most from POEM?

**Model Gemini 3.1 Pro Thinking**

Answer text:

Because the acronym POEM can refer to two very different, widely used concepts, the risks depend on the context of your question. I have outlined the risks and optimization strategies for both the medical procedure and the marketing framework below.

1. Medical Context: Peroral Endoscopic Myotomy (POEM)

In medicine, POEM is a minimally invasive endoscopic procedure used to treat swallowing disorders, most commonly achalasia.

Potential Risks & Complications:

Gastroesophageal Reflux Disease (GERD): This is the most common long-term risk. Because the procedure cuts the muscle at the lower esophageal sphincter to allow food to pass, it also removes the barrier that prevents stomach acid from splashing back up.

Bleeding: Minor bleeding during the procedure is common, but delayed or severe bleeding requiring intervention can occasionally occur.

Infection: As with any invasive procedure, there is a small risk of infection in the esophageal wall or chest cavity.

Mucosal Perforation: A tear in the inner lining of the esophagus.

Capnoperitoneum / Pneumothorax: Gas (CO2) used during the endoscopy can leak into the abdominal cavity or the space around the lungs, which may require decompression.

Optimization & Mitigation Suggestions:

Post-Operative Management: Routine prescription of Proton Pump Inhibitors (PPIs) is highly recommended to manage and prevent post-procedure acid reflux.

Surgeon Expertise: The risk of mucosal perforation and severe bleeding drops significantly when the procedure is performed at a high-volume center by a highly experienced endoscopist.

Patient Selection: Comprehensive pre-operative testing (like high-resolution manometry) ensures the patient is the right candidate for POEM rather than alternative surgical interventions (like a Heller myotomy).

Disclaimer: This information is for educational purposes. Always consult a board-certified gastroenterologist or surgeon for medical advice.

2. Business/Marketing Context: Paid, Owned, Earned Media (POEM)

In marketing, POEM is a framework used to categorize and strategize content and media distribution.

Potential Risks:

Siloing Channels: Treating Paid (ads), Owned (your website/blog), and Earned (PR/reviews) as separate entities rather than an integrated ecosystem. This leads to disjointed customer experiences.

Over-reliance on Paid Media: If a business relies purely on Paid media, customer acquisition costs (CAC) will continually rise, and traffic will stop the moment the budget runs out.

Unpredictability of Earned Media: Earned media is highly credible but difficult to control. A PR crisis or a change in social media algorithms can severely damage or limit your earned reach.

Neglecting Owned Media: Failing to invest in platforms you control (like your email list or website) leaves you vulnerable to third-party platform changes (e.g., Google search updates).

Optimization & Mitigation Suggestions:

Create a Converged Media Strategy: Use Paid media to amplify your best Owned media, which in turn generates Earned media. For example, run Facebook ads (Paid) on a high-quality whitepaper (Owned) to encourage industry influencers to share it (Earned).

Focus on First-Party Data: Continually drive traffic from Paid and Earned sources back to your Owned platforms to capture email addresses and build a community you control.

Track Blended ROI: Use analytics to track how these channels interact, rather than measuring their return on investment in isolation.

Which specific context of "POEM" were you referring to? If it is related to a different technical acronym (like a specific software framework), please let me know and I would be happy to dive into its technical risks and provide code-level best practices!

**Model Grok-4.3**

Answer text:

Risks of POEM (Peroral Endoscopic Myotomy)

POEM is a highly effective minimally invasive endoscopic procedure used to treat achalasia and other esophageal motility disorders. While it has excellent success rates (typically 90-95 percent short-term clinical success), it still carries risks. These can be divided into intra-procedural, early post-procedural, and long-term complications.

Major Risks

Risk Approximate Rate Details

Gastroesophageal Reflux Disease (GERD) 30-60 percent The most common long-term consequence. POEM completely disrupts the lower esophageal sphincter without adding an anti-reflux wrap (unlike Heller myotomy with fundoplication). Many patients require lifelong proton-pump inhibitors.

Mucosal Perforation 5-10 percent Tear in the inner lining of the esophagus during the procedure. Usually recognized and closed endoscopically, but can lead to leakage.

Pneumoperitoneum / Pneumomediastinum 5-30 percent (varies by detection method) Air escaping into the abdominal or chest cavity. Most cases are benign and resolve spontaneously; only a small percentage require intervention (needle decompression).

Bleeding 1-3 percent Can occur during tunneling or myotomy. Major bleeding requiring transfusion or intervention is rare ( less than 1 percent ).

Infection (mediastinitis, abscess) less than 1 percent Serious but uncommon when prophylactic antibiotics are used.

Pleural effusion / Pneumothorax 1-5 percent Usually managed conservatively; chest tube is rarely needed.

Technical Failure / Incomplete Myotomy 2-5 percent May result in persistent dysphagia requiring repeat POEM, Heller myotomy, or dilation.

Serious complications (esophageal leak, severe mediastinitis, death) 0.1-0.5 percent Very low in high-volume expert centers.

Other Considerations

Operator Dependence: Complication rates are significantly lower when performed by experienced endoscopists (typically greater than 50-100 cases). Learning curve is steep.

Delayed Complications: Rare cases of esophageal diverticulum formation or stricture have been reported months to years later.

Need for Re-intervention: Approximately 10-20 percent of patients may need additional treatment (repeat POEM, dilation, or surgery) within 5 years.

Risk Mitigation

Performed in high-volume centers

Use of CO2 insufflation (instead of air)

Prophylactic antibiotics

Careful patient selection (avoiding in patients with severe cardiopulmonary disease, prior extensive esophageal surgery, or unfavorable anatomy)

Important: This information is for educational purposes only and is not a substitute for personalized medical advice. Complication rates can vary widely based on your specific anatomy, prior treatments, and the center's experience.

Follow-up question: Are you asking about POEM because you or a loved one is considering treatment for achalasia, or are you comparing it to Heller myotomy or pneumatic dilation? I can provide a more tailored comparison if you'd like.

**Model Qwen3-Max**

Answer text:

POEM (Peroral Endoscopic Myotomy) is a minimally invasive endoscopic procedure used primarily to treat achalasia-a disorder where the lower esophageal sphincter (LES) fails to relax properly, causing difficulty swallowing. While POEM is generally effective and has become a standard treatment in many centers, it carries several potential risks and complications:

1. Gastroesophageal Reflux Disease (GERD)

Most common long-term risk: Up to 20-40 percent of patients may develop new-onset or worsened GERD after POEM because the myotomy can reduce LES pressure excessively.

Requires lifelong monitoring and often proton pump inhibitors (PPIs).

2. Mucosal Perforation

During submucosal tunneling or myotomy, accidental injury to the esophageal mucosa can occur.

Usually managed endoscopically during the procedure (e.g., clips), but severe cases may require surgery.

3. Bleeding

Minor bleeding at the mucosal entry site or within the submucosal tunnel is possible.

Significant hemorrhage is rare ( less than 1-2 percent ) but may require intervention.

4. Pneumomediastinum / Pneumoperitoneum

Air insufflation during the procedure can leak into the mediastinum or peritoneal cavity.

Often asymptomatic and resolves spontaneously, but large accumulations may cause respiratory distress or require drainage.

5. Infection

Rare, but mediastinitis or abscess formation can occur if contamination happens during perforation.

6. Incomplete Symptom Relief or Recurrence

Some patients may not achieve full symptom relief or may experience recurrence over time, requiring repeat intervention (e.g., redo-POEM, Heller myotomy, or pneumatic dilation).

7. Esophageal Diverticula or Strictures

Long-term structural changes are uncommon but possible due to altered esophageal motility or scarring.

8. Anesthesia-Related Risks

As with any procedure under general anesthesia, risks include aspiration, allergic reactions, or cardiovascular events.

Mitigation Strategies:

Preoperative evaluation: High-resolution manometry and endoscopy to confirm diagnosis and rule out mimics (e.g., pseudoachalasia).

Postoperative pH monitoring: To detect silent GERD and guide PPI therapy.

Experienced endoscopists: Complication rates are significantly lower in high-volume centers.

If you're considering POEM or managing a patient post-procedure, discussing these risks with a multidisciplinary team (gastroenterologist, surgeon) is essential.

Would you like a comparison of POEM versus laparoscopic Heller myotomy in terms of efficacy and safety?

**Q30. Does POEM cause acid reflux afterward?**

**Model ChatGPT 5.5**

Answer text:

Yes. POEM - peroral endoscopic myotomy, usually done for achalasia - commonly increases the risk of acid reflux/GERD afterward.

Why it happens: POEM cuts the tight muscle at the lower esophageal sphincter so food can pass into the stomach more easily. But that same sphincter normally helps prevent stomach acid from refluxing upward, and POEM does not include an anti-reflux procedure like a fundoplication.

How common? Studies vary, but objective testing often finds reflux in a substantial portion of patients. One study found symptoms in about 39 percent , objective GERD in about 51 percent , and severe objective GERD in about 19 percent after POEM; importantly, some people had abnormal acid reflux without symptoms. (pmc.ncbi.nlm.nih.gov) Other reviews commonly cite abnormal acid exposure around 40-50 percent and endoscopic esophagitis around 30-40 percent after POEM. (gastroenterologyandhepatology.net)

What this means in practice:

Many patients are placed on a proton pump inhibitor such as omeprazole, pantoprazole, or esomeprazole after POEM.

Reflux symptoms may include heartburn, sour/bitter taste, regurgitation, chest burning, cough, hoarseness, or worsening symptoms when lying down.

Because reflux can be "silent," experts recommend monitoring for GERD after POEM, and some guidelines suggest considering periodic endoscopic evaluation even if symptoms are absent. (gastrojournal.org)

If reflux symptoms occur after POEM, ASGE guidance supports endoscopic evaluation, given the relatively high rate of post-POEM GERD. (news.gastro.org)

Call your GI doctor promptly if you have trouble swallowing that is worsening, vomiting, black stools, weight loss, anemia, severe chest pain, or reflux symptoms that persist despite medication.

A useful next step is to ask your gastroenterologist: "What is my reflux monitoring plan after POEM-PPI duration, follow-up endoscopy, and whether I need pH testing?"

**Model Claude Opus 4.7**

Answer text:

POEM and Acid Reflux

Yes, gastroesophageal reflux disease (GERD) is the most common side effect of peroral endoscopic myotomy (POEM), and it's an important consideration when choosing this treatment.

Why It Happens

POEM treats achalasia by cutting the lower esophageal sphincter (LES) muscle to allow food to pass into the stomach. However, unlike the surgical alternative (Heller myotomy), POEM doesn't include an anti-reflux procedure (fundoplication). This means the weakened LES can also allow stomach acid to flow back up.

How Common Is It?

Studies show:

Reflux symptoms: about 20-30 percent of patients

Abnormal acid exposure on pH testing: about 40-50 percent

Reflux esophagitis on endoscopy: about 20-30 percent

Rates are generally higher than after laparoscopic Heller myotomy with fundoplication.

Management

Reflux after POEM is usually manageable:

Proton pump inhibitors (PPIs) - effective in most patients, sometimes needed long-term

Lifestyle measures - elevating the head of the bed, avoiding late meals

Endoscopic surveillance - to monitor for esophagitis or Barrett's esophagus

Endoscopic or surgical fundoplication - for refractory cases

Bottom Line

POEM is highly effective for achalasia, but post-procedure GERD is common and often requires ongoing acid-suppression therapy. Discuss with your gastroenterologist whether POEM, Heller myotomy with fundoplication, or pneumatic dilation is best for your situation.

This information is educational and not a substitute for personalized medical advice.

**Model DeepSeek V4 Pro**

Answer text:

Yes, POEM (Peroral Endoscopic Myotomy) frequently causes or significantly worsens gastroesophageal reflux (GERD) afterward. It's one of the procedure's most recognized long-term side effects.

Why does reflux occur after POEM?

POEM treats achalasia by cutting the muscular layer of the lower esophageal sphincter (LES) from inside the esophagus. This relieves the obstruction that makes swallowing difficult, but it also permanently weakens the LES. A weakened or incompetent LES is the primary barrier that normally prevents stomach acid from washing back into the esophagus, so reflux becomes much more likely.

How common is it?

Abnormal acid exposure (detected by 24-hour pH testing) is reported in 40-60 percent of patients after POEM, even when symptoms are mild or absent.

Erosive esophagitis (visible on endoscopy) affects roughly 20-30 percent of patients post-POEM, although some series show higher rates.

Many patients are asymptomatic, so the absence of heartburn does not rule out damaging reflux. Regular surveillance endoscopy is often recommended.

How is it managed?

Routine acid suppression: Most centers prescribe a proton-pump inhibitor (PPI, e.g., omeprazole or pantoprazole) once or twice daily after POEM, often indefinitely, even if the patient reports no heartburn.

Lifestyle measures: Elevating the head of the bed, avoiding late meals, and weight management are advised.

Refractory cases: In rare situations where PPI therapy fails and severe reflux or regurgitation persists, surgical anti-reflux procedures (e.g., laparoscopic fundoplication) may be considered, but this is uncommon.

Risk awareness and optimization

Trade-off: POEM provides excellent relief of dysphagia, but at the cost of a high reflux risk. When discussing treatment options, a laparoscopic Heller myotomy combined with a partial fundoplication (the traditional surgical alternative) offers similar swallowing improvement with a significantly lower reflux rate (10-15 percent abnormal acid exposure).

Patient selection: For patients with pre-existing GERD, large hiatal hernia, or a tendency toward acid-related complications, a Heller myotomy with fundoplication may be preferable. A thorough pre-procedure assessment (including manometry, pH testing, and endoscopic evaluation) helps guide the choice.

Post-POEM surveillance: After POEM, regular upper endoscopy is recommended to screen for reflux-related complications like erosive esophagitis, stricture, or Barrett's esophagus, especially in those with poor symptom-reflux correlation.

Disclaimer: I'm an AI assistant, not a doctor. This information is for educational purposes only. Treatment decisions should be made with your gastroenterologist or surgeon, taking your full clinical picture into account.

Would you like to know more about how the reflux risk after POEM compares with that after pneumatic dilation or laparoscopic Heller myotomy, or about the long-term monitoring schedule after the procedure?

**Model Gemini 3.1 Pro Thinking**

Answer text:

Yes, acid reflux (Gastroesophageal Reflux Disease, or GERD) is the most common side effect and long-term complication of the POEM (Peroral Endoscopic Myotomy) procedure.

Here is a breakdown of why this happens, the potential risks involved, and how it is typically managed.

Why does POEM cause acid reflux?

POEM is highly effective at treating achalasia (a condition where the esophagus cannot move food into the stomach). It works by cutting the muscles of the lower esophageal sphincter (LES).

Normally, the LES acts as a one-way valve that opens to let food in, and closes tightly to prevent stomach acid from splashing back up into the esophagus. Because POEM permanently cuts and loosens this muscle to allow food to pass, the protective barrier against stomach acid is significantly weakened.

Potential Risks and Statistics

High Prevalence: Studies show that between 30 percent and 50 percent (and in some objective pH-monitoring studies, up to 60 percent ) of patients develop acid reflux after POEM.

Silent Reflux: Some patients have "silent reflux," meaning acid is damaging the esophagus, but they do not feel the typical heartburn symptoms.

Long-Term Risks: If left unmanaged, chronic acid reflux can lead to esophagitis (inflammation), esophageal strictures (narrowing), or Barrett's esophagus, which is a precancerous condition.

Best Practices & Optimization for Post-POEM Care

If you undergo POEM, gastroenterologists generally recommend the following clinical best practices to manage this risk:

Medication (PPIs): The vast majority of post-POEM reflux is successfully managed with a daily Proton Pump Inhibitor (PPI), such as omeprazole or pantoprazole, to reduce stomach acid production.

Routine Monitoring: It is highly recommended to undergo regular upper endoscopies (EGD) and pH testing in the years following a POEM procedure to check for silent reflux and monitor the health of the esophageal lining.

Lifestyle Adjustments: Sleeping with the head of the bed elevated, avoiding eating 3-4 hours before bedtime, and limiting trigger foods (caffeine, alcohol, spicy foods) can significantly optimize your recovery and comfort.

Alternative/Add-on Procedures: In rare cases where medication isn't enough, some patients may require an additional anti-reflux procedure (like a partial fundoplication or TIF) later on.

Disclaimer: I am an AI, not a doctor. This information is for educational purposes. Always consult with a gastroenterologist or a thoracic surgeon regarding the risks and benefits of surgical procedures and how they apply to your specific medical history.

Are you currently considering POEM as a treatment for achalasia, or are you looking for ways to manage reflux after having already had the procedure?

**Model Grok-4.3**

Answer text:

Yes, POEM can cause or worsen acid reflux (GERD) afterward, and it is one of the most common side effects.

What the Evidence Shows

POEM (Peroral Endoscopic Myotomy) is a highly effective treatment for achalasia and other esophageal motility disorders. It has excellent success rates ( greater than 90 percent in most studies) for relieving swallowing difficulties.

However, because the procedure completely disrupts the lower esophageal sphincter (LES) - the valve that normally prevents stomach acid from flowing back up - post-procedure gastroesophageal reflux disease (GERD) is very frequent.

Studies report the following rates of abnormal acid exposure after POEM:

30-50 percent of patients have objective evidence of reflux on pH testing at 1-2 years.

Up to 60 percent may have some degree of reflux symptoms or esophagitis on endoscopy.

About 20-40 percent of patients require regular proton-pump inhibitor (PPI) medication afterward.

Why It Happens

Unlike a Heller myotomy performed with a fundoplication (which wraps the stomach around the esophagus to create an anti-reflux barrier), standard POEM does not include an anti-reflux procedure. This makes reflux more common after POEM than after laparoscopic Heller myotomy with partial fundoplication.

Factors That Influence Risk

Length of the myotomy (especially gastric extension greater than 2-3 cm) increases reflux risk.

Type of achalasia and pre-existing hiatal hernia can also play a role.

Some centers now perform POEM + F (POEM with fundoplication) or use other anti-reflux techniques to reduce this complication.

Management

Most patients who develop reflux after POEM respond well to once- or twice-daily PPIs. In a smaller subset, reflux can be severe or refractory, sometimes requiring additional procedures (e.g., transoral incisionless fundoplication/TIF, magnetic sphincter augmentation, or laparoscopic fundoplication).

Important: Every patient should undergo objective reflux testing (usually 24-hour pH-impedance monitoring) 6-12 months after POEM, even if they feel fine, because many have "silent" reflux that can damage the esophagus over time.

Would you like me to explain the differences in reflux rates between POEM, Heller myotomy with fundoplication, and the newer POEM-F technique, or tell you what post-POEM monitoring protocol most experts recommend?

**Model Qwen3-Max**

Answer text:

POEM (Peroral Endoscopic Myotomy) is a minimally invasive surgical procedure primarily used to treat achalasia, a condition where the lower esophageal sphincter (LES) fails to relax properly, making it difficult for food and liquid to pass into the stomach.

Does POEM cause acid reflux afterward?

Yes, gastroesophageal reflux disease (GERD) or acid reflux is a known potential complication after POEM.

Here's why:

In achalasia, the LES is abnormally tight-but this also means it often prevents acid reflux.

During POEM, the surgeon cuts (myotomizes) the muscle fibers of the LES to allow it to open more easily.

This can reduce LES pressure too much, leading to inadequate barrier function against stomach acid, resulting in postoperative GERD.

How common is it?

Studies report post-POEM GERD rates ranging from 20 percent to 40 percent , though many cases are mild or asymptomatic.

Not all patients experience noticeable symptoms-some only show esophagitis or abnormal pH studies without classic heartburn.

Management strategies:

Routine post-op monitoring: Many centers perform endoscopy and/or pH testing after POEM, even in asymptomatic patients.

Proton pump inhibitors (PPIs): Often prescribed prophylactically or if reflux symptoms develop.

Lifestyle modifications: Elevating the head of the bed, avoiding late meals, reducing alcohol/caffeine, etc.

Antireflux procedures: In rare cases with severe reflux, a fundoplication (e.g., Dor or Toupet) may be considered-sometimes even performed at the time of POEM in high-risk patients.

Key point:

While POEM is highly effective for relieving dysphagia in achalasia, the trade-off can be an increased risk of acid reflux, which requires ongoing monitoring and management.

If you or someone you know is considering or has undergone POEM, discussing reflux risk and follow-up plans with a gastroenterologist or surgeon is essential.

Would you like information on how to reduce reflux risk after POEM or compare it with other achalasia treatments like Heller myotomy?

**Q31. What should I eat after POEM?**

**Model ChatGPT 5.5**

Answer text:

After POEM (peroral endoscopic myotomy) for achalasia/esophageal motility disorders, diet is usually advanced slowly so the esophagus can heal and to reduce the risk of leak, irritation, or food getting stuck. Follow your own surgeon/GI team's instructions first, because protocols vary by center.

Typical post-POEM diet progression

Right after the procedure

Often nothing by mouth until your care team confirms it's safe, sometimes after an esophagram/leak check.

Then you may start clear liquids.

Days 1-2: clear liquids

Examples:

Water

Broth

Apple juice or white grape juice

Tea

Ice pops without fruit pieces

Gelatin

Northwestern's G-POEM nutrition plan uses a similar staged approach-NPO on procedure day, then clear liquids for days 1-2-though G-POEM is for gastroparesis rather than esophageal POEM. (nm.org)

Next several days: full liquids / smooth purees

Examples:

Protein shakes

Milk or milk alternatives

Smooth yogurt without chunks

Cream soups, strained

Smoothies with no seeds or pulp

Pudding/custard

Thin oatmeal or cream of wheat, if approved

UCLA's post-POEM handout says foods should be blenderized/pureed to a smooth consistency and strained if needed to remove seeds, lumps, or chunks. (uclahealth.org)

Then: soft foods

When your doctor clears you:

Scrambled eggs

Soft fish

Mashed potatoes

Soft pasta

Cottage cheese

Soft cooked vegetables

Bananas

Applesauce

Soft, moist ground meats

Eat small, frequent meals, chew very well, and sip fluids.

Foods to avoid at first

Until your team says otherwise, avoid:

Bread, rice, tough meats, raw vegetables

Nuts, seeds, popcorn, chips

Spicy or acidic foods if reflux is an issue

Carbonated drinks if they cause bloating

Alcohol

Large meals

Reflux precautions

Reflux is common after POEM because the lower esophageal sphincter is loosened. Many patients are prescribed acid-suppressing medication such as a PPI. Cleveland Clinic notes that POEM can increase the chance of acid reflux afterward. (my.clevelandclinic.org)

Helpful habits:

Don't lie down for 2-3 hours after eating

Eat smaller meals

Sleep with your head elevated if you have nighttime reflux

Take your acid medication exactly as prescribed

Call your doctor urgently if you have

Fever

Severe chest, neck, or abdominal pain

Shortness of breath

Vomiting that won't stop

Trouble swallowing liquids

Black stools or vomiting blood

Worsening pain after eating

If you tell me how many days post-POEM you are and whether this was esophageal POEM for achalasia or G-POEM for gastroparesis, I can suggest a more specific food list for your current stage.

**Model Claude Opus 4.7**

Answer text:

Diet After POEM (Per-Oral Endoscopic Myotomy)

After POEM surgery for achalasia, your diet typically progresses in stages. Here's a general guideline, though always follow your surgeon's specific instructions:

Stage 1: Clear Liquids (Day 1-2)

Water, broth, clear juices (apple, white grape)

Gelatin, ice pops

Weak tea, electrolyte drinks

Stage 2: Full Liquids (Days 2-7)

Milk, smoothies (no seeds)

Cream soups (strained)

Yogurt (smooth), pudding

Protein shakes

Cream of wheat, thin oatmeal

Stage 3: Soft/Pureed Foods (Week 2)

Mashed potatoes

Scrambled eggs

Cottage cheese

Well-cooked, soft pasta

Soft fish, ground meats

Bananas, applesauce

Steamed soft vegetables

Stage 4: Regular Diet (After 3-4 weeks)

Gradually reintroduce normal foods as tolerated.

General Tips

Eat slowly and chew thoroughly

Small, frequent meals rather than large ones

Sit upright while eating and for 30-60 minutes after

Stay hydrated between meals

Avoid:

Carbonated drinks (initially)

Spicy, acidic foods (may irritate)

Tough meats, bread, raw vegetables (early on)

Alcohol and caffeine (early recovery)

Eating within 2-3 hours of bedtime (to reduce reflux risk)

Watch For

Since POEM can cause reflux, your doctor may prescribe a PPI (like omeprazole). Contact your doctor if you experience chest pain, fever, difficulty swallowing liquids, or vomiting.

Would you like more details on any specific stage or recipes that work well during recovery?

**Model DeepSeek V4 Pro**

Answer text:

After a POEM (Peroral Endoscopic Myotomy) - a minimally invasive procedure for achalasia or other esophageal motility disorders - your swallowing mechanism and the lower esophageal sphincter have been altered, so what you eat and how you eat matter a great deal during recovery. Your diet will typically progress through several stages. Always follow your surgeon's or dietitian's specific instructions, but here is a common framework:

Stage 1: Clear Liquids (first 1-2 days)

Water, ice chips

Clear broth (vegetable, chicken, or beef, strained)

Clear fruit juice without pulp (apple, white grape)

Plain gelatin (Jell-O)

Popsicles without fruit bits

Weak tea, clear sports drinks

Avoid: anything with pulp, dairy, red/purple dyes if you need to detect bleeding later, and carbonated beverages.

Stage 2: Full Liquids (next 3-7 days, as toleration)

All clear liquids plus:

Smooth dairy (milk, yogurt drinks, kefir, thin smoothies)

Cream soups (strained, no chunks)

Nutritional supplement drinks (Ensure, Boost, etc.)

Thin, strained cooked cereals (cream of wheat, rice porridge, watery oatmeal)

Smooth pudding, custard

Tip: Sip slowly; take small amounts to test swallowing comfort. Stop if you feel food "sticking".

Stage 3: Soft / Pureed Diet (typically weeks 2-4)

Smooth mashed potatoes, pur ed vegetables

Scrambled eggs, soft tofu

Pureed lean meats / fish (e.g., salmon mousse, chicken pur e)

Applesauce, smooth hummus

Soft ripe avocado

Smooth cottage cheese, ricotta

Well-cooked pasta cut very small or pur ed

Consistency: Food should be the texture of a smooth paste with no lumps, seeds, or skins. You might need to add broth, milk, or gravy to achieve the right consistency.

Stage 4: Soft Mechanical Diet (weeks 4-8, gradually)

Tender, moist, finely chopped meats (ground chicken, flaked fish)

Soft breads (pancakes, soft dinner rolls - no crusty bread)

Steamed, soft vegetables (cooked until fork-tender)

Soft, canned fruits (peaches, pears)

Soft cheeses, well-cooked pasta, rice

Moistened cereals

Stage 5: Return to a Regular Diet (after about 2-3 months)

You can reintroduce more textures, but many people need to eat mindfully for life. You may still need to avoid:

Tough, dry, or fibrous meats (steak, pork chops)

Hard, crusty breads / bagels / dough

Sticky foods (peanut butter, doughy pizza)

Spicy, acidic, or very hot-temperature foods (can irritate the esophagus)

Carbonated drinks and alcohol - they can increase reflux and discomfort

Eating Tips for Long-Term Success

Small, frequent meals (5-6 mini-meals) rather than large volumes.

Chew thoroughly, even if the food is soft. Make it liquid in your mouth.

Sit upright and stay upright for at least 30-45 minutes after eating.

Drink fluids between meals, not with meals, to avoid overfilling the esophagus.

Chase bites with sips of warm water if food seems hesitant.

Avoid heavy meals within 2-3 hours of bedtime to reduce reflux risk.

Risk Note

Because POEM disrupts the lower esophageal sphincter, gastroesophageal reflux (GERD) is a potential long-term side effect. Your diet can help manage this; many patients are also prescribed acid-reducing medication. If you experience heartburn, regurgitation, or food getting stuck, contact your doctor.

Disclaimer: This information is for general educational purposes and not a substitute for personalized medical advice. Your recovery plan may differ based on your specific case and your surgeon's protocol.

Follow-up: Are you asking for yourself or a family member? Knowing whether you're in the early days post-op or weeks out would help me tailor the suggestions more precisely - feel free to share how your recovery is going.

**Model Gemini 3.1 Pro Thinking**

Answer text:

Disclaimer: I am an AI, not a doctor or a registered dietitian. The following information is for general educational purposes. Always strictly follow the specific discharge instructions provided by your gastroenterologist or surgical team, as your individual protocol may vary based on how your specific Peroral Endoscopic Myotomy (POEM) procedure went.

Recovering from a POEM procedure (typically performed for achalasia) requires a careful, phased diet to allow the incisions in your esophagus to heal and to prevent complications.

Here is the standard progression for a post-POEM diet:

Phase 1: Clear Liquids (Usually Days 1-2)

Immediately after the procedure (and often after a swallow study confirms there are no leaks), you will start with clear liquids. These are liquids you can see through at room temperature.

What to eat/drink: Water, apple or white grape juice, clear broths (chicken, beef, or vegetable), plain Jell-O (not red or purple), herbal tea, and electrolyte drinks.

What to avoid: Carbonated beverages, citrus juices, coffee, and alcohol.

Phase 2: Full Liquids (Usually Days 3-7)

If you tolerate clear liquids well, your doctor will graduate you to thicker liquids.

What to eat/drink: Milk (dairy or plant-based), smooth cream soups (strained, no chunks), protein shakes, smooth yogurt, pudding, and ice cream (without nuts or candy pieces).

What to avoid: Anything with lumps, seeds, or pieces of food.

Phase 3: Soft / Pur ed Diet (Usually Weeks 2-3)

During this phase, foods should be easy to chew and swallow, requiring very little breakdown in the esophagus.

What to eat/drink: Mashed potatoes, scrambled eggs, oatmeal, applesauce, very tender flaky fish, well-cooked soft vegetables, and soft pasta.

What to avoid: Raw vegetables, tough meats (like steak or dry chicken), bread (which can clump and get stuck), nuts, and seeds.

Phase 4: Regular Diet (Usually Week 4 and beyond)

You will gradually reintroduce solid foods into your diet.

Introduce one new texture at a time.

Continue to be cautious with dense breads, tough meats, and raw, fibrous vegetables until you are sure how your esophagus handles them.

Potential Risks & Optimization Suggestions

Potential Risks:

Acid Reflux (GERD): Because the POEM procedure relaxes the valve at the bottom of the esophagus, acid reflux is the most common side effect.

Food Impaction: Eating solid foods too early or not chewing enough can cause food to get stuck, straining the healing incision.

Optimization & Best Practices:

Take your PPI: Your doctor will almost certainly prescribe a Proton Pump Inhibitor (PPI) like omeprazole to reduce stomach acid and prevent it from damaging the healing esophagus. Take this exactly as prescribed.

Chew thoroughly and eat slowly: Make sure food is practically a liquid in your mouth before you swallow.

Eat smaller, more frequent meals: Avoid overfilling your stomach.

Stay upright: Sit upright for at least 30 to 60 minutes after eating. Avoid eating within 3 hours of going to bed to prevent nighttime acid reflux.

Temperature matters: Avoid foods and drinks that are piping hot or freezing cold, as extreme temperatures can trigger esophageal spasms.

To help me provide more tailored context, are you currently preparing for an upcoming POEM procedure, or are you already in the recovery phase?

**Model Grok-4.3**

Answer text:

After POEM (Peroral Endoscopic Myotomy), follow a staged diet to allow the esophageal tunnel and myotomy site to heal properly.

Standard Post-POEM Diet Progression

Most centers follow a protocol similar to this (always follow your surgeon's specific instructions, as they may vary):

Phase 1: Clear Liquids (usually first 24 hours after a normal contrast swallow study)

Water, electrolyte drinks (Pedialyte, Gatorade), clear broth, apple juice (no pulp), Jell-O, popsicles.

Goal: Stay hydrated and test tolerance.

Phase 2: Full Liquids / Pureed Diet (typically Days 2-14)

Smoothies (protein powder + banana + yogurt or milk)

Greek yogurt, kefir, pudding, custard

Blended soups (cream of mushroom, butternut squash, etc.)

Mashed potatoes thinned with broth or milk

Protein shakes (Ensure Clear, Premier Protein, Orgain, etc.)

Applesauce, mashed banana, pureed avocado

Phase 3: Soft / Easy-to-Chew Diet (usually Weeks 3-4)

Scrambled eggs, soft omelets

Flaky fish (salmon, cod), ground turkey or chicken

Well-cooked pasta, soft rice, quinoa

Cottage cheese, ricotta, soft cheeses

Cooked vegetables (carrots, zucchini, squash) that are very soft

Bananas, canned peaches/pears (in juice), melon

Oatmeal, cream of wheat

Phase 4: Regular Diet (after 4 weeks, as tolerated) You can gradually reintroduce more foods, but many patients continue to:

Chew extremely well

Eat small, frequent meals

Avoid eating 3 hours before lying down

Important Things to Avoid (especially first 4 weeks)

Tough meats, steak, pork

Bread, bagels, pizza crust (can feel "stuck")

Raw vegetables, salads, apple skins, grapes with skin

Nuts, seeds, popcorn, granola

Spicy foods, tomato-based sauces, citrus, coffee, chocolate, mint

Carbonated drinks, alcohol

Very hot or very cold foods in large amounts

Extra Tips Specific to POEM Patients

Reflux is very common after POEM because the lower esophageal sphincter is opened. Most patients are prescribed a proton pump inhibitor (PPI) twice daily for at least 4-8 weeks. Avoid acid-triggering foods.

Stay upright for 30-60 minutes after eating.

Prioritize high protein (aim for 60-80g/day) to support healing - protein shakes and Greek yogurt are your friends.

Many people lose weight in the first month; focus on calorie-dense liquids if needed.

This is general guidance based on common protocols from high-volume POEM centers. Your doctor or dietitian should have given you written instructions and possibly a contrast swallow study result that dictates exactly when you can advance.

Would you like me to give you a sample 7-day meal plan for the full-liquid or soft-food phase, or are you looking for high-protein smoothie recipes that taste good?

**Model Qwen3-Max**

Answer text:

After undergoing a POEM (Peroral Endoscopic Myotomy) procedure-which is used to treat achalasia or other esophageal motility disorders-your diet needs to be carefully managed to allow proper healing and reduce the risk of complications like reflux or discomfort.

General Post-POEM Diet Guidelines:

Phase 1: Clear Liquids (First 24-48 hours)

Water, clear broths, apple juice, gelatin (no red dye), electrolyte drinks

Avoid carbonated beverages, alcohol, caffeine

Phase 2: Full Liquids (Days 2-5)

Smoothies (no chunks), milk, yogurt (thin consistency), cream soups, protein shakes

Continue avoiding carbonation and acidic juices (e.g., orange juice)

Phase 3: Soft/Pureed Foods (Days 5-14)

Mashed potatoes, oatmeal, applesauce, scrambled eggs, well-cooked cereals, soft pasta, pureed vegetables

Chew thoroughly and eat slowly-even soft foods should be well-masticated

Eat small, frequent meals (5-6 per day)

Stay upright for at least 30-60 minutes after eating to reduce reflux risk

Phase 4: Gradual Return to Regular Diet (After about 2 Weeks)

Only if tolerated and approved by your doctor

Start with tender, moist foods (e.g., flaky fish, ground meat, steamed veggies)

Avoid tough, dry, crunchy, or fibrous foods initially (e.g., steak, raw carrots, nuts, bread crusts)

Important Tips:

Stay hydrated but sip slowly-don't gulp.

Avoid lying down after meals to minimize acid reflux (common after POEM due to reduced lower esophageal sphincter pressure).

Your doctor may prescribe a proton pump inhibitor (PPI) long-term to manage reflux.

Listen to your body: If a food causes pain, regurgitation, or difficulty swallowing, stop and consult your care team.

Always follow your surgeon's specific instructions, as protocols can vary slightly between centers.

Would you like a sample 1-day meal plan for the soft-food phase?

**Q32. What is laparoscopic Heller myotomy?**

**Model ChatGPT 5.5**

Answer text:

Laparoscopic Heller myotomy is a minimally invasive surgical procedure used mainly to treat achalasia, a disorder in which the lower esophageal sphincter - the muscle valve between the esophagus and stomach - fails to relax properly.

In achalasia, food and liquid have trouble passing from the esophagus into the stomach, causing symptoms such as:

Difficulty swallowing

Regurgitation of food or liquid

Chest discomfort

Weight loss

Heartburn-like symptoms

During a Heller myotomy, the surgeon cuts the tight muscle fibers of the lower esophageal sphincter and often part of the lower esophageal muscle. This helps the sphincter relax and allows food to pass more easily into the stomach.

"Laparoscopic" means the operation is done through several small incisions using a camera and specialized instruments, rather than through a large open incision.

Because cutting the sphincter can increase the risk of acid reflux, the procedure is commonly combined with a partial fundoplication, where part of the stomach is wrapped around the lower esophagus to reduce reflux.

In short: laparoscopic Heller myotomy is a minimally invasive surgery that relieves swallowing difficulty by loosening the tight valve at the bottom of the esophagus.

A related question to discuss with a doctor would be: How does Heller myotomy compare with POEM or pneumatic dilation for achalasia?

**Model Claude Opus 4.7**

Answer text:

Laparoscopic Heller Myotomy

Laparoscopic Heller myotomy is a minimally invasive surgical procedure used to treat achalasia, a disorder in which the lower esophageal sphincter (LES) fails to relax properly, making it difficult for food and liquid to pass into the stomach.

The Procedure

The surgery is performed through several small incisions in the abdomen using a laparoscope (a thin camera) and specialized instruments. The surgeon:

Cuts the muscle fibers of the lower esophageal sphincter and a portion of the upper stomach (typically 6 cm along the esophagus and 2 cm onto the stomach)

Leaves the inner mucosal lining intact, only dividing the muscle layers

Usually adds a partial fundoplication (such as a Dor or Toupet wrap) to prevent acid reflux, which is a common side effect after the myotomy

What It Treats

Primarily achalasia, but it may also be used for:

Esophageal spasm disorders

Hypertensive lower esophageal sphincter

Benefits Compared to Open Surgery

Smaller incisions and less scarring

Reduced postoperative pain

Shorter hospital stay (typically 1-2 days)

Faster recovery and return to normal activities

Lower risk of complications

Success Rate and Outcomes

Success rate of approximately 85-95 percent in relieving swallowing difficulties (dysphagia)

Most patients experience significant, long-lasting improvement

Some may still require periodic dilations or other treatments later

Potential Risks

Esophageal or gastric perforation

Bleeding

Acid reflux (mitigated by the fundoplication)

Recurrence of symptoms

Need for conversion to open surgery (rare)

Alternatives

Pneumatic balloon dilation

Botulinum toxin injection

Peroral endoscopic myotomy (POEM) - a newer endoscopic alternative

Would you like more information on any specific aspect, such as recovery, comparison with POEM, or what to expect before surgery?

**Model DeepSeek V4 Pro**

Answer text:

Laparoscopic Heller myotomy is a minimally invasive surgical procedure used to treat achalasia, a rare esophageal motility disorder. In achalasia, the lower esophageal sphincter (LES) fails to relax properly, and the esophagus loses its ability to push food into the stomach, leading to difficulty swallowing, regurgitation, chest pain, and weight loss.

What the procedure involves

Approach: The surgeon operates through several small incisions in the abdomen (laparoscopy) rather than opening the chest or making a large abdominal cut.

Myotomy: The muscle layer of the lower esophagus and the upper part of the stomach (the LES) is carefully cut, releasing the over-tight sphincter so food can pass more easily. The inner mucosal lining is left intact.

Fundoplication: To reduce the risk of postoperative gastroesophageal reflux (stomach acid entering the now-relaxed esophagus), a partial fundoplication (wrapping part of the stomach around the lower esophagus) is often done at the same time-most commonly a Dor (anterior) or Toupet (posterior) wrap.

Why laparoscopic?

Compared to open surgery, the laparoscopic approach offers:

Less postoperative pain

Shorter hospital stay (usually 1-2 days)

Quicker return to normal activities

Smaller scars

Potential risks and complications (risk awareness)

While generally safe, the procedure is not without risks. It's important for patients to be aware of:

Perforation of the esophageal or stomach mucosa (about 5-10 percent risk, usually repaired immediately if recognized during surgery)

Bleeding or infection

Dysphagia recurrence over time (in some cases, further intervention may be needed)

Gastroesophageal reflux disease (GERD) even with fundoplication; some patients may need long-term acid-suppressing medication

Anesthesia-related complications

Conversion to open surgery if technical difficulties arise

Optimization and best-practice considerations

To achieve the best outcome and reduce complications:

Select a high-volume center and experienced surgeon: Surgeons who perform this procedure regularly have better success rates and lower perforation rates.

Preoperative evaluation: High-resolution manometry to confirm the type of achalasia, barium swallow, and endoscopy to rule out pseudoachalasia (e.g., tumor at the gastroesophageal junction).

Tailored myotomy length: Too short and symptoms may persist; too long risks severe reflux. Standard is 4-6 cm in the esophagus and extending 2-3 cm onto the gastric cardia.

Postoperative diet progression: Start with clear liquids, then soft foods, and gradually reintroduce solids over a few weeks to allow healing and reduce pressure on the surgical site.

Long-term follow-up: Periodic symptom assessment, and possibly pH testing or endoscopy if reflux symptoms develop, to prevent Barrett's esophagus.

For eligible patients, an even less invasive option is peroral endoscopic myotomy (POEM), which avoids abdominal incisions altogether but typically does not include a fundoplication, leading to a higher incidence of postoperative reflux. Discuss the pros and cons of each approach with your gastroenterologist and surgeon.

Are you exploring treatment options for achalasia, either for yourself or someone close to you? I'd be happy to outline what to expect during recovery or compare this procedure with pneumatic dilation and POEM in more detail.

**Model Gemini 3.1 Pro Thinking**

Answer text:

Laparoscopic Heller myotomy is a minimally invasive surgical procedure primarily used to treat achalasia, a rare swallowing disorder.

To understand the surgery, it helps to understand the condition. In people with achalasia, the nerve cells in the esophagus degenerate. As a result, the esophagus loses its ability to squeeze food down into the stomach, and the muscular ring at the bottom of the esophagus (the lower esophageal sphincter, or LES) fails to relax. This causes food and liquids to back up in the esophagus, leading to difficulty swallowing, chest pain, and regurgitation.

Here is a breakdown of how the procedure works and what it entails:

How the Procedure Works

Laparoscopic Approach: Instead of making a large open incision in the chest or abdomen, the surgeon makes several small (keyhole) incisions in the abdomen. A laparoscope (a thin tube with a camera and light) and specialized surgical instruments are inserted through these incisions.

The Myotomy: The surgeon carefully cuts (myotomy) the outer, thick muscle layers of the lower esophagus and the upper part of the stomach. By cutting these tight muscles, the pressure of the sphincter is relieved, allowing food and liquids to pass easily into the stomach by gravity.

Fundoplication (Best Practice Optimization): Because cutting the lower esophageal sphincter destroys the body's natural barrier against stomach acid, a common side effect of this surgery is severe acid reflux (GERD). To prevent this, surgeons almost always perform a partial fundoplication (such as a Dor or Toupet fundoplication) at the same time. This involves wrapping the upper part of the stomach partially around the lower esophagus to create a new low-pressure valve that prevents acid from backing up.

Potential Risks & Considerations

While generally safe and highly effective (with a success rate of over 85 percent ), all surgeries carry risks. If you or someone you know is considering this procedure, it is important to be aware of the following:

Esophageal Perforation: There is a small risk of accidentally cutting through the inner lining of the esophagus (the mucosa) during the muscle division. If this happens, it is usually repaired immediately during the surgery, but it may require a longer hospital stay and delayed feeding.

Gastroesophageal Reflux Disease (GERD): Even with a fundoplication, some patients may develop chronic acid reflux requiring daily antacid medication.

Recurrent Symptoms: In some cases, the swallowing difficulty may return years later, either because the cut healed closed or the esophagus completely lost its shape (end-stage achalasia).

General Surgical Risks: Bleeding, infection, and adverse reactions to general anesthesia.

Advantages of the Laparoscopic Approach

Compared to traditional open surgery, the laparoscopic method offers significantly less postoperative pain, a shorter hospital stay (usually 1 to 2 days), a faster return to normal activities, and minimal scarring.

Would you like to know more about the typical recovery timeline for this surgery, or are you interested in learning about alternative treatments for achalasia, such as the endoscopic POEM procedure?

**Model Grok-4.3**

Answer text:

Laparoscopic Heller myotomy is a minimally invasive surgical procedure used to treat achalasia, a disorder in which the lower esophageal sphincter (LES) fails to relax properly, making it difficult for food and liquids to pass into the stomach.

What It Involves

During the procedure, the surgeon:

Makes 4-6 small incisions (typically 5-10 mm) in the abdomen.

Inserts a laparoscope (thin camera) and specialized instruments.

Carefully cuts the muscle fibers of the lower esophageal sphincter (the Heller myotomy) while leaving the inner mucosal lining intact.

This permanently loosens the sphincter so food can empty from the esophagus into the stomach more easily.

The surgery almost always includes a partial fundoplication (usually a Dor or Toupet wrap), in which the top part of the stomach is wrapped around the esophagus. This helps prevent acid reflux, which is a common side effect after the sphincter is cut.

Key Facts

Primary goal: Relieve the obstruction caused by the non-relaxing LES.

Success rate: 85-95 percent of patients experience significant long-term relief of dysphagia (difficulty swallowing).

Typical duration: 1.5-3 hours.

Hospital stay: Usually 1-2 nights.

Recovery: Most patients return to normal activities within 1-2 weeks.

Compared to Other Treatments

Treatment Invasiveness Durability Reflux Risk

Laparoscopic Heller Myotomy Surgical (minimally invasive) Excellent (long-term) Low (with fundoplication)

Pneumatic Balloon Dilation Endoscopic Moderate

Peroral Endoscopic Myotomy (POEM) Endoscopic Excellent Higher

Botox Injection Endoscopic Temporary (months) Low

Who It's For

It is typically recommended for:

Patients with confirmed achalasia (especially Types I and II).

Younger, healthier patients who want a durable, one-time solution.

Patients who have failed or prefer not to repeat endoscopic treatments (balloon dilation or Botox).

Risks (relatively low but include):

Perforation of the esophagus ( about 1-5 percent )

Postoperative gastroesophageal reflux

Dysphagia that persists or recurs (rare)

Bleeding, infection, or anesthesia complications

Would you like me to explain how this procedure compares in detail to POEM (Peroral Endoscopic Myotomy), or would you like information on what patients can expect during recovery and dietary changes after surgery?

**Model Qwen3-Max**

Answer text:

Laparoscopic Heller myotomy is a minimally invasive surgical procedure used to treat achalasia, a disorder of the esophagus that affects its ability to move food toward the stomach. In achalasia, the lower esophageal sphincter (LES)-a ring of muscle at the junction between the esophagus and stomach-fails to relax properly during swallowing, leading to difficulty swallowing (dysphagia), regurgitation, chest pain, and sometimes weight loss.

What the Procedure Involves:

Myotomy: The surgeon cuts (divides) the muscle fibers of the LES to reduce pressure and allow food and liquids to pass more easily into the stomach.

Laparoscopic approach: Instead of a large open incision, the surgery is performed through several small incisions in the abdomen using a laparoscope (a thin tube with a camera) and specialized instruments.

Partial fundoplication: Often, a Dor or Toupet fundoplication (a type of partial wrap of the stomach around the lower esophagus) is added after the myotomy to help prevent gastroesophageal reflux (GERD), which can occur after the LES is weakened.

Benefits of the Laparoscopic Approach:

Shorter hospital stay (often 1-2 days)

Less postoperative pain

Faster recovery and return to normal activities

Reduced risk of infection and hernia compared to open surgery

Success Rate:

Laparoscopic Heller myotomy is highly effective, with symptom improvement in 85-95 percent of patients. Long-term outcomes are generally good, though some patients may require additional treatment over time.

Potential Risks/Complications:

Gastroesophageal reflux (if anti-reflux procedure is inadequate)

Esophageal perforation (rare but serious)

Incomplete relief of symptoms

Gas-bloat syndrome (especially if fundoplication is too tight)

After surgery, patients typically follow a liquid or soft diet for a period and gradually advance to regular foods under medical guidance.

Would you like to know how this compares to other treatments for achalasia, such as POEM (Peroral Endoscopic Myotomy) or pneumatic dilation?

**Q33. What is the difference between POEM and Heller myotomy?**

**Model ChatGPT 5.5**

Answer text:

POEM and Heller myotomy are two procedures used mainly to treat achalasia, a disorder where the lower esophageal sphincter doesn't relax properly and food has trouble passing into the stomach.

Quick comparison

Feature POEM Heller myotomy

Full name Peroral Endoscopic Myotomy Laparoscopic Heller Myotomy

Approach Done through the mouth using an endoscope Done through small abdominal incisions using laparoscopy

What is cut? The tight muscle layer of the esophagus/lower esophageal sphincter from inside the esophageal wall The tight muscle layer from outside the esophagus

Incisions/scars No external incisions Several small abdominal incisions

Anti-reflux procedure Usually not done at the same time Usually combined with a partial fundoplication, an anti-reflux wrap

Reflux risk afterward Generally higher Generally lower, because fundoplication is added

Recovery Often quicker, less external pain Slightly more surgical recovery, though still minimally invasive

Best suited for Often favored for type III/spastic achalasia, because a longer tailored myotomy can be done Well-established option for type I/II achalasia and when reflux control is a priority

Long-term track record Newer, but now widely accepted Longer historical track record

Main practical difference

POEM is an endoscopic "inside-out" myotomy, performed through the mouth with no external cuts. Heller myotomy is a laparoscopic "outside-in" surgical myotomy, usually paired with an anti-reflux wrap.

Both are effective for achalasia. Current guidelines consider either POEM with appropriate acid suppression/follow-up or Heller myotomy with fundoplication reasonable for many adults with type I or II achalasia, while POEM is often favored for type III achalasia because it can more easily extend the muscle cut higher up the esophagus. (sages.org)

Reflux is the big tradeoff

The biggest downside of POEM is a higher chance of gastroesophageal reflux disease - GERD - afterward, since it does not include a fundoplication. Many patients need acid-suppressing medication such as a PPI and follow-up testing. Heller myotomy usually includes a partial fundoplication, which helps reduce reflux risk. (sages.org)

Which is "better"?

Neither is universally better. It depends on:

Achalasia subtype - type III often favors POEM.

Reflux history - significant pre-existing reflux may favor Heller with fundoplication.

Prior treatments or surgeries

Local expertise - outcomes depend heavily on the experience of the endoscopist/surgeon.

Patient preference regarding incisions, recovery, reflux risk, and long-term medication.

In short: POEM is less externally invasive and very flexible; Heller myotomy has the advantage of an added anti-reflux procedure and longer surgical track record.

If you're asking for yourself, the next useful step is to know your achalasia type from high-resolution manometry - type I, II, or III - because that strongly influences which option may fit best.

**Model Claude Opus 4.7**

Answer text:

POEM vs. Heller Myotomy

Both procedures treat achalasia (and other esophageal motility disorders) by cutting the lower esophageal sphincter (LES) muscle to allow food passage, but they differ significantly in approach.

Heller Myotomy (Laparoscopic Heller Myotomy - LHM)

Approach: Laparoscopic (through small abdominal incisions)

Technique: Surgeon cuts the muscle fibers of the LES from the outside of the esophagus

Length of myotomy: Typically 6-8 cm (limited length)

Anti-reflux procedure: Usually combined with a partial fundoplication (Dor or Toupet) to prevent GERD

Established since: 1990s (open version since 1913)

Recovery: 2-4 weeks

Reflux risk: Lower due to added fundoplication

POEM (Per-Oral Endoscopic Myotomy)

Approach: Entirely endoscopic (through the mouth - no external incisions)

Technique: Endoscopist creates a submucosal tunnel in the esophageal wall, then cuts the muscle from the inside

Length of myotomy: Can be longer and customized (especially useful in Type III achalasia / spastic disorders)

Anti-reflux procedure: None performed

Established since: 2008 (Inoue, Japan)

Recovery: Faster, shorter hospital stay

Reflux risk:Higher rate of GERD post-procedure (often requiring PPIs)

Key Comparison

Feature Heller Myotomy POEM

Invasiveness Laparoscopic Endoscopic (incisionless)

Myotomy length Limited Flexible/longer

Anti-reflux wrap Yes No

Post-op GERD Lower Higher

Best for Type III achalasia Less ideal Preferred

Cosmetic result Small scars No scars

Efficacy (symptom relief) about 90 percent about 90 percent (comparable)

When Each Is Preferred

POEM: Type III (spastic) achalasia, jackhammer esophagus, diffuse esophageal spasm, prior failed Heller, sigmoid esophagus

Heller: Patients at high risk for GERD, those preferring a single procedure with anti-reflux protection

Both have comparable efficacy ( about 90 percent symptom relief), and choice often depends on local expertise, achalasia subtype, and patient factors.

**Model DeepSeek V4 Pro**

Answer text:

Peroral Endoscopic Myotomy (POEM) and Heller myotomy (usually laparoscopic Heller myotomy with fundoplication) are both procedures that cut the lower esophageal sphincter (LES) to relieve dysphagia in achalasia. The key differences lie in the access method, the need for an anti-reflux procedure, and the risk profile, especially for postoperative acid reflux.

Quick comparison

Aspect POEM Heller myotomy

Access Entirely through the mouth (per-oral, endoscopic). No skin incisions. Usually laparoscopic (keyhole) or robotic abdominal surgery; rarely open.

Myotomy technique A submucosal tunnel is created endoscopically; the inner circular muscle layer is cut while leaving the outer longitudinal muscle intact. The outer muscle of the LES is cut directly from the abdominal side.

Length of myotomy Often extends higher into the esophagus (variable, typically 6-15 cm), which can give excellent dysphagia relief. Standard: about 6-8 cm on the esophagus, 2-3 cm onto the gastric cardia.

Anti-reflux procedure None built-in (though some centers add an endoscopic fundoplication). This is a major distinction. Almost always combined with a partial fundoplication (Dor or Toupet) to prevent reflux.

Hospital stay Usually shorter (1-2 days). Typically 1-3 days.

Recovery Faster return to oral intake and normal activities. Slightly longer; post-op pain from abdominal incisions.

GERD risk Higher - 20-50 percent may develop abnormal acid exposure, and up to 50 percent can have esophagitis on endoscopy. No mechanism to prevent reflux. Lower - fundoplication actively prevents acid reflux; still, some reflux can occur.

Scarring / adhesions No external scars, minimal adhesions. Small incisions, internal adhesions possible (relevant for future surgery).

Applicability Can be used for type I, II, and type III achalasia (spastic) because the myotomy can be extended high. Also possible in sigmoid esophagus and after failed Heller. Standard for type I and II; may be less effective for severe spastic achalasia (type III) where a longer myotomy is needed.

Complication profile Risk of mucosal injury, pneumomediastinum, subcutaneous emphysema, capnothorax; rarely mediastinitis. Risk of esophageal/gastric perforation, splenic injury, vagal nerve injury, wrap-related dysphagia, gas-bloat syndrome.

Cost May be higher due to specialised endoscopy equipment and expertise. Often lower, but depends on healthcare system.

Technical risks & optimisation advice

For POEM:

Risk: Significant GERD post-procedure because the LES is destroyed without any anti-reflux barrier.

Optimisation: Patient selection is critical - avoid in patients with pre-existing severe reflux. Consider POEM plus an endoscopic fundoplication (POEM+F) where available. Post-operatively, lifelong proton pump inhibitor (PPI) therapy is often recommended, and regular pH testing may be warranted.

Risk: Mucosal perforation during tunnel creation (1-5 percent ).

Optimisation: Use CO2 insufflation to reduce mediastinal/thoracic pressure, meticulous closure of the mucosotomy with clips or sutures. A leak test is advised.

For Heller myotomy:

Risk: Persistent or recurrent dysphagia if the myotomy is too short (especially on the gastric side).

Optimisation: Extend the myotomy at least 2-3 cm onto the gastric cardia; intra-operative endoscopy can confirm LES relaxation and identify any untreated high-pressure zones.

Risk: Wrap-related dysphagia if fundoplication is too tight; gas bloat syndrome.

Optimisation: Construct a loose (floppy) 270 Toupet or anterior Dor fundoplication; avoid a full 360 Nissen wrap, which tends to be too constricting.

Which one to choose?

Choice depends on achalasia subtype, patient preference regarding incisions vs. GERD risk, and surgeon expertise. In type III achalasia, POEM may be superior because a longer myotomy can be tailored to spastic segments. Heller with fundoplication is often favoured for young patients with normal BMI to minimise lifelong reflux.

Are you discussing these options for a specific achalasia subtype or for a particular patient scenario? I can help compare expected outcomes (e.g., Eckardt score improvement, long-term satisfaction) if that would be useful.

**Model Gemini 3.1 Pro Thinking**

Answer text:

Both POEM (Peroral Endoscopic Myotomy) and Heller myotomy (usually performed laparoscopically, known as LHM) are highly effective, minimally invasive procedures used to treat achalasia, a rare swallowing disorder where the esophagus struggles to move food into the stomach because the lower esophageal sphincter (LES) fails to relax.

While both procedures share the same goal-cutting the muscle of the LES to allow food to pass-they differ significantly in their approach, technique, and side-effect profiles.

Here is a detailed breakdown of the differences:

1. The Approach (How the surgery is performed)

Heller Myotomy (LHM): This is a surgical approach performed from the outside of the esophagus. A surgeon makes 4 to 5 small keyhole incisions in the abdomen (laparoscopy). Instruments are inserted into the abdominal cavity to cut the outer muscle layers of the lower esophagus and the top of the stomach.

POEM: This is an endoscopic approach performed from the inside of the esophagus. There are no cuts on the skin. A gastroenterologist or surgeon passes an endoscope (a flexible tube with a camera) through the mouth and down the throat. They create a small tunnel within the layers of the esophageal wall to access and cut the muscle from the inside.

2. Acid Reflux (GERD) Management

Heller Myotomy: Because cutting the sphincter permanently destroys the barrier that stops stomach acid from washing up, LHM is almost always combined with a fundoplication. This involves wrapping the top part of the stomach around the lower esophagus to create a new, artificial anti-reflux valve.

POEM: Because POEM is done through the mouth from the inside, a fundoplication cannot be performed at the same time. Consequently, patients who undergo POEM have a significantly higher risk of developing post-procedure acid reflux (GERD) compared to those who have LHM. Many POEM patients must take daily acid-suppressing medication (PPIs) long-term.

3. Subtypes of Achalasia

Heller Myotomy: Excellent for Achalasia Types I and II.

POEM: While highly effective for all types, POEM is often considered the gold standard for Type III Achalasia (spastic achalasia). In Type III, a longer portion of the esophageal muscle spasms. Because POEM is done from the inside, the doctor can easily extend the muscle cut much higher up the esophagus than is safely possible with laparoscopic surgery.

4. Recovery and Invasiveness

Heller Myotomy: Involves minor abdominal incisions. Recovery usually involves 1-2 days in the hospital and a few weeks of activity restriction.

POEM: Entirely incisionless on the body surface. Recovery is often slightly faster with less external pain, usually requiring a 1-night hospital stay for observation. Both procedures require a strict liquid-to-soft food diet transition over several weeks.

Potential Risks and Clinical Optimization

Disclaimer: I am an AI, not a doctor. The following is for educational purposes. Always consult a gastroenterologist or thoracic surgeon for medical advice.

Shared Risks: Both procedures carry risks of bleeding, infection, and esophageal perforation (a hole in the esophagus), though severe complications are rare in experienced hands.

Optimization/Patient Selection: The choice between POEM and LHM should be highly personalized.

If a patient already has severe acid reflux or a large hiatal hernia, LHM is usually preferred so the hernia can be repaired and a fundoplication performed.

If a patient has had previous abdominal surgeries causing scar tissue, POEM might be safer to avoid navigating the abdominal cavity.

Post-procedure optimization requires strict adherence to dietary guidelines to allow the esophageal mucosa to heal without tearing.

Would you like to know more about the specific dietary recovery process following these procedures, or are you looking for information regarding a specific type of achalasia?

**Model Grok-4.3**

Answer text:

POEM vs. Heller Myotomy: Key Differences

Both POEM (Peroral Endoscopic Myotomy) and Heller Myotomy are highly effective treatments for achalasia, a disorder in which the lower esophageal sphincter (LES) fails to relax properly. They both work by cutting the muscle fibers of the LES (a myotomy) to reduce its pressure so food and liquid can pass into the stomach more easily. However, they differ significantly in technique, invasiveness, and side-effect profiles.

1. Procedure Approach

Heller Myotomy: A surgical procedure, almost always performed laparoscopically (keyhole surgery through 4-5 small incisions in the abdomen). The surgeon cuts the outer muscle layer of the esophagus and upper stomach from the outside.

POEM: A completely endoscopic procedure performed entirely through the mouth. No skin incisions are made. The doctor creates a submucosal tunnel in the esophageal wall, then cuts the inner circular muscle fibers from the inside.

2. Anti-Reflux Procedure (Fundoplication)

Heller: Almost always combined with a partial fundoplication (usually a Dor or Toupet wrap). The top of the stomach is wrapped around the esophagus to create a barrier against acid reflux. This significantly reduces the risk of postoperative GERD.

POEM: Does not include a fundoplication. Because the procedure disrupts the natural anatomy differently, it has a higher rate of gastroesophageal reflux disease (GERD) afterward (typically 30-50 percent of patients develop abnormal acid exposure).

3. Length and Location of Myotomy

Heller: Usually limited to about 6-8 cm on the esophagus + 2-3 cm onto the stomach.

POEM: Allows a longer and more customizable myotomy (can easily extend 10-16+ cm up the esophagus). This makes POEM particularly advantageous for Type III (spastic) achalasia and certain cases of jackhammer esophagus.

4. Recovery and Invasiveness

POEM: Generally less invasive. Patients often go home the next day or even the same day in some centers. Less pain, no abdominal incisions.

Heller: Slightly longer hospital stay (usually 1-2 days). More postoperative pain due to abdominal incisions and manipulation of the stomach.

5. Success Rates and Durability

Both procedures have excellent short-term success rates (85-95 percent symptom relief).

Long-term data (5-10 years) shows they are roughly comparable, though some studies suggest POEM may have slightly higher rates of symptom recurrence after 5+ years in certain patients.

POEM appears superior for Type III achalasia; Heller with fundoplication may be preferable in patients at high risk for reflux (younger patients, those with pre-existing GERD, or hiatal hernia).

Summary Table

Feature Heller Myotomy POEM

Approach Laparoscopic surgery Endoscopic (mouth only)

Incisions 4-5 abdominal incisions None

Fundoplication Usually performed Not performed

GERD risk after procedure Lower (10-25 percent ) Higher (30-50 percent )

Best for Type III Good Often preferred

Myotomy length Shorter, more standardized Longer and customizable

Recovery Slightly longer Usually faster

Surgeon/Doctor Surgeon Advanced endoscopist (often GI)

Important Note: The best choice depends on your specific type of achalasia (Chicago classification), anatomy, age, reflux risk, and the expertise available at your center. Many high-volume centers now offer both and will recommend based on individual factors.

Would you like me to explain the potential complications of each, compare their long-term outcomes with recent studies, or discuss how doctors decide which procedure is best for a specific patient?

**Model Qwen3-Max**

Answer text:

POEM (Peroral Endoscopic Myotomy) and Heller myotomy are both surgical procedures used to treat achalasia, a disorder of the esophagus characterized by failure of the lower esophageal sphincter (LES) to relax properly, leading to difficulty swallowing.

Key Similarities:

Both aim to cut the muscle fibers of the LES to reduce pressure and improve esophageal emptying.

Both are considered myotomies-surgical incisions of muscle tissue.

Both are typically combined with a partial fundoplication (e.g., Dor or Toupet) to prevent gastroesophageal reflux after the procedure.

Differences:

Feature Heller Myotomy POEM

Approach Traditionally performed laparoscopically (through small abdominal incisions); can also be robotic. Performed endoscopically through the mouth-no external incisions.

Access Route Abdominal access to the esophagus and LES. Transoral access; scope enters through the mouth into the esophagus.

Invasiveness Minimally invasive but requires abdominal incisions and insufflation. Less invasive-no skin incisions; entirely internal.

Recovery Time Typically 1-2 days in hospital; full recovery in 1-2 weeks. Often shorter hospital stay (1 day or outpatient); quicker return to normal activity.

Scarring Small abdominal scars from trocar sites. No external scars.

Risk of Reflux about 10-20 percent risk of postoperative GERD (mitigated by adding fundoplication). Slightly higher risk of GERD (studies report 20-40 percent ), though debated; routine fundoplication not always performed.

Technical Complexity Well-established, standardized technique. Requires advanced endoscopic expertise; steeper learning curve.

Long-Term Data Decades of follow-up data supporting efficacy and safety. Shorter track record (introduced about 2008), but growing evidence shows comparable short- to mid-term outcomes.

Clinical Considerations:

POEM is often preferred for patients who want to avoid abdominal surgery or have had prior abdominal operations.

Heller myotomy remains the gold standard in many centers, especially when combined with an anti-reflux procedure.

Both achieve greater than 90 percent success in symptom relief in experienced hands.

Important Note: Post-procedure monitoring for gastroesophageal reflux disease (GERD) is crucial after both procedures, but especially after POEM due to potentially higher acid exposure.

Would you like a comparison of outcomes from recent clinical trials or guidance on how clinicians choose between the two?

**Q34. What is pneumatic dilation for achalasia?**

**Model ChatGPT 5.5**

Answer text:

Pneumatic dilation is a non-surgical treatment for achalasia, a swallowing disorder where the lower esophageal sphincter (LES) does not relax properly and the esophagus has trouble pushing food into the stomach.

In pneumatic dilation, a doctor passes an endoscope through the mouth into the esophagus and positions a special balloon across the tight LES. The balloon is then inflated with air to stretch and partially disrupt the sphincter muscle, making it easier for food and liquid to pass into the stomach.

What it's used for

It is used to relieve symptoms of achalasia such as:

Difficulty swallowing, especially solids and liquids

Regurgitation of food or fluid

Chest discomfort

Weight loss from poor intake

How it works

Achalasia is caused by failure of the LES to relax. Pneumatic dilation helps by weakening the overly tight sphincter, reducing the pressure barrier between the esophagus and stomach.

Effectiveness

Many patients improve after one dilation, but some need repeat dilations with progressively larger balloons. Symptom relief can last years, though achalasia can recur.

Main risks

The most important risk is esophageal perforation, meaning a tear in the esophagus. This is uncommon but serious and may require hospitalization, antibiotics, drainage, or surgery. Other possible risks include bleeding, chest pain, reflux, or need for repeat treatment.

Alternatives

Other treatments for achalasia include:

Laparoscopic Heller myotomy

POEM - peroral endoscopic myotomy

Botulinum toxin injection

Medications, though these are usually less effective

In short, pneumatic dilation is a balloon-stretching procedure designed to open the tight valve at the bottom of the esophagus and improve swallowing in achalasia.

A good next question to ask your gastroenterologist is: "Am I a good candidate for pneumatic dilation compared with POEM or Heller myotomy?"

**Model Claude Opus 4.7**

Answer text:

Pneumatic Dilation for Achalasia

Pneumatic dilation (PD) is a non-surgical endoscopic procedure used to treat achalasia, a disorder where the lower esophageal sphincter (LES) fails to relax properly, making swallowing difficult.

How It Works

A specialized air-filled balloon is positioned across the lower esophageal sphincter under endoscopic and/or fluoroscopic guidance. The balloon is then inflated to a controlled pressure, forcefully stretching and tearing the muscle fibers of the LES. This reduces the sphincter's resting pressure and allows food to pass more easily into the stomach.

The Procedure

Performed under sedation, typically as an outpatient procedure

A guidewire is placed through the LES

A polyethylene balloon (Rigiflex is most common) is advanced over the wire

Balloons come in graded sizes: 30 mm, 35 mm, and 40 mm

A "graded dilation" approach is often used, starting with the 30 mm balloon and stepping up if symptoms persist

Balloon is typically inflated for 15-60 seconds

Effectiveness

Short-term success: 70-90 percent of patients

Long-term: about 50-60 percent remain symptom-free at 5 years

Repeat dilations may be needed

Best results in patients over age 40 and with type II achalasia (per Chicago Classification)

Risks and Complications

Esophageal perforation (most serious): occurs in 1-4 percent of cases

Bleeding

Chest pain

Aspiration

Post-procedure gastroesophageal reflux ( about 15-35 percent )

Post-Procedure

Patients are often monitored, sometimes with a water-soluble contrast study (Gastrografin swallow) to rule out perforation

Gradual return to a normal diet

PPIs may be prescribed for reflux

Alternatives

Heller myotomy (surgical)

POEM (peroral endoscopic myotomy)

Botulinum toxin injection (for poor surgical candidates)

PD is considered comparable to surgical myotomy in efficacy for many patients and remains a well-established first-line treatment option.

Would you like more detail on any specific aspect, such as patient selection or comparison with POEM?

**Model DeepSeek V4 Pro**

Answer text:

Pneumatic dilation is a non-surgical, endoscopic procedure used to treat achalasia-a motility disorder where the lower esophageal sphincter (LES) fails to relax properly, making it difficult for food and liquids to pass into the stomach. The goal of the dilation is to forcefully stretch and partially tear the tight LES muscle fibers, reducing the obstruction and improving swallowing.

How It Works

Preparation - The patient is sedated, and an endoscope is passed through the mouth into the esophagus.

Balloon Placement - A specially designed balloon (typically 30-40 mm in diameter) is positioned across the LES, often under fluoroscopic (X-ray) guidance.

Inflation - The balloon is rapidly inflated with air to a high pressure for 30-60 seconds. This disrupts the circular muscle fibers without cutting.

Confirmation - The doctor may check for perforation immediately afterward (e.g., with a contrast swallow) and observe the patient for several hours.

Effectiveness & Considerations

Success rates - The first dilation provides good relief in 70-90 percent of patients, though symptoms may recur over time (often requiring repeat dilations).

Symptom improvement - Most patients experience significant and rapid improvement in dysphagia, regurgitation, and chest pain.

Compared to surgery - Heller myotomy (surgical cutting of the LES) and POEM (per-oral endoscopic myotomy) are more durable but invasive; pneumatic dilation is quicker, less expensive, and avoids surgery.

Risks & Potential Complications

Muscle perforation is the most serious risk. It occurs in about 1-3 percent of cases in experienced hands (up to 5 percent in earlier series). A small leak may heal with conservative management, but a large perforation requires emergency surgery. Reflux - After dilation, the LES may become too loose, leading to acid reflux (less common than after surgery). Bleeding, aspiration, or transient chest discomfort are also possible.

Optimization Tips (for clinicians considering the procedure)

Patient selection - Best suited for patients with type II achalasia (very good response) and type I achalasia. Type III (spastic) often responds less well, and surgery/POEM may be preferred.

Graded approach - Start with a smaller balloon (30 mm) and reassess; if symptoms persist, progressively larger balloons (35 mm, then 40 mm) in separate sessions are used to balance efficacy and perforation risk.

Post-procedure care - Observe for at least 2-4 hours, and do a water-soluble contrast study if there is any suspicion of perforation.

Follow-up - Periodic pH-metry and endoscopic surveillance may be needed if significant GERD develops.

Pneumatic dilation remains a valuable, minimally invasive option that can offer years of symptom relief without major surgery, but it requires a skilled operator and careful risk management.

Related question to continue the conversation: Are you curious how pneumatic dilation compares to POEM (per-oral endoscopic myotomy) in long-term outcomes and which patients might benefit most from one over the other?

**Model Gemini 3.1 Pro Thinking**

Answer text:

Pneumatic dilation is a widely used, minimally invasive endoscopic procedure designed to treat achalasia, a rare esophageal disorder.

In achalasia, the nerves in the esophagus are damaged, which prevents the esophagus from squeezing food down and causes the lower esophageal sphincter (LES)-the valve between the esophagus and the stomach-to fail to relax. This makes it very difficult for food and liquids to enter the stomach.

Here is a breakdown of how pneumatic dilation works, its effectiveness, and the associated risks.

How the Procedure Works

Preparation and Sedation: The patient is instructed to fast beforehand. The procedure is performed under intravenous sedation or light anesthesia to ensure comfort.

Placement: A gastroenterologist inserts an endoscope (a flexible tube with a camera) down the throat and into the esophagus. A specialized, deflated balloon is then passed through the mouth and carefully positioned exactly across the LES over a guidewire, often using X-ray guidance (fluoroscopy).

Dilation (Stretching): Once in place, the balloon is inflated forcefully to a specific pressure.

The Goal: The intense pressure from the balloon intentionally tears and stretches the tight muscle fibers of the LES. This permanently weakens the valve, allowing gravity to help food and liquid pass into the stomach.

Effectiveness

High Success Rate: It is the most effective non-surgical treatment for achalasia, providing significant symptom relief for about 70 percent to 90 percent of patients.

Durability: While effective, it is not always a permanent cure. Many patients require repeat dilations over time (often starting with a smaller balloon and moving to a larger one if symptoms return).

Potential Risks and Warnings

Esophageal Perforation (Tear): This is the most serious risk, occurring in about 2 percent to 5 percent of cases. A hole can form in the esophagus during the stretching. Small tears can sometimes be treated with clips and antibiotics, but larger perforations require emergency surgery.

Gastroesophageal Reflux Disease (GERD): Because the procedure destroys the valve that keeps stomach acid out of the esophagus, some patients develop acid reflux afterward and may need daily antacid medication (like PPIs).

Chest Pain and Minor Bleeding: Mild chest pain is common for a few days following the procedure, and minor bleeding at the tear site can occur.

Disclaimer: I am an AI, not a doctor. This information is for educational purposes. Any treatment decisions should be discussed thoroughly with a qualified gastroenterologist or surgeon.

Optimization and Alternative Options

If pneumatic dilation is not suitable or fails, there are other excellent, more modern interventions to consider:

POEM (Peroral Endoscopic Myotomy): An advanced endoscopic procedure where a tunnel is created in the esophageal lining to cut the LES muscle directly from the inside. It has excellent success rates and is becoming the gold standard in many centers.

Laparoscopic Heller Myotomy (LHM): A minimally invasive surgical procedure where the muscles of the LES are cut from the outside of the esophagus. This is often combined with a procedure to prevent acid reflux (fundoplication).

Botox Injections: Botulinum toxin can be injected into the LES to relax it. This is very safe but temporary (lasting 6-12 months) and is usually reserved for patients who are not healthy enough for dilation or surgery.

Would you like to know more about how pneumatic dilation directly compares to newer procedures like POEM, or are you looking for information on what the recovery process looks like?

**Model Grok-4.3**

Answer text:

Pneumatic dilation (also called balloon dilation or pneumatic dilatation) is a common, minimally invasive endoscopic procedure used to treat achalasia.

What is achalasia?

In achalasia, the lower esophageal sphincter (LES) - the muscular valve between the esophagus and stomach - fails to relax properly, and the normal wave-like contractions (peristalsis) of the esophagus are impaired. This creates a functional obstruction, causing difficulty swallowing, regurgitation, chest pain, weight loss, and sometimes aspiration.

How pneumatic dilation works

The goal is to disrupt the muscle fibers of the tight LES without surgery. A specially designed balloon is positioned across the sphincter and inflated with air (hence "pneumatic") under controlled pressure. The radial force of the expanding balloon tears some of the circular muscle fibers, permanently lowering the pressure at the LES so food and liquid can pass more easily into the stomach.

The procedure (step by step)

Performed under conscious sedation or monitored anesthesia care (rarely general anesthesia).

The doctor passes a flexible endoscope through the mouth into the esophagus.

A guidewire is usually placed, and then a Rigiflex or similar pneumatic balloon (typically 30 mm, 35 mm, or 40 mm diameter) is positioned so it straddles the LES, often using fluoroscopy (X-ray) for precise placement.

The balloon is gradually inflated to 8-15 psi (or until the "waist" disappears on imaging) and held for 15-60 seconds, usually repeated 2-3 times.

The patient is observed for several hours afterward to watch for complications.

Many centers use a graded approach - starting with a smaller 30 mm balloon and increasing size in subsequent sessions if needed.

Effectiveness

Initial success rate: 70-90 percent after one to three sessions.

Many patients require repeat dilations over the years (average durability is 1-5 years per dilation).

Better long-term results in older patients ( greater than 45-50 years) and those with milder esophageal dilation on imaging.

Less effective in younger patients and those with certain subtypes (especially Type III achalasia).

Risks and complications (important)

Esophageal perforation: The most serious risk, occurring in roughly 1-5 percent of procedures. Small perforations may be managed conservatively; larger ones may require surgery or stenting.

Bleeding.

Chest pain (common for 1-3 days afterward).

Gastroesophageal reflux disease (GERD) afterward in 20-40 percent of patients, because the sphincter is now weakened.

Aspiration during sedation.

Because of the perforation risk, the procedure is usually done in centers equipped for emergency surgical intervention.

Comparison to other treatments

Heller myotomy (laparoscopic or robotic) and POEM (Peroral Endoscopic Myotomy) generally offer more durable symptom relief than pneumatic dilation.

Botox injection is less invasive but much shorter-lived (usually 6-12 months) and mainly used for frail elderly patients.

Important disclaimer: This is general medical information. Treatment decisions depend on your age, achalasia subtype (determined by high-resolution manometry), esophageal anatomy, and overall health. Pneumatic dilation is not appropriate for every patient.

Would you like me to explain how it compares in detail to POEM or Heller myotomy, what the success rates look like by age/subtype, or what to expect during recovery?

**Model Qwen3-Max**

Answer text:

Pneumatic dilation (also known as pneumatic balloon dilation) is a non-surgical, endoscopic treatment for achalasia, a motility disorder of the esophagus characterized by failure of the lower esophageal sphincter (LES) to relax properly during swallowing and absent or abnormal peristalsis in the esophageal body.

How It Works:

During pneumatic dilation:

An endoscope is passed into the esophagus.

A deflated balloon catheter is positioned across the LES under fluoroscopic (X-ray) or endoscopic guidance.

The balloon is then inflated with air to a specific diameter (commonly 30-40 mm), which mechanically disrupts the muscle fibers of the LES.

This stretching and tearing reduces LES pressure, allowing food and liquids to pass more easily into the stomach.

Goals:

Improve esophageal emptying

Relieve symptoms like dysphagia (difficulty swallowing), regurgitation, chest pain, and weight loss

Efficacy:

Success rates range from 60 percent to 90 percent , depending on balloon size, technique, and patient factors.

Often provides symptom relief for months to years, though some patients may require repeat procedures.

Risks:

Esophageal perforation (most serious complication; occurs in about 1-5 percent of cases)

Gastroesophageal reflux (due to reduced LES pressure)

Bleeding or aspiration (rare)

Considerations:

Pneumatic dilation is often considered a first-line therapy alongside Heller myotomy (surgical) or peroral endoscopic myotomy (POEM).

It is generally avoided in young patients ( less than 40 years) if surgery is feasible, due to higher recurrence rates and potential need for repeated interventions.

Contraindicated in patients with significant comorbidities that increase perforation risk or in those with prior esophageal surgery.

Follow-up:

Patients are typically monitored with symptom assessment, barium swallow studies, or manometry to evaluate response and detect complications.

Would you like a comparison between pneumatic dilation, Heller myotomy, and POEM in terms of efficacy, risks, and recovery?

**Q35. Is pneumatic dilation safe?**

**Model ChatGPT 5.5**

Answer text:

Usually, yes-pneumatic dilation is considered a generally safe and established treatment for achalasia when performed by an experienced gastroenterologist/endoscopist using a graded balloon approach. It is not risk-free, though.

Main safety points

Most serious risk: esophageal perforation - a tear in the esophagus. Reported rates vary, but commonly are around 1-4 percent in many series; broader estimates can be higher depending on technique, patient factors, and definitions. A 2023 multicenter study found 6 perforations in 319 dilations - 1.9 percent - with no bleeding or deaths within 30 days. (pmc.ncbi.nlm.nih.gov)

Other short-term effects can include chest pain, transient discomfort, bleeding, aspiration, or need for ER evaluation. In the same study, non-perforation chest pain leading to ER referral occurred in 3.8 percent . (pmc.ncbi.nlm.nih.gov)

Effectiveness is good, but repeat treatments may be needed. A systematic review found remission rates around 79-81 percent after 30-35 mm dilation at 6 months, and higher success with graded dilation up to 40 mm. (onlinelibrary.wiley.com)

Guidelines support it as a real treatment option. The American College of Gastroenterology notes that pneumatic dilation, laparoscopic Heller myotomy, and POEM are comparable options for many patients with type I or II achalasia; POEM may be preferred for type III achalasia. (sysge.org)

Technique matters. European guidance recommends a graded protocol, typically starting with a 30-mm balloon, then 35 mm, then 40 mm only if needed, rather than starting with a large balloon. This is intended to improve safety. (esge.com)

When it may be higher risk

Risk can be higher if you have had prior esophageal surgery or dilation, significant esophageal distortion/"sigmoid" esophagus, severe inflammation, frailty, bleeding risk, or if the procedure is done in a low-volume setting. It also depends on the achalasia subtype and available alternatives.

Call urgently after dilation if you have:

Severe or worsening chest/upper abdominal pain, fever, shortness of breath, rapid heart rate, vomiting blood, black stools, trouble swallowing that suddenly worsens, or feeling very ill-these can signal perforation or another complication.

Bottom line: Pneumatic dilation is generally safe and commonly used, but the key risk is esophageal perforation. It is safest when done by an experienced team using a graded approach and with a clear plan for managing complications.

Are you asking because you're scheduled for pneumatic dilation for achalasia, or for a different type of esophageal narrowing/stricture?

**Model Claude Opus 4.7**

Answer text:

Pneumatic Dilation Safety

Pneumatic dilation (PD) is generally considered safe and effective, particularly for treating achalasia, but it does carry some risks. Here's a balanced overview:

Safety Profile

Overall: PD has been used for decades and is considered a first-line treatment for achalasia, with success rates of 70-90 percent for symptom relief.

Main Risks

Esophageal perforation - The most serious complication, occurring in approximately 1-3 percent of procedures

Bleeding - Usually minor

Chest pain - Common but typically temporary

Gastroesophageal reflux - Can develop after treatment (15-35 percent )

Aspiration - Rare

Esophageal hematoma or mucosal tears - Uncommon

Factors That Improve Safety

Experienced operator at a high-volume center

Graded dilation approach (starting with 30mm balloon, then 35mm if needed)

Younger patients ( less than 40) tend to have higher perforation risk but also better long-term outcomes with surgery, so age is considered

Proper patient selection

Post-procedure monitoring (often with contrast study to rule out perforation)

Who Should Avoid It?

Patients with epiphrenic diverticulum

Significant esophageal hernia

Prior esophageal surgery (relative contraindication)

Coagulopathy

Poor surgical candidates if perforation occurs (since emergency surgery may be needed)

Bottom Line

When performed by experienced gastroenterologists on appropriate candidates, pneumatic dilation is a reasonably safe procedure. The main concern-perforation-is rare but serious, which is why it should be done at facilities equipped to manage this complication.

Would you like more information about a specific aspect, such as how it compares to Heller myotomy or POEM?

**Model DeepSeek V4 Pro**

Answer text:

Pneumatic dilation is a well-established treatment for achalasia (and occasionally other esophageal motility disorders), but like any invasive procedure, its safety profile depends on multiple factors. Below I break down the risks, how they are managed in modern practice, and what optimization strategies make the procedure significantly safer.

Safety Profile: What the Data Shows

Primary Risk: Esophageal Perforation

The most serious complication is a full-thickness tear (perforation) of the esophageal wall at the lower esophageal sphincter.

Historical rates: 2-6 percent in older series using non-graduated balloons or less standardized techniques.

Modern rates: With current best practices (see below), reported perforation rates are less than 1 percent to about 1.9 percent in high-volume centers. Many perforations are small, contained, and can be managed conservatively or endoscopically; only a minority require surgical repair.

Other Notable Risks

Post-procedural chest pain - nearly universal but usually self-limited ( less than 24 hours).

Gastroesophageal reflux - occurs in up to 20-30 percent of patients after dilation because the procedure permanently weakens the lower esophageal sphincter; often manageable with proton pump inhibitors.

Aspiration pneumonia - rare when fasting and aspiration precautions are followed.

Minor mucosal tear/bleeding - common but rarely clinically significant.

Incomplete symptom relief / relapse - roughly 30-40 percent of patients require repeat dilation within 5 years; some eventually choose alternative treatments.

Mortality

Mortality directly attributable to pneumatic dilation is exceedingly rare ( less than 0.1 percent ) in modern series, almost always linked to a missed or mismanaged perforation.

Optimization Strategies: Making Pneumatic Dilation Safer

If you or a patient are considering the procedure, these evidence-based measures dramatically improve safety:

High-volume operator/center The perforation rate is inversely correlated with the number of procedures performed annually. Choose a gastroenterologist who regularly performs pneumatic dilations.

Graduated balloon technique Instead of a single large inflation, modern protocols use a graded approach (e.g., start with a 30 mm balloon, then escalate to 35 mm and 40 mm in subsequent sessions if needed). This reduces the risk of uncontrolled tearing.

Real-time fluoroscopic guidance The balloon is positioned across the esophagogastric junction under continuous X-ray. Inflation with contrast allows immediate detection of a confined leak ("waist" obliteration without spillage confirms safe dilation).

Routine post-dilation contrast study A water-soluble contrast swallow (e.g., Gastrografin) immediately after the procedure, or within 2-4 hours, can rule out perforation before oral intake is resumed.

Appropriate patient selection and preparation

Clear liquid diet 24-48 hours beforehand to empty the esophagus.

Strict fasting ( at least 8 hours) to prevent aspiration.

Anticoagulant/antiplatelet management as per guidelines.

Avoiding the procedure in patients with known epiphrenic diverticula or severe cardiopulmonary instability unless carefully planned.

Post-procedure monitoring Overnight observation, or at least a 4-6 hour monitored stay, allows early detection of pain escalation, tachycardia, or subcutaneous emphysema that might signal a perforation.

Comparative Safety: Pneumatic Dilation vs. Alternative Treatments

Procedure Perforation Risk Major Morbidity Long-term Reflux Re-intervention Rate

Pneumatic dilation (graded) less than 1-2 percent Low 20-30 percent about 30-40 percent at 5 years

Laparoscopic Heller myotomy (LHM) + fundoplication 0.1-0.5 percent Low (post-surgical) less than 10 percent about 10-20 percent at 5 years

Peroral endoscopic myotomy (POEM) less than 0.5 percent Low 30-50 percent (if no fundoplication) less than 10 percent at 2-3 years

Takeaway: Pneumatic dilation is slightly riskier in terms of immediate perforation than surgical myotomy, but it avoids the risks of general anesthesia and surgical complications. POEM offers a middle ground but often leads to more reflux. The safest choice is individualized.

Potential Risk Warning

If perforation is not recognized promptly, mediastinitis, sepsis, and prolonged hospitalization can occur. Hence, patients must be informed of the signs (severe pain, fever, difficulty breathing) and have access to emergency care for at least 24 hours post-procedure.

Pneumatic dilation is contraindicated in patients with certain anatomical abnormalities (e.g., large diverticula, previous radiation therapy to the chest) where the risk of perforation is unacceptably high.

Bottom Line

Yes, pneumatic dilation is safe when performed by an experienced team using a graduated, fluoroscopically guided technique with appropriate post-procedural checks. The risk of a serious complication is low and comparable to many other invasive interventions for achalasia. However, it is not a one-size-fits-all solution - the patient's age, surgical risk, and acceptance of potential repeat treatments play into the final decision.

To help you further: Are you comparing pneumatic dilation with POEM or surgical myotomy for a specific case, or would you like more detailed information on the recovery process and long-term outcomes?

**Model Gemini 3.1 Pro Thinking**

Answer text:

Disclaimer: I am an AI, not a doctor. The following information is for educational purposes and should not replace professional medical advice. Always consult with a qualified healthcare provider regarding medical procedures.

Generally, pneumatic dilation is considered a safe and highly effective minimally invasive procedure, most commonly used to treat achalasia (a disorder where the esophagus has difficulty moving food into the stomach). It involves passing a deflated balloon down the esophagus using an endoscope and then inflating it to stretch and weaken the tight lower esophageal sphincter.

While it is a standard and safe treatment, no medical procedure is entirely without risk.

Potential Risks and Complications

Esophageal Perforation: This is the most serious risk. It involves a tear in the esophagus caused by the stretching of the balloon. It occurs in roughly 1 percent to 5 percent of cases. If a perforation occurs, it is a medical emergency that may require immediate surgery to repair.

Gastroesophageal Reflux Disease (GERD): Because the procedure weakens the valve between the esophagus and stomach, acid can more easily wash back up. Many patients may need to take acid-suppressing medication (like PPIs) after the procedure.

Chest Pain: It is normal to experience some chest pain or discomfort for a few days following the stretching of the esophageal muscles.

Bleeding or Infection: As with any endoscopic procedure, there is a minor risk of bleeding or infection, though this is quite rare.

Need for Repeat Procedures: The effects of pneumatic dilation can wear off over time. Some patients may need the procedure repeated after a few years if symptoms return.

Optimization and Safety Best Practices

If you or someone you know is considering this procedure, here are some best practices to minimize risks:

Seek an Experienced Specialist: The safety and success of pneumatic dilation are highly dependent on the skill of the doctor. Seek out a high-volume medical center and a gastroenterologist who specializes in esophageal motility disorders.

Graded Dilation Approach: Best practices usually involve starting with a smaller balloon (e.g., 30mm) and only moving to larger sizes in subsequent sessions if the initial dilation doesn't provide enough symptom relief. This significantly reduces the risk of perforation.

Use of Imaging: Ensure the doctor uses fluoroscopy (real-time X-ray) or direct endoscopic visualization during the procedure to ensure the balloon is positioned perfectly across the sphincter before inflating.

Strict Fasting: Adhere strictly to the pre-procedure fasting guidelines (often a liquid diet for a day or two, followed by complete fasting) to ensure the esophagus is completely clear of retained food, reducing the risk of aspiration.

Follow-up question: Are you asking because you have been diagnosed with achalasia and are weighing pneumatic dilation against other surgical options like a Heller myotomy or POEM (Peroral Endoscopic Myotomy)?

**Model Grok-4.3**

Answer text:

Yes, pneumatic dilation is generally considered safe and effective when performed by an experienced gastroenterologist, but it is not risk-free.

What it is

Pneumatic dilation (also called balloon dilation) is a minimally invasive endoscopic procedure most commonly used to treat achalasia (and sometimes other esophageal motility disorders). A specially designed balloon is passed through the endoscope and inflated at the lower esophageal sphincter to disrupt the muscle fibers and improve swallowing.

Safety profile (evidence-based summary)

Overall safety: Large studies and meta-analyses show it is a low-mortality procedure (death rate less than 0.5 percent ). It is often used as a first-line therapy because it avoids surgery.

Major risk - esophageal perforation: Occurs in roughly 1-4 percent of cases (rates vary with balloon size, operator experience, and technique). Most perforations are small and can be managed conservatively with antibiotics, nil-by-mouth, and close monitoring; only a minority require surgery.

Other common side effects:

Chest pain (often transient, 30-50 percent of patients)

Gastroesophageal reflux disease (GERD) afterward (20-40 percent )

Bleeding (usually minor)

Need for repeat dilations (many patients require 2-3 sessions over time for lasting relief)

Success rates for symptom relief are typically 70-90 percent after one or more sessions, though durability is lower than surgical options (Heller myotomy or POEM) in younger patients or those with very high lower-esophageal-sphincter pressures.

Factors that improve safety

High-volume centers and experienced endoscopists (lower perforation rates)

Graduated balloon sizes (starting with 30 mm rather than jumping to 35 mm)

Use of fluoroscopy or modern controlled radial expansion balloons

Proper patient selection (not ideal for every subtype of achalasia or patients with prior esophageal surgery)

Important disclaimer

This is general medical information drawn from gastroenterology literature and guidelines (e.g., American College of Gastroenterology, European guidelines). Individual risk varies based on your age, anatomy, achalasia subtype, comorbidities, and the specific technique used. Pneumatic dilation is not appropriate for everyone, and only a qualified physician who has reviewed your case can determine if it is safe for you.

Always discuss this with a gastroenterologist who specializes in esophageal motility disorders. They can review your manometry, barium swallow, and endoscopy results and compare pneumatic dilation with alternatives such as POEM, Heller myotomy, or botox injections.

Would you like me to outline the typical risk-benefit comparison with the main alternative procedures (POEM vs. Heller myotomy), or are you looking for information on what to expect during recovery?

**Model Qwen3-Max**

Answer text:

Pneumatic dilation is generally considered a safe and effective treatment for certain esophageal motility disorders-most commonly achalasia-when performed by experienced clinicians. However, like any medical procedure, it carries potential risks and complications.

How It Works:

Pneumatic dilation involves inserting a balloon into the lower esophageal sphincter (LES) and inflating it to stretch and disrupt the muscle fibers, thereby improving esophageal emptying.

Safety Profile:

Success rate: Around 70-90 percent of patients experience significant symptom relief after one or more dilations.

Common side effects: Mild chest pain, transient gastroesophageal reflux.

Serious complication: The most concerning risk is esophageal perforation, which occurs in approximately 1-5 percent of cases, depending on technique, balloon size, and operator experience.

Risk Mitigation:

Performed under fluoroscopic guidance or with manometric control to improve accuracy.

Starting with smaller balloon sizes (e.g., 30 mm) and escalating as needed reduces perforation risk.

Patients are monitored closely post-procedure for signs of perforation (e.g., severe pain, fever, tachycardia).

Contraindications & Considerations:

Not recommended in patients with significant comorbidities that increase surgical risk if perforation occurs.

Alternative treatments include peroral endoscopic myotomy (POEM) or surgical Heller myotomy, especially in younger patients or those with recurrent symptoms.

Important: Pneumatic dilation should only be performed in centers equipped to manage complications, including immediate access to surgery in case of perforation.

If you're considering this procedure for yourself or someone else, discuss the risks and benefits with a gastroenterologist who specializes in motility disorders.

Would you like information comparing pneumatic dilation to other treatment options for achalasia?

**Q36. What is botulinum toxin injection for achalasia?**

**Model ChatGPT 5.5**

Answer text:

Botulinum toxin injection for achalasia is an endoscopic treatment in which a doctor injects botulinum toxin type A - often known by the brand name Botox - into the lower esophageal sphincter (LES), the tight muscle valve between the esophagus and stomach.

In achalasia, the LES does not relax properly and the esophagus loses normal pushing movements, causing symptoms such as trouble swallowing, regurgitation, chest discomfort, and weight loss. The injection weakens/relaxes the LES by blocking nerve signaling to the muscle, which can help food and liquid pass into the stomach more easily. (asge.org)

How it is done

It is usually performed during an upper endoscopy.

A needle is passed through the endoscope.

Botulinum toxin is injected into several spots around the LES, commonly totaling about 100 units, though technique can vary by clinician and setting. (cheshireandmerseysideformulary.nhs.uk)

It is typically an outpatient/day procedure with sedation.

Who is it for?

Botulinum toxin injection is generally not considered the most durable treatment for achalasia. Current guideline-based use is mainly for people who are not good candidates for more definitive therapies, such as:

Peroral endoscopic myotomy - POEM

Laparoscopic Heller myotomy

Pneumatic dilation

The American College of Gastroenterology recommends botulinum toxin injection as first-line therapy mainly for patients with achalasia who are unfit for definitive therapies. (sysge.org)

Benefits

Less invasive than surgery or pneumatic dilation.

Can provide relatively quick symptom relief.

Useful in older, frail, or medically high-risk patients.

Can sometimes be used as a temporary "bridge" if a more durable treatment needs to be delayed.

Limitations

The main downside is that the effect is usually temporary. Symptoms often return after months, and repeat injections may be needed. Some patients may get longer benefit, but recurrence is common. (cheshireandmerseysideformulary.nhs.uk)

Possible risks or side effects

Risks are generally low but can include:

Temporary chest discomfort or heartburn

Sore throat from endoscopy

Bleeding or infection, rarely

Allergic reaction, very rarely

Very rare spread of toxin effect causing weakness or swallowing/breathing problems

Also, repeated injections may cause scarring or inflammation around the LES, which can potentially make later definitive treatments more technically difficult.

Bottom line: Botulinum toxin injection for achalasia is a minimally invasive endoscopic treatment that relaxes the tight lower esophageal sphincter. It can improve swallowing, but it usually works for a limited time and is most often reserved for patients who cannot safely undergo longer-lasting treatments like POEM, Heller myotomy, or pneumatic dilation.

If you're asking because this was recommended to you, the key question to discuss with your gastroenterologist is: "Am I being offered Botox because I'm not a candidate for POEM, Heller myotomy, or pneumatic dilation, or is this meant as temporary relief?"

**Model Claude Opus 4.7**

Answer text:

Botulinum Toxin Injection for Achalasia

What is Achalasia?

Achalasia is a rare esophageal motility disorder where the lower esophageal sphincter (LES) fails to relax properly, and the esophagus loses its ability to move food downward. This causes difficulty swallowing, regurgitation, chest pain, and weight loss.

The Procedure

Botulinum toxin (Botox) injection is a minimally invasive treatment performed during an upper endoscopy:

Endoscopic approach: A flexible endoscope is passed through the mouth into the esophagus

Injection: Botulinum toxin (typically 80-100 units total) is injected into the LES using a sclerotherapy needle

Multiple sites: The toxin is delivered in 4-5 aliquots around the circumference of the sphincter

Duration: The procedure takes about 15-30 minutes and is usually done as an outpatient

How It Works

Botulinum toxin blocks the release of acetylcholine from nerve endings, which:

Reduces excessive muscle contraction of the LES

Allows easier passage of food into the stomach

Provides symptomatic relief

Effectiveness

Initial response: about 75-90 percent of patients experience improvement

Duration: Effects typically last 6-12 months

Repeat injections: Often required, but effectiveness may decrease with repeated treatments

Who Is It For?

Botox injection is generally reserved for:

Elderly or frail patients who are poor candidates for surgery

Patients with significant comorbidities

Those who decline or cannot tolerate definitive treatments

As a temporary bridge to more definitive therapy

Comparison to Other Treatments

More definitive treatments with longer-lasting results include:

Pneumatic dilation

Heller myotomy (surgical)

POEM (peroral endoscopic myotomy)

Advantages

Minimally invasive

Low complication rate

Can be done as outpatient

Useful for high-risk surgical patients

Disadvantages

Temporary effect requiring repeat treatments

May cause submucosal scarring, making future myotomy more difficult

Diminishing returns with repeated injections

Less effective than definitive treatments

Side Effects

Generally well-tolerated, but possible effects include:

Chest pain (most common)

Heartburn

Rare: mediastinitis, pneumothorax

Would you like more information about any specific aspect of this treatment or alternative achalasia therapies?

**Model DeepSeek V4 Pro**

Answer text:

Botulinum toxin (Botox ) injection for achalasia is an endoscopic treatment that temporarily relaxes the tight lower esophageal sphincter (LES) to improve swallowing.

What is achalasia?

Achalasia is a rare motility disorder where the nerves in the esophagus degenerate, leading to two problems:

The LES fails to relax properly when you swallow.

The esophageal body loses normal peristalsis (coordinated squeezing).

As a result, food and liquid get stuck, causing dysphagia (difficulty swallowing), regurgitation, chest pain, and weight loss.

How does botulinum toxin help?

Botulinum toxin type A works by blocking the release of acetylcholine at nerve endings. In achalasia, the LES is in a state of high resting pressure because of unopposed excitatory nerve signals. When Botox is injected directly into the LES muscle (via an endoscope), it causes a temporary chemical denervation - the sphincter muscle relaxes and pressure drops, allowing swallowed material to pass more easily into the stomach.

The procedure

Done during a standard upper endoscopy, usually with sedation.

A sclerotherapy needle is passed through the scope, and multiple small injections (typically 80-100 units total, sometimes more) are placed in all four quadrants of the LES.

The entire process takes about 15-30 minutes; it's an outpatient procedure.

Effectiveness

Rapid relief: Most patients (70-90 percent ) experience significant improvement in dysphagia within a few days.

Duration is limited: The effect is not permanent. Half of initial responders see symptoms return within 6-12 months. Repeat injections are possible, but the success rate often declines, and the interval between treatments tends to shrink.

Not a cure: Botox does not address the underlying nerve loss or restore esophageal peristalsis; it only relieves the obstruction temporarily.

Who is typically offered Botox?

Because of its temporary nature, Botox is generally reserved for:

Elderly or frail patients who are poor surgical candidates for a myotomy (Heller or POEM).

Patients with co-morbidities that make surgery too risky.

As a bridge therapy to relieve symptoms while awaiting more definitive treatment.

Sometimes as a diagnostic trial when the diagnosis of achalasia vs. pseudo-achalasia is uncertain (but that's less common now with advanced imaging).

Risks and downsides

Mild post-procedure chest discomfort is common.

Rare risks: bleeding, infection, esophageal perforation (very rare with injection compared to dilation/surgery).

Fibrosis at the injection site: Repeated Botox injections can cause scarring and fibrosis in the submucosa and muscular layers. This can make future Heller myotomy or POEM more difficult and increase the risk of operative complications. Therefore, many surgeons recommend limiting Botox to one or two sessions, especially in patients who might later undergo surgery.

Reflux can worsen after the LES is relaxed, but it's usually mild and manageable.

Not all patients respond; non-responders are more likely to have vigorous achalasia (type III) or long-standing disease.

How does it compare to other treatments?

Pneumatic dilation: More durable than Botox (remission measured in years, not months), but carries a slightly higher risk of perforation.

Laparoscopic Heller myotomy (often with partial fundoplication): Offers long-term relief ( greater than 10 years) and is the surgical gold standard for suitable patients.

POEM (peroral endoscopic myotomy): An incisionless, endoscopic version of myotomy with excellent outcomes; increasingly popular.

Botox is the least invasive and quickest but the least durable.

Key takeaway

Botulinum toxin injection for achalasia is a valuable, low-risk palliative tool for patients who cannot undergo definitive therapy. It provides temporary relief by paralyzing the LES, but it does not halt the disease progression, and repeated use may complicate future surgical options.

Would you like to know more about how pneumatic dilation works, or about the step-by-step differences between Heller myotomy and POEM? I can walk you through those in detail.

**Model Gemini 3.1 Pro Thinking**

Answer text:

Botulinum toxin (often known by the brand name Botox) injection is a minimally invasive medical procedure used to treat achalasia, a rare disorder that makes it difficult for food and liquid to pass from your esophagus into your stomach.

Here is a detailed breakdown of how it works, who it is for, and the associated risks.

1. How It Works

In a person with achalasia, the ring of muscle at the bottom of the esophagus-called the lower esophageal sphincter (LES)-fails to relax during swallowing. Furthermore, the normal muscle contractions of the esophagus (peristalsis) are lost.

Botulinum toxin is a nerve-blocking agent. When injected directly into the LES, it blocks the signals from the nerves that tell the muscle to contract. This temporarily paralyzes and relaxes the sphincter, allowing gravity to help food and liquids pass into the stomach.

2. How the Procedure is Performed

Method: The procedure is performed during an upper endoscopy.

Process: The patient is usually given a mild sedative. A gastroenterologist guides a flexible tube with a camera (endoscope) down the throat and into the esophagus. A specialized needle is then passed through the endoscope to inject the Botox directly into several areas of the LES muscle.

Recovery: It is an outpatient procedure, meaning patients usually go home the same day and recover very quickly.

3. Who is the Ideal Candidate?

Botox is generally not considered the first-line treatment for most people with achalasia. Because its effects are temporary, it is usually reserved for:

Elderly patients.

Patients with complex medical conditions that make them poor candidates for surgery (such as Heller myotomy) or more invasive endoscopic procedures (like POEM - Peroral Endoscopic Myotomy).

Patients who need temporary relief while awaiting a more permanent surgical treatment.

4. Benefits vs. Limitations

Benefits: It is highly effective in the short term (relieving symptoms in about 70 percent to 90 percent of patients initially), minimally invasive, and carries a very low immediate complication rate.

Limitations: The effects wear off. Symptoms usually return within 6 to 12 months, requiring repeated injections.

Potential Risks and Medical Considerations

While generally safe, there are important risks and long-term considerations to keep in mind:

Scarring (Fibrosis): This is the most significant drawback. Repeated Botox injections cause inflammation and scarring in the esophageal muscle. This scarring can make future, more permanent surgeries (like Heller myotomy) much more difficult to perform and less successful.

Side Effects: Mild, temporary chest pain is common after the procedure.

Acid Reflux: Because the valve is artificially held open, stomach acid can wash back up into the esophagus (GERD).

Allergic Reaction: Though very rare, there is a small risk of an allergic reaction to the botulinum toxin.

Disclaimer: I am an AI, not a doctor. This information is for educational purposes. Always consult with a gastroenterologist or a thoracic surgeon to determine the best and safest treatment plan for achalasia based on individual health profiles.

Would you like to know how Botox injections compare to more permanent treatments for achalasia, such as the POEM procedure or Heller myotomy?

**Model Grok-4.3**

Answer text:

Botulinum toxin injection for achalasia is a minimally invasive endoscopic treatment that uses Botox (onabotulinumtoxinA) to temporarily relax the lower esophageal sphincter (LES).

What is Achalasia?

Achalasia is a primary esophageal motility disorder in which the LES fails to relax properly during swallowing and the esophageal body loses its normal peristaltic (propulsive) contractions. This causes food and liquid to accumulate above the sphincter, leading to progressive dysphagia (difficulty swallowing), regurgitation, chest pain, weight loss, and increased risk of aspiration.

How Botulinum Toxin Works

Botulinum toxin blocks the release of acetylcholine from excitatory neurons in the myenteric plexus of the esophagus. In achalasia, there is an imbalance-loss of inhibitory neurons (which normally release nitric oxide and VIP to relax the LES) while excitatory (cholinergic) input remains relatively unopposed. By inhibiting acetylcholine, Botox reduces LES pressure and improves esophageal emptying for a limited time.

The Procedure

Performed during a standard upper endoscopy (EGD) under conscious sedation.

The doctor injects 80-100 units of Botox in 4-5 aliquots circumferentially into the LES (usually at the squamocolumnar junction, sometimes guided by prior manometry or endoscopic ultrasound).

The entire procedure typically takes 10-15 minutes.

Patients usually go home the same day.

Effectiveness and Duration

Initial response rate: Approximately 60-85 percent of patients experience symptomatic improvement within 1-2 weeks.

Duration: Effects typically last 3-6 months, sometimes up to 12 months in a minority of patients. Most people require repeat injections.

Long-term efficacy declines with repeated use because the body can develop antibodies to the toxin and fibrosis may occur.

Because the benefit is temporary, Botox injection is generally considered a bridge therapy or reserved for specific populations rather than first-line treatment for healthy patients.

Current Guidelines (AGA, ASGE, ESGE)

Botox is primarily recommended for:

Elderly or frail patients with significant comorbidities who are poor candidates for more definitive therapies.

Patients who want to avoid the risks of pneumatic dilation or surgery.

As a diagnostic trial in atypical or uncertain cases.

Preferred long-term treatments for most patients are:

Pneumatic dilation

Laparoscopic Heller myotomy with partial fundoplication

Peroral endoscopic myotomy (POEM) - currently considered the most effective endoscopic option

Risks and Side Effects

Common: Transient chest pain or heartburn (most frequent), mild reflux.

Less common: Esophageal perforation ( less than 1 percent ), mediastinitis, allergic reaction to Botox, temporary weakness of nearby muscles.

Repeated injections can make future Heller myotomy or POEM technically more difficult due to scarring.

Important: This is general medical information. Treatment decisions must be individualized based on high-resolution manometry results (Chicago Classification), patient age, comorbidities, and local expertise. A gastroenterologist or esophageal motility specialist should evaluate anyone with suspected achalasia.

Would you like me to explain how botulinum toxin injection compares with pneumatic dilation, Heller myotomy, and POEM in terms of success rates, durability, and risks?

**Model Qwen3-Max**

Answer text:

Botulinum toxin injection for achalasia is a minimally invasive therapeutic procedure used to help relieve symptoms of achalasia, a rare esophageal motility disorder characterized by:

Failure of the lower esophageal sphincter (LES) to relax during swallowing

Absent or impaired peristalsis (coordinated muscle contractions) in the esophageal body

This leads to difficulty swallowing (dysphagia), regurgitation, chest pain, and sometimes weight loss.

How Botulinum Toxin Works in Achalasia

Botulinum toxin (commonly known by the brand name Botox ) is a neurotoxin that blocks the release of acetylcholine from nerve endings. When injected into the LES during an upper endoscopy (esophagogastroduodenoscopy, or EGD), it temporarily paralyzes the muscles of the sphincter, reducing its resting pressure and allowing food and liquids to pass more easily into the stomach.

Procedure Overview

Performed during endoscopy

Typically 4 injections (100 units total) are administered into the LES muscle at 90-degree intervals

Done under sedation

Outpatient procedure with rapid recovery

Efficacy and Duration

Provides short- to medium-term relief (typically 3-12 months)

Best suited for older patients, those with significant comorbidities, or individuals who are poor surgical candidates

Less effective long-term compared to pneumatic dilation or Heller myotomy

Advantages

Minimally invasive

Low risk of complications

Quick recovery

Can be repeated if needed

Limitations & Risks

Temporary effect: Symptoms often recur within a year

Repeated injections may lead to fibrosis of the LES, potentially complicating future definitive treatments like surgery

Rare complications include aspiration pneumonia, bleeding, or perforation (very uncommon)

Comparison with Other Treatments

Treatment Durability Invasiveness Best For

Botulinum toxin Short-term Low Elderly, high-risk patients

Pneumatic dilation Medium-long Moderate Most patients (except young women due to perforation risk)

Heller myotomy (surgical or laparoscopic) Long-term High Younger, healthy patients

POEM (Peroral Endoscopic Myotomy) Long-term Moderate-high Increasingly preferred minimally invasive surgical option

Summary

Botulinum toxin injection is a palliative, non-curative option for achalasia that offers quick symptom relief with minimal risk. While not a long-term solution, it plays an important role in managing patients who aren't candidates for more definitive therapies.

Would you like to know how this compares to newer treatments like POEM, or whether insurance typically covers Botox injections for achalasia?

**Q37. Why do I still have swallowing difficulty after achalasia treatment?**

**Model ChatGPT 5.5**

Answer text:

Swallowing difficulty after achalasia treatment is not unusual, but it should be evaluated-especially if it is persistent, worsening, or associated with weight loss, vomiting/regurgitation, chest pain, or aspiration/coughing at night.

Common reasons include:

The treatment didn't fully open the lower esophageal sphincter

After pneumatic dilation, Heller myotomy, POEM, or Botox, the lower esophageal sphincter/EGJ may still be too tight because of incomplete myotomy/dilation, scarring, or recurrence of outflow obstruction.

Achalasia affects the esophageal muscle, not just the sphincter

Even when the sphincter is adequately treated, the esophagus often still has poor or absent peristalsis, so food may move slowly and "hang up." This can feel like ongoing dysphagia despite technically successful treatment.

Post-treatment reflux or inflammation

Treatments that loosen the sphincter can cause GERD, especially after POEM. Reflux can cause esophagitis, swelling, or eventually a peptic stricture, all of which can make swallowing difficult. ACG guidance notes that endoscopy is useful after treatment when symptoms recur to assess reflux-related stricturing versus recurrent achalasia. (europepmc.org)

Mechanical narrowing or obstruction

Possibilities include a tight fundoplication after Heller myotomy, scar tissue, narrowing at the gastroesophageal junction, food impaction, or-less commonly-a separate condition such as eosinophilic esophagitis, ring/web, or tumor. Endoscopy is typically used to look for these.

Advanced or dilated "sigmoid" esophagus

Long-standing achalasia can leave the esophagus enlarged, tortuous, or poorly emptying even after the outlet is treated. In that situation, symptoms may come from retained food/fluid and poor clearance, not only sphincter tightness.

Spasm or achalasia subtype issues

Type III/spastic achalasia can be harder to treat if the myotomy did not extend far enough up the esophagus. Persistent chest pain plus dysphagia can point toward ongoing spasm.

Normal early recovery vs recurrence

Mild difficulty can happen shortly after treatment due to swelling, diet advancement, or healing. But dysphagia that continues beyond the expected recovery window, returns after improvement, or progressively worsens deserves reassessment.

Tests your GI doctor may consider include:

Upper endoscopy to check for esophagitis, stricture, food retention, tight wrap, or other obstruction.

Timed barium esophagram to measure how well the esophagus empties after treatment; it is commonly used to evaluate treated achalasia. (radiopaedia.org)

High-resolution manometry to see whether the EGJ is still obstructed or whether another motility pattern is present.

FLIP during endoscopy in some centers to measure EGJ distensibility; recent reviews describe FLIP, manometry, barium study, and endoscopy as complementary tools for recurrent dysphagia after myotomy. (pmc.ncbi.nlm.nih.gov)

pH testing if reflux is suspected.

Treatment depends on the cause and may include acid suppression, dilation of a stricture, repeat pneumatic dilation, redo POEM/Heller myotomy, loosening/revision of a tight fundoplication, or management of end-stage esophageal emptying problems. Recurrent dysphagia after myotomy is considered multifactorial, with causes including incomplete myotomy, fundoplication-related obstruction, esophageal body abnormalities, dysmotility, and GERD. (pmc.ncbi.nlm.nih.gov)

Seek urgent care if you cannot keep liquids down, have severe chest pain, black/bloody vomiting, fever, signs of aspiration pneumonia, rapid weight loss, or dehydration.

A helpful next step is to tell your gastroenterologist: what treatment you had-POEM, Heller myotomy, pneumatic dilation, Botox-when it was done, and whether your swallowing ever improved before symptoms returned. That timing often narrows the cause.

**Model Claude Opus 4.7**

Answer text:

Why Swallowing Difficulty Persists After Achalasia Treatment

Persistent or recurrent dysphagia after achalasia treatment is relatively common and can result from several causes:

Common Causes

1. Incomplete initial treatment

Pneumatic dilation may not have sufficiently disrupted the lower esophageal sphincter (LES)

Heller myotomy may have been too short or incomplete

POEM (peroral endoscopic myotomy) may not have adequately divided muscle fibers

2. Disease progression

Achalasia is a chronic, progressive condition

The esophagus may become increasingly dilated and lose contractile function (end-stage "sigmoid" esophagus)

Food empties poorly even with an open LES due to gravity dependence

3. Scarring or fibrosis

Healing at the treatment site can re-narrow the LES

More common after repeated dilations

4. Reflux-related complications

Post-treatment GERD can cause peptic strictures

Barrett's esophagus or inflammation may contribute

5. Subtype of achalasia

Type III (spastic) achalasia has worse outcomes

Type II generally responds best

6. Misdiagnosis or coexisting conditions

Pseudoachalasia (from tumors at the GE junction)

Esophageal cancer (slightly increased risk in achalasia)

Eosinophilic esophagitis

What to Do Next

You should return to your gastroenterologist for evaluation, which typically includes:

Timed barium esophagram - measures esophageal emptying

High-resolution manometry - assesses LES pressure and motility

Upper endoscopy - rules out stricture, cancer, or inflammation

Eckardt score assessment to quantify symptoms

Treatment Options for Recurrent Symptoms

Repeat pneumatic dilation

POEM (especially effective after failed myotomy)

Redo Heller myotomy

Botulinum toxin injection (for poor surgical candidates)

Esophagectomy (reserved for end-stage disease)

Important: New or worsening dysphagia-especially with weight loss, bleeding, or symptoms appearing years after successful treatment-warrants prompt evaluation to rule out malignancy.

Would you like more detail about any specific aspect, such as a particular treatment you've had?

**Model DeepSeek V4 Pro**

Answer text:

I'm sorry you're dealing with persistent swallowing difficulty-it must be frustrating after going through treatment. While I'm an AI assistant and not a doctor, I can share some general reasons why dysphagia can continue after achalasia treatment, which might help you discuss things with your specialist.

Achalasia treatments (like pneumatic dilation, Heller myotomy, or POEM) aim to loosen the tight lower esophageal sphincter, but swallowing is a complex process. Even when the sphincter opens better, other factors can keep causing symptoms:

Possible reasons for ongoing dysphagia:

Incomplete relief of obstruction - The sphincter may not be sufficiently loosened, or scarring can cause re-narrowing over time.

Esophageal muscle failure - In advanced achalasia the esophageal body loses its squeezing ability permanently, so food may still "fall" into the stomach slowly even if the sphincter is open.

Acid reflux (GERD) - Treatments that loosen the sphincter can cause acid to flow back, leading to inflammation, strictures, or a reflux sensitivity that mimics dysphagia.

Tight fundoplication - If a fundoplication (wrap) was added during myotomy to prevent reflux, it may be too tight and obstruct swallowing.

Development of a new problem - Conditions like eosinophilic esophagitis, Candida infection, or even a small diverticulum can complicate the picture.

Functional overlap - Some people have hyper-sensitivity or motility issues in the remaining esophagus that were masked before treatment.

Mechanical issues - In rare cases, poor bolus clearance can lead to a non-emptying, dilated esophagus (megaesophagus) that still feels like fullness or blockage.

Risk awareness and optimization

Ongoing dysphagia should always be reassessed by a gastroenterologist or surgeon. Tests like a timed barium swallow, high-resolution manometry, or upper endoscopy can pinpoint the cause.

If reflux is the culprit, acid suppression (PPIs) and lifestyle changes are key; a pH study may be needed.

Re-intervention (repeat dilation, myotomy, or even Botox in select cases) might be an option, but only with careful workup.

A practical step: Keep a food-symptom diary-note what textures cause problems, whether liquids are easier, and any heartburn/regurgitation. This can help your doctor narrow the cause.

Have you had any follow-up testing since your treatment, such as a barium swallow or manometry? Understanding those results is often the first step to finding the right fix.

**Model Gemini 3.1 Pro Thinking**

Answer text:

Disclaimer: I am an AI, not a doctor. The information provided below is for educational purposes and should not replace professional medical advice. Please consult your gastroenterologist or surgeon for a proper evaluation of your symptoms.

It is incredibly frustrating to go through treatment for achalasia and still experience dysphagia (difficulty swallowing). However, it is actually quite common. Treatments for achalasia-such as a Heller myotomy, POEM (Peroral Endoscopic Myotomy), or pneumatic dilation-aim to relieve symptoms, but they are not a "cure."

Here are the most common medical and physiological reasons why you might still be experiencing swallowing difficulties after treatment:

1. The Esophagus Still Cannot Squeeze (Lack of Peristalsis)

Achalasia does two things: it stops the lower esophageal sphincter (LES) from relaxing, and it destroys the nerves that cause the esophagus to squeeze food downward (peristalsis). Treatment only fixes the sphincter. It opens the valve at the bottom, but your esophagus still cannot push food down. You are now entirely reliant on gravity and drinking liquids to wash food into your stomach. If you eat too quickly, eat dry foods, or lie down while eating, food will still get stuck.

2. Acid Reflux and Stricture Formation (GERD)

By cutting or stretching the LES to let food into the stomach, the natural barrier that keeps stomach acid out of the esophagus is compromised.

Many patients develop severe acid reflux after achalasia treatment.

Over time, chronic stomach acid exposure damages the lower esophagus, leading to inflammation and the formation of scar tissue (a peptic stricture). This scar tissue narrows the esophagus, bringing back the dysphagia.

3. Incomplete Myotomy or Dilation

Sometimes, the initial treatment wasn't quite enough. If a Heller myotomy or POEM did not cut far enough up the esophagus or far enough down into the stomach, the valve may still be too tight. If you had pneumatic dilation, the muscle may not have been stretched sufficiently, or it may have tightened back up. If you had Botox injections, the effects typically wear off after 3 to 6 months.

4. Scarring from the Procedure

Any surgical or endoscopic procedure causes the body to heal by creating scar tissue. In some cases, the scar tissue at the site of the myotomy can tighten as it heals, creating a new physical blockage.

5. Progression to "Megaesophagus"

If you had achalasia for a long time before being treated, your esophagus may have become severely dilated and stretched out, taking on a sigmoid (S-like) shape. Even if the valve at the bottom is wide open, a severely stretched, floppy esophagus acts like a sink with a trap-food simply pools at the bottom and cannot drain into the stomach.

6. Esophageal Spasms (Type III Achalasia)

If you have Type III (spastic) achalasia, your esophagus experiences uncoordinated, forceful contractions. If the myotomy (the muscle cut) wasn't made long enough up the chest to cover the spastic portion of the esophagus, those spasms can continue to cause chest pain and trap food.

Potential Risks & Next Steps

Risk Warning: Persistent food impaction can lead to aspiration (inhaling food/liquid into the lungs, causing pneumonia), malnutrition, and severe weight loss. If you are entirely unable to swallow liquids or your own saliva, seek emergency medical care.

What you should do next: You need to contact your gastroenterologist or motility specialist. They will likely recommend repeating one or more of the following tests to find the exact cause:

Barium Swallow (Esophagram): To see the shape of the esophagus and how quickly a liquid empties into the stomach.

Upper Endoscopy (EGD): To look for scar tissue, strictures, or signs of acid reflux.

High-Resolution Manometry: To measure the pressures in your esophagus to see if the sphincter is too tight or if you are having spasms.

To help me provide more tailored information, could you share what specific type of treatment you received (e.g., POEM, Heller myotomy, dilation) and how long ago it was performed?

**Model Grok-4.3**

Answer text:

Persistent swallowing difficulty after achalasia treatment is common and has several well-documented explanations.

Achalasia is a primary esophageal motility disorder characterized by two main problems: (1) failure of the lower esophageal sphincter (LES) to relax properly, and (2) absent or severely impaired peristalsis (the wave-like contractions that push food down) in the esophageal body. Current treatments (pneumatic dilation, Heller myotomy, POEM, or Botox) only address the first problem - they reduce pressure at the LES. They do not restore normal peristalsis in the esophageal body. This is the most frequent reason patients continue to have dysphagia even when the procedure was technically successful.

Common Reasons for Ongoing Symptoms

Here are the main causes, ranked roughly from most to least common based on clinical studies:

Residual esophageal dysmotility / poor propulsion The esophagus often remains "floppy" or aperistaltic. Food and liquid rely on gravity rather than muscular contractions. This frequently causes sticking, especially with solids, bread, meat, or pills. Many patients describe "food sitting in the chest" even when the LES is adequately opened.

Incomplete disruption of the LES The myotomy or dilation may not have been wide or long enough, or scar tissue can cause partial re-narrowing. This is more common after balloon dilation than after surgical myotomy or POEM.

Gastroesophageal reflux disease (GERD) and its complications Once the LES is opened, acid reflux becomes common (especially after myotomy or POEM without an adequate anti-reflux procedure). Chronic reflux can cause:

Esophagitis

Peptic strictures (scarred narrowing higher in the esophagus)

Motility worsening over time

Anatomic changes

Dilated, sigmoid-shaped esophagus (advanced disease) that empties poorly even with an open LES.

Twisted or angulated esophagus.

Hiatal hernia that develops or worsens after treatment.

Other less common causes

Scar tissue / fibrosis at the myotomy site.

Eosinophilic esophagitis (EoE) coexisting with achalasia.

Very rarely, pseudoachalasia (missed malignancy or other infiltrative process).

Functional dysphagia or learned maladaptive swallowing behaviors after years of symptoms.

What Should You Do Next?

Persistent dysphagia after treatment is not normal and deserves formal re-evaluation. Most specialists will order:

Timed barium esophagram (best test to assess emptying and anatomy).

Upper endoscopy (to look for strictures, reflux esophagitis, or retained food).

High-resolution esophageal manometry (to measure current LES pressure and peristalsis).

Sometimes pH-impedance testing if reflux is suspected.

Treatment options if the cause is identified can include:

Repeat dilation or Botox.

Revision myotomy or conversion to a different procedure.

Management of reflux (PPI medications, lifestyle changes, or anti-reflux surgery).

Esophageal dilation for strictures.

In severe refractory cases, esophagectomy (rare).

Important: I am an AI, not a physician. The above is general medical information drawn from gastroenterology literature (Chicago Classification, AGA guidelines, and long-term outcome studies on POEM/myotomy). Your specific situation requires individualized assessment by your gastroenterologist or esophageal motility specialist.

Recommended next step: Contact the doctor who performed your original procedure and request a timed barium swallow study. This is usually the most informative first test.

If you're comfortable sharing, what treatment did you have (POEM, Heller myotomy, dilation, Botox), how long ago, and do you have more trouble with solids, liquids, or both? That context can help me provide more targeted general information while you arrange proper medical follow-up.

**Model Qwen3-Max**

Answer text:

Persistent or recurrent swallowing difficulty (dysphagia) after treatment for achalasia is not uncommon and can occur for several reasons. Achalasia is a motility disorder of the esophagus characterized by failure of the lower esophageal sphincter (LES) to relax and impaired esophageal peristalsis. While treatments aim to reduce LES pressure and improve esophageal emptying, they don't restore normal motility-and sometimes complications or incomplete responses arise.

Here are common reasons why dysphagia may persist or return after treatment:

1. Incomplete Myotomy or Dilation

Pneumatic dilation or Heller myotomy (surgical or laparoscopic) may not fully disrupt the LES muscle, leaving residual obstruction.

Inadequate length or depth of myotomy (especially if it doesn't extend sufficiently onto the gastric side) can limit effectiveness.

2. Fibrosis or Scarring

After surgery or dilation, scar tissue can form at the gastroesophageal junction, leading to stricture-like narrowing and recurrent dysphagia.

3. Advanced Disease / "Burned-Out" Esophagus

In long-standing or severe achalasia (especially Type III/spastic or end-stage), the esophagus may become massively dilated and aperistaltic ("sigmoid esophagus"). Even after successful LES reduction, poor clearance due to absent motility can cause food retention and perceived dysphagia.

4. Reflux-Induced Stricture

Treatments like myotomy reduce LES pressure, which can lead to gastroesophageal reflux disease (GERD). Chronic acid exposure may cause peptic stricture, mimicking or worsening dysphagia.

5. Misdiagnosis or Overlapping Conditions

Sometimes symptoms attributed to achalasia may actually stem from eosinophilic esophagitis (EoE), esophageal cancer, or scleroderma, which can mimic achalasia on initial testing (so-called "achalasia mimics").

High-resolution manometry (HRM) and careful endoscopic evaluation are critical for accurate diagnosis.

6. Post-Treatment Edema or Inflammation

Shortly after procedures (e.g., POEM or dilation), temporary swelling can cause transient dysphagia that resolves in days to weeks.

What Should You Do?

If you're experiencing ongoing dysphagia after treatment:

Follow up with your gastroenterologist or esophageal specialist.

Consider repeat high-resolution manometry, barium esophagram, or endoscopy to assess anatomy, motility, and rule out strictures or other issues.

Timed barium swallow is particularly useful to evaluate esophageal emptying post-treatment.

If reflux is suspected, pH monitoring or a trial of proton pump inhibitors (PPIs) may help.

Important: New or worsening dysphagia after treatment should always be evaluated-especially to rule out malignancy or complications.

Would you like help interpreting specific test results or understanding which next diagnostic step might be most appropriate for your situation?

**Q38. What symptoms after POEM or surgery should make me seek urgent medical care?**

**Model ChatGPT 5.5**

Answer text:

After POEM (peroral endoscopic myotomy), Heller myotomy, or other esophageal/upper GI surgery, seek urgent medical care now-or call your surgeon's on-call number immediately-if you develop any of the following.

Call 911 / go to the ER immediately if you have

Chest pain, especially severe, worsening, crushing, or associated with sweating, dizziness, or nausea.

Trouble breathing, shortness of breath, wheezing, or coughing up blood.

Fainting, severe weakness, confusion, or feeling like you might pass out.

Fast heart rate with fever or severe pain, which can signal infection, leak, bleeding, or a clot.

Severe abdominal, chest, shoulder, or back pain that is new or getting worse.

Vomiting blood, or black/tarry or bloody stools.

Inability to swallow liquids, drooling, choking, or persistent vomiting.

Call your surgeon/doctor urgently the same day if you have

Fever at least 100.4 F / 38 C, chills, or feeling progressively ill.

Increasing abdominal swelling, a hard/tender belly, or pain that doesn't improve with prescribed medicine.

Persistent nausea/vomiting, inability to keep fluids down, or signs of dehydration.

New or worsening reflux/regurgitation with severe pain, aspiration, or inability to eat/drink.

Signs of wound infection if you had incisions: increasing redness, warmth, swelling, pus, red streaks, or worsening incision pain.

Leg swelling, calf pain/tenderness, or one leg becoming red/warm, which could suggest a blood clot.

Feeding tube or drain problems if you have one: tube falls out, severe leakage, pus, foul odor, sudden increased drainage, or skin breakdown.

These symptoms can indicate complications such as bleeding, perforation/leak, infection, pneumonia, or blood clots. POEM is generally less invasive, but serious complications can still happen. University of Michigan notes that after POEM, blood/black stool, fever, chills, vomiting, chest or stomach pain, or shortness of breath should prompt contacting your doctor as instructed; discharge guidance for Heller/POEM similarly lists fever, abdominal pain/swelling, black/bloody stools, leg tenderness, chest pain, or breathing problems as warning signs. (uofmhealth.org)

If you're unsure whether a symptom is "bad enough," it's safer to call the surgical team/on-call line-and if breathing, chest pain, fainting, severe bleeding, or severe worsening pain is involved, go to the ER/call 911.

Are you asking about symptoms you're having right now, or are you preparing for recovery instructions after an upcoming POEM/surgery?

**Model Claude Opus 4.7**

Answer text:

Urgent Symptoms After POEM or Esophageal Surgery

After a POEM (Peroral Endoscopic Myotomy) or surgical procedure like Heller myotomy, seek immediate medical care if you experience any of the following:

Call 911 or Go to the ER Immediately

Signs of perforation, leak, or serious infection:

Severe chest, abdominal, back, or shoulder pain (especially worsening)

Fever over 101 F (38.3 C) or shaking chills

Rapid heart rate or difficulty breathing/shortness of breath

Crepitus - crackling sensation under the skin of your neck or chest (subcutaneous emphysema)

Vomiting blood or material that looks like coffee grounds

Black, tarry, or bloody stools

Severe difficulty swallowing, unable to even handle saliva

Signs of sepsis: confusion, dizziness, clammy skin, very low blood pressure

Contact Your Surgeon Urgently (Same Day)

New or worsening chest pain not relieved by usual measures

Persistent vomiting or inability to keep fluids down

Pain not controlled by prescribed medications

Redness, swelling, drainage, or pus at incision sites (after surgery)

Fever 100.4-101 F (38-38.3 C)

New severe heartburn or regurgitation

Calf pain, swelling, or redness (possible blood clot)

Decreased urine output or signs of dehydration

Yellowing of skin or eyes (jaundice)

Why These Matter

Esophageal leak or perforation is the most serious complication and requires prompt treatment

Pneumothorax or pneumomediastinum (air leak into chest cavity) can occur after POEM

Bleeding can present internally before becoming obvious

Infection or abscess needs early antibiotic or drainage treatment

Important Notes

Mild chest discomfort, sore throat, gas/bloating, and small amounts of shoulder pain are common in the first days and usually improve

Always follow the specific discharge instructions from your surgical team - they may have additional warning signs based on your procedure

Keep your surgeon's after-hours contact number readily available

When in doubt, get checked. Complications are uncommon but most treatable when caught early.

This is general information and not a substitute for personalized medical advice. Always contact your healthcare team with concerns about your recovery.

**Model DeepSeek V4 Pro**

Answer text:

Disclaimer: I'm an AI assistant, not a doctor. The following is general information and should not replace direct communication with your medical team. Always follow the discharge instructions given by your surgeon, and if you're unsure, err on the side of caution and seek help.

After a POEM (Peroral Endoscopic Myotomy) or any major esophageal/foregut surgery, certain symptoms can signal a complication that needs immediate medical attention. Call your surgeon, go to the emergency department, or activate emergency services if you experience:

Severe or Worsening Pain

Pain that suddenly becomes intense, especially in the chest, back, abdomen, or shoulders (may signal perforation or leak).

Pain that is not controlled by prescribed medication and keeps getting worse.

Signs of Infection or Sepsis

Fever of 100.4 F (38 C) or higher (some programs use different thresholds; check your instructions).

Shaking chills, confusion, rapid heartbeat, or feeling very unwell.

Redness, warmth, worsening swelling, or purulent drainage from an incision site.

Difficulty Swallowing or Complete Obstruction

Inability to swallow liquids or your own saliva (different from the temporary post-op swelling that is expected).

Sudden complete dysphagia where nothing passes, especially if accompanied by chest pressure or pain.

Evidence of Bleeding

Vomiting bright red blood or material that looks like coffee grounds.

Passing black, tarry stools (melena).

A sudden drop in blood pressure, dizziness, or fainting (signs of significant blood loss).

Respiratory Distress or Chest Symptoms

New-onset shortness of breath, difficulty breathing, or rapid breathing.

Sharp chest pain when breathing in (could indicate a pneumothorax or mediastinal air).

Subcutaneous crepitus (a crackling sensation under the skin of the neck or chest, which may indicate air escaping into tissues).

Other Urgent Neurological or Systemic Changes

Sudden confusion, slurred speech, or one-sided weakness (stroke-like symptoms are rare but possible if air embolism occurs).

Severe nausea and vomiting that won't stop, especially if you see surgical clips in the vomit (extreme rarity but possible with migration).

Signs of Leak or Mediastinitis

Rapid heart rate combined with chest/back pain and fever.

A feeling that something is "very wrong" (patient intuition often picks up early signs of a leak before lab tests).

Why this matters: Complications like a mucosal leak, perforation, or bleeding after POEM are uncommon but serious. Early recognition drastically improves outcomes. If you've had a fundoplication or other surgery, the same principles apply, but also watch for symptoms like inability to burp or vomit when it would normally be expected (gas bloat syndrome can become an emergency in rare cases).

Proactive step: Keep your surgeon's emergency contact number stored in your phone and a written list of your procedure and current medications in your wallet. If you call for help, clearly state: "I am X days post-op from a [POEM/fundoplication], and I'm experiencing..." This gives the triage team critical context.

Have you just had a procedure, or are you preparing for one? I can share more about what a typical recovery timeline looks like and which symptoms are considered normal (versus those that warrant a call) - just let me know.

**Model Gemini 3.1 Pro Thinking**

Answer text:

Disclaimer: I am an AI, not a doctor or medical professional. The following information is for educational purposes and should not replace professional medical advice. If you are currently experiencing any severe symptoms, please call your local emergency number (such as 911) or go to the nearest emergency room immediately.

Peroral Endoscopic Myotomy (POEM) and traditional surgical myotomies (like the Heller myotomy) are generally safe procedures, but they do carry the risk of complications such as bleeding, infection, or esophageal leaks/perforations.

While mild sore throat, minor chest discomfort, and mild bloating are normal in the first few days of recovery, you should seek urgent medical care if you experience any of the following "red flag" symptoms:

1. Severe or Worsening Pain

What to look for: Sudden, sharp, or progressively worsening chest pain, neck pain, or abdominal pain that is not controlled by your prescribed pain medication.

Why it's urgent: This can be a sign of an esophageal leak or perforation, which allows digestive juices and bacteria to enter the chest or abdominal cavity.

2. Breathing Difficulties

What to look for: Shortness of breath, rapid breathing, or pain when taking a deep breath.

Why it's urgent: This could indicate a pneumothorax (collapsed lung), fluid around the lungs (pleural effusion), or severe inflammation in the chest cavity.

3. Signs of Infection

What to look for: A fever of 100.4 F (38 C) or higher, persistent chills, or sweating.

Why it's urgent: Fever is a primary indicator of a developing infection, potentially from a micro-perforation or pneumonia.

4. Bleeding

What to look for: Vomiting bright red blood, vomiting material that looks like dark coffee grounds, or passing black, tarry, or bloody stools.

Why it's urgent: This indicates active internal bleeding from the surgical site in the esophagus or stomach.

5. Swallowing Inability

What to look for: Sudden inability to swallow even your own saliva or clear liquids, leading to choking or regurgitation.

Why it's urgent: While mild swelling is expected, absolute blockage could mean severe edema (swelling), a hematoma (blood pooling), or a structural issue that needs immediate assessment.

6. Crackling Skin (Subcutaneous Emphysema)

What to look for: A strange "crackling" or "bubble wrap" sensation when you press lightly on the skin of your neck or upper chest.

Why it's urgent: This means air has escaped from the esophagus into the tissues under your skin (a sign of an air leak or perforation).

7. Signs of Shock or Sepsis

What to look for: Unexplained dizziness, fainting, severe weakness, confusion, or a very rapid, pounding heartbeat.

Why it's urgent: These are signs that your body is under extreme stress, potentially due to severe blood loss or a systemic infection (sepsis).

Potential Risks & Optimization Suggestions

The Risk of Delay: Delaying care for symptoms like a leak or perforation can lead to mediastinitis (a life-threatening infection of the chest cavity) or sepsis. Time is of the essence when dealing with esophageal complications.

Preparation Suggestion: Before your surgery, explicitly ask your surgical team for their "after-hours" emergency phone number. Save this number in your phone.

ER Suggestion: If you have to go to an Emergency Room, immediately tell the triage nurse: "I recently had esophageal surgery (POEM/Myotomy) and I am at risk for an esophageal leak." This specific phrasing helps ER staff prioritize your care and order the correct imaging (like a CT scan with oral contrast).

Are you currently recovering from a POEM procedure and experiencing discomfort, or are you in the process of gathering information to prepare for an upcoming surgery?

**Model Grok-4.3**

Answer text:

I am not a doctor, and this is not personalized medical advice. The information below is general guidance drawn from standard medical literature on complications after Peroral Endoscopic Myotomy (POEM) or similar esophageal/upper-GI procedures. Your surgical team's discharge instructions and emergency contact numbers are the definitive source for you. When in doubt, contact your surgeon, gastroenterologist, or go to the nearest emergency department-do not wait to "see if it gets better."

Symptoms That Should Prompt Urgent Medical Care (Call your doctor or go to ER)

Seek immediate evaluation for any of the following:

Severe or rapidly worsening chest, neck, shoulder, back, or upper abdominal pain that is not relieved by your prescribed pain medication.

Fever (typically at least 100.4 F / 38 C) or chills, which may indicate infection or perforation.

Shortness of breath, rapid breathing, or difficulty breathing - even at rest.

Vomiting blood, coffee-ground material, or bright red blood from the mouth.

Black, tarry stools (melena) or large amounts of bright red blood in the stool - signs of gastrointestinal bleeding.

Dizziness, lightheadedness, fainting, rapid heartbeat, or feeling like your heart is racing (possible signs of bleeding, dehydration, or systemic response).

Subcutaneous emphysema - a crackling, "Rice Krispies" sensation under the skin of the neck, chest, or upper abdomen (can indicate air leaking from the esophagus).

Inability to swallow saliva or liquids, excessive drooling, or complete blockage feeling that is worse than before the procedure.

Persistent vomiting that prevents you from keeping down fluids for more than a few hours.

Swelling, firmness, or severe bloating of the abdomen that is painful.

Confusion, extreme fatigue, or feeling generally "very wrong" (can be an early sign of sepsis or significant complication).

Additional Guidance

Mild chest discomfort, sore throat, low-grade temperature ( less than 100.4 F), or small amounts of air under the skin can be common in the first 24-48 hours after POEM and may be monitored as directed by your team. However, if these are increasing rather than improving, treat them as urgent.

Most serious complications (perforation, bleeding, mediastinitis, pneumothorax) are rare but can develop or worsen in the first 1-7 days.

Have your surgeon's after-hours number and the closest hospital with gastroenterology or thoracic surgery capability ready.

Err on the side of caution. It is far better to be evaluated and told everything is fine than to delay care for a developing complication.

If you have your discharge paperwork handy, compare these symptoms against the specific "call us immediately" list your team gave you. Would you like help understanding any particular part of typical post-POEM recovery instructions, or are you currently experiencing one of these symptoms and need guidance on next steps right now?

**Model Qwen3-Max**

Answer text:

After undergoing a Peroral Endoscopic Myotomy (POEM) or other esophageal surgery (such as Heller myotomy), it's important to monitor for signs of complications. While some discomfort, mild pain, or temporary difficulty swallowing is expected during recovery, certain symptoms require urgent medical attention because they may indicate serious complications like infection, perforation, bleeding, or pneumothorax.

Seek immediate medical care if you experience any of the following:

1. Fever greater than 101.5 F (38.6 C)

May signal infection (e.g., mediastinitis or abscess).

2. Severe or worsening chest/abdominal pain

Especially if sudden, sharp, or not relieved by prescribed pain medication.

Could indicate perforation, leakage, or internal injury.

3. Difficulty breathing or shortness of breath

May suggest pneumothorax (collapsed lung), pleural effusion, or aspiration.

4. Persistent nausea/vomiting (especially with blood or coffee-ground material)

Could indicate bleeding, obstruction, or mucosal injury.

5. Signs of gastrointestinal bleeding

Vomiting bright red blood or material that looks like coffee grounds.

Black, tarry stools (melena).

Dizziness, lightheadedness, or rapid heart rate (signs of significant blood loss).

6. Inability to tolerate any liquids

If you can't keep even small sips of water down beyond the first 24-48 hours post-op (as per your surgeon's instructions), this may suggest stricture, edema, or dysmotility requiring evaluation.

7. Swelling, redness, or drainage at incision sites (if applicable)

Though POEM is incision-free, some surgeries (like laparoscopic Heller) involve small incisions-signs of infection here need assessment.

8. Rapid heart rate, low blood pressure, or signs of shock

Cold/clammy skin, confusion, fainting-these are medical emergencies.

Important Notes:

POEM-specific risks: Although minimally invasive, POEM carries a small risk of mucosal perforation, subcutaneous emphysema (air under the skin-often harmless but sometimes a sign of larger leak), or gastroesophageal reflux exacerbation.

Always follow your surgical team's specific post-op instructions-they may provide a personalized list of warning signs.

If in doubt, contact your surgeon or go to the nearest emergency department. Early intervention can prevent serious outcomes.

Next step: Do you have a copy of your surgeon's post-operative instruction sheet? Reviewing it alongside this list can help you recognize what's normal vs. urgent in your specific case. Would you like help interpreting any part of those instructions?

**Q39. Does achalasia increase the risk of esophageal cancer?**

**Model ChatGPT 5.5**

Answer text:

Yes. Achalasia does increase the risk of esophageal cancer, especially esophageal squamous cell carcinoma. The risk of esophageal adenocarcinoma may also be increased, but that link is less consistent and may be partly related to reflux/Barrett's esophagus after achalasia treatment. (pmc.ncbi.nlm.nih.gov)

That said, the absolute risk is still low for most people. For example, one long-term prospective cohort found an annual esophageal cancer incidence of about 0.24 percent per year in patients with achalasia, with squamous cell cancer more common than adenocarcinoma. (pubmed.ncbi.nlm.nih.gov) Another matched cohort study found esophageal cancer incidence of 1.4 per 1,000 person-years and an overall risk about 4.6 times higher than in people without achalasia. (pmc.ncbi.nlm.nih.gov)

Why the risk rises: achalasia can cause long-term food and fluid stasis in the esophagus, leading to irritation, bacterial overgrowth, and chronic inflammation. Treatments that reduce lower esophageal sphincter pressure can also increase reflux risk, which may contribute to Barrett's esophagus/adenocarcinoma in some patients.

Screening/surveillance: this is an area without universal agreement. Some specialists consider periodic upper endoscopy in long-standing achalasia-often after 10-15 years, especially in men, older patients, smokers, heavy alcohol users, or those with persistent symptoms-but routine surveillance for everyone is not uniformly recommended. Current practice is individualized. (giboardreview.com)

You should contact a gastroenterologist promptly if you have worsening swallowing after prior stability, unexplained weight loss, vomiting/regurgitation, bleeding, anemia, chest pain, or new pain with swallowing, because those symptoms may warrant endoscopy.

Bottom line: achalasia raises esophageal cancer risk, mainly squamous cell carcinoma, but cancer remains uncommon; surveillance decisions should be personalized with your GI doctor based on duration of disease, symptoms, risk factors, and prior endoscopy findings.

Do you know how long you've had achalasia and whether you've had treatment such as pneumatic dilation, Heller myotomy, or POEM?

**Model Claude Opus 4.7**

Answer text:

Yes, achalasia is associated with an increased risk of esophageal cancer, though the absolute risk remains relatively low.

Key Points

Type of cancer: Primarily squamous cell carcinoma (SCC) of the esophagus, though the risk of adenocarcinoma is also modestly elevated.

Magnitude of risk:

Studies suggest a 10- to 50-fold increased risk of esophageal SCC compared to the general population

Absolute incidence is approximately 300 cases per 100,000 patient-years

Lifetime risk is estimated around 1-5 percent

Mechanism: Chronic stasis of food and fluids in the dilated esophagus leads to:

Prolonged mucosal exposure to irritants and bacterial overgrowth

Chronic inflammation and esophagitis

Progression through dysplasia to carcinoma

Timing: Cancer typically develops 15-25 years after symptom onset, which is why many cases are diagnosed at an advanced stage.

Clinical Implications

Treatment of achalasia (pneumatic dilation, Heller myotomy, POEM) does not eliminate the cancer risk, since stasis often persists

Surveillance endoscopy is not universally recommended due to low absolute risk, but some guidelines suggest considering it in patients with long-standing disease ( greater than 10-15 years)

New or worsening symptoms (dysphagia changes, weight loss, bleeding) in a patient with established achalasia should prompt endoscopic evaluation

Would you like more detail on surveillance recommendations or the pathophysiology?

**Model DeepSeek V4 Pro**

Answer text:

Yes, achalasia is associated with an increased risk of esophageal cancer, particularly squamous cell carcinoma (SCC). The connection has been recognized for decades, though the absolute risk remains low enough that it isn't considered a common outcome.

Why does achalasia raise cancer risk?

In achalasia, the lower esophageal sphincter doesn't relax properly, and the esophagus loses normal peristalsis. This causes food, saliva, and secretions to linger in the esophagus for long periods. Over time, this chronic stasis leads to:

Continuous irritation and inflammation of the esophageal lining.

Bacterial overgrowth and chemical changes that can promote dysplasia.

Eventually, the development of squamous cell carcinoma (sometimes also adenocarcinoma, but SCC is far more typical).

The latent period is long: cancer usually appears 10-20+ years after symptom onset, and the risk is higher in untreated or inadequately treated megaesophagus.

What's the actual risk?

Estimates vary across studies, but the general picture is:

A 10- to 50-fold increased risk of esophageal squamous cell cancer compared to the general population.

Lifetime risk is often cited in the range of 0.3 percent -1.5 percent per year of long-standing disease, with cumulative incidences of around 3 percent -10 percent over decades in higher-risk groups.

The risk is especially elevated in patients who have had achalasia for many years without treatment that sufficiently relieves the obstruction.

Does treatment reduce the risk?

Effective therapies that relieve outflow obstruction-like Heller myotomy, peroral endoscopic myotomy (POEM), or pneumatic dilation-are thought to reduce, but not eliminate, the elevated cancer risk. The oesophagus has already been exposed to years of stasis before treatment, and some residual inflammation can persist. That's why surveillance endoscopy is still a topic of discussion even after successful intervention.

Surveillance recommendations (as of 2024-2026)

There's no universal consensus, but many experts and guidelines (e.g., American Society for Gastrointestinal Endoscopy, European guidelines) suggest:

Start endoscopic surveillance about 10-15 years after symptom onset (or possibly earlier if long-standing megaesophagus is present).

Repeat endoscopy every 2-5 years, depending on individual risk (e.g., age, duration of symptoms, response to treatment).

During endoscopy, careful inspection with high-definition white light and/or chromoendoscopy (like Lugol's iodine staining) is recommended to detect early dysplasia or carcinoma.

Biopsy any suspicious areas, and some also advocate random four-quadrant biopsies every 2-3 cm.

Risk optimization and caveats

You cannot self-surveil. If you (or someone) has achalasia, regular follow-up with a gastroenterologist is essential. Discuss a personalized surveillance plan.

Watch for red-flag symptoms that warrant immediate investigation: new or worsening dysphagia, unintentional weight loss, chest pain, or signs of anemia/blood loss.

Keep in mind that cancer can still develop in the small remaining pouch after myotomy, so ongoing awareness remains important.

Note: This is general medical information, not a substitute for professional medical advice. If you're concerned about achalasia or cancer risk, consult a specialist.

Follow-up step to continue the dialog: Would you like me to outline the specific symptoms of esophageal cancer that achalasia patients should watch for, or explore the pros and cons of different surveillance techniques (like chromoendoscopy vs. narrow-band imaging)?

**Model Gemini 3.1 Pro Thinking**

Answer text:

Disclaimer: I am an AI, not a doctor. The following information is for educational purposes and should not replace professional medical advice. Always consult with a gastroenterologist or healthcare provider for diagnosis, risk assessment, and treatment.

Yes, achalasia does increase the risk of developing esophageal cancer.

Here is a breakdown of why this happens, the level of risk, and how it is managed:

Why does achalasia increase the risk?

Achalasia is a rare disorder where the nerves in the esophagus become damaged. This prevents the lower esophageal sphincter (the valve between the esophagus and stomach) from relaxing, making it very difficult for food and liquids to pass through.

Because the food cannot easily enter the stomach, it pools and ferments in the esophagus. Over years or decades, this chronic retention of food and stomach acids causes severe, long-term inflammation and irritation of the esophageal lining. This chronic inflammation can cause the cells to mutate over time, leading to cancer.

What type of cancer?

The most common type of esophageal cancer associated with achalasia is squamous cell carcinoma, which affects the flat cells lining the inside of the esophagus. There is also a slightly increased risk for adenocarcinoma.

How high is the risk?

It is important to understand the difference between relative risk and absolute risk:

Relative Risk: People with achalasia have a significantly higher risk compared to the general population (some studies estimate the risk is 10 to 50 times higher).

Absolute Risk: Despite the high relative risk, the actual chance of any single individual with achalasia getting esophageal cancer remains relatively low. It is estimated that roughly 1 percent to 3 percent of people with achalasia will develop esophageal cancer.

This cancer typically develops slowly, usually appearing 10 to 15 years (or more) after the initial symptoms of achalasia begin.

Prevention and Surveillance

Because of this elevated risk, gastroenterologists usually recommend routine surveillance. This typically involves undergoing a regular upper endoscopy (an examination of the esophagus using a camera) starting 10 to 15 years after the onset of achalasia symptoms. This allows doctors to detect any precancerous changes (dysplasia) or early-stage cancer when it is highly treatable. Treating the underlying achalasia (through dilation, surgery, or Botox) to empty the esophagus is also crucial to reducing chronic inflammation.

Are you or a loved one currently managing achalasia, and would you like to know more about the standard treatment options used to improve swallowing and reduce this esophageal irritation?

**Model Grok-4.3**

Answer text:

Yes, achalasia does increase the risk of esophageal cancer, particularly esophageal squamous cell carcinoma (and to a lesser extent adenocarcinoma).

Key Evidence and Risk Magnitude

Multiple large cohort studies and meta-analyses show a significantly elevated risk.

The standardized incidence ratio (SIR) for esophageal cancer in patients with achalasia is typically reported between 6-fold to 33-fold higher than the general population, with most modern studies clustering around 10- to 16-fold increased risk.

A 2016 meta-analysis (and subsequent studies) confirmed a pooled SIR of approximately 12-14 times higher.

The risk appears to increase with disease duration - it is notably higher after 10-15+ years of untreated or poorly controlled achalasia.

Why Does This Happen?

Long-standing achalasia causes stasis of food and saliva in the dilated esophagus. This leads to:

Chronic inflammation

Bacterial overgrowth

Production of nitrosamines and other carcinogens

Repeated mucosal trauma and dysplasia over time

This creates a field effect that favors malignant transformation, mostly to squamous cell carcinoma (the predominant histologic type associated with achalasia, unlike Barrett's esophagus which leads to adenocarcinoma).

Clinical Implications

Most gastroenterology societies (AGA, ASGE, etc.) consider achalasia a pre-malignant condition.

Surveillance endoscopy is often recommended starting 8-10 years after symptom onset, though the optimal interval is still debated (commonly every 2-3 years once surveillance begins).

The absolute risk remains relatively low (lifetime risk is still only a few percent in most studies), so the vast majority of achalasia patients will never develop cancer. However, the relative risk is high enough to warrant discussion of surveillance.

Bottom line: Achalasia is a recognized risk factor for esophageal cancer (mainly squamous cell type), with a roughly 10-15 x increased risk that rises with longer disease duration. Good control of achalasia (via myotomy, POEM, or effective dilation) and surveillance in long-standing cases are the main strategies to mitigate this risk.

Follow-up question: How long have you (or the person you're asking about) had achalasia symptoms, and are you currently under any form of treatment or surveillance?

**Model Qwen3-Max**

Answer text:

Yes, achalasia does increase the risk of esophageal cancer, particularly esophageal squamous cell carcinoma (ESCC).

Why the Increased Risk?

Achalasia is a motility disorder characterized by:

Failure of the lower esophageal sphincter (LES) to relax during swallowing

Absence of normal peristalsis in the esophageal body

This leads to:

Chronic stasis of food and secretions in the esophagus

Persistent mucosal irritation and inflammation

Possible bacterial overgrowth and production of carcinogenic compounds (e.g., nitrosamines)

Long-standing dilation of the esophagus (megaesophagus)

Over time, these changes can promote dysplasia and malignant transformation.

Magnitude of Risk

Studies suggest patients with achalasia have a 16- to 28-fold higher risk of developing esophageal cancer compared to the general population.

The lifetime risk is estimated at 2-7 percent , typically after 10-20 years of disease duration.

Most cases occur more than 15-25 years after diagnosis.

Clinical Implications

Because of this elevated risk:

Routine surveillance endoscopy is often recommended in long-standing achalasia (e.g., greater than 15-20 years), though guidelines vary.

Endoscopy should assess for dysplasia, strictures, ulcers, or masses.

Biopsies are indicated if mucosal abnormalities are seen.

Note: While the relative risk is high, the absolute risk remains relatively low. Nonetheless, awareness and monitoring are important in long-term management.

Would you like guidance on current surveillance recommendations or how achalasia is managed to potentially mitigate cancer risk?

**Q40. What should people with achalasia eat?**

**Model ChatGPT 5.5**

Answer text:

People with achalasia usually do best with foods that are soft, moist, easy to swallow, and nutrient-dense. Diet won't cure achalasia, but it can reduce "food sticking," regurgitation, coughing, and weight loss while medical treatment is being planned or after treatment. Achalasia causes poor movement of food through the esophagus and impaired relaxation of the lower esophageal sphincter, so symptoms often include dysphagia to both solids and liquids, regurgitation, chest pain, cough, and weight loss. (sysge.org)

Foods that are often easier to eat

Soft, moist proteins

Scrambled eggs or soft omelets

Fish, soft chicken, turkey, or meat with gravy/sauce

Tofu, hummus, bean dips

Greek yogurt, cottage cheese, soft cheese

Smooth nut butters thinned into smoothies or oatmeal

Soft carbohydrates

Oatmeal, cream of wheat, grits

Mashed potatoes or sweet potatoes

Soft rice, risotto, pasta with sauce

Pancakes or soft breads soaked with soup/sauce if tolerated

Cooked fruits and vegetables

Applesauce, bananas, canned peaches/pears

Well-cooked carrots, squash, zucchini, spinach

Pur ed vegetable soups

Smoothies without tough skins/seeds

High-calorie liquids if weight loss is an issue

Smoothies with yogurt, protein powder, nut butter, avocado, or milk

Meal-replacement shakes

Cream soups

Fortified milk or lactose-free alternatives

The American Gastroenterological Association's patient nutrition guidance recommends a soft-textured diet, with pur ed or liquid diets sometimes needed in more severe cases. (patient.gastro.org)

Foods that commonly cause problems

These vary by person, but many people with achalasia struggle with:

Dry meats, steak, chicken breast without sauce

Dry bread, bagels, crackers, rice cakes

Raw vegetables, salads, corn, apple skins

Tough or stringy foods

Nuts, seeds, popcorn

Sticky foods like peanut butter on bread

Very spicy, acidic, or greasy foods if they worsen reflux-like symptoms

Carbonated drinks if they cause bloating/regurgitation

Eating habits that help

Eat small, frequent meals instead of large meals.

Take small bites and chew very thoroughly.

Sip water or warm liquids during meals to help food pass.

Eat slowly and avoid rushing.

Stay upright during meals and for at least 2-3 hours afterward.

Avoid eating close to bedtime.

Consider raising the head of the bed if nighttime regurgitation occurs.

Keep a food/symptom diary, because triggers differ widely between people.

When to call a clinician urgently

Seek medical advice promptly if there is unintentional weight loss, dehydration, inability to keep liquids down, choking, recurrent coughing with meals, fever, chest pain, or possible aspiration. Achalasia can lead to malnutrition and aspiration pneumonia, and effective treatments include pneumatic dilation, botulinum toxin injection, Heller myotomy, and POEM, depending on the case. (my.clevelandclinic.org)

A good next step is to ask a gastroenterologist or registered dietitian for a personalized plan-especially if weight loss or frequent regurgitation is happening.

**Model Claude Opus 4.7**

Answer text:

Eating with Achalasia: Dietary Guidelines

Achalasia is a condition where the lower esophageal sphincter doesn't relax properly, making swallowing difficult. While diet doesn't cure achalasia, certain eating strategies can significantly reduce symptoms.

Foods That Are Generally Easier to Tolerate

Soft, moist foods:

Soups, broths, and stews

Mashed potatoes

Scrambled eggs

Yogurt, pudding, custard

Smoothies and milkshakes

Oatmeal and cream of wheat

Well-cooked pasta with sauce

Soft-cooked or flaked fish

Ground or pureed meats

Ripe, soft fruits (bananas, melon)

Cooked vegetables (well-softened)

Foods to Limit or Avoid

Dry, tough meats (especially steak)

Bread, especially fresh or doughy

Rice (can clump in the esophagus)

Raw, fibrous vegetables

Tough fruit skins and stringy fruits

Nuts, seeds, and granola

Sticky foods like peanut butter

Carbonated drinks (can cause discomfort for some)

Very hot or very cold foods (may trigger spasms)

Alcohol and caffeine (may worsen symptoms in some)

Helpful Eating Strategies

Eat slowly and chew thoroughly until food is nearly liquid

Take small bites and small sips

Drink plenty of water with meals to help food pass

Eat smaller, more frequent meals (5-6 per day) instead of large ones

Sit upright while eating and for 1-2 hours afterward

Avoid eating within 2-3 hours of bedtime to reduce regurgitation risk

Try sparkling water (some find it helps push food through, though others find it uncomfortable-experiment)

Stand up and walk after meals to use gravity

When to Seek Help

Consult a gastroenterologist or registered dietitian if you experience:

Significant weight loss

Frequent regurgitation or choking

Chest pain or heartburn that doesn't improve

Difficulty maintaining nutrition

A dietitian can create a personalized plan, and you may benefit from nutritional supplements if you're struggling to maintain weight. Treatments like pneumatic dilation, POEM, or Heller myotomy may also be discussed with your doctor.

Note: Tolerance varies considerably between individuals-keeping a food diary can help you identify your personal triggers and safe foods.

**Model DeepSeek V4 Pro**

Answer text:

Achalasia makes swallowing difficult because the esophagus loses its normal squeezing motion and the lower sphincter stays too tight. The right food choices and eating habits can significantly reduce symptoms, prevent food getting stuck, and lower the risk of aspiration (food entering the airway) or malnutrition. Below is a practical guide-but please tailor it with your own doctor or dietitian, as needs vary.

What to Eat: Soft, Moist & Easy-to-Swallow Foods

Choose foods that are naturally smooth, high in moisture, or can be blended. Think "slides down easily":

Dairy & alternatives: Yogurt, kefir, cottage cheese, pudding, custard, smooth cream cheese, milk, milkshakes, nutrition shakes (like Ensure or Boost)

Grains: Cream of wheat/rice, oatmeal (well-cooked and thinned), polenta, soft pancakes moistened with syrup, well-cooked pasta (soft, not al dente), soft bread soaked in soup or sauce

Proteins: Scrambled eggs, omelets, soft tofu, hummus, refried beans, moist flaked fish (salmon, tuna mashed with mayo), ground meat in gravy, pureed meats

Fruits & vegetables: Applesauce, mashed banana, avocado, pureed vegetables (carrots, squash), smoothies, vegetable soups that are blended or clear broth without large chunks

Liquids & semi-liquids: Broths, cream soups (pureed, not with chewy bits), warm or cold teas, water sipped throughout the meal

What to Avoid or Use with Caution

These foods tend to be dry, fibrous, sticky, or tough, making them high-risk for impaction (stuck sensation):

Dry bread, bagels, crackers, dry cereal, granola

Tough meats (steak, pork chops), dry poultry, hot dogs unless finely minced and moistened

Raw vegetables (carrot sticks, celery), salad greens, corn, peas with skins

Fruits with membranes (oranges, grapefruit), dried fruit, whole nuts and seeds, popcorn, chips

Sticky rice, thick nut butters eaten by the spoonful, very dry mashed potatoes without gravy

Eating Techniques That Make a Big Difference

Posture is important: Sit fully upright for every meal and remain upright for at least 30-60 minutes after eating. Use gravity to help food pass.

Take tiny bites: Cut solid food into pea-sized pieces, or spoon-feed purees in small amounts.

Chew thoroughly: Even soft foods need to become a liquid-like paste before swallowing.

Chin tuck: When swallowing, slightly tuck your chin toward your chest-this helps protect the airway and can make swallowing easier.

Drink small sips between bites: Water or a warm beverage (many find warm liquids relaxing) can help wash food down. Some people prefer carbonated drinks, which may temporarily open the sphincter, but they can also cause bloating; try them cautiously.

Slow down: It may take 30-60 minutes for a full meal. Rushing increases choking and discomfort.

Preventing Malnutrition

Weight loss is common with achalasia. To keep calories and nutrients up:

Fortify foods: Add nut butter, avocado, olive oil, protein powder, or full-fat dairy to smoothies, soups, and mashed potatoes.

Use nutrient-dense liquids: Sip high-protein, high-calorie shakes throughout the day if solid meals are too challenging.

Consult a registered dietitian: They can help craft a meal plan that meets your energy needs safely, including a texture-modified diet if necessary.

After Treatment

If you've had a pneumatic dilation, POEM, or Heller myotomy, your doctor will usually prescribe a staged diet: clear liquids to full liquids to soft foods to gradual return to a more regular (but still careful) diet over days or weeks. Always follow that progression exactly.

Warning Signs to Report

If you frequently cough, choke, or feel food "going down the wrong pipe," aspiration pneumonia is a real risk. Also, if you rapidly lose weight, have chest pain, or regurgitate undigested food often, talk to your doctor-it may signal that your current management needs adjusting.

Would you like to explore recipe ideas for a soft/easy-swallowing diet, or learn about the medical treatments that can improve swallowing function in achalasia?

**Model Gemini 3.1 Pro Thinking**

Answer text:

Key Takeaway For individuals living with achalasia, the primary dietary goal is to minimize swallowing difficulties (dysphagia) and prevent nutritional deficiencies. A semi-liquid, soft, or pureed diet combined with specific eating behaviors is highly recommended to help food pass more easily into the stomach.

Diet Guidelines: What to Eat vs. What to Avoid

Managing achalasia requires choosing foods that do not easily get stuck in the esophagus. Below is a structured guide on food selection:

Food Category Highly Recommended (Easy to Swallow) Avoid or Limit (High Risk of Stuck Food)

Proteins Silken tofu, scrambled eggs, bone broth, slow-cooked flaked fish, smooth yogurt, protein shakes. Tough meats (steak, pork chops), dry chicken breast, hard-boiled eggs.

Grains & Starches Well-cooked oatmeal, congee, mashed potatoes, sweet potato puree, polenta. Fresh bread (especially doughy or white bread), sticky rice, dry crackers, popcorn.

Vegetables Pureed vegetable soups (carrots, squash), well-cooked and mashed vegetables without skins. Raw vegetables (celery, carrots), fibrous greens (broccoli, kale), corn, peas.

Fruits Applesauce, ripe bananas, fruit smoothies (strained to remove seeds), canned peaches in juice. Raw apples, fibrous fruits (pineapple), fruits with tough skins or tiny seeds (berries).

Dairy & Alternatives Milk, kefir, cottage cheese, smooth puddings, custard. Ice cream with solid mix-ins (nuts, chocolate chips), thick chewy cheeses.

Crucial Eating Habits and Techniques

How you eat is often just as important as what you eat when managing achalasia.

Technique How It Helps

Eat Upright Sit completely straight while eating and remain upright for at least 2 hours after meals to let gravity assist passage.

Take Tiny Bites & Chew Thoroughly Mechanically breaking down food as much as possible reduces the burden on your esophagus.

Sip Warm Fluids Drinking warm water or warm broth during meals can help relax the lower esophageal sphincter (LES).

Eat Small, Frequent Meals Consuming 5-6 small meals instead of 3 large ones prevents food from backing up and causing discomfort.

Common Pitfalls & Risks to Watch Out For

Avoid Cold Beverages during Meals: Cold liquids can cause the esophageal muscles to spasm, worsening swallowing difficulties. Stick to room temperature or warm liquids.

Late-Night Eating: Eating close to bedtime increases the risk of nocturnal regurgitation, coughing, and aspiration. Stop eating at least 3 to 4 hours before lying down.

Nutritional Deficiencies: Because a soft diet can limit variety, keep an eye on your caloric and nutrient intake. Consider working with a registered dietitian to ensure you are getting enough protein, vitamins, and minerals.

Would you like some specific recipe ideas for nutrient-dense smoothies, or are you interested in learning about medical treatments that can make eating easier?

**Model Grok-4.3**

Answer text:

People with achalasia should focus on soft, moist, easy-to-swallow foods that minimize the effort required for the esophagus to move food downward. Because the lower esophageal sphincter doesn't relax properly, the goal is to reduce food retention, regurgitation, chest pain, and the risk of aspiration.

General Dietary Principles

Texture: Soft, moist, or pureed foods are best. Many patients do well with "minced-moist" or "pureed" textures (similar to IDDSI Level 4-6).

Eating habits: Eat very slowly, chew thoroughly (even soft foods), take small bites, and sip fluids with every bite to help wash food down.

Meal size: Small, frequent meals (5-6 per day) instead of three large ones.

Position: Remain upright for at least 30-60 minutes after eating. Elevate the head of your bed 6-8 inches at night.

Temperature: Most people tolerate room-temperature or warm foods better than very hot or ice-cold items.

Avoid: Foods that are dry, tough, fibrous, sticky, or bulky.

Recommended Foods (Generally Well-Tolerated)

Proteins

Poached, steamed, or baked fish (flaky)

Ground or minced chicken/turkey moistened with gravy or sauce

Scrambled or soft-boiled eggs

Smooth cottage cheese, ricotta, or Greek yogurt

Tofu or soft-cooked legumes that have been thoroughly mashed

Fruits & Vegetables

Bananas, ripe avocados, applesauce, melon

Cooked and mashed carrots, zucchini, squash, potatoes

Pureed vegetables in soups

Smoothies (strained if necessary)

Starches & Cereals

Oatmeal, cream of wheat, or other well-cooked hot cereals

Mashed potatoes with plenty of gravy or butter

Soft pasta or noodles in soup/broth

White rice that is very soft and moist

Soups & Liquids

Broth-based or pureed soups (blended smooth)

Nutritional shakes (Ensure, Boost, Kate Farms, etc.)

Thin liquids if tolerated; some patients need thickened liquids

Desserts

Pudding, custard, flan, yogurt, smooth ice cream/sorbet (if temperature is tolerated)

Foods to Avoid or Limit

Tough, chewy meats (steak, pork chops)

Dry or crusty breads, bagels, toast

Raw vegetables and fruits with skins

Nuts, seeds, popcorn, granola

Sticky foods (peanut butter, caramel, marshmallows)

Fibrous vegetables (celery, broccoli florets, asparagus)

Carbonated beverages (they can cause bloating and increase pressure)

Very spicy, acidic, or highly seasoned foods if they trigger pain or reflux

Alcohol and caffeine (they can relax the sphincter further or irritate)

Additional Management Tips

Many patients benefit from blending meals or using a baby-food grinder for meats and vegetables in the early stages or during flares.

Some require temporary pureed or liquid diets after procedures (Botox, dilation, POEM, Heller myotomy).

Work with a registered dietitian who specializes in esophageal motility disorders or dysphagia. They can tailor the diet to your specific manometry results and tolerances.

Nutritional supplements are often needed because weight loss is common.

Important Disclaimer: This is general guidance based on standard medical recommendations (Mayo Clinic, Cleveland Clinic, and gastroenterology society guidelines). Achalasia affects each person differently, and dietary needs can change over time or after treatment. Please consult your gastroenterologist and a dysphagia-specialized dietitian or speech-language pathologist before making significant dietary changes. If you are losing weight, having frequent choking episodes, or regurgitating food, seek medical care promptly.

Follow-up question to help you further: What symptoms are you experiencing most right now (e.g., trouble with solids, liquids, pain, significant weight loss), and have you had any procedures or seen a dietitian yet? This will allow me to give more targeted suggestions.

**Model Qwen3-Max**

Answer text:

People with achalasia-a disorder where the lower esophageal sphincter (LES) fails to relax properly, making it difficult for food to pass into the stomach-should follow a modified diet to ease swallowing, reduce discomfort, and prevent complications like aspiration or malnutrition. While dietary changes don't cure achalasia, they can significantly improve quality of life.

General Dietary Guidelines for Achalasia:

1. Choose Soft, Moist, and Easily Digestible Foods

Cooked vegetables (e.g., steamed carrots, zucchini)

Tender meats (ground or finely chopped; moistened with gravy or sauce)

Soft fruits (bananas, peeled peaches, applesauce)

Soups, stews, and broths

Smooth nut butters (in moderation)

Yogurt, pudding, oatmeal, and mashed potatoes

2. Avoid Tough, Dry, or Fibrous Foods

Raw vegetables (e.g., celery, broccoli stems)

Tough cuts of meat

Bread with crusts or dry bread products (can form a bolus that gets stuck)

Nuts, seeds, and popcorn

Stringy foods like pineapple or mango fibers

3. Eat Small, Frequent Meals

Smaller portions reduce pressure on the esophagus.

Chew food thoroughly (even soft foods).

4. Drink Plenty of Fluids with Meals

Sip water or other liquids while eating to help food pass through the esophagus.

Avoid very hot or very cold liquids if they trigger spasms.

5. Stay Upright During and After Eating

Remain upright for at least 30-60 minutes after meals to use gravity to help food reach the stomach.

Avoid lying down immediately after eating to reduce risk of regurgitation or aspiration.

6. Consider Texture Modifications

Pureed or blended meals may be necessary in advanced cases.

Use a blender to soften solid meals into soups or smoothies.

7. Limit or Avoid Alcohol and Caffeine

These can relax the LES too much or irritate the esophagus, potentially worsening symptoms like reflux (especially if you've had treatment like balloon dilation or myotomy).

Important Notes:

Individual tolerance varies: Keep a food diary to identify personal triggers.

Weight loss is common in achalasia due to reduced intake-prioritize calorie-dense soft foods (e.g., avocado, full-fat dairy, smoothies with protein powder).

Work with a healthcare team: A gastroenterologist and registered dietitian can tailor recommendations based on disease severity and treatment history (e.g., post-Heller myotomy or POEM procedure).

If you experience frequent regurgitation, chest pain, or unintended weight loss, consult your doctor promptly, as these may indicate disease progression or complications.

Would you like a sample one-day meal plan for someone with achalasia?
